# Molecular Genetic Features of Polyploidization and Aneuploidization Reveal Unique Patterns for Genome Duplication in Diploid *Malus*

**DOI:** 10.1371/journal.pone.0029449

**Published:** 2012-01-10

**Authors:** Michael J. Considine, Yizhen Wan, Mario F. D'Antuono, Qian Zhou, Mingyu Han, Hua Gao, Man Wang

**Affiliations:** 1 College of Horticulture, Northwest A&F University, Yangling, Shaanxi, People's Republic of China; 2 School of Plant Biology, and the Institute of Agriculture, University of Western Australia, Crawley, Western Australia, Australia; 3 Department of Agriculture and Food Western Australia, South Perth, Western Australia, Australia; University of Missouri, United States of America

## Abstract

Polyploidization results in genome duplication and is an important step in evolution and speciation. The *Malus* genome confirmed that this genus was derived through auto-polyploidization, yet the genetic and meiotic mechanisms for polyploidization, particularly for aneuploidization, are unclear in this genus or other woody perennials. In fact the contribution of aneuploidization remains poorly understood throughout Plantae. We add to this knowledge by characterization of eupolyploidization and aneuploidization in 27,542 F_1_ seedlings from seven diploid *Malus* populations using cytology and microsatellite markers. We provide the first evidence that aneuploidy exceeds eupolyploidy in the diploid crosses, suggesting aneuploidization is a leading cause of genome duplication. Gametes from diploid *Malus* had a unique combinational pattern; ova preserved euploidy exclusively, while spermatozoa presented both euploidy and aneuploidy. All non-reduced gametes were genetically heterozygous, indicating first-division restitution was the exclusive mode for *Malus* eupolyploidization and aneuploidization. Chromosome segregation pattern among aneuploids was non-uniform, however, certain chromosomes were associated for aneuploidization. This study is the first to provide molecular evidence for the contribution of heterozygous non-reduced gametes to fitness in polyploids and aneuploids. Aneuploidization can increase, while eupolyploidization may decrease genetic diversity in their newly established populations. Auto-triploidization is important for speciation in the extant *Malus*. The features of *Malus* polyploidization confer genetic stability and diversity, and present heterozygosity, heterosis and adaptability for evolutionary selection. A protocol using co-dominant markers was proposed for accelerating apple triploid breeding program. A path was postulated for evolution of numerically odd basic chromosomes. The model for *Malus* derivation was considerably revised. Impacts of aneuploidization on speciation and evolution, and potential applications of aneuploids and polyploids in breeding and genetics for other species were evaluated in depth. This study greatly improves our understanding of evolution, speciation, and adaptation of the *Malus* genus, and provides strategies to exploit polyploidization in other species.

## Introduction

Whole or partial genome duplication can result from eupolyploidization and aneuploidization; first- and second-division restitutions are their primary paths [1]–[12]. Sexual polyploidization is more reproductively stable than somatic means, and has been a major route for evolution, speciation and domestication [13]–[22]. Between 30–70% of extant plant species are polyploids [1], [6], [8], [9], [13], [19], [20], [22]–[24]. Up to 15% of angiosperm speciation is associated with paleo-polyploidization [1]–[6], [10], [12], [16]–[18]. And it is being increasingly confirmed by genomics [4], [6], and supported by the recent release of *Malus* genome [7]. This contests historical views that neopolyploidy is low among woody perennials due to cambium formation constraining genomic duplication [2], [8]. Our current knowledge of the polyploidization meiotic mechanisms is drawn almost entirely from annual herbaceous species, notably bread wheat (*Triticum aestivum*), *Arabidopsis thaliana*, or oilseed rape (*Brassica napus*) [1], [5], [8], [14], [22], [25]–[27]. Similar investigation in woody perennials has been problematic owing to long generation cycles, complex secondary metabolites and poor transferability of flow cytometry and cytological methods for sporogenesis [1], [14], [26].

Apple is an economically important crop world-wide [7], [28]–[33]. Polyploidy was identified in apples seventy years ago; several commercial cultivars are triploids [28], [32]. Root-tip chromosome count is reliable for cytotype detection but not informative for meiotic analysis [26], [32]. Microsatellite markers have been successfully applied to genetic and parentage studies [29], [30], [34], but not previously used for cytotypic analysis. Microsatellite markers are co-dominant, highly polymorphic, reproducible, and transferable [29], [30], [34]. The *Malus* 17 linkage groups (LG) correspond to its 17 chromosomes [7], [31]. These advantages of microsatellites enable accurate study of the cytotype genetics and characterization of polyploidization in apples. Molecular phylogenetics accompanying the *Malus* genome confirmed derivation of *Malus* species by auto-polyploidization [7], however, detailed mechanisms of polyploidization in this genus (or in other perennial woody species) are unknown. Several models have been postulated for *Malus* polyploidization based on comparison to the mechanisms described for the herbaceous species [28], [32]. We provide empirical data to test these models integrating cytology and molecular genetic characterization. This study seeks to characterize the details of polyploidization in *Malus* with particularly emphasis on aneuploidization. These data not only add to our knowledge of the evolution and domestication history of the genus, but also are critical in developing breeding strategies and molecular tools for apple breeding programs.

## Results

### Eupolyploids and aneuploids detected in the F_1_ *Malus* populations

We observed a range of non-diploid cytotypes including triploids, tetraploids, and aneuploids, averaging 0.199%, 0.0521%, and 0.778%, respectively, and totaling approximately 1% in the F_1_ seedlings from the seven diploid *Malus* crosses (Table 1). The frequency of aneuploid exceeded eupolyploid seedlings (Table 1), irrespective of crosses; whether intra- or interspecific (*N* = 28, Deviance = 182.3 compared to a χ^2^ distribution with 1 *d.f.*, *P*<2.0×10^−16^). Yet, genetic background did influence frequency of eupolyploid and aneuploid seedlings. Aneuploidy was more frequent among interspecific progeny, while eupolyploid frequency was greater among intraspecific progeny (*N* = 28, Deviance = 11.9 compared to a χ^2^ distribution with 1 *d.f.*, *P* = 5.55×10^−4^).

**Table 1:** Percentage (%) of cytotypes in the seven F_1_ diploid *Malus* populations^a^ and statistical comparisons^b^.

Type of crosses	Crosses	2*n*	2*n*±y^c^	3*n*	4*n*	Sum 3*n*+4*n*	Total
Intraspecific	Gala×Fuji	99.1 (6789)	0.584 (40)	0.219 (15)	0.0584 (4)	0.277 (19)	100 (6848)
Intraspecific	Fuji×Gala	99.0 (5510)	0.665 (37)	0.287 (16)	0.0539 (3)	0.341 (19)	100 (5566)
Intraspecific	Fuji×Pink Lady	98.8 (3249)	0.791 (26)	0.274 (9)	0.0913 (3)	0.365 (12)	100 (3287)
Intraspecific	Pink Lady×Fuji	99.0 (3526)	0.674 (24)	0.281 (10)	0.0561 (2)	0.337 (12)	100 (3562)
Interspecific	M 26×Fu 2	99.0 (2828)	0.805 (23)	0.140 (4)	0.0700 (2)	0.210 (6)	100 (2857)
Interspecific	M 27×Fu 2	99.0 (2833)	0.874 (25)	0.105 (3)	0.0349 (1)	0.140 (4)	100 (2862)
Interspecific	CO 2×RO 6	98.9 (2531)	1.050 (27)	0.0871 (2)	0.0000 (0)	0.0781 (2)	100 (2560)
Intraspecific	Mean	99.0	0.659	0.260	0.0623	0.322	100
	95% CI	98.9, 99.2	0.550, 0.778	0.192, 0.337	0.0321, 0.1024	0.247. 0.404	-
Interspecific	Mean	98.9	0.906	0.109	0.0362	0.145	100
	95% CI	98.7, 99.2	0.711, 1.117	0.050, 0.191	0.0321, 0.0877	0.074, 0.238	-
Chi-square test^b^	-	*P* = 0.5960	*P* = 0.0310	*P* = 0.0081	*P* = 0.3769	*P* = 0.0058	-

a Actual population numbers are presented in parentheses.

b Analysis of Deviance table was constructed to determine the effects due to cytotypes, crosses and type of cross in a generalized linear model (*N* = 28). Coverage intervals (CI) were calculated using an equivalent Bayesian model (refer to Methods).

c y = number of chromosomes (linkage groups) greater or fewer than the diploid number 2*n* = 34.

### Aneuploid cytotypes and chromosomal contribution

Nine cytotypes were detected among the aneuploid seedlings (Table 2 and Table 3). Their frequency was non-uniform; cytotypes 2*n*+2, 2*n*+4, 2*n*+5 or ≥2*n*+12 were absent from the populations (*N* = 28, Deviance = 520.5 compared to a χ^2^ with 16 *d.f.*, *P*<2.0×10^−16^). A multinomial trend represented across aneuploid cytotypes, showing a peak frequency at 2*n*+6, 2*n*+7, and 2*n*+8 (Table 2). Only one cytotype with one chromosome loss, ‘monosomic (2*n*−1)’, but eight cytotypes with chromosomal gain were found in this study (Table 2), indicating genomic increase was primary for aneuploidization in the diploid *Malus*.

**Table 2:** Genetic summary of cytotype distribution and the contribution of individual linkage group (LG) to aneuploid cytotypes.

Linkage group (LG)	2*n*−1^b^	2*n*+1	2*n*+3	2*n*+6	2*n*+7	2*n*+8	2*n*+9	2*n*+10	2*n*+11	Sum of aneuploids affected by LG	Sum of intraspecific aneuploids affected by LG	Sum of interspecific aneuploids affected by LG	Percentage (%) of aneuploids affected by LG
LG01	0	0	0	0	0	0	0	0	0	0	0	0	0.00
LG02	2	0	2	12	21	21	16	11	5	90	60	30	44.6
LG03	0	0	3	11	19	19	13	9	4	78	50	28	38.6
LG04	0	1	3	17	27	27	18	13	6	112	73	39	55.4
LG05	0	2	3	14	26	27	18	12	7	109	66	43	54.0
LG06	0	0	1	13	21	22	14	10	4	85	54	31	42.1
LG07	0	0	0	2	3	4	3	3	1	16	9	7	7.90
LG08	0	0	0	0	0	0	0	0	0	0	0	0	0.00
LG09	0	5	3	18	32	32	19	14	7	130	80	50	64.4
LG10	2	2	4	18	29	30	19	14	6	124	78	46	61.4
LG11	0	0	0	7	9	11	7	5	3	42	26	16	20.8
LG12	0	4	3	13	26	28	16	12	5	107	70	37	53.0
LG13	0	0	1	12	20	20	13	10	6	82	51	31	40.6
LG14	0	2	2	11	16	15	10	8	4	68	35	33	33.7
LG15	0	0	3	12	21	20	14	11	6	87	50	37	43.1
LG16	0	1	3	16	26	27	18	14	7	112	66	46	55.4
LG17	2	2	2	16	26	25	18	14	6	111	71	40	55.0
Sum of individual seedlings^a^	6	19	11	32	46	41	24	16	7	202	-	-	-

a Based on analysis of all aneuploid seedlings in the study, summed over the crosses.

b In column “2*n*−1”, data represents absence of linkage group, for all other aneu-cytotypes, data represents one duplicate linkage group. For example, of the 16 individual “2*n*+10” seedlings, 11 had a duplicate copy of LG02.

**Table 3:** Average percentage (%) of individual chromosome contributing to aneuploids in the seven diploid *Malus* crosses.

	LG01	LG02	LG03	LG04	LG05	LG06	LG07	LG08	LG09	LG10	LG11	LG12	LG13	LG14	LG15	LG16	LG17
2*n*−1	0.00	33.33^a^	0.00	0.00	0.00	0.00	0.00	0.00	0.00	33.33	0.00	0.00	0.00	0.00	0.00	0.00	33.33
2*n*+1	0.00	0.00	0.00	5.26	10.53	0.00	0.00	0.00	26.32	10.53	0.00	21.05	0.00	10.53	0.00	5.26	10.53
2*n*+3	0.00	18.18	27.27	27.27	27.27	9.09	0.00	0.00	27.27	36.36	0.00	27.27	9.09	18.18	27.27	27.27	18.18
2*n*+6	0.00	37.50	34.38	53.13	43.75	40.63	6.25	0.00	56.25	56.25	21.88	40.63	37.50	34.38	37.50	50.00	50.00
2*n*+7	0.00	45.65	41.30	58.70	56.52	45.65	6.52	0.00	69.57	63.04	19.57	56.52	43.48	34.78	45.65	56.52	56.52
2*n*+8	0.00	51.22	46.34	65.85	65.85	53.66	9.76	0.00	78.05	73.17	26.83	68.29	48.78	36.59	48.78	65.85	60.98
2*n*+9	0.00	66.67	54.17	75.00	75.00	58.33	12.50	0.00	79.17	79.17	29.17	66.67	54.17	41.67	58.33	75.00	75.00
2*n*+10	0.00	68.75	56.25	81.25	75.00	62.50	18.75	0.00	87.50	87.50	31.25	75.00	62.50	50.00	68.75	87.50	87.50
2*n*+11	0.00	71.43	57.14	85.71	100.00	57.14	14.29	0.00	100.00	85.71	42.86	71.43	85.71	57.14	85.71	100.00	85.71
Average	0.00	43.64	35.21	50.24	50.44	36.33	7.56	0.00	58.24	58.34	19.06	47.43	37.91	31.47	41.33	51.94	53.08

a The percentage was calculated based on the summary data in the Table 2; Six aneuploid seedlings with cytotype of 2*n*−1 were found in the seven crosses, and among them two seedlings were affected by the LG02 (See Table 2), thus the percentage of LG02 was approximately estimated 33.333% as contributors to 2*n*−1 cytotype.

Chromosomes LG01 and LG08 did not contribute to aneuploidy (Table 2 and Table 3), implying essential functional roles on these chromosomes that require their conservation [7]. Chromosomes LG07 and LG11 were also sparsely represented. Three chromosomes, LG02, LG10, and LG17, were most commonly absent among monosomes (2*n*−1). The *Malus* genome showed that strong co-linearity was between large segments of chromosomes LG05 and LG10, LG09 and LG17, chromosome LG02 strongly with LG07 and ‘chromosome 18’ [7]. Thus one loss of these three sets of chromosomes 10, 17, or 2 will not affect survival of the monosomes because the genic functions on these chromosomes may be redundant. Chromosomes LG09 and LG12 were frequent additional among trisomes (2*n*+1). Chromosomes LG04, LG05, LG09, LG10, LG12, LG16 and LG17, were frequently extra among aneuploids (Table 2 and Table 3). Genetic background had no effect on the contribution of individual chromosomes to aneuploids (*N* = 28, Deviance = 0.01 compared to a χ^2^ with 8 *d.f.*, *P* = 0.990) (Table S12, S13, S14, S15, S16, S17, S18, S19, S20, S21).

### Gametic cytotype patterns contributing to eupolyploids and aneuploids

**Figure 1:**
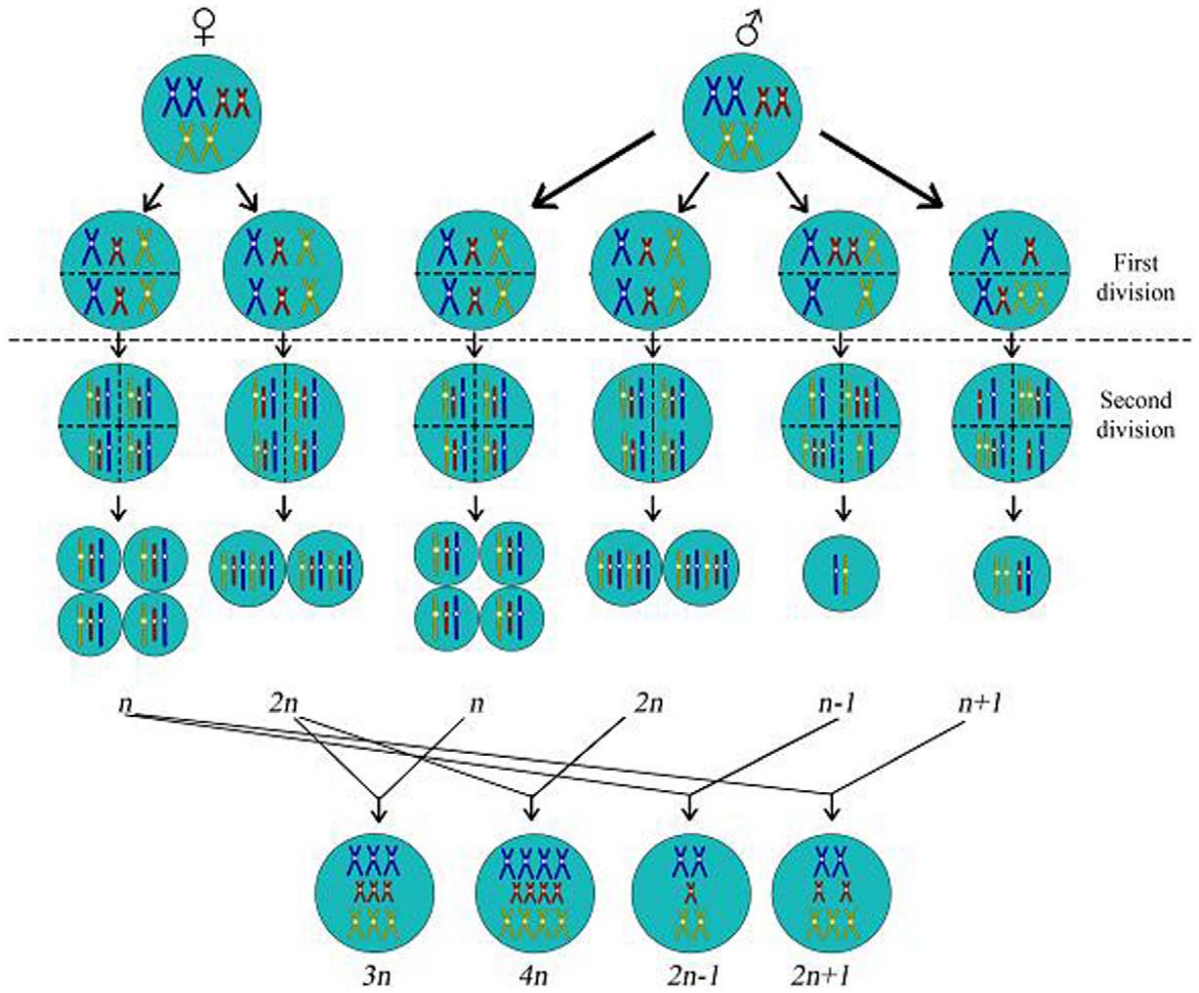
Schematic Summary of the Features of Gametic Combinations for Apple Polyploidization in Diploid *Malus*. Ova have two cytotypes, *n* and 2*n* ova; spermatozoa have a range but classified-into three cytotypes, *n*, 2*n*, and aneuploid spermatozoa for apple polyploidization. ‘*n*−1’ and ‘*n*+1’ refers to two aneuploid spermatozoa for aneuploidization. Diploid *Malus* exhibited a unique gametic combinational pattern, ova preserving euploidy exclusively, while spermatozoa presenting both euploidy and aneuploidy, for polyploidization. Molecular features showed that non-reduced gametes were genetically heterozygous, indicating first-division restitution was the exclusive mode for apple polyploidization. Figure depicts only three basic chromosomes with different colours in the legend to elucidate the basic chromosome number in the apples is odd.

Various gametic patterns among polyploids and aneuploids were found in the diploid herbaceous species [1], [3]–[6], [12]–[13], [15], [18], [27]. For example, triploids were derived from 2*n* (diploid) ova fertilized with *n* (haploid) spermatozoa, and/or *n* ova fertilized with 2*n* spermatozoa. Aneuploids were derived from *n* ova fertilized with aneuploid spermatozoa, or aneuploid ova fertilized with *n* spermatozoa [1], [2], [14], [20]. Polyploidization in *Malus* has been assumed to possess the same features as those in the diploid herbs [28], [32]. However, our data show a unique pattern in the diploid *Malus* that, without exception, all tetraploid seedlings were derived from 2*n* ova fertilized with 2*n* spermatozoa, all triploids from 2*n* ova fertilized with *n* spermatozoa, and all aneuploids from *n* ova fertilized with aneuploid spermatozoa. Thus ova only contributed euploidy while spermatozoa contributed a range of cytotypes, including aneuploidy, to non-diploid seedlings in the diploid *Malus* (Figure 1).

### Meiotic mechanisms for polyploidization in *Malus*

Meiotic mechanisms of non-reduced gametes for polyploidization were previously uncertain in the diploid *Malus* species, for example, whether from first-division restitution (FDR), or from second-division restitution (SDR), or from both [28], [32]. But the distributional features of markers in the non-diploid seedlings showed that all diploid and aneuploid gametes with a cytotype of ≥*n*+1 were genetically heterozygous (Table S1, S2, S3, S4, S5, S6, S7, S8, S9, S10, S11). Thus, FDR was the exclusive mechanism for non-reduction of eudiploid gametes and aneuploid spermatozoa (with a cytotype ≥*n*+1), perhaps by partial non-disjunction [1], [9], [14]. An alternative mechanism may be a two-step process of restitution in the first-division followed by non-disjunction in the second-division, but concurrent diploidization and aneuploidization during a meiotic course has not been established in any species to date [1], [8], [9], [14], [22].

### Auto-triploidization, auto-tetraploidization and auto-aneuploidization in *Malus*

Triploids are considered very important to speciation and evolution as “a bridge for polyploidization” [1], [9]. Triploids were 3.8-fold more frequent than tetraploid seedlings across the diploid *Malus* crosses (Table 1). Triploidy prevails in certain *Malus* species, e.g. ‘*M. hupehensis*’, ‘*M. sikkimensis*’, and ‘*M. toringoides*’, but very few tetraploids have been identified in the *Malus* species [28]. The number of aneuploid seedlings exceeded the number of eupolyploids (Table 1), suggesting that auto-aneuploidization was a major route for genomic duplication in the diploid *Malus*.

## Discussion

### Impacts of gametic polyploidization derived by FDR on evolution

Non-reduced gametes derived by FDR possess a higher heterozygosity and more complex epistatic combinations from their parents than those by SDR [1], [8], [11], [35]–[37], Thus, the genetic differences between FDR and SDR may have an important influence on the fate of neopolyploids either in speciation or evolution [1], [8], [11], [37]. Polyploidy is widely considered to confer ecological adaptability [1], [8], [11], [37], but a molecular genetic basis for this has not been established. This study shows that the molecular genetic composition of diploid gametes is similar to their mother cells (Table S8, S9, S10, S11) and aneuploid gametes had more genotypes than haploid gametes (Table S1, S2, S3, S4, S5, S6, S7), thus polyploids and aneuploids are more heterozygous or genetically diverse than diploid seedlings, providing an expanded base for natural selection.

### Gametic polyploidization patterns in diploid *Malus*

The *Malus* polyploidization patterns presented differently between ova and spermatozoa (Figure 1), and these features are interesting from an evolutionary perspective. Ova, through eupolyploidization, may account for maintenance of genetic stability, preventing biological disturbance and imbalance. And spermatozoa, through a greater range of cytotypes, may convey increased genetic diversity, providing a broad base for natural selection, improving adaptability [1], [2], [6], [10], [11], [22]. Environmental exposure during micro- and megasporogenesis may account for their respective features in polyploidization. Deep in the ovary, the developing megaspore may be less affected by abiotic stressors such as radiation and fluctuant temperature than the microspore. Therefore, at once, the meiotic features of the male and female gametes of *Malus* impart genetic stability and variability, assisting evolution of this genus [2], [9]–[11], [22].

### An alternative model for derivation of *Malus*

In many cases, neopolyploids have displayed a diploid-like meiotic behaviour after their establishment, and underwent diploidization to finally form ‘diploids’ but with genomic duplication [22], [38]. It was recently deduced that *Malus* (*x* = 17) was derived from auto-polyploidization of two sister taxa (*x* = 9, 2*n* = 18), followed by diploidization and then aneu-ploidization to *x* = 17 [7]. Given the relatively reproductive instability of aneuploids, we propose an alternative but more parsimonious three-step path to derivation of *Malus*: auto-aneuploidization of two sister taxa (*x* = 9, 2*n* = 18) to monosomes (*x* = 9, 2*n* = 17), followed by whole genomic duplication in both ova and spermatozoa, involving FDR non-reduction (Figure 1), to tetraploids (*x* = 9, 4*n* = 34), and through diploidization to the extant diploid state (*x* = 17, 2*n* = 34). The frequency of monosomes was lower than other aneuploids in the diploid *Malus* (Table 1). How were monosomes (*x* = 9, 2*n* = 17) more successful than other aneuploids during *Malus* derivation? The reasons may be that monosomes had a higher evolutionary pressure to produce a higher frequency of tetraploids (*x* = 9, 4*n* = 34) than other aneuploids, and these tetraploids presented a stronger adaptability during speciation.

### A path to evolution of odd basic chromosome numbers in the genome

Both auto-polyploidization and allo-polyploidization have been important for plant speciation and domestication, for example apple (*Malus*) [7] and wheat (*Triticale*) [39]. Speciation resulting in an odd basic chromosome number (*x*) was thought to have derived from allo-polyploidization of two species with even and odd basic chromosomes [40]. But recent studies have elevated the importance of auto-polyploidization for natural speciation [21]–[22]. Auto-triploidization of species with odd basic chromosomes such as *Malus* species and auto-aneuploidization of species with odd or even basic chromosomes may be an important contributor to speciation with odd basic chromosome numbers during evolution.

### Impacts of sexual aneuploidization on plant evolution

Our finding in *Malus* conflicts with prevailing views: (i) that eupolyploidy is primary for genomic duplication in diploid crosses, or (ii) that aneuploidy exceeded eupolyploidy in crosses from the parents with different ploidy, e.g. triploid crossed with diploid parents [1], [9], [14], [20], [22]. Impacts of aneuploidization on speciation and evolution have long been ignored. . Aneuploidization can result in speciation with both odd and even basic chromosome numbers, while eupolyploidization can only contribute to even basic chromosome numbers (Table 2 and Table 3). Aneuploids derived from the same taxa can be of much genetic discrepancy. For example, species in Maloideae and certain species in Spiraeoideae have the same basic chromosome numbers (*x* = 17) [7]; they may derive from the same taxa through aneuploidization but with different chromosomal patterns. Regulation of chromosomal loss or gain contributing to aneuploids may be controlled by different genes, thus their effects on survival and adaptation of aneuploids may be very distinct. And this may result in the different evolutionary fates among the aneuploids under the natural selection from diverse environmental cues [1], [14], [22]. Thus aneuploidization can result in multi-directional evolution. Genetic diversity in a polyploid individual may be higher than its diploid ancestor, but within a polyploid population it will consequently decrease. For example, on a ‘abxcd’ locus [28], four diploid genotypes, ‘ac’, ‘ad’, ‘bc’, and ‘bd’ can be found in the diploid *Malus* population, but two genotypes of ‘abc’ and ‘abd’, and one genotype of ‘abcd’ can be detected in the triploids and tetraploids, respectively (Table S8, S9, S10, S11). Thus, eupolyploidization can cause a decrease of genetic diversity on a whole. In contrast, aneuploidization will increase genetic diversity because aneuploids contain more genotypes than diploids (Table S1, S2, S3, S4, S5, S6, S7) [41]. Eupolyploidization can cause gene silencing [42], [43]. Aneuploidization may also result in gene silencing but it may not be so prevalent as in eupolyploidization. In addition, aneuploids may initiate special gene expression, e.g. resistance to a disease or tolerance to coldness or drought because of ‘pseudo-dominant expression’ by chromosomal loss or ‘super-dominant expression’ by chromosomal gain in these aneuploids. Thus, aneuploidization provides a broader base and more diverse conditions for natural selection. Speciation through both eupolyploidization and aneuploidization, particularly aneuploidization with chromosomal loss, may have a higher probability of success than by eupolyploidization alone because of a greater evolutionary pressure in aneuploids during speciation and evolution. However, aneuploids are often less reproductively stable than eupolyploids [1], [14], [22]. Thus an integrated route of ‘aneuploidization-eupolyploidization-diploidization’ for speciation and evolution of *Malus* as defined by our model, is more parsimonious with current data than other routes, e.g. only from eupolyploidization or from aneuploidization. Therefore, advantages and disadvantages in both aneuploidization and eupolyploidization should be properly evaluated, and thus their impacts on speciation and evolution can be appropriately determined.

### A protocol using co-dominant markers for triploid apple breeding

Triploid apples, generally characterized by their large fruits, have been very attractive to both growers and consumers: old cultivars such as ‘Gravenstein’, ‘Baldwin’, ‘Rhode Island Greening’, ‘Blenheim Orange’, ‘Stayman Winesap’, ‘Tompkins County King’, as well as newer cultivars ‘Jonagold’ and ‘Mutsu’ [28]. The fertility of triploids has, however, been found to be lower than that of the diploids and tetraploids, but rarely zero and often with differences between cultivars [1], [9], [28]. This character appeals to both apple breeders and growers because of less labour needed for blossom and fruit thinning in triploids [28]. Despite years of effort, attempts of chromosome doubling using colchicine [36] and endosperm culture [28] have not contributed to release of triploid apple cultivars. More than 90% commercially superior triploid apple cultivars have proved to be produced by sexual polyploidization [28]. Thus, diploid crosses remain the principal method to breed apple triploid cultivars.

There are approximately 8,000 diploid apple cultivars in the world, but fewer than 50 are triploids [28]. Given importance of triploid cultivars in the apple industry, we present a protocol using co-dominant markers aimed to efficiently identify a small number of triploid seedlings in a large population and thus to accelerate the triploid breeding program (Figure S1). In addition, parental genetic backgrounds influenced the frequency of triploids in the diploid F_1_ seedlings (Table 1), thus parents should be carefully chosen for successful triploid breeding.

### Applications of the strategies in this research to the studies for other species

To date, methodology has not been established to determine natural polyploids in a diploid population whether derived by sexual 2*n* gametes or by somatic chromosomal doubling [1], [22]. This question can be addressed by using the strategy outlined in the current work. Polyploids identified by root-tip chromosome count would present extremely close microsatellite features as those in the diploid seedlings, suggesting non-reduced gametes contributing to these polyploids were not genetically heterozygous, further indicating these gametes should have derived from SDR [1], [8], [9]. The sexual 2*n* gametes by FDR are heterozygous and co-dominant markers will present segregation. On the contrary, co-dominant makers will have non-segregation in the asexual 2*n* gametes. Though co-dominant markers in the polyploids derived from somatic polyploidization present similar patterns to those derived from sexual gametes by SDR, crossovers during meiosis resulting in a few heterozygous loci in the sexual 2*n* gametes allows discrimination between these modes. At least one in several hundred co-dominant markers in these sexual gametes will present a different pattern from their parents because of crossover occurrence between homologous chromosomes in these diploid gametes. However, asexual gametes do present the same patterns as their diploid mothers. Thus, our strategy is much more precise than the current widely-used flow cytometry and cytological methods for sporogenesis study.

Spermatozoa in triploids and aneuploids are mostly sterile, but their ova are usually fertile because they can produce euhaploid or a few eudiploid ova [1], [6], [14], [22], [26]. Thus, triploids and aneuploids may be useful in the seed crop breeding program and for commercial seed production as alternative male-sterile materials. Polyploids and aneuploids with extra chromosomes can be used for analysis of genic or chromosomal dosage effects in the study of evolution and genetics; monosomes can be applied to study of function of certain genes or used for rapid genetic mapping in both plants and animals when corresponding dominant genes are affected in these monosomes. Polyploidy and aneuploidy are important for horticultural crops; they usually express valuable traits in the tree architectures or biological styles, for example dwarfing tree-systems or multiple petals, or as male-sterility [44], [45]. Vegetative propagation will facilitate extension of these polyploids and aneuploids in the horticultural industry. Polyploidy was found in bacterium, yeast, fungi, and virus [46]–[49]. Polyploidization is also common in certain animals, or organs of the higher form animals [22], [50]–[51]; polyploidy occurs in humans and causes miscarriages and sterility [2], [9]. This study will encourage people to develop the molecular markers to detect polyploids and aneuploids, thus to precisely diagnose miscarriages and sterility in the early stage in humans or other animals caused by polyploidy or aneuploidy.

### Conclusions

In conclusion, diploid *Malus* presented unique patterns for eupolyploidization and aneuploidization contributing to genomic duplication. For example, aneuploidy exceeded eupolyploidy (Table 1), and ova only contributed euploidy while spermatozoa contributed both euploidy and aneuploidy (Figure 1) for apple polyploidization. These unique characters of polyploidization in the diploid *Malus* confer genetic diversity and multi-directions for evolution and speciation. This is the first demonstration of a strategy using co-dominant markers to successfully analyze meiotic mechanisms and cytotypic derivation of unreduced gametes contributing to polyploids and aneuploids. This study is the first to provide molecular evidence for the contribution of heterozygous non-reduced gametes to genetic heterozygosity and fitness in polyploids and aneuploids, thus aids us understanding of superiority in polyploids and aneuploids. Impacts of aneuploidization on speciation and evolution were previously ignored. Aneuploidy can increase, while eupolyploidy may decrease genetic diversity in their newly established aneuploid or eupolyploid populations. Aneuploidization can result in a greater range of cytotypes for speciation, a higher genetic diversity and a broader base for natural selection. Eupolyploids and aneuploids should be further applied to the studies of genetics, breeding, and evolutionary science. Marker-assisted breeding using co-dominant markers will greatly accelerate breeding of triploid apples. We proposed a path to evolution of numerically odd basic chromosomes and considerably revised the model for derivation of *Malus*. The findings in this research will aid better understanding of evolution, speciation, and domestication of *Malus*. This study provides strategies for a further exploration of genetics of polyploidization and an enhanced polyploid breeding program for other living species, and polyploid genetics for humans.

## Materials and Methods

### Plant Materials

Four intra- and three inter-specific crosses were made, yielding 27,542 viable F_1_ seedlings in 2001. All maternal parents were *Malus*×*domestica* (Borkh.): ‘Gala’, ‘Fuji’, ‘Pink Lady’, ‘M 26’, and ‘M 27’. Inter-specific paternal parents were: ‘Fu 2’ (*M. prunifolia* ((Willd.) Borkh.)), ‘CO 2’ (*M. baccata* ((L.) Borkh.)×*M.*×*domestica* hybrid), and ‘RO 6’ (*M. baccata*×*M. prunifolia* hybrid). The domesticated apples, *Malus*×*domestica*, are primarily derived from *Malus sieversii* ((Ledeb.) M. Roem), but other species including *M. orientalis* and *M.*×*asiatica* are considered to have contributed to its genetic makeup [7]. Because hybridization is very common among *Malus* species, *M. prunifolia* used in this study may also come from the interspecific hybridization [28].

### Root-tip Chromosome Count

Root-tip chromosome count for ploidy identification was assessed according to Pratt and co-authors', with minor modifications [52]. Root tips of no more than 3.0 cm, and collected during 10:30 am and 12:30 pm from early June to early August yielded maximum numbers of metaphase cells. Seedlings more than one year old were analyzed by root-tip chromosome count.

### Microsatellites Genotyping

Microsatellite primers were labelled (6-FAM™, HEX™, or NED™; Applied Biosystems, CA, USA) for amplification of microsatellite loci in single PCR reactions (Promega, WI, USA) from parent or seedling DNA (QIAGEN, CA, USA). PCR reactions were performed in single 20 µL reactions comprising 20 ng DNA, 2.0 mM MgCl_2_, 0.125 mM dNTPs, 0.5 µM primers, 0.3 U GoTaq® Flexi DNA polymerase and GoTaq® Flexi buffer (Promega, WI, USA). PCR protocol: denaturation at 95°C for 1.5 min, 40 cycles amplification [95°C for 30 s, annealing (primer dependent temperature) for 1.5 min, 72°C for 1.5 min], final extension at 72°C for 10 min. A 12.0 µL mixture of three primers pairs' PCR products [3.0 µL 6-FAM™-, 4.0 µL HEX™-, 5.0 µL NED™-labelled products] was made. A 2.0 µL aliquot of this mixture was mixed with 0.4 µL 500 ROX™ size standard plus 8.0 µL Hi-Di™ formamide, and resolved using a 3130 Genetic Analyser (Applied Biosystems, ABI). Genotypes were determined using GeneMarker® v1.6 with manual binning (Softgenetics, PA, USA).

### Framework Genetic Linkage Analysis

Framework linkage groups were constructed using one reciprocal cross with 62 arbitrarily-chosen diploid F_1_ individuals and their parents [41], and compared to public references (http://users.unimi.it/hidras) to confirm transferability and consistency of the markers between crosses.

From 152 pairs of apple microsatellite primers, 136 (90.0%) pairs amplified robust PCR products and their signals were strong enough for genotyping on the ABI instrument. Among these 126 pairs, 113 (89.7%) pairs can amplify 129 consistent, polymorphic and segregating markers in the seven crosses. The 129 microsatellites [29], [30] spanned all 17 linkage groups: per linkage group, 4–12 markers (mean = 7.59) were polymorphic, and 1–4 markers amplified four co-dominant alleles per locus (abxcd) in segregating progeny [41].

The marker order, genetic distance between markers, the overall coverage of genetic distance in the linkage differed among the seven crosses. Reasons resulting in these differences would be F_1_ diploid seedlings and markers in this study may be insufficient for calculation of a ‘precise’ genetic distance between markers, and chromosomal crossovers may differently occur among the seven crosses thus influencing calculation of genetic distance and marker order [53]. Fortunately, we had a few published *Malus* maps as the references [29]–[30], [34] and data from the seven crosses were compared with each others. At last, the markers which consistently clustered in the same linkage group were chosen for genetic analysis.

When a pair of primers produced no less than two markers and were mapped in the different loci, an extension numbers (e. g. ‘_1’, ‘_2’, ‘_3’, or ‘_4’) attached the original name were given for each marker. Sequences of primers can be obtained at the public website (HIDRAS: http://users.unimi.it/hidras).

### Strategies of Cytotypically Genetics Using Micro-satellites

DNA was isolated by a modified CTAB (cetyltrimethylammonium bromide) protocol. DNA of eupolyploids, aneuploids, two arbitrarily-chosen diploid F_1_ seedlings (as the control) identified by cytology, and their parents were genetically analyzed using microsatellite markers. Microsatellites exhibit different distributional features in polyploid and aneuploid seedlings compared to diploids. Consider the markers with the segregation pattern of four different alleles, ‘abxcd’, where ‘ab’ is the maternal genotype, ‘cd’ is the paternal genotype. If the unreduced gametes were heterozygous, we would expect the following allelic distributions in a locus [41]: Diploid seedlings (eudiploids) will present one of four bi-allelic genotypes; ‘ac’, ‘ad’, ‘bc’, or ‘bd’. Eutriploid seedlings will present one of two tri-allelic genotypes; ‘abc’ or ‘abd’, if the unreduced gametes were maternal. Tetraploid seedlings will present one tetra-allelic genotype, ‘abcd’, where each parent contributed an unreduced gamete. Aneuploid seedlings will present allelic distributions similar to eudiploids, except that those markers in the chromosomes “affected” by the aneuploidy will present distributional features with extra chromosomes; ‘acd’ or ‘bcd’, or distributional features with ‘lacking’ chromosomes; ‘a’ or ‘b’, if the extra or missing chromosomes came from the paternal gametes. The genetic features can be confirmed by other markers presenting the segregation patterns of ‘efxeg’, ‘nnxnp’, or ‘lmxll’ alleles [41]. Therefore, cytotype genetics in polyploid and aneuploid seedlings can be characterized by distributional features of microsatellites in these seedlings.

### Statistical Analysis

Generalized linear models were fitted using the R 2.11.1 (http://www.r-project.org). Analysis of Deviance tables were constructed to investigate comparisons and interactions testing the deviance terms against a chi-squared distribution. Coverage intervals were calculated using an equivalent Bayesian model (OpenBUGS software, http://www.openbugs.info) of the generalized linear model fitted, since the standard errors are not symmetric for low percentages [54].

## Supporting Information

Figure S1 **A Protocol Used for the Efficient Identification of Apple Triploid Individuals from a Diploid Parent Crossing.** This protocol includes three stages, (a) ‘identification of the properties of SSR markers using 8 DNA samples (including Step 1, Step 2, and Step 3 in the Figure S1)’, (b) ‘identification of potential triploid individuals (including Step 4, and Step 5 in the Figure S1)’, and (c) ‘confirmation test (Step 6 in the Figure S1)’.
(PDF)

Table S1 **The distributional features of microsatellite markers in the aneuploid seedlings from the cross of ‘Gala×Fuji’.**
(PDF)

Table S2 **The distributional features of microsatellite markers in the aneuploid seedlings from the cross of ‘Fuji×Gala’.**
(PDF)

Table S3 **The distributional features of microsatellite markers in the aneuploid seedlings from the cross of ‘Fuji×Pink Lady’.**
(PDF)

Table S4 **The distributional features of microsatellite markers in the aneuploid seedlings from the cross of ‘Pink Lady×Fuji’.**
(PDF)

Table S5 **The distributional features of microsatellite markers in the aneuploid seedlings from the cross of ‘M 26×Fu 2’.**
(PDF)

Table S6 **The distributional features of microsatellite markers in the aneuploid seedlings from the cross of ‘M 27×Fu 2’.**
(PDF)

Table S7 **The distributional features of microsatellite markers in the aneuploid seedlings from the cross of ‘CO 2×RO 6’.**
(PDF)

Table S8 **The distributional features of microsatellite markers in the triploid seedlings from the crosses of ‘Gala×Fuji’ and ‘M 26×Fu 2’.**
(PDF)

Table S9 **The distributional features of microsatellite markers in the triploid seedlings from the crosses of ‘Fuji×Gala’ and ‘M 27×Fu 2’.**
(PDF)

Table S10 **The distributional features of microsatellite markers in the triploid seedlings from three crosses.**
(PDF)

Table S11 **The distributional features of microsatellite markers in the tetraploid seedlings from six crosses.**
(PDF)

Table S12 **Aneuploid seedlings and their cytotypes in the seven apple diploid crosses.**
(PDF)

Table S13 **‘** ***2n−1*** **’ aneuploid seedlings and their affected chromosome.** ‘LG02’ represents a chromosome; ‘GF01’ is a seedling from a cross of ‘Gala×Fuji’. ‘GF’, ‘FG’, ‘FP’, ‘PF’, ‘M26F’, ‘M27F’, and ‘CR’ represent a cross of ‘Gala×Fuji’, ‘Fuji×Gala’, ‘Fuji×Pink Lady’, ‘Pink Lady×Fuji’, ‘M 26×Fu 2’, ‘M 27×Fu 2’, and ‘CO 2×RO 6’, respectively. 1 = the affected Linkage Group (chromosome) from the spermatozoa that fertilized “normal” ova in the respective individual progeny.
(PDF)

Table S14 **‘** ***2n+1*** **’ aneuploid seedlings and their extra chromosome.**
(PDF)

Table S15 **‘** ***2n+3*** **’ aneuploid seedlings and their extra chromosomes.**
(PDF)

Table S16 **‘** ***2n+6*** **’ aneuploid seedlings and their extra chromosomes.**
(PDF)

Table S17 **‘** ***2n+7*** **’ aneuploid seedlings and their extra chromosomes.**
(PDF)

Table S18 **‘** ***2n+8*** **’ aneuploid seedlings and their extra chromosomes.**
(PDF)

Table S19 **‘** ***2n+9*** **’ aneuploid seedlings and their extra chromosomes.**
(PDF)

Table S20 **‘** ***2n*** **+** ***10*** **’ aneuploid seedlings and their extra chromosomes.**
(PDF)

Table S21 **‘2** ***n*** **+** ***11*** **’ aneuploid seedlings and their extra chromosomes.**
(PDF)
