# Supplementary material for: Molecular Genetic Features of Polyploidization and Aneuploidization Reveal Unique Patterns for Genome Duplication in Diploid Malus
Source: PLoS One. 2012 Jan 10;7(1):e29449. doi: 10.1371/journal.pone.0029449 (PMC3254611; doi:10.1371/journal.pone.0029449)
Supplement: Figure S1 — A Protocol Used for the Efficient Identification of Apple Triploid Individuals from a Diploid Parent Crossing. This protocol includes three stages, (a) ‘identification of the properties of SSR markers using 8 DNA samples (including Step 1, Step 2, and Step 3 in the Figure S1)’, (b) ‘identification of potential triploid individuals (including Step 4, and Step 5 in the Figure S1)’, and (c) ‘confirmation test (Step 6 in the Figure S1)’. (PDF) [file pone.0029449.s001.pdf]

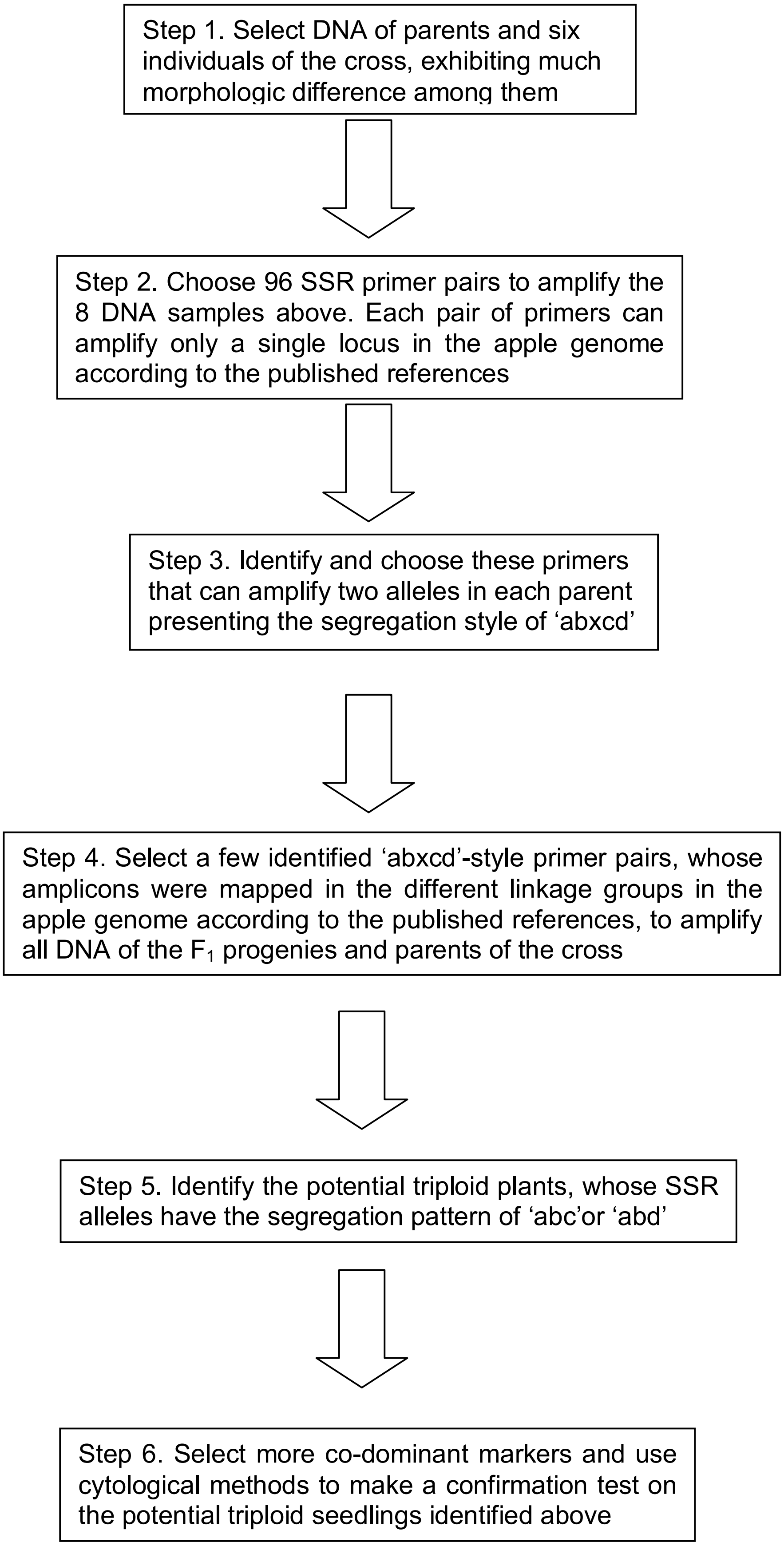
Step 1. Select DNA of parents and six individuals of the cross, exhibiting much morphologic difference among them
Step 2. Choose 96 SSR primer pairs to amplify the 8 DNA samples above. Each pair of primers can amplify only a single locus in the apple genome according to the published references
Step 3. Identify and choose these primers that can amplify two alleles in each parent presenting the segregation style of 'abxcd'
Step 4. Select a few identified 'abxcd'-style primer pairs, whose amplicons were mapped in the different linkage groups in the apple genome according to the published references, to amplify all DNA of the F_1 progenies and parents of the cross
Step 5. Identify the potential triploid plants, whose SSR alleles have the segregation pattern of 'abc'or 'abd'
Step 6. Select more co-dominant markers and use cytological methods to make a confirmation test on the potential triploid seedlings identified above
