# Supplementary material for: Molecular Genetic Features of Polyploidization and Aneuploidization Reveal Unique Patterns for Genome Duplication in Diploid Malus
Source: PLoS One. 2012 Jan 10;7(1):e29449. doi: 10.1371/journal.pone.0029449 (PMC3254611; doi:10.1371/journal.pone.0029449)
Supplement: Table S1 — The distributional features of microsatellite markers in the aneuploid seedlings from the cross of ‘Gala×Fuji’. (PDF) [file pone.0029449.s002.pdf]

| Markers | LG | Aneuploid seedlings from the cross of ‘Gala × Fuji’ | | | | | | | | | | | | | | | | | | | |
|---|---|---|---|---|---|---|---|---|---|---|---|---|---|---|---|---|---|---|---|---|---|
| | | GF01 | GF02 | GF03 | GF04 | GF05 | GF06 | GF07 | GF08 | GF09 | GF10 | GF11 | GF12 | GF13 | GF14 | GF15 | GF16 | GF17 | GF18 | GF19 | GF20 |
| CH05g08 | 1 | ad | bd | ac | bc | ad | ad | bd | bd | bc | bc | bd | bc | bc | bd | ac | ac | ad | bd | bc | ad |
| Hi07d08 | 1 | bd | ad | bc | ac | bc | bc | ac | ac | ad | ad | ac | ad | ad | ac | bd | bd | bc | ac | ad | bc |
| Hi12c02 | 1 | ac | ac | bd | ad | ad | bc | bc | ad | ac | ad | ad | bc | ac | ad | bc | bc | bc | ad | ad | bc |
| KA4B | 1 | hk | hk | h- | kk | h- | h- | h- | kk | hk | kk | kk | h- | hk | kk | h- | h- | h- | kk | kk | h- |
| Hi02c07 | 1 | ll | ll | lm | lm | lm | lm | lm | ll | lm | ll | ll | lm | lm | ll | lm | lm | lm | ll | ll | lm |
| Hi02b10 | 1 | nn | nn | np | np | nn | np | np | nn | np | nn | nn | np | -- | nn | np | np | np | nn | nn | np |
| CH02a04z | 2 | b | bc | bc | bd | bd | bc | bd | ac | acd | bd | bcd | bcd | ad | bd | ad | bd | ad | bcd | bcd | ac |
| CH02c02a_3 | 2 | b | bd | bc | bc | bc | bd | bc | ad | ac- | ad | ac- | ac- | ad | bc | ac | bc | ac | ac- | ac- | bc |
| CH02c06 | 2 | a | bc | bd | bc | bc | bd | bc | ad | bcd | ad | acd | acd | ac | bd | ac | bc | ac | acd | acd | bc |
| CH03d01 | 2 | - | ad | ac | ad | ad | ac | ad | bc | c-- | ad | ac- | ac- | bd | ac | bd | ad | bd | ac- | ac- | bc |
| CH05e03 | 2 | b | ac | ad | ac | ac | ad | ac | bd | bcd | ac | acd | acd | bc | ad | bc | ac | bc | acd | acd | bd |
| CN493139 | 2 | a | ad | bd | ad | bc | bc | ac | ad | acd | bd | bcd | bcd | ac | ad | ac | bc | ac | bcd | acd | bd |
| Hi02a07 | 2 | a | bc | bd | bd | bc | bd | ac | ad | ac- | bc | c-- | c-- | ac | bd | ac | bc | ad | c-- | c-- | ad |
| CH02c02a_2 | 2 | l | ll | lm | lm | lm | lm | ll | ll | lll | ll | lll | llm | ll | lm | ll | lm | ll | llm | lll | ll |
| CH03d10 | 2 | l | lm | lm | lm | ll | lm | ll | ll | lll | lm | llm | llm | ll | lm | ll | lm | ll | llm | lll | lm |
| Hi05c06_3 | 2 | n | nn | np | np | nn | np | nn | np | nnp | np | nnp | nnp | nn | np | nn | nn | nn | nnp | nnp | np |
| CH03e03 | 3 | ac | bd | bd | ad | ad | ac | bd | acd | acd | ac | bcd | bcd | acd | acd | bd | ad | bd | bc | bcd | acd |
| CH03g07 | 3 | bc | bc | ad | ad | ad | bc | bd | acd | acd | ac | bcd | bcd | acd | acd | bd | ad | bc | ac | bcd | acd |
| HGA8bY | 3 | bc | bd | bd | bd | ad | bc | bd | bc- | ac- | bc | ac- | ac- | bc- | bc- | bd | ad | bd | bc | bc- | bc- |
| Hi04c10x_1 | 3 | bc | ac | bd | bd | bd | ac | ad | ac- | c-- | bc | c-- | ac- | c-- | c-- | ad | bd | ac | bd | c-- | ac- |
| Hi07e08x | 3 | ad | ac | ac | bc | bc | ad | ac | c-- | c-- | ad | ac- | ac- | c-- | c-- | ac | bc | ac | bd | ac- | c-- |
| AU223657 | 3 | ll | ll | lm | lm | lm | ll | ll | llm | llm | ll | lll | llm | lll | llm | ll | ll | ll | lm | lll | llm |
| CH02c02b | 4 | bd | ac | bd | ac | ad | bd | bd | acd | acd | ac | bc | bcd | ad | bc | bd | bcd | ac | bcd | bcd | acd |
| CH05d02 | 4 | ad | bc | bc | ac | bd | bd | bd | bc- | ac- | bc | bc | -- | ad | bd | bd | bc- | bd | bc- | bc- | bc- |
| GD162 | 4 | ad | ac | bc | ac | bd | bd | bd | bcd | acd | ac | bc | bcd | ad | bd | bd | bcd | bd | bcd | bcd | bcd |
| Hi04c10x_3 | 4 | bd | ac | ac | bd | ad | ad | bd | ac- | c-- | bc | ac | c-- | bd | ad | ac | ac- | ad | ac- | ac- | ac- |
| CH04e02 | 4 | ee | eg | ee | fg | ef | ee | ee | eeg | eef | fg | eg | efg | ef | ef | ee | efg | ee | efg | efg | efg |
| Hi07b02_4 | 4 | fg | ef | fg | ee | eg | fg | fg | efg | eeg | ee | ef | efg | eg | ef | fg | efg | fg | efg | eeg | efg |
| CH03a04 | 5 | ad | ac | ad | ac | acd | bc | ac | bcd | bd | ad | acd | ad | acd | ac | acd | acd | bcd | ad | bc | ac |
| CH04g09y | 5 | bd | ad | ac | ac | acd | ac | bc | acd | ad | ad | acd | bc | acd | bd | acd | bcd | bcd | bd | ac | bc |
| CH05e06 | 5 | bc | bd | ac | bd | bcd | bd | ad | acd | bd | bc | bcd | ad | bcd | ac | bcd | acd | acd | ac | bd | ad |
| CH03a09 | 5 | ef | fg | ef | eg | efg | eg | fg | eeg | ee | ef | eeg | ef | eeg | fg | efg | efg | efg | ef | eg | fg |
| CH04e03 | 5 | ef | fg | eg | fg | efg | fg | eg | eeg | fg | ef | efg | eg | efg | ee | efg | eeg | eeg | ee | fg | eg |
| CH04h02_2 | 5 | hk | hk | hh | hk | hhk | hk | k- | hkk | k- | hk | hhk | hk | hkk | hk | hhk | hhk | hkk | hk | hk | k- |

| Markers | LG | Aneuploid seedlings from the cross of 'Gala × Fuji' | | | | | | | | | | | | | | | | | | | |
|---|---|---|---|---|---|---|---|---|---|---|---|---|---|---|---|---|---|---|---|---|---|
| | | GF01 | GF02 | GF03 | GF04 | GF05 | GF06 | GF07 | GF08 | GF09 | GF10 | GF11 | GF12 | GF13 | GF14 | GF15 | GF16 | GF17 | GF18 | GF19 | GF20 |
| CH04h02_4 | 5 | kk | h- | h- | h- | hk- | h- | h- | hk- | h- | kk | hk- | kk | hk- | h- | hk- | hk- | hk- | kk | h- | h- |
| Hi04d02 | 5 | hk | hk | hh | hk | hkk | hk | k- | hhk | k- | hk | hkk | hk | hhk | hk | hkk | hhk | hkk | hk | hk | k- |
| Hi11a03 | 5 | hh | hk | hk | hk | hhk | hk | k- | hkk | hk | k- | hhk | hk | hkk | hk | hhk | hkk | hkk | hk | hk | k- |
| Hi21c08 | 5 | hk | kk | hk | hk | hkk | hk | h- | hhk | hk | h- | hhk | hk | hkk | hk | hhk | hkk | hhk | hk | hk | h- |
| CH02a08z | 5 | nn | np | np | np | np1p2 | np | np | np1p2 | np | nn | np1p2 | np | np1p2 | nn | np1p2 | np | np1p2 | nn | np | np |
| CH03d07 | 6 | bc | ac | ac | bc | bd | ad | bc | bc | ac | ac | bcd | bcd | acd | bcd | bcd | bc | acd | bc | bc | bcd |
| CH03d12 | 6 | bd | bd | ad | ad | ac | bc | ad | ad | bd | bd | ac- | ac- | bc- | ac- | ac- | ad | bc- | ad | ad | ac- |
| Hi01d05 | 6 | eg | fg | eg | eg | ee | ef | fg | eg | fg | fg | eeg | eeg | eeg | eeg | eeg | eg | efg | eg | eg | eeg |
| AJ000761 | 6 | k- | k- | k- | k- | k- | hh | k- | k- | k- | k- | hk- | hk- | hk- | hk- | hk- | k- | hk- | k- | k- | hk- |
| CH03c01 | 6 | h- | h- | h- | h- | kk | kk | h- | h- | h- | h- | hk- | hk- | hk- | hk- | hk- | h- | hk- | h- | h- | hk- |
| Hi04c10x_2 | 7 | ad | ad | bc | bc | bd | ac | ac | ac | bc | bc | bc | bc | bc- | bd | ac | ac | ac | ad | ad | bd |
| CH05b06z_2 | 7 | eg | eg | ef | ef | fg | ee | ef | ee | ef | ef | ef | ef | efg | fg | ee | ee | ee | eg | eg | fg |
| CH04e05 | 7 | lm | ll | ll | lm | lm | ll | ll | ll | lm | lm | lm | lm | lll | lm | ll | ll | ll | ll | ll | lm |
| Hi05b09 | 7 | lm | lm | ll | ll | ll | lm | lm | lm | ll | ll | ll | ll | lll | ll | lm | lm | lm | lm | lm | ll |
| CH01c06 | 8 | ad | bc | ac | bc | ad | ac | bd | bc | bc | bc | ac | ac | bd | bd | ac | bd | bc | ac | bd | bc |
| Hi04b12 | 8 | ad | bd | bc | ad | bc | bd | ac | ad | ad | ad | bd | bd | ac | ac | bd | ac | ad | bd | ac | ad |
| CH02g09 | 8 | lm | ll | ll | lm | ll | ll | lm | lm | ll | ll | ll | ll | lm | lm | ll | lm | lm | ll | ll | lm |
| Hi04e05 | 8 | lm | lm | ll | ll | lm | lm | ll | ll | lm | lm | lm | lm | ll | ll | lm | ll | ll | lm | lm | ll |
| Hi23g12 | 8 | np | np | nn | np | nn | np | nn | np | np | np | np | np | nn | nn | np | nn | np | np | nn | np |
| CH01h02_2 | 9 | ad | bc | bc | ac- | ad | ad | ac | ad | ac | bc- | bc | ad | ad | ac- | bc- | bd | bc- | ac- | ad | ac- |
| CH05c07 | 9 | bc | bc | bc | bcd | bd | ac | ac | bd | ad | bcd | ad | bd | ac | bcd | bcd | ac | acd | bcd | bd | bcd |
| GD142 | 9 | bd | bd | ac | acd | ac | ac | ad | ac | ad | acd | ad | bd | ad | acd | acd | bc | bcd | acd | ac | acd |
| Hi05e07 | 9 | ad | ad | bc | c-- | bc | bc | bd | bc | bd | ac- | bc | ad | bc | c-- | ac- | ac | ac- | c-- | bc | c-- |
| NH029a | 9 | ac | ac | bd | bcd | bd | bd | bc | bd | bc | acd | bd | ac | bd | bcd | acd | ad | acd | bcd | bd | bcd |
| CH01h02_1 | 9 | k- | k- | hh | hhk | hh | hh | k- | hh | k- | hkk | hh | k- | k- | hkk | hhk | hk | hkk | hhk | hh | hkk |
| Hi01d01 | 9 | ll | ll | ll | llm | lm | lm | lm | lm | lm | lll | lm | ll | lm | llm | lll | ll | lll | llm | lm | llm |
| CH05d08y_2 | 9 | nn | nn | np | np1p2 | np | np | np | np | np | np1p2 | nn | nn | np | np1p2 | np1p2 | nn | np1p2 | np1p2 | np | np1p2 |
| CH01f07a | 10 | ac | bc | ad | bd | bd | ac | ac | ad | bd | acd | bd | bd | bd | bcd | ad | acd | acd | bcd | acd | acd |
| CH01f12 | 10 | bd | bc | ac | bd | bd | ad | ad | ad | bd | bcd | ac | ad | bd | bcd | bd | bcd | acd | bcd | bcd | acd |
| CH02b03b | 10 | ad | bc | bd | ac | ac | bd | bd | bc | ac | bc- | ac | ac | ac | ac- | bc | bc- | bc- | ac- | bc- | bc- |
| CH02b07 | 10 | ac | bc | bd | bc | ac | ac | bc | ac | bd | bcd | ac | ad | bc | bcd | bc | bcd | acd | bcd | bcd | acd |
| CH02c11 | 10 | ad | bc | bd | ac | ac | bc | bc | bc | ac | bc- | ac | ac | ac | ac- | bc | bc- | bc- | ac- | bc- | bc- |
| MS06g03 | 10 | ad | ac | bd | ad | bd | bd | ad | bd | ac | ac- | bc | bd | ad | ac- | ad | ac- | c-- | ac- | ac- | c-- |
| CH02a10 | 10 | ll | lm | lm | lm | ll | ll | lm | ll | lm | lll | ll | ll | lm | lll | lm | lll | lll | lll | lll | lll |

| Markers | LG | Aneuploid seedlings from the cross of ‘Gala × Fuji’ | | | | | | | | | | | | | | | | | | | |
|---|---|---|---|---|---|---|---|---|---|---|---|---|---|---|---|---|---|---|---|---|---|
| | | GF01 | GF02 | GF03 | GF04 | GF05 | GF06 | GF07 | GF08 | GF09 | GF10 | GF11 | GF12 | GF13 | GF14 | GF15 | GF16 | GF17 | GF18 | GF19 | GF20 |
| CH03d11 | 10 | lm | lm | ll | lm | ll | ll | lm | ll | lm | llm | ll | ll | lm | llm | lm | llm | llm | llm | llm | llm |
| CH04c06y_1 | 10 | ll | ll | lm | ll | lm | lm | ll | lm | ll | lll | lm | lm | ll | lll | ll | lll | llm | lll | lll | llm |
| Hi02d04 | 10 | ll | ll | ll | ll | lm | ll | lm | ll | ll | llm | lm | lm | lm | lll | lm | llm | llm | lll | lll | lll |
| Hi04f08 | 10 | nn | np | nn | nn | nn | nn | nn | nn | nn | nnp | np | np | nn | nnp | nn | nnp | nnp | nnp | nnp | nnp |
| MS02a01 | 10 | np | nn | np | nn | nn | np | np | nn | nn | nnp | nn | nn | nn | nnp | nn | nnp | nnp | nnp | nnp | nnp |
| CH02d08 | 11 | ac | bc | bd | ad | ac | ad | ad | bd | bd | ac | ac | acd | bd | ac | bc | ad | bcd | bd | bc | ad |
| CH04g07 | 11 | ac | ac | ad | bd | bc | bd | bc | bd | ad | bc | ac | bcd | bd | bc | ac | bc | bcd | ad | ad | bd |
| CH04h02_1 | 11 | bd | ad | ac | bc | bd | bc | ac | ac | ac | ad | bd | bc- | ac | bd | ad | bc | ac- | ac | ad | bc |
| Hi06b06 | 11 | ad | ad | ac | bc | bd | bc | bd | bc | ac | bd | ad | acd | bc | bd | ad | bd | bcd | ac | bc | bc |
| CH04h02_3 | 11 | nn | nn | np | np | nn | np | np | nn | np | nn | nn | nnp | np | nn | nn | np | nnp | np | nn | np |
| CH01b12y | 12 | bd | ad | acd | bd | ad | bcd | bd | bc | ad | acd | ad | bc | ac | bc | acd | bcd | acd | acd | acd | acd |
| CH01g12 | 12 | bc | ac | bcd | ac | bc | acd | ac | ad | bc | bcd | ad | bc | bd | ad | bcd | acd | bcd | bcd | bcd | bcd |
| NZ28f04 | 12 | bd | ad | bcd | ad | bd | acd | ad | ac | bd | bcd | ac | bd | ac | ac | bcd | acd | bcd | bcd | bcd | bcd |
| CH01f02 | 12 | fg | eg | eef | fg | ee | eef | fg | ef | fg | efg | eg | ee | ef | ef | efg | eef | efg | efg | eef | eeg |
| CH05d04 | 12 | ee | eg | efg | ee | eg | eef | ee | ef | eg | eef | fg | eg | ef | ef | eeg | efg | efg | eeg | eef | efg |
| CH05d11 | 12 | ef | ee | efg | ee | fg | eeg | ee | eg | ee | efg | fg | ef | eg | eg | efg | eeg | eeg | efg | eeg | efg |
| CH03h03z_2 | 12 | nn | nn | nnp | nn | np | nnp | nn | np | nn | nnp | np | nn | np | np | nnp | nnp | nnp | nnp | nnp | nnp |
| CH03a08 | 13 | ad | ad | bd | ad | ad | bc | bc | ad | ad | bd | bc | bc | bcd | ad | bcd | ad | acd | acd | bc | bcd |
| CH03h03z_1 | 13 | bd | bd | ac | bd | bd | ac | ac | ad | bd | ad | ac | ac | ac- | bd | ac- | bd | c-- | c-- | ac | ac- |
| CH05c06_1 | 13 | ad | bc | ac | bc | bc | ad | bc | ad | bc | ad | bc | ac | c-- | bc | bc- | ac | bc- | c-- | ac | c-- |
| CH05f04 | 13 | bc | ac | bd | bd | ac | ac | ac | bd | bd | ac | bc | ac | c-- | ac | ac | bc | ac- | c-- | bc | c-- |
| CH05h05 | 13 | bd | ad | bc | ad | ad | bc | ad | bc | bc | ad | bd | ad | bcd | ad | ac- | bd | acd | bcd | bd | bcd |
| Hi03e04 | 13 | bc | bc | ac | ac | bc | ad | ad | bc | bc | ac | ad | ad | ac- | ad | ac- | ad | bc- | bc- | ad | ac- |
| Hi20b03 | 13 | ac | bd | ad | bd | bd | ac | ad | ad | ad | ac | ac | ac | acd | bd | acd | ad | bcd | bcd | ac | acd |
| NH009b | 13 | bc | ac | bd | ac | ac | bd | bc | bc | bd | bc | bd | bd | bcd | ac | bcd | bc | acd | acd | bd | bcd |
| AU223486 | 13 | k- | k- | hk | k- | k- | hk | hk | k- | k- | hh | hk | hk | hhk | k- | hkk | k- | hkk | hhk | hk | hkk |
| GD147 | 13 | k- | hh | k- | hh | hh | k- | k- | k- | k- | k- | k- | k- | hkk | hh | hhk | k- | hhk | hhk | k- | hkk |
| Hi05c06_2 | 13 | h- | kk | hk | kk | kk | h- | h- | hk | h- | h- | h- | h- | hhk | kk | hkk | h- | hhk | hkk | h- | hhk |
| Hi07b02_3 | 13 | ll | lm | ll | lm | lm | ll | ll | ll | ll | ll | ll | ll | lll | lm | lll | ll | llm | llm | ll | lll |
| NZ03c01x_2 | 13 | lm | lm | lm | lm | lm | ll | ll | ll | lm | lm | ll | ll | lll | lm | lll | lm | llm | llm | lm | lll |
| CH01g05 | 14 | ac | bc | ad | ac | bc | ad | bcd | ad | ad | bd | bc | bd | bc | ad | acd | ad | bc | bc | bcd | ad |
| CH03a02 | 14 | ac | ad | ac | ac | bc | ac | bcd | ac | bc | ad | bc | ad | bd | ac | acd | bc | bd | bc | bcd | ac |
| CH03d08 | 14 | bc | bd | bd | ad | ad | bc | acd | bc | ac | bc | ac | ad | ad | bc | bcd | bc | ad | ad | acd | bc |
| CH05g07z_1 | 14 | hk | hk | hk | hk | hk | hk | hhk | k- | k- | hh | hk | hh | k- | k- | hkk | hk | hk | hk | hhk | k- |

| Markers | LG | Aneuploid seedlings from the cross of 'Gala × Fuji' | | | | | | | | | | | | | | | | | | | |
|---|---|---|---|---|---|---|---|---|---|---|---|---|---|---|---|---|---|---|---|---|---|
| | | GF01 | GF02 | GF03 | GF04 | GF05 | GF06 | GF07 | GF08 | GF09 | GF10 | GF11 | GF12 | GF13 | GF14 | GF15 | GF16 | GF17 | GF18 | GF19 | GF20 |
| CH05g07z_2 | 14 | hk | hk | hk | hk | hk | hk | hhk | h- | kk | h- | kk | hk | h- | h- | hkk | hk | hk | hk | hhk | h- |
| CH02c02a_1 | 15 | ad | ad | ad | bc | bd | ad | bd | ad | ac | bc | ac- | bc | ac | ac | ac | ac- | bd | bc | ad | ad |
| CH02d11 | 15 | bc | ac | bc | bd | ad | bd | ad | bd | bc | bc | acd | ac | bc | bc | bc | bcd | bd | ac | ad | ac |
| CH03b10 | 15 | ac | bc | ac | ad | bd | ad | bd | ad | ac | ac | bcd | bc | ac | ac | ac | acd | ad | bc | bd | bc |
| Hi04c05 | 15 | ad | ac | ad | bd | bc | ac | bc | ac | ad | ad | bc- | bd | ad | ad | ad | ac- | ac | bd | bc | bd |
| Hi06f09 | 15 | ad | bc | bd | ad | ac | bc | ac | bc | bd | ad | bcd | ad | bd | bd | ad | bcd | ac | ad | bc | bd |
| NZ02b01 | 15 | ad | ac | ad | bd | bc | ac | bc | ac | ad | ad | bc- | bd | ad | ad | ad | ac- | ac | bd | bc | bd |
| CH02c09 | 15 | lm | lm | lm | lm | ll | lm | ll | lm | lm | ll | lll | ll | lm | lm | lm | llm | lm | ll | ll | ll |
| Hi02g06 | 15 | np | nn | np | nn | nn | nn | nn | nn | np | np | np1p2 | np | np | nn | np | np1p2 | nn | np | nn | np |
| CH02d10a | 16 | bc | bc | ac | bd | bd | ad | bc | bd | bc | bc | ac | bc- | bc- | ac- | bd | ac- | ad | bd | ad | ac |
| CH04f10 | 16 | bd | bd | bd | bc | ad | ac | ac | ac | ad | ad | bd | bc- | bc- | bc- | ad | bc- | bc | ad | bc | bd |
| CH05a04 | 16 | bc | ac | ac | ad | bd | bd | ad | bd | bc | bc | ac | acd | acd | acd | bc | acd | ad | bd | ad | ac |
| CH05c06_2 | 16 | bd | ad | bd | bd | bd | ad | ad | bd | bd | ac | ad | bcd | acd | acd | ad | acd | bd | bd | ad | ad |
| Hi01d06y | 16 | ad | ad | ad | ac | bd | bc | bc | bc | bd | bd | ad | acd | acd | acd | bd | acd | ac | bd | ac | ad |
| Hi04e04 | 16 | ad | bc | bd | ac | ac | bc | bc | ac | ad | ad | bd | bcd | bcd | bcd | ac | bcd | bc | ac | bc | bd |
| CH05b06z_1 | 16 | ef | ef | ef | ef | ef | ee | ee | ef | eg | ee | ee | eef | efg | eef | ee | efg | ef | ef | ee | ee |
| Hi01c11x | 16 | eg | eg | eg | eg | eg | fg | fg | eg | fg | ef | fg | eeg | eeg | efg | fg | efg | eg | eg | fg | fg |
| CH01h01 | 17 | bd | b | bc | bd | bd | ac | ac | bd | ad | bd | acd | bd | ac | bcd | bd | acd | ac | bcd | bcd | bd |
| CH04c06y_2 | 17 | bd | a | ac | ac | ad | ad | bd | bd | ad | ad | c-- | bd | ac | bc- | bd | c-- | bc | bc- | c-- | ac |
| CH05d08y_1 | 17 | bd | a | bd | ac | bc | bc | ad | ac | bc | bc | c-- | ac | bd | ac- | ac | c-- | ad | ac- | c-- | bd |
| CH05g03 | 17 | ad | a | ac | ad | ad | bc | bc | ad | ad | ad | bcd | ad | bc | acd | ad | bcd | bc | acd | acd | ad |
| CH04c06y_3 | 17 | lm | m | lm | lm | lm | lm | ll | ll | lm | lm | llm | ll | ll | lll | lm | llm | ll | llm | llm | lm |
| GD96 | 17 | lm | l | lm | lm | ll | lm | ll | lm | ll | lm | lll | ll | ll | lll | ll | lll | ll | lll | lll | lm |
| Hi03c05 | 17 | ll | l | ll | ll | lm | ll | lm | ll | ll | lm | llm | lm | lm | lll | lm | llm | lm | lll | lll | ll |
| Hi05c06_1 | 17 | ll | m | ll | lm | lm | ll | lm | lm | ll | ll | lll | lm | ll | llm | lm | lll | lm | llm | lll | ll |
| Hi07b02_1 | 17 | lm | l | lm | ll | lm | ll | lm | ll | lm | ll | llm | ll | lm | lll | lm | lll | lm | lll | llm | ll |
| Hi07b02_2 | 17 | np | n | nn | np | np | nn | np | nn | nn | np | nnp | nn | nn | nnp | np | nnp | np | nnp | nnp | nn |

| Markers | LG | Aneuploid seedlings from the cross of 'Gala × Fuji' | | | | | | | | | | | | | | | | | | | |
|---|---|---|---|---|---|---|---|---|---|---|---|---|---|---|---|---|---|---|---|---|---|
| | | GF21 | GF22 | GF23 | GF24 | GF25 | GF26 | GF27 | GF28 | GF29 | GF30 | GF31 | GF32 | GF33 | GF34 | GF35 | GF36 | GF37 | GF38 | GF39 | GF40 |
| CH05g08 | 1 | ac | bd | bc | bd | ac | bd | ad | ad | bd | bd | bd | bd | ad | bd | bd | bd | ad | bd | bc | ac |
| Hi07d08 | 1 | bd | ac | bc | ac | ad | ac | bc | bc | ac | ac | ac | ac | bc | ac | ac | ac | ad | bd | bc | ac |
| Hi12c02 | 1 | bc | bc | ac | ad | ac | ad | bd | ad | ad | ad | ad | bd | ac | ad | ad | ad | ad | bc | ad | ad |
| KA4B | 1 | h- | h- | h- | kk | hk | kk | h- | kk | kk | kk | h- | h- | h- | kk | kk | kk | kk | h- | kk | kk |
| Hi02c07 | 1 | lm | lm | lm | lm | lm | ll | lm | ll | ll | ll | lm | lm | lm | ll | ll | ll | ll | lm | ll | lm |
| Hi02b10 | 1 | np | np | np | np | np | nn | np | nn | nn | nn | np | np | np | nn | nn | nn | nn | np | nn | np |
| CH02a04z | 2 | bcd | ad | ac | ad | acd | bcd | acd | bcd | bd | ac | ad | acd | acd | acd | acd | acd | bcd | bcd | acd | bcd |
| CH02c02a_3 | 2 | bc- | ac | ad | ac | ac- | ac- | bc- | bc- | bc | ad | ac | ac- | bc- | ac- | ac- | ac- | ac- | bc- | bc- | ac- |
| CH02c06 | 2 | bcd | ac | ad | ac | acd | bcd | acd | bcd | bc | ad | ac | bcd | bcd | bcd | acd | bcd | acd | bcd | acd | bcd |
| CH03d01 | 2 | ac- | bd | bc | bd | c-- | ac- | ac- | ac- | ad | bc | bd | c-- | c-- | c-- | c-- | ac- | ac- | ac- | c-- | ac- |
| CH05e03 | 2 | acd | bc | bd | bc | bcd | acd | bcd | acd | ac | bd | bc | bcd | bcd | bcd | bcd | acd | acd | acd | bcd | acd |
| CN493139 | 2 | bcd | bc | ad | bc | acd | bcd | acd | bcd | ac | ad | bc | bcd | bcd | acd | bcd | acd | acd | bcd | bcd | bcd |
| Hi02a07 | 2 | ac- | ac | ad | ac | ac- | c-- | ac- | c-- | ac | ad | ac | ac- | ac- | ac- | c-- | c-- | c-- | c-- | ac- | c-- |
| CH02c02a_2 | 2 | llm | ll | ll | ll | lll | llm | lll | lll | ll | ll | ll | llm | llm | lll | llm | lll | lll | llm | llm | lll |
| CH03d10 | 2 | llm | ll | ll | ll | lll | llm | lll | llm | ll | ll | ll | llm | llm | lll | llm | lll | lll | llm | llm | llm |
| Hi05c06_3 | 2 | nnp | nn | np | nn | nnp | nnp | nnp | nnp | nn | np | nn | nnp | nnp | nnp | nnp | nnp | nnp | nnp | nnp | nnp |
| CH03e03 | 3 | ad | bcd | acd | acd | bc | bc | acd | ac | ad | ad | bcd | acd | ac | ac | acd | bcd | ad | bcd | bcd | bd |
| CH03g07 | 3 | ad | bcd | bcd | acd | bd | bc | acd | bc | ad | ad | bcd | bcd | ac | bc | acd | acd | ad | bcd | bcd | bd |
| HGA8bY | 3 | bd | ac- | bc- | bc- | ac | ac | bc- | bc | ad | ad | ac- | bc- | bc | bc | bc- | bc- | ad | bc- | ac- | ac |
| Hi04c10x_1 | 3 | bd | c-- | ac- | c-- | ad | ac | c-- | ac | bd | bd | ac- | ac- | ac | ac | c-- | c-- | bd | ac- | ac- | ad |
| Hi07e08x | 3 | bc | ac- | c-- | c-- | bd | bd | c-- | ad | bc | bc | ac- | c-- | ad | ad | c-- | ac- | bc | ac- | ac- | ac |
| AU223657 | 3 | ll | lll | llm | llm | lm | lm | lll | ll | lm | lm | llm | lll | lm | ll | lll | lll | ll | llm | llm | ll |
| CH02c02b | 4 | ad | ac | acd | acd | ad | bcd | acd | bcd | acd | ad | acd | acd | bc | ad | bcd | bcd | bcd | bcd | ad | bcd |
| CH05d02 | 4 | ad | bd | ac- | ac- | bc | ac- | ac- | bc- | ac- | -- | bc- | bc- | bc | bd | ac- | bc- | bc- | bc- | ad | ac- |
| GD162 | 4 | ad | bd | acd | acd | bc | acd | acd | bcd | acd | bd | bcd | bcd | bc | bd | acd | bcd | bcd | bcd | ad | acd |
| Hi04c10x_3 | 4 | bd | ad | c-- | c-- | ac | c-- | c-- | ac- | c-- | ad | ac- | ac- | ac | ad | c-- | ac- | ac- | ac- | bd | c-- |
| CH04e02 | 4 | ef | fg | eef | eef | eg | eef | eef | efg | eef | ee | efg | efg | eg | ef | eef | efg | efg | eef | ef | eef |
| Hi07b02_4 | 4 | eg | ee | eeg | eeg | ef | eeg | eeg | efg | eeg | fg | efg | efg | ef | eg | eeg | efg | efg | eeg | eg | eeg |
| CH03a04 | 5 | ad | acd | acd | acd | acd | bcd | bc | acd | ad | acd | acd | acd | bc | ad | bcd | acd | ad | bcd | acd | bcd |
| CH04g09y | 5 | bd | bcd | bcd | bcd | bcd | acd | ac | bcd | bd | bcd | bcd | acd | bd | bd | bcd | acd | ad | acd | bcd | acd |
| CH05e06 | 5 | ac | acd | acd | acd | acd | acd | bd | acd | ac | acd | bd | bcd | bd | ac | acd | acd | ac | bcd | acd | bcd |
| CH03a09 | 5 | ef | efg | efg | efg | efg | eeg | eg | efg | ef | efg | efg | eeg | eg | ef | eeg | eeg | ef | eeg | efg | eeg |
| CH04e03 | 5 | ee | eeg | eeg | eeg | eeg | eeg | fg | eeg | eg | eeg | efg | efg | fg | ee | eeg | eeg | ee | efg | eeg | efg |
| CH04h02_2 | 5 | k- | hkk | hhk | hhk | hkk | hkk | hh | hkk | k- | hhk | hkk | hhk | hk | hk | hkk | hhk | hk | hkk | hk- | hk- |

| Markers | LG | Aneuploid seedlings from the cross of 'Gala × Fuji' | | | | | | | | | | | | | | | | | | | |
|---|---|---|---|---|---|---|---|---|---|---|---|---|---|---|---|---|---|---|---|---|---|
| | | GF21 | GF22 | GF23 | GF24 | GF25 | GF26 | GF27 | GF28 | GF29 | GF30 | GF31 | GF32 | GF33 | GF34 | GF35 | GF36 | GF37 | GF38 | GF39 | GF40 |
| CH04h02_4 | 5 | kk | hk- | hk- | hk- | hk- | hk- | h- | hk- | kk | hk- | hk- | hk- | h- | kk | hk- | hk- | kk | hk- | hk- | hk- |
| Hi04d02 | 5 | k- | hkk | hhk | hkk | hkk | hhk | hh | hkk | k- | hhk | hkk | hhk | hk | hk | hhk | hhk | hk | hhk | hk- | hk- |
| Hi11a03 | 5 | k- | hhk | hkk | hhk | hhk | hkk | hh | hhk | k- | hkk | hhk | hkk | hk | hk | hhk | hkk | hk | hhk | hk- | hk- |
| Hi21c08 | 5 | h- | hhk | hkk | hhk | hkk | hhk | kk | hk- | h- | hkk | hhk | hhk | hk | hk | hkk | hhk | hk | hkk | hk- | hk- |
| CH02a08z | 5 | nn | np1p2 | np1p2 | np1p2 | np1p2 | np1p2 | np | np1p2 | nn | np1p2 | np1p2 | np1p2 | np | nn | np1p2 | np1p2 | nn | np1p2 | np1p2 | np1p2 |
| CH03d07 | 6 | bc | acd | acd | acd | bd | bcd | acd | acd | ac | acd | bc | bd | bc | bc | bcd | bcd | bd | bcd | ac | acd |
| CH03d12 | 6 | ad | bc- | bc- | bc- | ac | ac- | bc- | bc- | bd | bc- | ad | ac | ad | ad | ac- | ac- | ac | ac- | bd | bc- |
| Hi01d05 | 6 | eg | efg | efg | efg | ee | eeg | efg | efg | fg | efg | eg | ee | eg | eg | eeg | eeg | ee | eeg | fg | efg |
| AJ000761 | 6 | k- | hk- | hk- | hk- | k- | hk- | hk- | hk- | k- | hk- | k- | k- | k- | k- | hk- | hk- | k- | hk- | k- | hk- |
| CH03c01 | 6 | h- | hk- | hk- | hk- | h- | hk- | hk- | hk- | h- | hk- | h- | h- | h- | h- | hk- | hk- | h- | hk- | h- | hk- |
| Hi04c10x_2 | 7 | bcd | bc | bc | ad | bc | bd | bd | ad | bd | bc | ad | bd | acd | bcd | bcd | ad | ad | ad | ac | ac |
| CH05b06z_2 | 7 | eeg | ef | ef | eg | fg | fg | fg | eg | fg | ef | eg | fg | efg | eeg | efg | eg | eg | eg | ee | ee |
| CH04e05 | 7 | lll | lm | lm | ll | lm | lm | lm | ll | lm | lm | ll | lm | lll | lll | lll | ll | ll | ll | ll | ll |
| Hi05b09 | 7 | lll | ll | ll | lm | ll | ll | ll | lm | ll | ll | lm | ll | llm | lll | lll | lm | lm | lm | lm | lm |
| CH01c06 | 8 | bc | ac | bc | ac | ac | ac | bc | bc | ad | bd | ac | ad | ad | bc | bd | bd | ad | bc | bc | bc |
| Hi04b12 | 8 | ad | bd | ad | bd | bd | bd | ad | ad | bc | ac | bd | bc | bc | ad | ac | ac | ad | ad | bc | ad |
| CH02g09 | 8 | lm | ll | lm | lm | ll | ll | lm | lm | ll | lm | ll | ll | ll | lm | lm | lm | ll | lm | lm | lm |
| Hi04e05 | 8 | ll | lm | ll | ll | lm | lm | ll | ll | lm | ll | lm | lm | lm | ll | ll | ll | ll | ll | lm | ll |
| Hi23g12 | 8 | nn | np | np | np | np | np | nn | np | nn | nn | np | nn | nn | np | nn | nn | np | np | nn | np |
| CH01h02_2 | 9 | ac- | bd | ac | bc- | bc | bd | bc | bc- | ac- | ac- | bc- | ac | ac- | ac- | ac- | bc- | bc- | bc | ac- | bc- |
| CH05c07 | 9 | bcd | bc | ad | bcd | ac | bd | bd | acd | acd | acd | acd | ac | bcd | bcd | bcd | bcd | acd | ac | acd | bcd |
| GD142 | 9 | acd | bc | ad | acd | ac | bc | bd | acd | acd | acd | bcd | ad | bcd | acd | acd | bcd | acd | bd | acd | acd |
| Hi05e07 | 9 | c-- | ac | bd | ac- | ad | ac | ad | ac- | c-- | c | ac- | bd | c-- | c-- | c-- | ac- | ac- | ad | c-- | ac- |
| NH029a | 9 | bcd | ad | bc | acd | ac | ad | ac | acd | bcd | bcd | acd | bc | bcd | bcd | bcd | acd | acd | ac | bcd | acd |
| CH01h02_1 | 9 | hhk | hk | k- | hkk | hh | hk | k- | hkk | hhk | hkk | hkk | k- | hhk | hhk | hhk | hhk | hkk | k- | hk- | hk- |
| Hi01d01 | 9 | llm | ll | ll | lll | ll | ll | ll | lll | llm | llm | lll | lm | llm | llm | llm | lll | lll | ll | llm | lll |
| CH05d08y_2 | 9 | np1p2 | nn | np | np1p2 | nn | nn | nn | np1p2 | np1p2 | np1p2 | np1p2 | np | np1p2 | np1p2 | np1p2 | np1p2 | np1p2 | nn | np1p2 | np1p2 |
| CH01f07a | 10 | bcd | bcd | ad | acd | bcd | acd | bcd | ad | acd | acd | bd | ac | acd | acd | bcd | acd | acd | bcd | acd | acd |
| CH01f12 | 10 | acd | bcd | ac | acd | acd | acd | bcd | ac | acd | acd | bd | ad | acd | bcd | acd | bcd | acd | bcd | acd | bcd |
| CH02b03b | 10 | ac- | ac- | bc | bc- | ac- | bc- | ac- | bc | bc- | bc- | ac | bd | bc- | bc- | ac- | bc- | bc- | ac- | bc- | bc- |
| CH02b07 | 10 | acd | acd | ad | acd | acd | acd | bcd | ad | acd | acd | bc | ac | acd | bcd | acd | bcd | acd | bcd | acd | acd |
| CH02c11 | 10 | ac- | ac- | bc | bc- | ac- | bc- | ac- | bc | bc- | bc- | ac | bd | bc- | bc- | ac- | bc- | bc- | ac- | bc- | bc- |
| MS06g03 | 10 | c-- | bcd | bc | c-- | c-- | c-- | ac- | bc | c-- | c-- | ad | bd | c-- | acd | c-- | ac- | c-- | ac- | c-- | c-- |
| CH02a10 | 10 | lll | lll | ll | lll | lll | lll | lll | ll | lll | lll | lm | ll | lll | lll | lll | lll | lll | lll | lll | lll |

| Markers | LG | Aneuploid seedlings from the cross of 'Gala × Fuji' | | | | | | | | | | | | | | | | | | | |
|---|---|---|---|---|---|---|---|---|---|---|---|---|---|---|---|---|---|---|---|---|---|
| | | GF21 | GF22 | GF23 | GF24 | GF25 | GF26 | GF27 | GF28 | GF29 | GF30 | GF31 | GF32 | GF33 | GF34 | GF35 | GF36 | GF37 | GF38 | GF39 | GF40 |
| CH03d11 | 10 | llm | llm | ll | llm | lm | llm | llm | ll | llm | llm | lm | lm | llm | llm | llm | llm | llm | llm | llm | llm |
| CH04c06y_1 | 10 | llm | llm | lm | llm | llm | llm | lll | lm | llm | llm | ll | lm | llm | lll | llm | lll | llm | lll | llm | llm |
| Hi02d04 | 10 | lll | lll | lm | lll | lll | lll | llm | lm | llm | lll | lm | lm | llm | lll | lll | lll | llm | llm | lll | lll |
| Hi04f08 | 10 | nnp | nnp | np | nnp | nnp | nnp | nnp | np | nnp | nnp | nn | nn | nnp | nnp | nnp | nnp | nnp | nnp | nnp | nnp |
| MS02a01 | 10 | nnp | nnp | nn | nnp | nnp | nnp | nnp | nn | nnp | nnp | nn | np | nnp | nnp | nnp | nnp | nnp | nnp | nnp | nnp |
| CH02d08 | 11 | ad | ac | ac | ad | bcd | ac | bcd | ad | bd | acd | bd | acd | ad | ac | ad | acd | acd | ad | bc | ad |
| CH04g07 | 11 | bd | ac | ad | bc | acd | bd | bcd | ad | ad | bcd | ad | bcd | ad | bc | bc | bcd | acd | bd | ac | bd |
| CH04h02_1 | 11 | ac | bd | bd | bc | ac- | bd | ac- | bc | ac | bc- | ad | ac- | bc | bd | bc | bc- | bc- | bc | ad | bc |
| Hi06b06 | 11 | bc | ad | ac | bd | acd | bc | bcd | ac | ac | bcd | ac | bcd | ac | bd | bd | bcd | acd | bd | ad | bc |
| CH04h02_3 | 11 | np | nn | nn | np | nnp | np | nnp | np | nn | nnp | np | nnp | np | nn | np | nnp | nnp | np | nn | np |
| CH01b12y | 12 | acd | acd | ad | bcd | acd | ac | bc | bc | acd | acd | bcd | bcd | bc | ad | bc | acd | acd | acd | bcd | bcd |
| CH01g12 | 12 | bcd | bcd | bc | acd | bcd | bd | ad | ad | bcd | bcd | acd | acd | ad | bc | ad | bcd | bcd | bcd | acd | acd |
| NZ28f04 | 12 | bcd | acd | bd | acd | bcd | bc | ad | ac | bcd | bcd | acd | acd | ac | bd | ac | bcd | bcd | bcd | acd | acd |
| CH01f02 | 12 | efg | eef | eg | eeg | efg | ee | fg | ef | efg | efg | eef | efg | fg | eg | ef | efg | eef | efg | eef | eef |
| CH05d04 | 12 | eef | eef | eg | efg | eef | fg | ee | ef | eeg | efg | efg | efg | ee | eg | ef | efg | efg | eeg | eef | eef |
| CH05d11 | 12 | efg | eeg | ef | efg | efg | fg | ee | eg | efg | efg | eeg | efg | ee | ef | eg | efg | eeg | efg | eeg | eeg |
| CH03h03z_2 | 12 | nnp | nnp | nn | nnp | nnp | np | nn | np | nnp | nnp | nnp | nnp | np | nn | np | nnp | nnp | nnp | nnp | nnp |
| CH03a08 | 13 | ac | ad | ac | bcd | bd | ac | ac | bcd | acd | ac | bcd | acd | acd | bcd | ac | acd | acd | ac | acd | acd |
| CH03h03z_1 | 13 | bc | bd | bc | ac- | ad | bc | bc | ac- | c-- | bc | ac- | c-- | c-- | ac- | bc | c-- | c-- | bc | c-- | c-- |
| CH05c06_1 | 13 | bd | ac | bd | bc- | bc | ad | ad | c-- | c-- | bd | c-- | c-- | bc- | bc- | ac | bc- | c-- | bd | bc- | bc- |
| CH05f04 | 13 | ad | bc | ad | ac- | ac | bd | bd | c-- | c-- | ad | c-- | c-- | ac- | ac- | bc | ac- | c-- | ad | ac- | ac- |
| CH05h05 | 13 | ac | bd | ac | acd | ad | bc | bc | bcd | bcd | ac | bcd | bcd | acd | acd | bd | acd | bcd | ac | acd | acd |
| Hi03e04 | 13 | bd | ac | bd | bc- | ac | bd | bd | ac- | bc- | ac | bc- | bc- | ac- | bc- | bd | bc- | ac- | bd | bc- | bc- |
| Hi20b03 | 13 | bc | ad | bc | acd | ad | bc | ac | acd | bcd | bc | acd | bcd | bcd | acd | bd | bcd | bcd | bc | bcd | bcd |
| NH009b | 13 | ad | bc | ad | bcd | bc | ad | bd | bcd | acd | ad | bcd | acd | acd | bcd | ac | acd | acd | ad | acd | acd |
| AU223486 | 13 | k- | k- | k- | hhk | hh | k- | k- | hkk | hhk | k- | hhk | hhk | hkk | hkk | k- | hkk | hhk | k- | hk- | hk- |
| GD147 | 13 | hk | k- | hk | hkk | hk | hk | k- | hkk | hhk | hk | hkk | hhk | hhk | hkk | hh | hhk | hkk | hk | hk- | hk- |
| Hi05c06_2 | 13 | hk | h- | hk | hkk | hk | hk | h- | hhk | hkk | hk | hhk | hkk | hkk | hhk | kk | hkk | hhk | hk | hk- | hk- |
| Hi07b02_3 | 13 | lm | ll | lm | lll | ll | lm | ll | lll | llm | lm | lll | lll | lll | lll | lm | llm | llm | lm | llm | llm |
| NZ03c01x_2 | 13 | ll | lm | ll | lll | ll | lm | ll | lll | llm | lm | lll | lll | lll | lll | ll | llm | llm | lm | llm | llm |
| CH01g05 | 14 | bc | bcd | acd | bd | ac | ad | bc | bc | ac | bd | ac | bcd | acd | acd | bd | bd | ad | acd | bcd | ad |
| CH03a02 | 14 | bd | bcd | acd | bd | bc | ac | bd | bd | ad | bc | ad | bcd | acd | acd | bc | bc | ac | acd | bcd | ad |
| CH03d08 | 14 | ad | acd | bcd | ac | bd | bc | ad | ad | bd | ac | bd | acd | bcd | bcd | ac | ac | bc | bcd | acd | bc |
| CH05g07z_1 | 14 | k- | hhk | hkk | k- | hk | k- | hk | k- | k- | hh | k- | hkk | hhk | hkk | hh | hh | k- | hhk | hk- | k- |

| Markers | LG | Aneuploid seedlings from the cross of 'Gala × Fuji' | | | | | | | | | | | | | | | | | | | |
|---|---|---|---|---|---|---|---|---|---|---|---|---|---|---|---|---|---|---|---|---|---|
| | | GF21 | GF22 | GF23 | GF24 | GF25 | GF26 | GF27 | GF28 | GF29 | GF30 | GF31 | GF32 | GF33 | GF34 | GF35 | GF36 | GF37 | GF38 | GF39 | GF40 |
| CH05g07z_2 | 14 | h- | hkk | hhk | h- | hk | h- | hk | h- | h- | kk | h- | hhk | hhk | hkk | kk | kk | h- | hkk | hk- | h- |
| CH02c02a_1 | 15 | bc | bc | ac- | bd | ac- | bc- | ad | ac | ac- | bc- | ac- | bd | ac- | bc- | ad | ac | ac- | ad | bc- | ac- |
| CH02d11 | 15 | ac | ac | bcd | ad | ac- | ac- | bd | ac | acd | acd | acd | ac | acd | acd | bc | bc | acd | bd | acd | bcd |
| CH03b10 | 15 | bc | bd | acd | bd | bcd | bcd | ad | bc | bcd | bcd | bcd | bc | bcd | bcd | ac | ac | bcd | ac | bcd | acd |
| Hi04c05 | 15 | bd | bd | ac- | bc | bc- | bc- | ac | bd | bc- | bc- | bc- | bc | bc- | bc- | ad | ad | bc- | ac | bc- | ac- |
| Hi06f09 | 15 | ad | ad | acd | ac | acd | acd | bc | bd | bcd | acd | acd | ad | acd | ad | bd | bd | bcd | bc | acd | bcd |
| NZ02b01 | 15 | bd | bd | ac- | bc | bc- | bc- | ac | bd | bc- | bc- | bc- | bc | bc- | bc- | ad | ad | bc- | ac | bc- | ac- |
| CH02c09 | 15 | ll | ll | llm | ll | lll | lll | lm | ll | lll | llm | lll | ll | llm | lll | lm | lm | lll | lm | lll | llm |
| Hi02g06 | 15 | nn | np | np1p2 | nn | np1p2 | np1p2 | nn | np | np1p2 | np1p2 | np1p2 | nn | np1p2 | np1p2 | np | np | np1p2 | nn | np1p2 | np1p2 |
| CH02d10a | 16 | ad- | ac | bc- | ad | bc- | bc- | ac- | ac- | bc- | bc- | ac- | ac- | ac- | bc- | ac- | bc | bc- | bc- | ac- | ac- |
| CH04f10 | 16 | ac- | bc | bc- | ad | bc- | ac- | ac- | bc- | ac- | bc- | ac- | bc- | bc- | ac- | bc- | ac | ac- | ac- | bc- | ac- |
| CH05a04 | 16 | bcd | ad | acd | bc | acd | bcd | bcd | acd | bcd | acd | bcd | bcd | acd | bcd | acd | bd | acd | bcd | acd | bcd |
| CH05c06_2 | 16 | bcd | bd | acd | bc | acd | bcd | bcd | acd | bcd | acd | bcd | bcd | acd | bcd | bcd | bc | acd | bcd | bcd | bcd |
| Hi01d06y | 16 | bcd | ac | acd | bd | acd | bcd | bcd | acd | bcd | acd | bcd | acd | acd | bcd | ad | bc | bcd | bcd | acd | bcd |
| Hi04e04 | 16 | acd | ac | bcd | ad | bcd | acd | acd | bcd | acd | bcd | acd | bcd | bcd | acd | acd | ad | bcd | acd | bcd | acd |
| CH05b06z_1 | 16 | efg | ef | efg | fg | eef | efg | eef | eef | eeg | efg | eef | eef | efg | efg | eef | fg | eef | efg | eef | eef |
| Hi01c11x | 16 | efg | eg | efg | ee | eeg | eeg | eeg | eeg | efg | efg | eeg | eeg | efg | eeg | efg | ee | efg | efg | eeg | eeg |
| CH01h01 | 17 | bcd | bcd | acd | bd | acd | bcd | bcd | acd | acd | acd | acd | ac | bcd | bcd | bcd | bc | acd | acd | ad | bcd |
| CH04c06y_2 | 17 | bc- | bc- | c-- | ad | c-- | bc- | bc- | bc- | bc- | c-- | c-- | bd | bc- | bc- | c-- | ad | c-- | c-- | c-- | bc- |
| CH05d08y_1 | 17 | ac- | ac- | c-- | bc | c-- | ac- | ac- | ac- | ac- | c-- | c-- | ad | ac- | ac- | c-- | bc | c-- | c-- | c-- | ac- |
| CH05g03 | 17 | acd | acd | bcd | ad | bcd | acd | acd | bcd | bcd | acd | bcd | bc | acd | acd | ac | ac | bcd | bcd | bcd | acd |
| CH04c06y_3 | 17 | llm | lll | llm | lm | llm | lll | lll | llm | lll | llm | llm | lm | lll | llm | llm | ll | llm | llm | llm | llm |
| GD96 | 17 | lll | lll | lll | lm | lll | lll | lll | lll | lll | lll | lll | ll | lll | lll | lll | lm | lll | lll | lll | lll |
| Hi03c05 | 17 | lll | lll | llm | ll | lll | lll | llm | llm | llm | lll | llm | lm | llm | lll | lll | ll | llm | llm | lll | lll |
| Hi05c06_1 | 17 | llm | llm | lll | lm | lll | llm | llm | llm | llm | lll | lll | lm | llm | llm | lll | ll | lll | lll | lll | lm |
| Hi07b02_1 | 17 | llm | lll | llm | ll | llm | lll | llm | lll | llm | lll | llm | lm | llm | llm | llm | lm | llm | llm | lll | lll |
| Hi07b02_2 | 17 | nnp | nnp | nnp | nn | nnp | nnp | nnp | nnp | nnp | nnp | nnp | np | nnp | nnp | nnp | np | nnp | nnp | nnp | nnp |

Note: '-' represents a null allele, or missing data; 'p1' and 'p2' are con-dominant alleles.
