# Supplementary material for: Molecular Genetic Features of Polyploidization and Aneuploidization Reveal Unique Patterns for Genome Duplication in Diploid Malus
Source: PLoS One. 2012 Jan 10;7(1):e29449. doi: 10.1371/journal.pone.0029449 (PMC3254611; doi:10.1371/journal.pone.0029449)
Supplement: Table S2 — The distributional features of microsatellite markers in the aneuploid seedlings from the cross of ‘Fuji×Gala’. (PDF) [file pone.0029449.s003.pdf]

Markers	LG	Aneuploid seedlings from the cross of ‘Fuji × Gala’																		
		FG01	FG02	FG03	FG04	FG05	FG06	FG07	FG08	FG09	FG10	FG11	FG12	FG13	FG14	FG15	FG16	FG17	FG18	FG19
CH05g08	1	ad	bd	ad	bd	bd	ac	bd	bc	bc	bd	ad	bc	ad	bd	ac	ad	ac	ac	bc
Hi07d08	1	bc	ac	bc	ac	ac	bc	ac	ad	ad	ac	bc	ad	bc	ac	bd	bc	bd	bd	bc
Hi12c02	1	ad	ad	bd	ad	ad	bc	ad	bc	ad	ad	bc	ad	ad	bc	ad	bc	bc	bc	bd
KA4B	1	h-	kk	h-	h-	kk	h-	kk	h-	kk	kk	h-	kk	kk	h-	h-	h-	h-	h-	h-
Hi02b10	1	ll	lm	ll	ll	lm	lm	lm	ll	ll	lm	ll	ll	lm	ll	ll	ll	lm	lm	ll
Hi02c07	1	nn	np	nn	nn	np	np	np	nn	nn	np	nn	nn	np	nn	nn	nn	np	np	nn
CH02a04z	2	ad	bc	ad	ac	ad	ac	ad	ac	ad	bcd	ac	acd	bcd	acd	acd	ad	ac	bcd	bd
CH02c02a_3	2	ac	bc	ac	ad	ad	ad	ac	ad	ac	acd	bc	acd	bcd	acd	bcd	ac	ac	bcd	bc
CH02c06	2	ac	ac	bc	bd	ac	bd	ac	bd	ac	bcd	ad	acd	bcd	bcd	acd	ac	bd	bcd	bc
CH03d01	2	bd	bc	ad	ac	bd	ac	bd	ac	bd	ac-	bc	c--	ac-	c--	c--	bd	ad	ac-	ad
CH05e03	2	bc	bd	bc	ad	bc	bd	bc	bc	bc	acd	bd	bcd	acd	bcd	bcd	bc	bc	acd	ac
CN493139	2	bc	bd	bc	ac	ad	ad	ad	ac	bc	ac-	bd	bc-	ac-	bc-	ac-	ad	bc	bc-	ac
Hi02a07	2	ac	ad	ac	bd	ac	ad	ac	ac	bc	c--	ad	ac-	c--	ac-	ac-	ac	ac	c--	bc
Hi05c06_3	2	lm	ll	lm	ll	lm	ll	lm	lm	lm	llm	lm	lll	lll	llm	lll	lm	lm	llm	lm
CH02c02a_2	2	nn	nn	nn	np	nn	nn	nn	nn	np	np1p2	nn	np1p2	np1p2	np1p2	np1p2	nn	nn	np1p2	np
CH03d10	2	nn	nn	nn	np	nn	nn	nn	nn	np	np1p2	nn	np1p2	np1p2	np1p2	np1p2	nn	nn	np1p2	np
CH03e03	3	bd	bd	ad	ad	ad	bc	ac	ac	ad	bc	bd	bd	bc-	bc	ad	bc-	bc	bc-	ac-
CH03g07	3	ad	ad	bc	bd	ad	ac	ac	ac	bc	bc	ad	bc	bcd	bc	ad	acd	bc	bcd	acd
HGA8bY	3	ad	bd	bd	bd	ad	bc	ac	ac	ad	bc	bc	ad	bcd	bc	ad	bcd	ac	bcd	bcd
Hi04c10x_1	3	bd	bd	ac	bd	bd	bc	bd	bc	ac	ac	bd	ac	ac-	bc	bd	c--	ac	ac-	c--
Hi07e08x	3	bc	bc	ac	ac	bc	ad	bd	bd	bc	ad	ad	ac	ac-	ad	bc	ac-	ad	ac-	c--
AU223657	3	np	np	nn	np	np	nn	np	np	np	nn	nn	nn	np1p2	ad	bc	np1p2	nn	np1p2	np1p2
CH02c02b	4	ac	ac	bd	ad	ac	bc	acd	ac	bcd	ad	ad	acd	bcd	acd	ad	ac	acd	bc	bcd
CH05d02	4	ac	ac	bd	ad	ac	bc	acd	bc	acd	ad	ad	acd	acd	bcd	ac	ac	acd	ad	bcd
GD162	4	ac	ac	bd	ad	ac	bc	acd	bc	acd	ad	ad	acd	acd	bcd	ac	ac	acd	ad	---
Hi04c10x_3	4	bc	bc	ad	bd	bc	ac	c--	ad	c--	bd	bd	c--	c--	ac-	bc	bc	c--	bd	ac-
CH04e02	4	ee	ef	fg	fg	fg	eg	eef	eg	efg	ef	ef	efg	eef	eef	ef	fg	efg	ef	eef
Hi07b02_4	4	ee	ee	fg	eg	ee	ef	eeg	ef	efg	eg	eg	efg	eeg	eeg	eg	ee	efg	eg	eeg
CH03a04	5	ad	ac	bd	ad	bd	ad	bcd	bc	bd	bd	bcd	ac	ac	ac	bcd	bc	bcd	acd	bc
CH04g09y	5	bc	bc	bc	bc	ad	bd	acd	ad	ad	ac	bcd	bc	bc	bc	bcd	bc	acd	bcd	bc
CH05e06	5	ad	ad	ad	bd	bc	ac	bcd	bc	bc	bd	acd	ad	ad	ad	acd	ad	bcd	acd	ad
CH03a09	5	ef	fg	ef	fg	ee	ee	eeg	ee	ee	eg	afg	fg	fg	fg	eeg	fg	eeg	efg	eg
CH04e03	5	fg	eg	eg	fg	ef	fg	efg	ef	fg	fg	eeg	eg	eg	eg	eeg	eg	efg	eeg	eg
CH04h02_2	5	k-	hk	hk	k-	hk	hk	hhk	k-	hh	hk	hkk	hk	hk	hk	hhk	hk	hkk	hhk	hk-

Markers	LG	Aneuploid seedlings from the cross of 'Fuji × Gala'																		
		FG01	FG02	FG03	FG04	FG05	FG06	FG07	FG08	FG09	FG10	FG11	FG12	FG13	FG14	FG15	FG16	FG17	FG18	FG19
CH04h02_4	5	h-	h-	h-	kk	kk	h-	hk-	h-	h-	h-	hk-	h-	h-	h-	hk-	kk	hk-	hk-	h-
Hi04d02	5	k-	hk	hk	k-	hk	hk	hhk	k-	hh	hk	hhk	hk	hk	hk	hkk	hk	hhk	hhk	hk-
Hi11a03	5	hk	k-	k-	hk	hk	hk	hkk	k-	hh	hk	hhk	hk	hk	hk	hhk	hk	hkk	hhk	k-
Hi21c08	5	hk	h-	h-	hk	hk	hk	hhk	h-	kk	hk	hkk	hk	h-	hk	hkk	hk	hhk	hkk	h-
CH02a08z	5	ll	ll	ll	ll	lm	ll	lll	lm	ll	ll	llm	ll	ll	ll	llm	ll	llm	lll	ll
CH03d07	6	ac	ac	bd	ac	bc	ac	bcd	ac	ad	bd	acd	ad	ac	acd	acd	ad	acd	bcd	bd
CH03d12	6	ac	bd	bd	bd	ad	bd	acd	bd	bc	ac	bcd	bc	bd	bcd	bcd	bc	bcd	acd	ad
Hi01d05	6	ee	fg	fg	fg	fg	fg	eeg	fg	ee	ee	efg	ef	fg	efg	efg	ee	eeg	eeg	eg
AJ000761	6	k-	k-	k-	k-	k-	k-	hk-	k-	k-	k-	hk-	k-	k-	hk-	hk-	k-	hk-	hk-	k-
CH03c01	6	h-	h-	h-	h-	h-	h-	hk-	h-	h-	h-	hk-	h-	h-	hk-	hk-	h-	hk-	hk-	h-
Hi04c10x_2	7	ac	bd	bd	bc	ad	ac	ad	ad	bd	ac	ac	ac	ac	bc	ad	ad	ad	ad	ad
CH05b06z_2	7	ee	fg	fg	ef	eg	ee	eg	fg	fg	ee	ef	ee	ee	ef	eg	eg	eg	eg	eg
CH04e05	7	nn	np	np	nn	np	np	np	np	nn	np	np	np	np	nn	np	np	np	np	nn
Hi05b09	7	nn	np	np	nn	nn	nn	np	nn	np	nn	nn	nn	nn	np	nn	nn	np	nn	nn
CH01c06	8	ac	bd	bc	ac	ad	ad	bd	bc	ad	ac	ac	bc	ad	ac	bd	ac	ac	bd	ad
Hi04b12	8	ad	bd	bd	ac	bc	bc	ac	ad	bc	bd	bd	ad	bd	bd	ac	bd	bd	ac	ac
Hi23g12	8	ll	ll	ll	lm	lm	lm	lm	ll	lm	ll	ll	ll	lm	ll	lm	ll	ll	lm	lm
CH02g09	8	np	nn	nn	np	np	np	nn	nn	np	np	np	nn	nn	np	nn	np	np	nn	nn
Hi04e05	8	nn	nn	nn	np	nn	nn	nn	np	nn	nn	nn	np	np	nn	np	nn	nn	np	np
CH01h02_2	9	bc	bc	bd	bcd	ad	bd	ac	bc	acd	bcd	acd	bcd	ad	bcd	bcd	acd	bcd	bc	acd
CH05c07	9	ad	bcd	ac	bc	bd	ac	ac	bc	acd	bcd	bcd	acd	ac	bcd	bcd	acd	bcd	ac	acd
GD142	9	bc	bcd	ad	ac	bc	bd	bd	bc	acd	bcd	bcd	bcd	bc	bcd	acd	acd	bcd	ac	acd
Hi05e07	9	ac	acd	ad	ad	bc	ac	bd	ad	c--	ac-	c--	ac-	bc	ac-	ac-	c--	ac-	ad	c--
NH029a	9	ad	acd	ac	ac	bd	ad	bc	ac	bc-	ac-	bc-	ac-	bd	ac-	ac-	bc-	ac-	ac	bc-
CH01h02_1	9	hk	hk-	k-	hk	hh	hk	k-	k-	hhk	hkk	hhk	hhk	hh	hkk	hhk	hhk	hkk	k-	hkk
CH05d08y_2	9	lm	lm-	lm	lm	ll	lm	ll	lm	lll	llm	lll	llm	ll	llm	llm	lll	llm	lm	lll
Hi01d01	9	np	np1p2	np	np	nn	np	nn	np	np1p2	np1p2	np1p2	np1p2	nn	np1p2	np1p2	np1p2	np1p2	np	np1p2
CH01f07a	10	a	bd	ac-	bc	ac-	ac	bd	ac-	bc-	ac	ad	bc-	ac-	bd	bc-	bc-	ad	bc	ac-
CH01f12	10	b	bd	bcd	bd	bcd	bc	bd	bcd	bcd	ad	ad	acd	acd	bc	bcd	acd	ad	bd	acd
CH02b03b	10	a	ac	cd-	ad	cd-	bd	ac	bcd	acd	bd	bc	acd	bcd	ac	acd	acd	bc	ad	bcd
CH02b07	10	a	ac	acd	bc	bcd	bd	bc	bcd	bcd	bc	ac	acd	acd	ad	bcd	acd	ad	bc	acd
CH02c11	10	a	ac	bcd	ad	bcd	bd	ac	bcd	acd	bd	bc	acd	bcd	ac	acd	acd	bc	ad	bcd
MS06g03	10	a	ac	c--	ad	c--	bd	ad	c--	ac-	bd	bc	ac-	c--	ac	ac-	ac-	bc	ad	c--
Hi04f08	10	l	lm	llm	ll	llm	ll	ll	llm	llm	lm	lm	lll	lll	ll	lll	llm	lm	lm	lll

Markers	LG	Aneuploid seedlings from the cross of 'Fuji × Gala'																		
		FG01	FG02	FG03	FG04	FG05	FG06	FG07	FG08	FG09	FG10	FG11	FG12	FG13	FG14	FG15	FG16	FG17	FG18	FG19
MS02a01	10	l	lm	llm	ll	llm	ll	lm	llm	llm	ll	lm	lll	llm	lm	lll	lll	lm	ll	lll
CH02a10	10	n	nn	np1p2	np	np1p2	np	np	np1p2	np1p2	np	nn	np1p2	np1p2	nn	np1p2	np1p2	nn	np	np1p2
CH03d11	10	n	np	np1p2	nn	np1p2	nn	nn	np1p2	np1p2	nn	np	np1p2	np1p2	nn	np1p2	np1p2	np	nn	np1p2
CH04c06y_1	10	n	nn	np1p2	np	np1p2	np	np	np1p2	np1p2	np	nn	np1p2	np1p2	nn	np1p2	np1p2	nn	np	np1p2
Hi02d04	10	n	np	nnp	np	nnp	nn	nn	nnp	nnp	np	np	nnp	nnp	np	nnp	nnp	nn	nn	nnp
CH02d08	11	bd	bc	bc	ad	ad	ac	ac	bc	bd	ad	bd	ac	ad	acd	bc	bc	bd	bd	bd
CH04g07	11	ad	bc	ac	bd	bd	ac	bc	ad	ac	bd	ad	ac	bd	acd	ac	ac	ac	bd	bc
CH04h02_1	11	ac	ad	ad	bc	bc	bd	bd	ad	ac	bc	ac	bd	bc	bcd	bc	ad	ac	ac	ac
Hi06b06	11	ac	bd	ad	bc	bd	ad	bd	ac	ad	bc	ac	ad	bc	ac-	ad	ad	ad	bc	bd
CH04h02_3	11	lm	lm	lm	ll	ll	ll	ll	lm	lm	ll	lm	ll	ll	llm	lm	lm	lm	lm	lm
CH01b12y	12	bc	bd	ac	ad	bd	bd	bc	ad	acd	acd	acd	bcd	bd	ac	ad	bcd	bcd	ac	acd
CH01g12	12	ac	bd	ad	bc	ac	ac	ad	bc	bcd	bcd	bcd	acd	ac	bd	bc	acd	acd	bc	bcd
NZ28f04	12	bd	bc	bc	bd	ac	ad	bc	bd	bcd	bcd	bcd	acd	ad	ac	bd	bcd	acd	bc	acd
CH01f02	12	ee	eg	eg	ef	ef	fg	ee	ee	efg	efg	eef	eef	eg	ef	ee	efg	eef	ee	eef
CH05d04	12	eg	eg	fg	ee	ef	fg	ee	fg	efg	efg	efg	eef	eg	ef	fg	efg	eef	fg	eef
CH05d11	12	ef	ef	fg	eg	eg	ee	fg	fg	efg	efg	eeg	eeg	ef	eg	fg	efg	eeg	fg	eeg
CH03h03z_2	12	lm	ll	ll	lm	ll	lm	ll	lm	lll	lll	llm	llm	lm	ll	lm	lll	llm	ll	lll
CH03a08	13	bd	ad	bd	bc	ac	bc	bd	acd	bc	ac	bcd	acd	bd	ad	acd	ac	bcd	bcd	bc
CH03h03z_1	13	ad	bd	ad	ac	bc	ac	ad	c--	ac	bc	ac-	c--	ad	bd	c--	bc	ac-	ac-	ac
CH05c06_1	13	ac	ad	bc	ac	ad	bd	bd	bc-	bd	bd	bc-	bc-	bc	ac	c--	bc	bc-	bc-	ad
CH05f04	13	bd	ac	bc	bc	bd	ad	ad	ac-	ac	ad	ac-	ac-	ac	bc	c--	ac	ac-	ac-	bd
CH05h05	13	bc	ad	bd	bd	bc	ac	ac	acd	ac	ac	acd	acd	ad	bd	bcd	ad	acd	acd	bc
Hi03e04	13	ac	bc	bc	ad	bd	ad	ac	acd	ad	bd	acd	bcd	ac	bc	acd	ad	bcd	acd	ad
Hi20b03	13	ad	ad	bd	ac	bc	ac	ad	bcd	ac	bc	bcd	acd	ad	ad	bcd	bc	acd	bcd	ac
NH009b	13	bc	ac	bc	bd	ad	bd	bc	acd	bd	ad	acd	bcd	bc	bc	acd	ad	bcd	acd	bd
AU223486	13	hh	k-	hh	hk	k-	hk	hh	hkk	hk	k-	hhk	hkk	hh	k-	hhk	k-	hkk	hhk	hk
GD147	13	k-	k-	hh	k-	hk	k-	k-	hhk	k-	hk	hkk	hhk	hh	k-	hkk	hk	hhk	hkk	k-
Hi05c06_2	13	h-	kk	h-	h-	hk	h-	h-	hhk	h-	hk	hhk	hkk	kk	h-	hhk	hk	hkk	hhk	h-
Hi07b02_3	13	np	np	np	np	nn	np	np	nnp	np	nn	nnp	nnp	nn	np	nnp	nn	nnp	nnp	np
NZ03c01x_2	13	np	nn	np	np	np	np	np	nnp	np	nn	nnp	nnp	np	nn	nnp	nn	nnp	nnp	nn
CH01g05	14	ad	bc	ac	ac	bc	ad	ac	bc	bcd	bc	ac	bd	bcd	bc	bc	ad	bcd	ac	acd
CH03a02	14	bd	ac	ac	ad	bd	bc	ac	bd	bcd	bd	ad	bc	bcd	bd	bd	ac	bcd	ad	acd
CH03d08	14	ad	bd	bc	bd	ad	bc	bd	ad	acd	ad	bd	ac	acd	ad	ad	bc	acd	bd	bcd
CH05g07z_1	14	k-	hk	hk	hk	k-	hk	k-	hk	hhk	k-	hk	hh	hkk	k-	hk	k-	hhk	k-	hkk

Markers	LG	Aneuploid seedlings from the cross of ‘Fuji × Gala’																		
		FG01	FG02	FG03	FG04	FG05	FG06	FG07	FG08	FG09	FG10	FG11	FG12	FG13	FG14	FG15	FG16	FG17	FG18	FG19
CH05g07z_2	14	hk	hk	h-	hk	h-	hk	h-	hk	hkk	h-	hk	kk	hhk	h-	hk	h-	hkk	h-	hhk
CH02c02a_1	15	ac	ad	ad	bd	ac	bcd	bc	ad	bd	bcd	bcd	bd	ac	bcd	ad	bcd	ac	bcd	ac
CH02d11	15	bd	bc	bd	ad	ac	acd	ac	ac	ac	acd	acd	ac	ac	acd	bd	bcd	ad	acd	ac
CH03b10	15	ad	ac	ad	bd	bc	bcd	bc	bc	bc	bcd	acd	bc	bc	bcd	ad	acd	bd	bcd	bc
Hi04c05	15	ad	ac	ac	bc	bd	bcd	bd	bd	bc	bcd	bcd	bc	bd	bcd	ac	bcd	bc	bcd	bd
Hi06f09	15	bd	bc	bc	ac	bd	ac-	ad	bd	ad	ac-	ac-	ac	bd	ac-	bc	ac-	ac	ac-	ad
NZ02b01	15	ad	ac	ac	bc	bd	bcd	bd	bd	bc	bcd	bcd	bc	bc	bcd	ac	bcd	bc	bcd	bd
Hi02g06	15	ll	ll	ll	lm	lm	lll	lm	ll	lm	llm	lll	lm	ll	llm	lm	llm	ll	llm	ll
CH02c09	15	nn	np	nn	np	np	np1p2	np	np	np	np1p2	np1p2	np	np	np1p2	nn	np1p2	np	np1p2	np
CH02d10a	16	ad	ad	bc	ad	ac	acd	ad	bcd	bd	bcd	ac	bc	bc	bcd	bcd	acd	ac	bcd	bd
CH04f10	16	bd	bc	ac	ac	ad	bcd	bc	acd	bc	acd	bd	ad	ad	bcd	bcd	bcd	bd	bcd	ac
CH05a04	16	ac	ad	bc	bc	ac	acd	ad	bcd	ad	bcd	ac	bc	bc	acd	acd	bcd	ac	acd	bd
CH05c06_2	16	ad	bd	bc	bc	ac	bcd	ad	acd	ad	bcd	ac	bc	bc	bcd	acd	acd	bd	acd	bc
Hi01d06y	16	ad	ac	bc	bc	bd	acd	ad	bcd	ac	bcd	ad	bd	bd	acd	acd	acd	ad	acd	bc
Hi04e04	16	bc	bc	ad	ad	bd	acd	bc	acd	bc	acd	bd	ad	ad	acd	bcd	bcd	ac	bcd	ac
CH05b06z_1	16	fg	ee	ef	fg	eg	eef	ee	efg	ee	eef	eg	eg	fg	eef	efg	efg	ef	efg	fg
Hi01c11x	16	eg	eg	ee	ee	ef	eeg	eg	eeg	fg	eeg	ef	ef	ee	eeg	efg	eeg	eg	efg	ee
CH01h01	17	ad	bd	ac	bcd	bd	acd	ac	bc	acd	bcd	bd	ac	acd	bc	bc	acd	bd	ac	acd
CH04c06y_2	17	bc	ad	ad	c--	bc	bc-	ac	bc	bc-	bc-	bc	ad	c--	bc	bc	c--	ad	bc	c--
CH05d08y_1	17	bc	bc	ad	c--	ad	ac-	bd	ad	ac-	ac-	ad	bc	c--	ad	ad	c--	bc	ad	c--
CH05g03	17	bd	ac	bc	acd	ad	bcd	bc	ac	bcd	acd	ad	bc	bcd	ac	ac	bcd	ad	bc	bcd
Hi07b02_2	17	ll	ll	ll	lll	lm	lll	ll	ll	llm	lll	lm	ll	lll	ll	lm	llm	ll	lm	lll
CH04c06y_3	17	np	np	nn	np1p2	np	np1p2	np	np	np1p2	np1p2	np	np	np1p2	np	nn	np1p2	np	nn	np1p2
GD96	17	nn	np	np	np1p2	np	np1p2	nn	nn	np1p2	np1p2	np	np	np1p2	np	np	np1p2	np	nn	np1p2
Hi03c05	17	np	np	nn	nnp	np	nnp	nn	nn	nnp	nnp	nn	np	nnp	np	np	nnp	np	nn	nnp
Hi05c06_1	17	np	np	nn	nnp	nn	nnp	np	nn	nnp	nnp	nn	np	nnp	nn	nn	nnp	np	nn	nnp
Hi07b02_1	17	np	nn	nn	nnp	nn	nnp	nn	nn	nnp	nnp	nn	np	nnp	np	nn	nnp	nn	np	nnp

Markers	LG	Aneuploid seedlings from the cross of 'Fuji × Gala'																	
		FG20	FG21	FG22	FG23	FG24	FG25	FG26	FG27	FG28	FG29	FG30	FG31	FG32	FG33	FG34	FG35	FG36	FG37
CH05g08	1	ad	bc	ac	ad	bd	bc	ad	bc	ad	bc	ad	ac	ad	ac	ad	ac	bd	ad
Hi07d08	1	ad	ad	bd	bc	ac	ad	bc	ad	bc	ad	bc	bd	bc	bd	bc	bd	ac	bc
Hi12c02	1	ad	bd	bc	bd	ac	ac	bc	ac	ad	ac	bc	bc	ad	bc	bd	bc	ad	bd
KA4B	1	kk	kk	h-	h-	hk	hk	h-	hk	kk	hk	h-	h-	kk	h-	h-	h-	kk	h-
Hi02b10	1	lm	ll	ll	lm	--	lm	lm	ll	lm	lm	ll	ll	lm	ll	ll	ll	ll	lm
Hi02c07	1	np	nn	nn	np	np	np	np	nn	np	np	nn	nn	np	nn	nn	nn	nn	np
CH02a04z	2	bd	bd	bd	acd	bc	acd	bc	ac	bc	acd	bcd	bcd	bcd	bcd	ad	bcd	bcd	bcd
CH02c02a_	2	bc	bd	bc	bcd	ac	acd	bd	ad	bd	acd	bcd	bcd	bcd	bcd	bc	bcd	acd	bcd
CH02c06	2	bc	bc	bc	acd	ac	acd	bd	ad	bd	acd	bcd	bcd	bcd	bcd	bc	bcd	bcd	bcd
CH03d01	2	ad	ad	ad	c--	ac	c--	ac	bc	ac	c--	ac-	ac-	ac-	ac-	bd	ac-	ac-	ac-
CH05e03	2	ac	ac	ac	bcd	ad	bcd	ad	bd	ad	bcd	acd	acd	acd	acd	bc	acd	acd	acd
CN493139	2	bc	bc	ac	ac-	ad	ac-	bc	bc	bd	ac-	bc-	bc-	bc-	ac-	ac	ac-	ac-	bc-
Hi02a07	2	bc	bc	bc	ac-	ad	ac-	bd	bd	bd	ac-	c--	c--	c--	c--	ac	c--	c--	c--
Hi05c06_3	2	lm	lm	lm	lll	ll	lll	ll	ll	ll	lll	llm	lll	lll	llm	lm	llm	lll	lll
CH02c02a_	2	np	np	np	np1p2	np	np1p2	np	np	np	np1p2	np1p2	np1p2	np1p2	np1p2	np	np1p2	np1p2	np1p2
CH03d10	2	np	np	np	np1p2	np	np1p2	np	np	np	np1p2	np1p2	np1p2	np1p2	np1p2	np	np1p2	np1p2	np1p2
CH03e03	3	ad	bc	bd	ac-	ac	bc-	bc-	ad	bc-	ac	bd	bc-	ac-	bc	ac-	bc-	ac-	ad
CH03g07	3	bc	bc	ad	acd	ac	bcd	bcd	bd	bcd	bc	bd	bcd	acd	bc	acd	bcd	acd	ad
HGA8bY	3	bc	ac	bd	bcd	bd	bcd	bcd	bd	bcd	bd	ac	acd	acd	ac	bcd	acd	bcd	bc
Hi04c10x_1	3	ac	ad	bd	c--	bd	ac-	ac-	ad	ac-	ac	bd	ac-	c--	bc	ac-	c--	c--	ad
Hi07e08x	3	ad	bd	ac	bc-	bd	ac-	ac-	bc	ac-	bd	ac	ac-	bc-	ad	bc-	ac-	bc-	bc
AU223657	3	np	np	nn	np1p2	np	np1p2	np1p2	nn	np1p2	nn	np	np1p2	np1p2	nn	np1p2	np1p2	np1p2	nn
CH02c02b	4	bc	bd	bcd	ad	acd	acd	bcd	bd	bcd	acd	bcd	bcd	bcd	bcd	acd	bcd	bcd	bcd
CH05d02	4	bc	bd	bcd	ac	bcd	acd	acd	ac	bcd	acd	bcd	bcd	bcd	bcd	acd	bcd	bcd	bcd
GD162	4	bc	bd	bcd	ac	bcd	acd	acd	ac	bcd	acd	bcd	bcd	bcd	bcd	acd	bcd	bcd	bcd
Hi04c10x_3	4	ac	ad	ac-	bc	ac-	c--	c--	bc	ac-	ac-	ac-	ac-	ac-	ac-	c--	ac-	ac-	ac-
CH04e02	4	ee	eg	efg	eg	efg	efg	eef	fg	efg	eef	efg	efg	eef	eef	eef	eef	efg	eef
Hi07b02_4	4	ef	fg	efg	ef	efg	efg	eeg	ee	efg	eeg	efg	efg	eeg	eeg	eeg	eeg	efg	eeg
CH03a04	5	bcd	bcd	acd	bcd	acd	acd	acd	acd	acd	acd	ad	bd	acd	bcd	acd	bcd	bcd	acd
CH04g09y	5	acd	acd	acd	acd	bcd	bcd	bcd	bcd	acd	bcd	bc	ad	acd	acd	bcd	acd	acd	acd
CH05e06	5	bcd	acd	bcd	bcd	acd	acd	acd	acd	bcd	acd	ad	bc	bcd	bcd	acd	acd	bcd	bcd
CH03a09	5	eeg	eeg	efg	eeg	efg	efg	efg	efg	efg	efg	ef	eg	efg	eeg	efg	eeg	eeg	eeg
CH04e03	5	efg	efg	efg	efg	eeg	eeg	eeg	eeg	efg	eeg	eg	ef	efg	efg	eeg	efg	efg	efg
CH04h02_2	5	hkk	hhk	hkk	hhk	hhk	hkk	hhk	hhk	hkk	hhk	k-	k-	hkk	hkk	hhk	hkk	hhk	hkk

Markers	LG	Aneuploid seedlings from the cross of 'Fuji × Gala'																	
		FG20	FG21	FG22	FG23	FG24	FG25	FG26	FG27	FG28	FG29	FG30	FG31	FG32	FG33	FG34	FG35	FG36	FG37
CH04h02_4	5	hk-	hk-	hk-	hk-	hk-	hk-	hk-	hk-	hk-	hk-	h-	kk	hk-	hk-	hk-	hk-	hk-	hk-
Hi04d02	5	hhk	hkk	hkk	hhk	hkk	hhk	hkk	hhk	hkk	hhk	k-	k-	hkk	hhk	hkk	hhk	hkk	hhk
Hi11a03	5	hkk	hkk	hhk	hhk	hkk	hkk	hhk	hkk	hhk	hkk	k-	k-	hkk	hkk	hhk	hhk	hkk	hkk
Hi21c08	5	hkk	hhk	hkk	hhk	hkk	hkk	hhk	hkk	hkk	hhk	h-	h-	hhk	hkk	hkk	hhk	hkk	hhk
CH02a08z	5	lll	llm	llm	lll	lll	lll	llm	lll	llm	lll	ll	lm	lll	lll	llm	lll	lll	lll
CH03d07	6	bc	acd	acd	ad	bd	bcd	acd	ac	ac	bc	bcd	bcd	acd	bcd	acd	bcd	bcd	bcd
CH03d12	6	ac	bcd	bcd	bc	ac	acd	bcd	bd	bd	ad	acd	acd	bcd	acd	bcd	acd	acd	acd
Hi01d05	6	ee	efg	efg	ef	ee	efg	efg	fg	fg	eg	eeg	eeg	efg	eeg	efg	eeg	eeg	eeg
AJ000761	6	k-	hk-	hk-	k-	k-	hk-	hk-	k-	k-	hk-	hk-	hk-	hk-	hk-	hk-	hk-	hk-	hk-
CH03c01	6	h-	hk-	hk-	h-	h-	hk-	hk-	h-	h-	h-	hk-	hk-	hk-	hk-	hk-	hk-	hk-	hk-
Hi04c10x_2	7	bc	bd	ac	bc	bd	bd	ad	bd	bc	ad	ac	ad	acd	ad	bd	ad	ad	bd
CH05b06z_2	7	ef	fg	ee	ef	fg	fg	eg	fg	ef	eg	ee	eg	efg	eg	fg	eg	eg	fg
CH04e05	7	np	nn	np	nn	nn	nn	np	nn	nn	np	np	np	np1p2	np	nn	np	np	nn
Hi05b09	7	np	np	np	np	np	np	nn	np	np	nn	nn	nn	nnp	nn	np	nn	nn	np
CH01c06	8	bd	ad	ac	ad	bd	bd	bd	bd	ad	ac	ad	ac	bc	ad	bc	ad	ac	bc
Hi04b12	8	bc	bc	bd	bc	ac	ac	ac	ac	bc	bc	bd	bd	ad	bc	ad	bc	bd	ad
Hi23g12	8	lm	lm	ll	lm	lm	lm	lm	lm	lm	lm	ll	ll	ll	lm	ll	lm	ll	ll
CH02g09	8	nn	np	np	np	nn	nn	np	nn	np	np	np	np	nn	np	nn	np	np	np
Hi04e05	8	np	nn	nn	nn	np	np	nn	np	nn	nn	nn	nn	np	nn	np	nn	nn	nn
CH01h02_2	9	bcd	ac	acd	bcd	acd	bd	bcd	bcd	bd	bcd	acd	bcd	bcd	acd	bcd	acd	acd	acd
CH05c07	9	acd	bc	acd	acd	acd	bd	acd	acd	bd	acd	bcd	bcd	acd	bcd	bcd	bcd	bcd	ad
GD142	9	bcd	ac	acd	bcd	acd	bc	bcd	bcd	ac	bcd	bcd	acd	bcd	acd	bcd	acd	acd	bcd
Hi05e07	9	ac-	bd	c--	ac-	c--	ac	ac-	ac-	ac	ac-	c--	ac-	ac-	c--	ac-	c--	c--	c--
NH029a	9	ac-	bc	bc-	ac-	ac-	ad	ac-	ac-	ad	ac-	ac-	ac-	ac-	bc-	ac-	bc-	bc-	bc-
CH01h02_1	9	hhk	hh	hkk	hhk	hkk	hk	hkk	hhk	hk	hkk	hkk	hhk	hhk	hhk	hkk	hkk	hhk	hkk
CH05d08y_2	9	llm	ll	lll	llm	lll	lm	llm	lll	lm	llm	llm	llm	llm	lll	llm	lll	lll	lll
Hi01d01	9	np1p2	nn	np1p2	np1p2	np1p2	np	np1p2	np1p2	np	np1p2	np1p2	np1p2	np1p2	np1p2	np1p2	np1p2	np1p2	np1p2
CH01f07a	10	bc-	ac-	ad	ac-	bc-	ad	ac-	ac-	bc	ac-	ac-	ac-	ac-	bc-	bd	bc-	ac-	bc-
CH01f12	10	bcd	acd	ac	bcd	bcd	ad	acd	bcd	bc	bcd	acd	bcd	acd	bcd	bc	bcd	acd	acd
CH02b03b	10	acd	bcd	bc	bcd	acd	bc	bcd	bcd	ad	bcd	bcd	bcd	bcd	acd	ac	acd	bcd	acd
CH02b07	10	bcd	acd	ad	bcd	acd	ad	acd	bcd	bd	bcd	acd	bcd	acd	bcd	bd	bcd	acd	acd
CH02c11	10	acd	bcd	bc	bcd	acd	bc	bcd	bcd	ad	bcd	bcd	bcd	bcd	acd	ac	acd	bcd	acd
MS06g03	10	ac-	bc-	bc	c--	ac-	bc	c--	c--	ad	c--	c--	c--	c--	ac-	ac	ac-	c--	ac-
Hi04f08	10	lll	lll	ll	lll	lll	ll	lll	lll	ll	lll	lll	lll	lll	lll	ll	lll	lll	llm

Markers	LG	Aneuploid seedlings from the cross of ‘Fuji × Gala’																	
		FG20	FG21	FG22	FG23	FG24	FG25	FG26	FG27	FG28	FG29	FG30	FG31	FG32	FG33	FG34	FG35	FG36	FG37
MS02a01	10	lll	lll	lm	lll	lll	lm	lll	llm	ll	lll	lll	lll	llm	lll	lm	lll	lll	lll
CH02a10	10	np1p2	np1p2	nn	np1p2	np1p2	nn	np1p2	np1p2	np	np1p2	np1p2	np1p2	np1p2	np1p2	np	np1p2	np1p2	np1p2
CH03d11	10	np1p2	np1p2	np	np1p2	np1p2	np	np1p2	np1p2	nn	np1p2	np1p2	np1p2	np1p2	np1p2	nn	np1p2	np1p2	np1p2
CH04c06y_1	10	np1p2	np1p2	nn	np1p2	np1p2	nn	np1p2	np1p2	np	np1p2	np1p2	np1p2	np1p2	np1p2	np	np1p2	np1p2	np1p2
Hi02d04	10	nnp	nnp	nn	nnp	nnp	np	nnp	nnp	np	nnp	nnp	nnp	nnp	nnp	np	nnp	nnp	nnp
CH02d08	11	ad	ac	ac	ad	acd	ad	acd	bcd	acd	bd	ac	bc	bd	bcd	bd	bc	bd	acd
CH04g07	11	ad	ac	ac	bd	bcd	bd	bcd	bcd	bcd	bd	ac	ac	ad	acd	ad	ad	bd	bcd
CH04h02_1	11	bc	bd	bd	bc	bcd	bc	bcd	acd	bcd	ac	bd	ad	ac	acd	ac	ad	ac	bcd
Hi06b06	11	ac	ad	ad	bc	bc-	bc	bc-	bc-	bc-	bc	ad	ad	ac	ac-	ac	ac	bc	bc-
CH04h02_3	11	ll	ll	ll	ll	llm	lm	llm	lll	llm	lm	ll	lm	lm	lll	lm	lm	lm	llm
CH01b12y	12	bcd	acd	acd	bd	acd	acd	acd	acd	bcd	bcd	acd	ac	ad	acd	ad	acd	bd	bcd
CH01g12	12	acd	bcd	bcd	ac	bcd	bcd	bcd	bcd	acd	acd	bcd	bd	bc	bcd	bc	bcd	ac	acd
NZ28f04	12	bcd	bcd	bcd	ad	bcd	bcd	bcd	bcd	acd	acd	bcd	bd	bd	bcd	bd	bcd	bd	acd
CH01f02	12	eef	efg	efg	fg	eef	efg	efg	efg	eef	eef	efg	eg	ef	efg	eg	efg	eg	eef
CH05d04	12	eef	efg	efg	ee	eef	efg	efg	efg	eef	eef	efg	eg	ef	efg	eg	efg	eg	eef
CH05d11	12	eeg	efg	efg	ee	eeg	efg	efg	efg	eeg	eeg	efg	ef	eg	efg	ef	efg	ef	eeg
CH03h03z_2	12	llm	lll	llm	lm	lll	lll	lll	lll	llm	lll	llm	lm	lm	llm	lm	llm	lm	lll
CH03a08	13	ad	acd	bcd	bcd	bd	ac	ad	ad	acd	bcd	acd	bd	bd	bcd	bcd	bcd	acd	bc
CH03h03z_1	13	bd	c--	ac-	ac-	ad	bc	bd	bd	c--	ac-	c--	ad	ad	ac-	ac-	ac-	c--	ac
CH05c06_1	13	ac	c--	bc-	c--	bc	ad	ac	bd	bc-	c--	bc-	bc	bc	c--	c--	c--	bc-	bd
CH05f04	13	bc	c--	ac-	c--	bc	bd	ac	ad	ac-	c--	ac-	ac	ac	c--	c--	c--	ac-	ad
CH05h05	13	bd	bcd	acd	bcd	bd	bc	ad	ac	acd	bcd	acd	ad	ad	bcd	bcd	bcd	acd	ac
Hi03e04	13	bc	acd	acd	bcd	ac	bd	bc	bc	bcd	bcd	bcd	ac	bc	bcd	acd	bcd	acd	ad
Hi20b03	13	bd	bcd	acd	acd	bd	bc	ad	bd	acd	acd	---	bd	ad	acd	bcd	bcd	---	bd
NH009b	13	ac	acd	bcd	bcd	bc	ad	ac	ac	bcd	bcd	acd	ac	bc	bcd	bcd	bcd	acd	bd
AU223486	13	k-	hhk	hkk	hhk	hh	k-	k-	k-	hhk	hkk	hhk	hh	hh	hkk	hkk	hhk	hhk	hk
GD147	13	k-	hhk	hhk	hkk	hh	hk	hk	hh	hkk	hkk	hhk	hh	k-	hhk	hkk	hkk	hhk	k-
Hi05c06_2	13	hk	hkk	hhk	hhk	hk	hk	kk	kk	hhk	hkk	hhk	kk	h-	hhk	hkk	hhk	hkk	h-
Hi07b02_3	13	np	nnp	nnp	nnp	np	nn	nn	nn	nnp	nnp	nnp	nn	np	nnp	nnp	nnp	nnp	nn
NZ03c01x_2	13	np	nnp	nnp	nnp	np	nn	nn	nn	nnp	nnp	nnp	np	np	nnp	nnp	nnp	nnp	np
CH01g05	14	bcd	bd	acd	bcd	ad	ac	bc	bd	bd	acd	ad	bcd	ac	bd	acd	bd	acd	ad
CH03a02	14	acd	bc	acd	bcd	bd	ac	ad	bc	bd	acd	ac	bcd	ac	bc	acd	bc	acd	ac
CH03d08	14	acd	ac	bcd	acd	ad	bd	bc	ac	ac	bcd	bc	acd	bd	ac	bcd	ac	bcd	bc
CH05g07z_1	14	hhk	hh	hhk	hkk	k-	k-	k-	k-	hh	hhk	hk	hkk	hk	hh	hhk	hh	hkk	k-

Markers	LG	Aneuploid seedlings from the cross of 'Fuji × Gala'																	
		FG20	FG21	FG22	FG23	FG24	FG25	FG26	FG27	FG28	FG29	FG30	FG31	FG32	FG33	FG34	FG35	FG36	FG37
CH05g07z_2	14	hkk	kk	hkk	hhk	h-	h-	h-	h-	kk	hkk	hk	hhk	hk	kk	hkk	kk	hhk	h-
CH02c02a_1	15	bcd	acd	acd	bc	bc	bcd	ad	acd	bd	bcd	bcd	bd	bd	ac	acd	ac	bc	bcd
CH02d11	15	bcd	bcd	bcd	ac	bd	acd	ac	acd	bd	acd	acd	bd	ac	bc	bcd	bc	ac	bcd
CH03b10	15	acd	acd	acd	bc	ad	bcd	bc	bcd	ad	bcd	bcd	ad	bc	ac	acd	ac	bc	acd
Hi04c05	15	acd	acd	acd	bd	bd	bcd	ac	bcd	ac	bcd	bcd	ac	bc	ad	acd	ad	bd	acd
Hi06f09	15	ac-	bc-	bcd	ad	ad	acd	bc	acd	bc	acd	acd	ac	ad	bd	acd	bd	ad	acd
NZ02b01	15	acd	acd	acd	bd	bd	bcd	ac	bcd	ac	bcd	bcd	ac	bc	ad	acd	ad	bd	acd
Hi02g06	15	lll	lll	lll	lm	lm	llm	ll	llm	ll	llm	lll	ll	lm	ll	llm	ll	lm	llm
CH02c09	15	np1p2	np1p2	np1p2	np	np	np1p2	nn	np1p2	nn	np1p2	np1p2	nn	np	nn	np1p2	nn	nn	np1p2
CH02d10a	16	bc	ac	bd	acd	bcd	bcd	bd	bcd	bd	bc	bcd	bcd	bcd	bc	bcd	bc	acd	acd
CH04f10	16	bc	ac	bd	acd	bcd	acd	bc	bcd	ac	ad	acd	bcd	acd	bc	acd	bc	acd	bcd
CH05a04	16	bc	ad	ad	bcd	acd	bcd	ad	acd	bd	bc	bcd	acd	bcd	bd	bcd	bd	bcd	acd
CH05c06_2	16	bc	ac	ad	bcd	bcd	bcd	ad	acd	bd	bc	bcd	acd	bcd	bd	acd	bd	acd	acd
Hi01d06y	16	ac	bc	ad	bcd	acd	bcd	ac	acd	bc	bd	bcd	acd	bcd	ac	bcd	ac	bcd	acd
Hi04e04	16	ad	bd	ac	acd	bcd	acd	bc	bcd	ac	ad	acd	bd	acd	ac	acd	ac	bcd	bcd
CH05b06z_1	16	fg	eg	ee	eef	ee	eef	eef	efg	ef	fg	efg	efg	eef	ef	efg	ef	efg	efg
Hi01c11x	16	ee	ef	fg	eeg	eeg	eeg	fg	efg	eg	ee	efg	efg	eeg	eg	efg	eg	efg	eeg
CH01h01	17	acd	bcd	ac	ac	acd	bd	ac	acd	bcd	ad	bc	bcd	ad	bd	bcd	bcd	bcd	acd
CH04c06y_2	17	bc-	bc-	ac	ad	c--	bc	bc	c--	bc-	ac	bc	bc-	ad	bd	c--	bc-	bc-	c--
CH05d08y_1	17	ac-	ac-	bd	bc	c--	ad	ad	c--	ac-	bd	ad	ac-	bc	ac	c--	ac-	ac-	c--
CH05g03	17	bcd	acd	bc	bc	bcd	ad	bc	bcd	acd	bd	ac	acd	bd	ad	acd	acd	acd	bcd
Hi07b02_2	17	llm	lll	lm	ll	lll	lm	ll	lll	lll	ll	ll	lll	ll	ll	llm	lll	llm	llm
CH04c06y_3	17	np1p2	np1p2	nn	nn	np1p2	np	np	np1p2	np1p2	np	np	np1p2	nn	np	np1p2	np1p2	np1p2	np1p2
GD96	17	np1p2	np1p2	nn	nn	np1p2	np	nn	np1p2	np1p2	np	np	np1p2	nn	np	np1p2	np1p2	np1p2	np1p2
Hi03c05	17	nnp	nnp	nn	nn	nnp	np	nn	nnp	nnp	np	np	nnp	nn	np	nnp	nnp	nnp	nnp
Hi05c06_1	17	nnp	nnp	np	np	nnp	nn	nn	nnp	nnp	np	nn	nnp	np	nn	nnp	nnp	nnp	nnp
Hi07b02_1	17	nnp	nnp	np	np	nnp	nn	np	nnp	nnp	nn	nn	nnp	nn	nn	nnp	nnp	nnp	nnp

Note: '-' represents a null allele, or missing data; 'p1' and 'p2' are con-dominant alleles.
