# Supplementary material for: Molecular Genetic Features of Polyploidization and Aneuploidization Reveal Unique Patterns for Genome Duplication in Diploid Malus
Source: PLoS One. 2012 Jan 10;7(1):e29449. doi: 10.1371/journal.pone.0029449 (PMC3254611; doi:10.1371/journal.pone.0029449)
Supplement: Table S3 — The distributional features of microsatellite markers in the aneuploid seedlings from the cross of ‘Fuji×Pink Lady’. (PDF) [file pone.0029449.s004.pdf]

Markers	LG	Aneuploid seedlings from the cross of ‘Fuji × Pink Lady’																									
		FP01	FP02	FP03	FP04	FP05	FP06	FP07	FP08	FP09	FP10	FP11	FP12	FP13	FP14	FP15	FP16	FP17	FP18	FP19	FP20	FP21	FP22	FP23	FP24	FP25	FP26
CH05g08	1	bc	ad	ad	ad	ad	bc	bd	bc	ac	ad	ac	ad	bc	bc	bc	ad	ad	bc	ad	ad	bc	bd	bc	bd	bd	ac
Hi07d08	1	bd	ad	ac	bd	ac	ac	bd	bc	bc	bd	bc	bc	ac	bc	ac	bc	bd	bc	bd	bd	ac	ac	ac	ac	ac	ad
Hi12c02	1	bc	bc	ad	bc	bc	ad	ac	ad	bd	bc	bd	bc	ad	ad	ad	bc	bc	ad	bc	bc	ad	ac	ad	ac	ac	bd
KA4B	1	h-	h-	kk	h-	h-	kk	hk	kk	h-	h-	h-	h-	kk	kk	kk	h-	h-	kk	h-	h-	kk	h-	h-	hk	h-	kk
Hi02b10	1	lm	ll	ll	lm	ll	lm	lm	lm	ll	ll	ll	ll	lm	lm	lm	ll	ll	lm	ll	ll	lm	lm	ll	lm	lm	ll
Hi02c07	1	np	np	nn	np	np	nn	nn	nn	np	np	nn	np	nn	nn	nn	np	np	nn	np	np	nn	nn	nn	nn	nn	np
CH02a04z	2	ad	bd	a	ad	ac-	ac	ac	ac-	ad	ac-	bc-	bc-	bc	bc-	bc	ac-	bc	bd	bc-	bc-	bc-	ac-	ad	bc-	bd	bd
CH02c02a_3	2	b	ad	ac	bd	acd	ac	bd	bcd	bc	bcd	bcd	bcd	ac	bcd	ac	bcd	bd	bc	bcd	bcd	acd	acd	ac	acd	ac	ad
CH02c06	2	a	ad	bd	bd	bcd	bc	ac	acd	bc	bcd	bcd	acd	bd	bcd	bc	acd	bd	bc	bcd	bcd	acd	acd	bc	acd	ac	bd
CH03d01	2	a	ad	bd	ad	bcd	bc	bc	bcd	bd	bcd	acd	acd	bc	acd	ad	acd	ac	ad	acd	acd	acd	bcd	bd	acd	ad	ad
CH05e03	2	a	bc	bc	ac	bcd	bd	bd	bcd	bc	bcd	acd	acd	ad	acd	ad	bcd	ad	ac	acd	acd	acd	bcd	bc	acd	ac	ac
CN493139	2	a	ad	ad	bc	bcd	ad	ad	bcd	ac	acd	acd	acd	ad	bcd	ad	acd	bd	bc	acd	bcd	acd	acd	bc	acd	ad	bc
Hi02a07	2	h	kk	kk	h-	hkk	hk	h-	hhk	kk	hkk	hhk	hhk	hk	hkk	h-	hhk	h-	h-	hhk	hkk	hkk	hhk	kk	hkk	h-	h-
Hi05c06_3	2	m	lm	lm	lm	llm	ll	ll	lll	lm	lll	llm	lll	ll	lll	ll	lll	ll	lm	llm	llm	llm	lll	lm	lll	lm	lm
CH02c02a_2	2	n	nn	np	nn	nnp	np	np	nnp	np	nnp	nnp	nnp	np	nnp	np	nnp	nn	nn	nnp	nnp	nnp	nnp	nn	nnp	np	nn
CH03d10	2	n	nn	nn	nn	nnp	np	np	nnp	nn	nnp	nnp	nnp	np	nnp	np	nnp	np	nn	nnp	nnp	nnp	nnp	nn	nnp	nn	nn
CH03e03	3	ad	ad	bd	bc	bc	bc-	bc	bd	ac-	ad	ac	bc	bc	ac	bc-	bc-	bd	bc	bc	bc-	ad	bc-	bc-	ac	ac-	ac-
CH03g07	3	ad	bc	ad	ad	bc	bc-	bc	bd	ac-	ad	ac	bc	bc	ac	ac-	ac-	bd	bc	bc	bc-	ad	bc-	bc-	ac	ac-	ac-
HGA8bY	3	bd	bc	ad	ad	bc	bcd	bc	bd	bcd	ad	ac	ac	bc	ac	bcd	ad	bd	bc	bc	bcd	ad	acd	acd	bd	bcd	acd
Hi04c10x_1	3	bd	ac	bd	bd	ad	acd	bc	ad	acd	ad	bc	ac	bd	bc	bcd	bcd	ad	ac	bc	acd	bd	acd	acd	bd	bcd	acd
Hi07e08x	3	ac	ad	bc	bc	ad	acd	ad	ac	bcd	bc	bd	ad	ad	bd	acd	acd	ac	ad	ad	acd	bc	acd	acd	bd	bcd	bcd
AU223657	3	nn	np	nn	nn	np	np1p2	np	nn	np1p2	nn	np	np	nn	np	np1p2	np1p2	nn	np	np	np1p2	nn	np1p2	np1p2	np	np1p2	np1p2
CH02c02b	4	bc	ac	ac	bc	ac	acd	acd	acd	acd	acd	bcd	bcd	acd	bcd	bcd	ad	bd	bcd	bcd	bcd	acd	acd	acd	ad	bcd	bcd
CH05d02	4	bc	bd	ac	bd	bd	c--	ac-	ac-	ac-	ac-	c--	ac-	ac-	c--	c--	bc	bc	c--	c--	ac-	ac-	ac-	c--	bc	ac-	c--
GD162	4	bc	bd	ac	bd	bd	bcd	acd	acd	acd	acd	--	acd	acd	bcd	bcd	bc	bc	bcd	bcd	acd	acd	acd	bcd	bc	acd	bcd
Hi04c10x_3	4	ac	ad	bc	ad	ad	acd	bcd	bcd	bcd	bcd	acd	bcd	bcd	acd	acd	ac	ad	acd	acd	bcd	bcd	bcd	acd	ac	bcd	acd
Hi07b02_4	4	bc	ad	ac	bd	bd	bc-	ac-	ad	bc-	ac-	bc-	ad	ac-	bc-	bc-	bc	bd	bc-	bc-	bc-	ac-	ac-	bc-	bd	ac-	bc-
CH04e02	4	ef	ef	ee	fg	fg	efg	efg	eeg	eeg	eeg	eeg	eeg	eeg	eeg	efg	ef	ef	efg	eef	efg	eeg	efg	efg	ef	eeg	efg
CH03a04	5	ac	bd	bc	bd	bc	acd	bc	bcd	bc	ad	ad	bcd	bc	bd	bcd	bc	ac	bcd	bd	ad	bc	bcd	bcd	bcd	bcd	acd
CH03a09	5	ad	ac	bc	ac	ad	acd	ac	bcd	ac	bd	bd	bcd	ac	ad	bcd	ac	bc	bcd	bd	ad	ac	bcd	bcd	bcd	bcd	bcd
CH04e03	5	bc	ac	bc	ad	ac	bcd	bc	acd	ac	ad	ac	acd	bc	bd	bcd	ac	bc	acd	bd	ad	ac	bcd	ad	ad	bcd	bcd
CH04h02_2	5	ad	ac	bd	ac	ad	c--	ad	ac-	ad	bc	bc	ac-	ad	ac	ac-	ad	bd	ac-	bc	ac	ad	ac-	ac-	ac-	ac-	c--
CH05e06	5	ad	bc	ad	bc	bd	acd	ad	bcd	bd	bc	bc	bcd	ad	ac	bcd	bd	ad	bcd	bc	ac	bd	acd	acd	bcd	acd	bcd
Hi04d02	5	ac	bd	bd	ad	ac	acd	bd	bcd	ad	ac	ac	bcd	bd	bc	bcd	ad	bd	bcd	bc	ac	ad	acd	acd	ac	acd	bcd

Markers	LG	Aneuploid seedlings from the cross of 'Fuji × Pink Lady'																									
		FP01	FP02	FP03	FP04	FP05	FP06	FP07	FP08	FP09	FP10	FP11	FP12	FP13	FP14	FP15	FP16	FP17	FP18	FP19	FP20	FP21	FP22	FP23	FP24	FP25	FP26
Hi11a03	5	bc	ad	bc	ad	ac	c--	bc	ac-	ac	ad	ad	ac-	bc	bd	ac-	ac	bc	ac-	bd	ad	ac	c--	c--	ad	c--	ac-
CH04g09y	5	ef	fg	ee	fg	ef	eeg	ef	efg	ef	fg	eg	efg	ee	fg	eeg	ee	eg	efg	eg	fg	ef	eeg	efg	efg	eeg	eeg
Hi21c08	5	hk	hk	hk	hk	hk	hhk	hk	hkk	h-	hk	h-	hhk	hk	hk	hkk	hk	h-	hhk	kk	kk	h-	hkk	hhk	hhk	hkk	hkk
CH02a08z	5	ll	lm	ll	lm	ll	lll	ll	llm	ll	lm	lm	lll	ll	lm	llm	lm	ll	llm	lm	lm	ll	llm	llm	llm	llm	lll
CH04h02_4	5	lm	ll	lm	ll	lm	llm	lm	lll	lm	ll	ll	llm	lm	ll	lll	ll	lm	lll	ll	ll	lm	lll	lll	lll	lll	llm
AJ000761	6	bc	bc	ad	bd	bc	ac	ad	c--	c--	bc	bc	ad	ad	ad	bc	ad	bc-	ad	bd	bd	ac	c--	bc-	bd	c--	ad
CH03c01	6	bc	bd	bd	bd	ac	ad	ac	acd	acd	bd	bd	ac	ac	ac	bd	ac	bcd	ac	bc	bc	ac	acd	bcd	bc	acd	ac
CH03d07	6	ad	bd	ac	ac	ac	bc	bc	bcd	bcd	ac	ac	bd	bd	bd	ac	bc	acd	bd	ad	ad	bc	bcd	acd	ad	bcd	bd
CH03d12	6	bc	ac	ad	ad	ad	bd	bd	bcd	bcd	ad	ad	bc	bc	bc	ad	bd	acd	bc	ac	ac	bd	bcd	acd	ac	bcd	bc
Hi01d05	6	ef	ee	fg	eg	eg	fg	eg	efg	efg	eg	eg	ef	ef	ef	eg	fg	eeg	ef	ee	ee	fg	efg	eeg	ee	efg	ef
CH05b06z_2	7	bc	bd	bd	ac	ac	bd	bc	bd	ac	bc	bc	bc	bc	bc	bc	bcd	bcd	bc	bc	bc	ac	bc	bc	ac	ac	ac
Hi04c10x_2	7	ac	bd	ad	ac	bd	ac	ad	ac	bd	ad	ad	ad	ad	ad	ad	acd	acd	ad	ad	ad	bd	ad	ad	bd	bd	bd
CH04e05	7	nn	np	np	nn	nn	np	nn	np	nn	nn	nn	nn	nn	nn	nn	np1p2	np1p2	nn	nn	nn	nn	nn	nn	nn	nn	nn
Hi05b09	7	nn	np	np	nn	np	nn	np	nn	np	np	np	np	np	np	np	np1p2	np1p2	np	np	np	np	np	np	np	np	np
CH01c06	8	ad	bd	ac	bc	ad	ac	bc	ad	bc	bc	bd	bd	bc	bc	bd	ad	ac	bc	bd	ad	ad	ad	ac	bd	bc	ad
Hi04b12	8	ac	bc	bd	bd	ad	bc	ac	bd	ac	ac	ad	ad	ac	ac	ad	bd	bc	ac	bd	ad	bd	bd	bc	ad	ac	bd
Hi23g12	8	bd	ad	bc	ac	bc	bd	ad	bc	ad	ad	ac	ac	ad	ad	ac	bc	bd	ad	bc	ac	bc	bc	bd	ac	ad	bc
CH02g09	8	np	np	nn	nn	np	nn	np	np	nn	nn	np	np	nn	nn	np	np	nn	nn	np	np	nn	np	nn	np	nn	np
Hi04e05	8	nn	nn	np	np	np	nn	nn	np	nn	nn	np	np	nn	nn	np	nn	nn	nn	np	nn	np	np	nn	np	nn	np
CH01h02_2	9	ac	bd	acd	bd	bd	bcd	bcd	ad	bd	bcd	acd	bcd	acd	bcd	bd	bcd	bcd	bcd	acd	bcd	acd	bc	bcd	acd	acd	acd
CH05c07	9	bd	ad	acd	bd	ad	bcd	acd	bc	ad	acd	bcd	bcd	acd	bcd	ad	bcd	acd	bcd	acd	bcd	bcd	bc	bcd	bcd	acd	acd
GD142	9	ac	bc	bc-	bc	bc	bc-	bc-	ac	bc	bc-	ac-	bc-	bc-	bc-	bc	bc-	ac-	ac-	ac-	ac-	ac-	ad	bc-	ac-	ac-	ac-
Hi05e07	9	ad	bc	bcd	ad	ad	acd	acd	bd	ad	acd	bcd	acd	bcd	acd	ad	acd	acd	acd	bcd	acd	bcd	ac	acd	bcd	bcd	bcd
NH029a	9	bd	ac	bcd	ac	ac	acd	acd	bc	ac	acd	bcd	acd	bcd	acd	ac	acd	acd	acd	bcd	acd	bcd	ad	acd	bcd	bcd	bcd
CH01h02_1	9	k-	hk	hkk	hk	hk	hhk	hkk	hh	hk	hkk	hkk	hhk	hkk	hhk	hk	hhk	hkk	hhk	hkk	hhk	hkk	k-	hhk	hkk	hkk	hhk
CH05d08y_2	9	ll	lm	lll	lm	lm	llm	llm	ll	lm	llm	lll	llm	lll	llm	lm	llm	llm	llm	lll	llm	lll	lm	llm	lll	lll	lll
Hi01d01	9	nn	np	np1p2	np	np	np1p2	np1p2	nn	np	np1p2	np1p2	np1p2	np1p2	np1p2	np	np1p2	np1p2	np1p2	np1p2	np1p2	np1p2	np	np1p2	np1p2	np1p2	np1p2
CH01f07a	10	ad	ac	ad	ad	acd	bcd	bcd	ad	bd	acd	acd	ac	bcd	acd	ad	acd	acd	acd	bcd	bcd	acd	acd	bd	bcd	acd	acd
CH01f12	10	ac	bc	bd	bc	bc-	bc-	bc-	ad	bc	ac-	ac-	ac	bc-	ac-	bd	bc-	bc-	ac-	bc-	bc-	ac-	bc-	bd	bc-	ac-	ac-
CH02b03b	10	bc	bc	bc	bd	bcd	acd	ad	bc	ac	bcd	bcd	bd	acd	bcd	bc	bcd	bcd	bcd	acd	acd	bcd	bcd	ac	acd	bcd	bcd
CH02b07	10	bc	ac	ad	ac	acd	acd	ad	bd	ac	bcd	bcd	bc	acd	bcd	ad	acd	acd	bcd	acd	acd	bcd	acd	ad	bcd	bcd	bcd
CH02c11	10	bc	bc	bc	bd	bcd	acd	ad	bc	ac	bcd	bcd	bd	acd	bcd	bc	bcd	bcd	bcd	acd	acd	bcd	bcd	ac	acd	bcd	bcd
MS02a01	10	bd	ad	ac	ad	ac-	ac-	c--	bc	ad	c--	c--	ad	ac-	c--	ac	ac-	ac-	c--	ac-	ac-	c--	ac-	ac	c--	c--	c--
MS06g03	10	bc	bc	bd	bd	bcd	acd	acd	bc	ac	bcd	bcd	bd	acd	bcd	bc	bcd	bcd	bcd	acd	acd	bcd	bcd	ac	acd	acd	bcd

Markers	LG	Aneuploid seedlings from the cross of ‘Fuji × Pink Lady’																									
		FP01	FP02	FP03	FP04	FP05	FP06	FP07	FP08	FP09	FP10	FP11	FP12	FP13	FP14	FP15	FP16	FP17	FP18	FP19	FP20	FP21	FP22	FP23	FP24	FP25	FP26
Hi04f08	10	ll	ll	lm	ll	lll	lll	llm	lm	ll	llm	lll	ll	llm	lll	lm	lll	lll	lll	lll	llm	llm	llm	lm	lll	lm	lll
CH02a10	10	nn	nn	np	nn	np1p2	np1p2	np1p2	np	nn	np1p2	np1p2	nn	np1p2	np1p2	np	np1p2	np1p2	np1p2	np1p2	np1p2	np1p2	np1p2	np	np1p2	np1p2	np1p2
CH03d11	10	np	nn	nn	np	np1p2	np1p2	np1p2	nn	nn	np1p2	np1p2	np	np1p2	np1p2	nn	np1p2	np1p2	np1p2	np1p2	np1p2	np1p2	np1p2	nn	np1p2	np1p2	np1p2
CH04c06y_1	10	nn	np	np	np	nnp	nnp	nnp	nn	np	nnp	nnp	nn	nnp	nnp	np	nnp	nnp	nnp	nnp	nnp	nnp	nnp	np	nnp	nnp	nnp
Hi02d04	10	nn	np	np	nn	np1p2	np1p2	np1p2	np	np	np1p2	np1p2	nn	np1p2	np1p2	np	np1p2	np1p2	np1p2	np1p2	np1p2	np1p2	np1p2	nn	np1p2	np1p2	np1p2
CH02d08	11	ac	ac	ad	ad	bd	bc	ac	bc	bcd	bcd	bd	ad	ac	bcd	acd	ac	bc	ad	bc	bd	bd	bc	bd	acd	ad	acd
CH04g07	11	ac	ac	bd	bc	ac	bc	ac	ac	ac-	ac-	bc	bc	ac	ac-	bc-	ac	ac	ad	ad	bd	ad	ad	ad	bc-	bd	ac-
CH04h02_1	11	bd	bd	ac	bc	ac	ad	bd	ad	ac-	c--	ac	bc	bd	c--	c--	bd	ad	bc	ad	bc	ac	ad	ac	c--	bc	c--
Hi06b06	11	ad	ad	bd	bd	ad	bd	ad	ad	acd	acd	bd	bd	ad	acd	bcd	ad	ad	ac	ac	bc	ac	ac	ac	bcd	bc	acd
CH04h02_3	11	ll	ll	ll	ll	lm	lm	ll	lm	llm	lll	lm	ll	ll	llm	llm	ll	lm	ll	lm	lm	lm	lm	lm	llm	lm	lll
CH01b12y	12	bd	bd	ad	bc	ac-	bd	ad	bc	ad	bc-	ac-	ac	bc	ad	ac-	bd	ac-	bc-	ac-	ac	ac-	bc-	bc	ac-	ac-	ac-
CH01f02	12	ac	ad	bd	bc	ac-	bc	ac	bd	ac	ac-	bc-	ad	ad	ac	ac-	bc	ac-	bc-	ac-	ad	ac-	bc-	bd	bc-	ac-	ac-
CH01g12	12	bc	ad	ac	ac	bcd	ac	bc	ad	bc	acd	bcd	bd	ad	bc	bcd	ac	bcd	acd	bcd	bc	bcd	acd	ad	bcd	bcd	bcd
NZ28f04	12	bd	bc	ac	ad	bc-	ad	bd	ac	bd	bc-	ac-	bc	bc	bd	bc-	ad	bc-	ac-	bc-	bc	bc-	ac-	ac	bc-	bc-	bc-
CH05d04	12	ef	fg	eg	ee	eef	fg	eg	ef	eg	efg	eef	ee	ee	ef	efg	fg	efg	eef	efg	ee	efg	eef	ef	eef	efg	efg
CH05d11	12	ef	fg	eg	ee	eeg	ee	ef	eg	ef	efg	eeg	fg	fg	eg	efg	ee	efg	eeg	efg	fg	efg	eeg	eg	eeg	efg	efg
CH03h03z_2	12	lm	lm	ll	ll	lll	ll	lm	ll	lm	llm	lll	lm	ll	lm	llm	lm	llm	lll	llm	ll	lll	llm	ll	lll	llm	lll
CH03a08	13	bd	ad	ac	ad	bc	ad	ad	ad	bd	bcd	bc	acd	acd	bd	acd	bc	bd	bcd	bcd	bd	acd	ad	bc	bd	ad	acd
CH03h03z_1	13	ad	ac	bc	bc	bd	ac	ac	ac	bc	bc-	bd	c--	c--	bc	c--	bd	bc	bc-	bc-	bc	c--	ac	bd	bc	ac	c--
CH05f04	13	ac	bc	bd	bc	ad	bc	bc	ac	ac	bcd	bd	acd	acd	ac	acd	ad	ac	bcd	bcd	ad	bcd	bc	ad	ac	bc	bcd
CH05h05	13	ac	bc	bc	bd	ac	bd	bd	ad	bc	bcd	bc	acd	acd	ad	acd	ac	ad	bcd	bcd	ac	bcd	ad	bd	bd	bd	bcd
Hi05c06_2	13	bd	bd	bc	ad	bc	bd	bd	bd	bd	bc-	ac	ac-	bc-	ac	ac-	bc	ad	ac-	bc-	ad	bc-	bd	bc	bd	ad	ac-
Hi20b03	13	bc	ad	ad	ad	ac	bd	ad	bd	ad	acd	ac	bcd	bc	ad	bcd	ac	bd	acd	acd	bc	bcd	bd	ac	ad	bd	bcd
NH009b	13	bc	bc	ad	bc	bd	ac	bc	ac	bc	bcd	bd	acd	ad	bc	acd	bd	ac	bcd	bcd	ad	acd	ac	bd	bc	ac	acd
GD147	13	h-	h-	hk	h-	h-	kk	h-	kk	h-	hhk	h-	hhk	hkk	h-	hhk	h-	kk	hhk	hkk	hk	hkk	kk	h-	hk	kk	hhk
Hi03e04	13	kk	kk	h-	kk	hk	h-	h-	h-	kk	hkk	hk	hkk	hhk	kk	hkk	hk	kk	hkk	hhk	hk	hhk	h-	hk	kk	h-	hkk
AU223486	13	ll	ll	lm	ll	ll	ll	ll	lm	ll	lll	ll	llm	llm	lm	llm	lm	lm	lll	lll	lm	llm	lm	ll	ll	ll	lll
CH05c06_1	13	lm	lm	ll	lm	ll	ll	lm	ll	lm	llm	lm	lll	lll	ll	lll	lm	ll	llm	llm	ll	lll	ll	lm	lm	ll	llm
Hi07b02_3	13	np	np	nn	np	np	nn	np	nn	np	nnp	np	nnp	nnp	np	nnp	np	nn	nnp	nnp	nn	nnp	nn	np	np	np	nnp
NZ03c01x_2	13	nn	np	np	nn	np	nn	nn	nn	nn	nnp	nn	nnp	nnp	np	nnp	np	np	nnp	nnp	np	nnp	nn	np	np	nn	nnp
CH01g05	14	bc	bd	ac	acd	bc	ad	ad	bd	acd	bd	acd	acd	ac	acd	bd	ad	bc	bcd	bd	acd	ac	bcd	acd	bcd	ac	bd
CH03a02	14	ad	bc	bd	bcd	bc	ac	ac	bc	acd	bc	acd	acd	bc	acd	bc	bc	bd	bcd	bc	acd	ad	acd	acd	bcd	ac	bc
CH03d08	14	bd	ac	ad	bcd	ad	bc	bc	ac	bcd	ac	bcd	bcd	bd	bcd	ac	bc	ad	acd	ac	bcd	bd	acd	bcd	acd	bd	ac
CH05g07z_1	14	k-	hk-	hk	hkk	k-	hk	hk	hh	hhk	hh	hkk	hhk	k-	hkk	hh	hk	hk	hkk	hh	hhk	k-	hkk	hhk	hkk	hk	hh

Markers	LG	Aneuploid seedlings from the cross of ‘Fuji × Pink Lady’																									
		FP01	FP02	FP03	FP04	FP05	FP06	FP07	FP08	FP09	FP10	FP11	FP12	FP13	FP14	FP15	FP16	FP17	FP18	FP19	FP20	FP21	FP22	FP23	FP24	FP25	FP26
CH05g07z_2	14	hk	h-	h-	hhk	h-	hk	hk	kk	hkk	kk	hhk	hkk	h-	hhk	kk	hk	hk	hhk	kk	hkk	h-	hhk	hkk	hhk	hk	kk
CH02c02a_1	15	ac	ad	ac	ad	ad	bd	ac-	c--	ac	bc	ac	ad	c--	bd	ac	c--	c--	ac-	ac-	bd	ac-	bc	ac-	c--	ac-	ac-
CH02d11	15	ac	bd	bc	bd	bc	ad	bcd	acd	bc	bc	ac	bd	acd	ac	bc	acd	bcd	acd	bcd	ad	acd	ad	acd	acd	bcd	bcd
CH03b10	15	bd	bc	ad	bc	bd	ac	bcd	acd	bd	bd	ad	bc	acd	ad	bd	acd	bcd	acd	bcd	ac	acd	ac	acd	acd	bcd	bcd
Hi04c05	15	ad	ac	bc	ad	ad	bd	ac-	c--	ac	ac	bc	ad	c--	bd	ac	c--	ac-	c--	ac-	ac	c--	ac	c--	c--	ac-	ac-
NZ02b01	15	ad	ac	bd	ac	ac	bc	acd	bcd	ad	ad	bd	ac	bcd	bc	ad	bcd	acd	bcd	acd	bc	bcd	bc	bcd	bcd	acd	acd
Hi02g06	15	ll	ll	ll	ll	lm	lm	llm	lll	lm	lm	ll	ll	llm	ll	ll	lll	llm	lll	llm	lm	lll	ll	lll	lll	lll	lll
Hi06f09	15	lm	lm	lm	ll	ll	lm	lll	lll	ll	ll	lm	ll	llm	ll	lm	lll	lll	llm	llm	ll	llm	lm	llm	lll	llm	llm
CH02c09	15	nn	np	np	nn	nn	nn	np1p2	np1p2	nn	nn	np	nn	np1p2	np	nn	np1p2	np1p2	np1p2	np1p2	np	np1p2	np	np1p2	np1p2	np1p2	np1p2
CH02d10a	16	bc	acd	ac	ac	bd	acd	bcd	ad	bc	ac	bd	bcd	acd	bc	bcd	acd	bcd	ad	bcd	bcd	bcd	ac	acd	bcd	bcd	bcd
CH05a04	16	ad	acd	ac	ac	ac	acd	bcd	ad	bd	ad	bd	acd	bcd	bd	bcd	acd	acd	ac	bcd	acd	bcd	bd	bcd	acd	acd	acd
CH05b06z_1	16	bd	bcd	ad	bd	bd	bcd	bcd	bc	ac	bd	ac	bcd	acd	ac	acd	bcd	bcd	bd	bcd	bcd	acd	bc	acd	bcd	bcd	acd
CH05c06_2	16	ad	acd	ac	ad	ac	acd	bcd	bd	ad	ad	bc	acd	bcd	bd	bcd	bcd	acd	ac	bcd	acd	bcd	bd	bcd	bcd	bcd	acd
Hi01c11x	16	fg	efg	ef	fg	ef	efg	eeg	eg	ef	fg	ee	efg	eeg	eg	eeg	eeg	efg	ee	eeg	efg	efg	eg	eeg	eeg	eeg	efg
Hi01d06y	16	eg	eeg	ee	eg	ee	eg	efg	fg	ee	eg	ef	eeg	efg	fg	efg	efg	eeg	ef	efg	eeg	eeg	fg	efg	efg	eeg	eeg
Hi04e04	16	fg	efg	ef	fg	ef	efg	eeg	eg	ef	fg	ee	efg	eeg	eg	eeg	eeg	efg	ee	eeg	efg	efg	eg	eeg	eeg	eeg	efg
CH04f10	16	lm	lll	ll	lm	lm	llm	llm	lm	ll	lm	ll	llm	lll	ll	lll	llm	llm	lm	llm	llm	lll	lm	lll	llm	llm	lll
CH01h01	17	ac	bc	bd	ac	bc	ad	bcd	acd	bcd	bd	acd	ac	bcd	acd	bcd	acd	bcd	ad	bd	acd	acd	acd	acd	acd	bcd	bcd
CH05d08y_1	17	bd	bd	ad	bd	ad	ac	c--	c--	c--	bc	c--	ad	c--	c--	c--	ac-	ac-	ac	ac	ac-	ac-	c--	c--	c--	ac-	ac-
CH04c06y_2	17	lm	ll	ll	lm	lm	lm	lll	lll	lll	ll	lll	lm	lll	llm	lll	llm	llm	lm	ll	llm	llm	llm	llm	llm	lll	lll
CH05g03	17	ll	lm	lm	ll	ll	lm	lll	lll	lll	ll	lll	lm	lll	lll	lll	llm	llm	lm	lm	lm	lm	lll	lll	lll	llm	llm
Hi07b02_2	17	lm	lm	lm	ll	lm	ll	lll	lll	llm	ll	lll	ll	lll	lll	lll	lll	lll	lm	ll	llm	lll	lll	llm	lll	lll	lll
CH04c06y_3	17	nn	np	np	nn	np	nn	nnp	nnp	nnp	np	nnp	np	nnp	nnp	nnp	nnp	nnp	nn	np	nnp	nnp	nnp	nnp	nnp	nnp	nnp
GD96	17	nn	nn	nn	nn	nn	np	nnp	nnp	nnp	np	nnp	nn	nnp	nnp	nnp	nnp	nnp	np	np	nnp	nnp	nnp	nnp	nnp	nnp	nnp
Hi03c05	17	nn	nn	nn	np	np	np	np1p2	np1p2	np1p2	nn	nn	np	np1p2	np1p2	np1p2	np1p2	np1p2	np	np	np1p2	np1p2	np1p2	np1p2	np1p2	np1p2	np1p2
Hi05c06_1	17	np	nn	nn	np	np	nn	nnp	nnp	nnp	np	np	nn	nnp	nnp	nnp	nnp	nnp	nn	nn	nnp	nnp	nnp	nnp	nnp	nnp	nnp
Hi07b02_1	17	np	np	nn	np	nn	np	np1p2	np1p2	np1p2	np	nn	np	np1p2	np1p2	np1p2	np1p2	np1p2	np	nn	np1p2	np1p2	np1p2	np1p2	np1p2	np1p2	np1p2

Note: ‘-‘ represents a null allele, or missing data; ‘p1’ and ‘p2’ are con-dominant alleles.
