# Supplementary material for: Molecular Genetic Features of Polyploidization and Aneuploidization Reveal Unique Patterns for Genome Duplication in Diploid Malus
Source: PLoS One. 2012 Jan 10;7(1):e29449. doi: 10.1371/journal.pone.0029449 (PMC3254611; doi:10.1371/journal.pone.0029449)
Supplement: Table S4 — The distributional features of microsatellite markers in the aneuploid seedlings from the cross of ‘Pink Lady×Fuji’. (PDF) [file pone.0029449.s005.pdf]

Markers	LG	Aneuploid seedlings from the cross of 'Pink Lady × Fuji'																							
		PF01	PF02	PF03	PF04	PF05	PF06	PF07	PF08	PF09	PF10	PF11	PF12	PF13	PF14	PF15	PF16	PF17	PF18	PF19	PF20	PF21	PF22	PF23	PF24
CH05g08	1	ad	bd	ad	bd	bc	bc	ad	bc	bc	ad	bd	ad	ad	bd	bd	bc	ad	bc	ac	ad	bd	bd	bc	ad
Hi07d08	1	bd	bd	bd	ad	ad	bc	bd	bc	ac	ad	ad	bd	ad	bc	bc	ad	bc	ac	bc	bc	bc	ad	ac	bc
Hi12c02	1	bc	ac	bc	ac	ad	ad	bc	ad	ad	bc	ac	bc	bc	ac	ac	ad	bc	ad	bd	bc	ac	ac	ad	bc
KA4B	1	h-	hk	h-	hk	kk	h-	h-	kk	kk	h-	hk	h-	h-	h-	hk	kk	h-	kk	h-	h-	h-	hk	h-	hk
Hi02c07	1	lm	ll	lm	ll	ll	ll	lm	ll	ll	lm	ll	lm	lm	ll	lm	ll	lm	ll	lm	lm	ll	ll	ll	lm
Hi02b10	1	np	nn	np	np	nn	nn	np	nn	nn	np	np	nn	np	np	np	nn	np	nn	nn	np	np	np	nn	np
CH02a04z	2	ad	ad	bc	ac	ac	bcd	ad	bd	ac	acd	bd	acd	bcd	acd	ac	acd	bcd	acd	bc	bcd	ad	bc	ad	acd
CH02c02a_3	2	bd	bd	bd	ad	ad	bc-	bd	bc	ac	ac-	ad	bc-	ac-	bc-	bc	ac-	bc-	ac-	bc	bc-	bc	ad	ac	bc-
CH02c06	2	ac	ac	bc	ad	bc	bcd	bd	bd	ac	bcd	bd	acd	acd	bcd	bc	acd	acd	bcd	ac	bcd	ac	ac	ac	acd
CH03d01	2	bc	bc	bd	bd	bc	ac-	bd	ad	bc	ac-	ad	bc-	ac-	bc-	bc	bc-	ac-	bc-	ac	ac-	bd	ac	bd	bc-
CH05e03	2	ad	bd	bc	bc	bd	acd	bc	ac	bd	bcd	ac	bcd	acd	bcd	bd	bcd	acd	bcd	ad	acd	bc	ad	bc	bcd
CN493139	2	ad	ad	ad	bd	ac	bcd	ac	bc	ad	acd	bc	acd	bcd	acd	bd	acd	bcd	bcd	bd	bcd	ad	bc	bc	bcd
Hi02a07	2	h-	h-	kk	kk	hk	hhk	kk	h-	hk	hkk	h-	hhk	hkk	hhk	hk	hkk	hhk	hkk	h-	hhk	kk	h-	kk	hkk
CH02c02a_2	2	ll	ll	ll	lm	ll	llm	ll	lm	ll	lll	lm	llm	llm	lll	ll	lll	llm	llm	lm	llm	ll	lm	lm	llm
CH03d10	2	ll	ll	lm	lm	ll	llm	lm	lm	ll	llm	lm	llm	lll	lll	ll	llm	lll	lll	ll	llm	lm	ll	lm	llm
Hi05c06_3	2	np	np	nn	np	np	nnp	nn	nn	np	nnp	nn	nnp	nnp	nnp	np	nnp	nnp	nnp	np	nnp	nn	np	nn	nnp
CH03e03	3	bd	bc	bc	bc	bd	ad	ac	bcd	acd	ac	bcd	bcd	bd	acd	bd	bd	acd	acd	bcd	bd	acd	bd	acd	bcd
CH03g07	3	bc	bc	bd	bc	bd	ad	ac	acd	acd	ac	bcd	bcd	bd	acd	bd	bd	bcd	acd	acd	bd	acd	ad	acd	bcd
HGA8bY	3	ac	ad	ad	bc	ad	ad	ac	bc-	bc-	ac	bc-	bc-	ad	ac-	bd	bd	bc-	bc-	ac-	ac	bc-	bc	bc-	ac-
Hi04c10x_1	3	ac	bc	ad	ad	ac	bd	ac	bc-	ac-	bc	bc-	ac-	ad	bc-	ad	ac	ac-	bc-	bc-	bd	bc-	bc	ac-	ac-
Hi07e08x	3	ad	ad	ac	ad	ac	bc	bd	ac-	bc-	bd	ac-	bc-	ac	bc-	ac	ac	bc-	bc-	ac-	ac	bc-	ac	bc-	ac-
AU223657	3	lm	ll	ll	ll	ll	lm	lm	lll	llm	ll	lll	llm	lm	llm	ll	ll	llm	llm	llm	ll	lll	lm	llm	llm
CH02c02b	4	bd	ad	ac	ad	ad	bcd	acd	bcd	acd	ac	bc	acd	bd	acd	acd	acd	bc	acd	bcd	bc	acd	bcd	acd	acd
CH05d02	4	ac	ad	bc	ac	bc	c--	c--	c--	c--	bc	ad	c--	--	c--	ac-	ac-	bd	ac-	c--	bd	c--	ac-	c--	ac-
GD162	4	ac	ad	bc	ac	bc	bcd	bcd	bcd	bcd	bc	ad	bcd	bd	bcd	acd	--	bd	acd	bcd	bd	bcd	acd	bcd	acd
Hi04c10x_3	4	bc	bd	ac	bc	ac	ac-	ac-	ac-	ac-	ad	bd	ac-	bd	ac-	bc-	bc-	ad	bc-	ac-	ad	ac-	bc-	bc-	bc-
Hi07b02_4	4	ac	ad	bc	ac	bc	bcd	bcd	bcd	bcd	bd	bd	acd	ad	bcd	bcd	acd	ad	bcd	bcd	bd	bcd	acd	acd	acd
CH04e02	4	ee	fg	fg	ee	fg	eeg	efg	efg	efg	ef	eg	efg	ef	efg	eeg	eeg	fg	eeg	efg	fg	efg	efg	efg	eeg
CH03a04	5	bd	ad	bc	bc	ac	bcd	ad	acd	ac	acd	acd	bc	bcd	acd	acd	ad	bcd	bd	bcd	bcd	bcd	acd	acd	acd
CH03a09	5	ad	ad	ac	bd	bc	acd	bd	acd	bc	bcd	bcd	ac	acd	bcd	bcd	bd	acd	ad	acd	acd	acd	bcd	bcd	acd
CH04e03	5	ac	bc	ad	ad	bc	bcd	ac	bcd	bc	bcd	bcd	bc	acd	bcd	bcd	bc	acd	ad	acd	acd	acd	acd	bcd	bcd
CH04h02_2	5	ad	ad	ac	bc	bd	ac-	bc	c--	bd	c--	c--	ad	ac-	c--	c--	bc	ac-	ac	ac-	ac-	ac-	c--	c--	ac-
CH05e06	5	bc	bc	bd	ad	ac	acd	bc	acd	bd	acd	acd	ad	bcd	acd	acd	ad	bcd	bc	bcd	bcd	bcd	acd	acd	acd
Hi04d02	5	ac	ac	ad	ac	bc	bcd	ac	bcd	ad	bcd	bcd	bd	acd	bcd	bcd	bd	acd	ac	acd	acd	acd	bcd	bcd	bcd

Markers	LG	Aneuploid seedlings from the cross of ‘Pink Lady × Fuji’																							
		PF01	PF02	PF03	PF04	PF05	PF06	PF07	PF08	PF09	PF10	PF11	PF12	PF13	PF14	PF15	PF16	PF17	PF18	PF19	PF20	PF21	PF22	PF23	PF24
Hi11a03	5	ac	bc	ad	ad	bd	c--	ad	c--	ac	c--	c--	bc	ac-	c--	c--	bc	ac-	ad	ac-	ac-	ac-	c--	c--	c--
CH04g09y	5	ef	ef	fg	fg	ee	efg	fg	eeg	ee	eeg	eeg	ef	efg	eeg	eeg	ee	efg	fg	efg	efg	efg	eeg	eeg	efg
Hi21c08	5	hk	hk	h-	hk	kk	hkk	hk	hhk	h-	hkk	hhk	hk	hkk	hhk	hkk	hk	hhk	h-	hkk	hhk	hkk	hhk	hkk	hhk
CH02a08z	5	nn	nn	np	np	nn	np1p2	nn	np1p2	np	np1p2	np1p2	np	np1p2	np1p2	np1p2	np	np1p2	nn	np1p2	np1p2	np1p2	np1p2	np1p2	np1p2
CH04h02_4	5	nn	nn	np	np	np	nnp	np	nnp	nn	nnp	nnp	nn	nnp	nnp	nnp	nn	nn	np	nnp	nnp	nnp	nnp	nnp	nnp
AJ000761	6	bc	ac	ac	ad	ad	bd	ac	c--	bc-	ac	bc-	bc-	bc-	c--	c--	bc	bc-	c--	bc-	bc	c--	bc-	ad	bc
CH03c01	6	ad	ac	bd	ad	ac	bc	ac	ac-	bc-	ad	bc-	bc-	bc-	ac-	ac-	bd	bc-	ac-	bc-	bd	ac-	bc-	ac	bd
CH03d07	6	ad	ad	ad	bc	bd	ad	bc	acd	acd	ad	acd	acd	acd	acd	acd	ac	acd	acd	acd	ac	acd	acd	bd	ac
CH03d12	6	bd	bd	ad	bd	bc	ac	bd	bc-	ac-	bd	ac-	ac-	ac-	bc-	bc-	ad	ac-	bc-	ac-	ad	bc-	ac-	bc	ad
Hi01d05	6	fg	eg	eg	fg	ef	ee	fg	efg	eeg	fg	eeg	efg	eeg	efg	efg	eg	eeg	efg	eeg	eg	efg	eeg	ef	eg
CH05b06z_2	7	ac	bc	bc	bc	bc	ac	ad	ac	bd	bc	ad	bc	ad	acd	bc	bc	ac	ac	ad	bd	ac	ac	bd	ad
Hi04c10x_2	7	ad	ad	bd	ad	ad	bd	bc	bd	ac	ad	bc	ad	bc	bc-	ad	ad	bd	bd	bc	ac	bd	bd	ac	bc
CH04e05	7	lm	ll	ll	lm	lm	lm	ll	lm	ll	lm	ll	lm	ll	llm	lm	ll	lm	lm	ll	ll	lm	lm	ll	ll
Hi05b09	7	lm	lm	ll	ll	ll	ll	lm	ll	lm	ll	lm	ll	lm	lll	ll	ll	ll	lm	lm	lm	ll	ll	lm	lm
CH01c06	8	bc	bd	ad	bc	ac	ad	bc	ad	bc	bc	bd	bc	ac	bd	bd	bc	bd	ac	ad	ac	bc	bd	ad	ad
Hi04b12	8	ac	ad	ac	ad	bc	bd	ac	bd	ac	ac	ad	ac	bc	ad	ad	ac	ad	bc	bd	bc	ac	ad	bd	bd
Hi23g12	8	bc	ad	ad	ac	bd	bc	ad	bc	ad	ad	ac	ad	bd	ac	ac	ad	ac	bd	bc	bd	ad	ac	bc	bc
CH02g09	8	lm	lm	ll	lm	lm	ll	lm	ll	lm	lm	lm	lm	lm	ll	ll	lm	ll	ll	ll	lm	lm	ll	ll	ll
Hi04e05	8	ll	ll	ll	ll	lm	ll	lm	ll	lm	lm	ll	lm	lm	ll	lm	lm	ll	lm	ll	lm	ll	ll	ll	ll
CH01h02_2	9	bc	ad	ac-	ac-	ac-	ac-	ac-	ac	ac-	bc-	ac	bd	bc-	ac-	ac-	ac-	ac	bc-	ac-	ac-	bc	ac-	ac-	bd
CH05c07	9	ad	ac	acd	acd	bcd	acd	acd	bc	acd	acd	ac	ad	acd	bcd	acd	acd	bd	acd	acd	acd	bc	bcd	acd	bd
GD142	9	ac	bd	acd	acd	acd	acd	bcd	ad	acd	bcd	bd	bd	bcd	acd	bcd	bcd	ac	bcd	acd	bcd	bc	acd	acd	bc
Hi05e07	9	ac	ad	ac-	ac-	ac-	ac-	ac-	bd	ac-	ac-	bc	ad	ac-	ac-	ac-	ac-	bc	ac-	ac-	ac-	ac	ac-	ac-	ac
NH029a	9	bc	ad	bcd	bcd	bcd	bcd	acd	bc	bcd	acd	bd	ac	acd	bcd	bcd	bcd	bd	acd	bcd	bcd	ad	bcd	bcd	ac
CH01h02_1	9	hh	k-	hk-	hhk	hkk	hhk	hkk	hh	hhk	hkk	k-	hk	hkk	hhk	hhk	hkk	k-	hkk	hhk	hkk	k-	hhk	hkk	hk
Hi01d01	9	lm	ll	llm	llm	llm	llm	lll	lm	llm	lll	lm	ll	lll	llm	llm	llm	lm	lll	lll	lll	ll	llm	llm	ll
CH05d08y_2	9	np	nn	np1p2	np1p2	np1p2	np1p2	np1p2	np	np1p2	np1p2	np	nn	np1p2	np1p2	np1p2	np1p2	np	np1p2	np1p2	np1p2	nn	np1p2	np1p2	np
CH01f07a	10	bc	bd	bd	bcd	acd	bd	acd	bc	acd	acd	ac	ad	bd	bc	acd	acd	bcd	bcd	acd	acd	acd	bcd	bcd	ac
CH01f12	10	ac	bd	bd	bcd	acd	bd	bcd	bc	acd	bcd	bc	bd	ad	bc	bcd	bcd	acd	bcd	acd	acd	acd	acd	bcd	ac
CH02b03b	10	ac	ad	ac	ac-	bc-	ac	bc-	ad	bc-	bc-	bd	bc	ac	ad	bc-	bc-	ac-	ac-	bc-	bc-	bc-	ac-	ac-	bd
CH02b07	10	bc	ad	ac	acd	bcd	bd	acd	ad	bcd	acd	ac	ad	bd	ac	bcd	bcd	acd	bcd	bcd	bcd	bcd	bcd	acd	bc
CH02c11	10	ac	ad	ac	ac-	bc-	ac	bc-	ad	bc-	bc-	bc	bc	ac	ad	bc-	bc-	ac-	ac-	bc-	bc-	bc-	ac-	ac-	bd
MS02a01	10	bd	bd	ad	ac-	c--	ac	c--	--	c--	ac-	ad	ac	bc	ad	c--	c--	ac-	bc	c--	c--	c--	c--	ac-	ad
MS06g03	10	ac	ad	ac	ac-	bc-	ac	bc-	ad	bc-	bc-	bd	bc	ac	ad	bc-	bc-	ac-	ac-	bc-	bc-	bc-	ac-	ac-	bd

Markers	LG	Aneuploid seedlings from the cross of ‘Pink Lady × Fuji’																							
		PF01	PF02	PF03	PF04	PF05	PF06	PF07	PF08	PF09	PF10	PF11	PF12	PF13	PF14	PF15	PF16	PF17	PF18	PF19	PF20	PF21	PF22	PF23	PF24
CH02a10	10	lm	ll	lm	llm	llm	ll	lll	lm	lll	lll	lm	ll	ll	lm	llm	llm	llm	lll	lll	llm	llm	llm	lll	lm
CH03d11	10	ll	ll	lm	lll	lll	lm	lll	ll	llm	llm	ll	lm	lm	ll	lll	lll	llm	llm	lll	lll	lll	lll	llm	ll
CH04c06y_1	10	lm	ll	ll	lll	lm	lm	llm	ll	llm	lll	ll	ll	lm	ll	llm	llm	lll	llm	llm	llm	llm	llm	lll	lm
Hi02d04	10	ll	ll	ll	llm	lll	lm	lll	lm	lll	llm	lm	lm	lm	lm	lll	lll	llm	lll	lll	lll	lll	llm	llm	lm
Hi04f08	10	np	nn	np	nnp	nnp	nn	nnp	np	nnp	nnp	np	nn	np	np	nnp	nnp	nnp	nnp	nnp	nnp	nnp	nnp	nnp	np
CH02d08	11	bd	ac	bc	bd	bcd	ac	ac	bc	bd	bc	bc	acd	ad	bd	bcd	ac	bc	bc	bd	acd	ad	bcd	bc	bcd
CH04g07	11	ad	ac	ad	bc	bcd	bc	bc	bc	bd	ad	bc	bcd	bd	ad	acd	bd	bd	bc	ad	acd	bd	bcd	bc	acd
CH04h02_1	11	bc	bd	bd	bc	ac-	bd	bd	ad	ac	bd	ad	c--	bc	ac	ac-	bd	ad	ad	ac	c--	ac	ac-	bd	ac-
Hi06b06	11	ac	ad	ac	bd	bcd	bd	bd	bd	bc	ac	bd	bcd	ac	ac	ac	bc	bc	bd	ac	ad	bc	bcd	bd	ad
CH04h02_3	11	nn	np	np	nn	nnp	nn	nn	np	np	np	np	nnp	nn	np	nnp	nn	np	nn	np	nnp	nn	nnp	np	nnp
CH01b12y	12	ad	acd	bc	ad	acd	ad	acd	ac	bcd	acd	acd	ac	ad	bc	acd	bcd	acd	acd	acd	acd	bcd	acd	acd	acd
CH01f02	12	ac	acd	ad	ac	acd	ac	bcd	ad	bcd	acd	acd	ad	ac	bd	acd	bcd	acd	acd	acd	acd	bcd	acd	acd	acd
CH01g12	12	bc	bcd	ad	bc	bcd	bc	bcd	bd	acd	bcd	bcd	bd	bc	ad	bcd	acd	bcd	bcd	bcd	bcd	acd	bcd	bcd	bcd
NZ28f04	12	bd	bcd	ac	bd	bcd	bd	acd	bc	acd	bcd	bcd	bc	bd	ac	bcd	acd	bcd	bcd	bcd	bcd	acd	bcd	bcd	bcd
CH05d04	12	eg	efg	ee	fg	efg	eg	eef	ee	eef	efg	efg	eg	eg	ef	efg	eef	efg	efg	efg	efg	eef	eef	efg	efg
CH05d11	12	ef	efg	fg	ee	efg	fg	eeg	fg	eeg	efg	efg	ef	ef	ee	efg	eeg	efg	efg	efg	efg	eeg	eeg	efg	efg
CH03h03z_2	12	nn	nnp	np	nn	nnp	np	nnp	np	nnp	nnp	nnp	np	nn	np	nnp	nnp	nnp	nnp	nnp	nnp	nnp	nnp	nnp	nnp
CH03a08	13	bd	ad	ac	acd	bd	acd	acd	bc	acd	ad	ac	bd	bcd	bc	acd	bcd	ac	acd	bcd	ad	bd	bc	bcd	acd
CH03h03z_1	13	ad	ac	bc	c--	bc	c--	c--	bc	bc-	ac	ad	bc	bc-	bd	c--	bc-	ad	c--	bc-	ac	bc	bd	bc-	c--
CH05f04	13	ac	ad	ac	ac-	bd	ac-	ac-	bc	ac-	ad	ac	ad	ac-	bc	ac-	ac-	bd	ac-	ac-	ac	ad	ad	ac-	ac-
CH05h05	13	bc	bd	ac	acd	bc	acd	acd	bd	bcd	ac	ad	ac	acd	bd	bcd	bcd	bc	acd	bcd	ad	ac	ac	bcd	bcd
Hi05c06_2	13	ac	bd	bc	bcd	bd	acd	bcd	ac	bcd	bd	ac	bd	acd	bc	acd	bcd	ac	acd	acd	ad	bd	ac	acd	bd
Hi20b03	13	bc	bd	ad	bcd	ad	bcd	acd	ac	acd	ad	bc	ad	acd	ad	bcd	acd	bc	bcd	acd	--	ad	bc	acd	bcd
NH009b	13	bc	bc	ad	acd	bc	acd	bcd	bd	bcd	ac	ad	bc	bcd	bc	acd	bcd	ad	acd	bcd	ac	bc	ad	bcd	acd
GD147	13	hk	h-	hk	hkk	kk	hhk	hkk	hk	hhk	kk	kk	h-	hkk	h-	hhk	hkk	h-	hkk	hhk	kk	h-	hk	hhk	hkk
Hi03e04	13	kk	h-	h-	hhk	kk	hkk	hhk	hk	hkk	h-	h-	kk	hhk	kk	hkk	hhk	h-	hhk	hkk	h-	kk	hk	hkk	hhk
Hi07b02_3	13	ll	ll	lm	lll	ll	lll	lll	lm	llm	lm	ll	lm	llm	lm	lll	llm	lm	llm	llm	ll	ll	ll	llm	llm
NZ03c01x_2	13	lm	lm	lm	llm	ll	llm	llm	ll	lll	lm	ll	ll	lll	ll	llm	lll	lm	llm	llm	lm	lm	ll	lll	lll
AU223486	13	nn	nn	np	nnp	np	nnp	nnp	nn	nnp	np	np	nn	nnp	nn	nnp	nnp	nn	nnp	nnp	np	np	np	nnp	nnp
CH05c06_1	13	nn	nn	np	nnp	np	nnp	nnp	nn	nnp	np	np	nn	nnp	nn	nnp	nnp	nn	nnp	nnp	np	np	np	nnp	nnp
CH01g05	14	ac	bc	ad	acd	ad	bcd	ad	acd	ad	ad	bc	bc	bc	bd	ac	ad	ac	ac	ac	ad	ad	ac	bc	acd
CH03a02	14	ac	ac	ad	acd	ac	bcd	ac	acd	ac	bd	bd	bd	ad	bc	ac	ac	ac	ac	ac	ac	ad	ad	ad	acd
CH03d08	14	bc	bc	bd	bcd	bc	acd	bc	bcd	bc	ad	ad	ad	ad	ac	bd	bc	bd	bd	bd	bc	bc	bd	ad	bcd
CH05g07z_1	14	k-	hk	hk	hhk	hk	hkk	hh	hhk	k-	hk	k-	k-	hk	hh	k-	k-	hk	hk	hk	hk	k-	k-	k-	hhk

Markers	LG	Aneuploid seedlings from the cross of ‘Pink Lady × Fuji’																							
		PF01	PF02	PF03	PF04	PF05	PF06	PF07	PF08	PF09	PF10	PF11	PF12	PF13	PF14	PF15	PF16	PF17	PF18	PF19	PF20	PF21	PF22	PF23	PF24
CH05g07z_2	14	hk	hk	h-	hkk	hk	hhk	kk	hkk	h-	hk	h-	h-	hk	kk	h-	h-	hk	hk	hk	hk	h-	h-	h-	hkk
CH02c02a_1	15	bd	ad	ad	ac	c--	bd	ac-	ac-	ad	ad	c--	ac-	bc	ac	ad	ac-	bc	ad	ad	c--	ac-	c--	c--	ac
CH02d11	15	bd	ac	ad	bd	acd	ad	acd	bcd	bd	bc	bcd	bcd	ac	bc	bd	acd	bc	bd	bc	acd	bcd	acd	bcd	ac
CH03b10	15	ac	bd	bc	bc	acd	ac	acd	bcd	bc	ad	bcd	bcd	ad	bd	bc	acd	bd	bc	bd	acd	bcd	acd	bcd	ad
Hi04c05	15	ac	ad	ac	ac	c--	bd	c--	ac-	ad	bc	ac-	ac-	bc	ac	ad	c--	ac	ad	ac	c--	ac-	c--	ac-	bc
NZ02b01	15	bc	ad	ac	ac	bc-	bc	bc-	ac-	ac	bd	ac-	ac-	bd	ad	ac	bc-	ad	ac	ad	bc-	ac-	bc-	ac-	bd
CH02c09	15	ll	lm	lm	lm	lll	ll	llm	llm	lm	ll	llm	lll	ll	lm	lm	llm	lm	ll	lm	lll	llm	llm	llm	lm
Hi02g06	15	nn	nn	nn	np	np1p2	np	np1p2	np1p2	nn	nn	np1p2	np1p2	np	np	np	np1p2	np	np	nn	np1p[illegible]	np1p2	np1p2	np1p2	nn
Hi06f09	15	nn	np	nn	nn	np1p2	nn	np1p2	np1p2	np	nn	np1p2	np1p2	nn	nn	nn	np1p2	np	nn	np	np1p[illegible]	np1p2	np1p2	np1p2	nn
CH02d10a	16	bc	bc	ad	bc-	bc	bc	bc	bd	ad	bc-	bc-	bc-	bc-	ac-	bd	ad	bc-	bd	bc	bc-	ac-	bc-	bc-	bc-
CH05a04	16	ac	bc	bc	acd	ad	bd	bc	ad	bd	bcd	acd	bcd	acd	acd	ac	bd	bcd	ad	ac	bcd	bcd	acd	acd	acd
CH05b06z_1	16	bd	bd	ad	bcd	bc	ad	ad	bc	ac	acd	acd	acd	bcd	bcd	bc	ac	acd	bc	bd	acd	acd	bcd	bcd	bcd
CH05c06_2	16	ad	bd	ac	acd	ad	bd	ad	bd	bd	acd	acd	bcd	bcd	bcd	bc	ad	acd	bd	bd	bcd	bcd	acd	acd	bcd
Hi01c11x	16	fg	eg	ef	eeg	fg	eg	eg	eg	eg	eeg	efg	eeg	eeg	eeg	eg	fg	efg	eg	eg	efg	efg	efg	efg	eeg
Hi01d06y	16	eg	fg	ee	efg	eg	fg	eg	fg	fg	eeg	eeg	efg	efg	efg	fg	eg	eeg	fg	fg	eeg	eeg	eeg	eeg	efg
Hi04e04	16	fg	eg	ef	eeg	fg	eg	eg	eg	eg	efg	efg	eeg	eeg	eeg	eg	fg	efg	eg	eg	efg	efg	efg	efg	eeg
CH04f10	16	nn	nn	np	nnp	nn	np	np	nn	np	nnp	nnp	nnp	nnp	nnp	nn	np	nnp	nn	nn	nnp	nnp	nnp	nnp	nnp
CH01h01	17	b	bd	bd	bcd	acd	bd	bd	acd	bd	bcd	bcd	bcd	bcd	ac	bc	bcd	acd	bd	bcd	bcd	acd	acd	bcd	acd
CH05d08y_1	17	a	bd	ac	c--	c--	bc	ac	c--	ac	ac-	ac-	c--	ac	ad	ac	c--	ac-	bc	c--	ac-	c--	ac-	ac-	c--
CH04c06y_3	17	m	lm	lm	llm	llm	lm	ll	llm	ll	lll	lll	lll	lll	lm	ll	lll	llm	ll	llm	lll	lll	lll	llm	llm
GD96	17	l	lm	ll	llm	llm	ll	ll	llm	ll	llm	lll	llm	lll	lm	lm	llm	llm	ll	llm	llm	lll	llm	llm	llm
Hi03c05	17	l	lm	lm	llm	llm	ll	ll	llm	ll	lll	lll	llm	lll	lm	ll	llm	llm	lm	lll	lll	llm	lll	lll	llm
Hi05c06_1	17	m	ll	lm	lll	lll	lm	lm	lll	lm	llm	llm	lll	llm	lm	lm	lll	llm	ll	lll	llm	lll	llm	llm	lll
Hi07b02_1	17	l	lm	ll	llm	lll	lm	ll	llm	ll	llm	lll	llm	lll	lm	ll	llm	llm	lm	llm	llm	llm	llm	llm	llm
CH04c06y_2	17	n	np	np	nnp	nnp	nn	np	nnp	np	nnp	nnp	nnp	nnp	nn	np	nnp	nnp	np	nnp	nnp	nnp	nnp	nnp	nnp
CH05g03	17	n	np	nn	np1p2	np1p2	nn	nn	np1p2	nn	np1p2	np1p2	np1p2	np1p[illegible]	nn	nn	np1p2	np1p2	np	np1p2	np1p2	np1p2	np1p2	np1p2	np1p2
Hi07b02_2	17	n	np	nn	nnp	nnp	np	nn	nnp	nn	nnp	nnp	nnp	nnp	np	np	nnp	nnp	np	nnp	nnp	nnp	nnp	nnp	nnp

Note: ‘-‘ represents a null allele, or missing data; ‘p1’ and ‘p2’ are con-dominant alleles.
