# Supplementary material for: Molecular Genetic Features of Polyploidization and Aneuploidization Reveal Unique Patterns for Genome Duplication in Diploid Malus
Source: PLoS One. 2012 Jan 10;7(1):e29449. doi: 10.1371/journal.pone.0029449 (PMC3254611; doi:10.1371/journal.pone.0029449)
Supplement: Table S5 — The distributional features of microsatellite markers in the aneuploid seedlings from the cross of ‘M 26×Fu 2’. (PDF) [file pone.0029449.s006.pdf]

Markers	LG	Aneuploid seedlings from the cross of ‘M 26 × Fu 2’																						
		M26F01	M26F02	M26F03	M26F04	M26F05	M26F06	M26F07	M26F08	M26F09	M26F10	M26F11	M26F12	M26F13	M26F14	M26F15	M26F16	M26F17	M26F18	M26F19	M26F20	M26F21	M26F22	M26F23
CH05g08	1	bd	bc	bd	bc	ad	bc	ad	bd	bd	bd	bc	bc	bc	ac	bc	ad	bc	bc	bc	ac	bc	ac	bc
Hi02b10	1	ac	bd	ac	ac	ad	ad	bd	ad	ad	ad	ac	ac	ac	bc	ac	bd	ac	ad	ac	bc	ac	bc	ac
Hi07d08	1	bc	ad	bc	bd	bd	ad	ad	ad	bc	bc	bd	ad	bc	ad	bd	ac	bd	bd	bd	bd	bd	bd	bd
Hi12c02	1	bc	ad	ad	ac	ac	bc	bc	bc	ad	ad	ac	bc	ad	bc	ac	bd	ac	ac	ac	ac	ac	ac	ac
KA4B	1	ad	ac	ac	ad	bc	ad	bc	ac	ac	ac	ad	ad	ad	bd	ad	bc	ad	ad	ad	bd	ad	bd	ad
Hi02c07	1	kk	hk	hk	kk	h-	kk	h-	h-	h-	hk	kk	kk	kk	h-	kk	h-	kk	kk	kk	h-	kk	h-	kk
CH02a04z	2	ad	bc	bcd	bd	ad	bcd	acd	ac	ad	acd	ad	acd	ac	acd	ad	acd	acd	acd	bcd	ad	bc	acd	acd
CH02c06	2	bc	ad	bcd	ad	ad	acd	acd	ac	bc	bcd	bd	bcd	ad	bcd	bd	bcd	acd	acd	bcd	bc	ac	acd	acd
CH03d01	2	ac	bd	acd	ad	bd	acd	bcd	bc	bd	bcd	bd	bcd	bc	acd	bd	bcd	bcd	bcd	acd	bd	ac	bcd	bcd
CH05e03	2	ad	bc	acd	ac	bc	acd	bcd	bd	bc	bcd	bc	bcd	bd	bcd	bc	bcd	bcd	bcd	acd	bc	ad	bcd	bcd
CN493139	2	bd	ac	acd	bc	bc	bcd	bcd	ad	ac	acd	bc	acd	ac	acd	ac	acd	acd	acd	acd	ac	bd	bcd	acd
Hi02a07	2	bd	ac	bcd	bc	ac	bcd	acd	ad	ac	acd	ac	acd	ad	acd	ac	acd	acd	acd	bcd	ac	bd	acd	acd
CH03d10	2	h-	kk	hkk	h-	kk	hkk	hhk	hk	kk	hhk	kk	hkk	h-	hhk	kk	hkk	hhk	hkk	hhk	kk	h-	hkk	hhk
CH02c02a_2	2	lm	ll	lll	lm	lm	llm	llm	ll	ll	lll	lm	lll	ll	lll	lm	llm	llm	lll	lll	lm	lm	llm	llm
CH02c02a_3	2	np	nn	np1p2	nn	nn	np1p	np1p2	nn	nn	np1p2	nn	np1p	nn	np1p2	nn	np1p2	np1p	np1p2	np1p2	nn	np	np1p2	np1p2
Hi05c06_3	2	np	nn	nnp	nn	nn	nnp	nnp	np	nn	nnp	nn	nnp	np	nnp	nn	nnp	nnp	nnp	nnp	nn	np	nnp	nnp
AU223657	3	bd	acd	bc	bcd	bcd	bd	acd	bcd	bd	bcd	ac	acd	ac	ad	bcd	bd	bc	bcd	ad	bcd	bc	bc	acd
CH03e03	3	bd	acd	bd	bcd	bcd	bd	acd	acd	bd	acd	ad	acd	bc	ad	bcd	bd	bc	bcd	ad	bcd	ac	ad	acd
Hi07e08x	3	ac	bcd	ac	acd	acd	ac	bcd	bcd	ac	bcd	bc	bcd	ad	bc	acd	ac	ad	acd	bc	acd	bd	bc	bcd
CH03g07	3	ee	efg	ee	eeg	eeg	fg	eeg	eeg	eg	eeg	ee	eeg	fg	fg	eeg	eg	ef	efg	fg	eeg	eg	ef	efg
HGA8bY	3	np	np1p2	nn	np1p	np1p2	np	np1p2	np1p	nn	np1p2	nn	np1p	np	np	np1p2	nn	np	np1p2	np	np1p	nn	np	np1p2
Hi04c10x_1	3	ll	ll	lm	llm	llm	ll	llm	llm	lm	llm	lm	llm	ll	ll	llm	lm	ll	lll	ll	llm	lm	ll	lll
CH02c02b	4	bcd	ac	bc	bcd	acd	bc	ad	ad	acd	ad	acd	acd	acd	ac	ac	ac	acd	acd	bcd	ac	bd	ac	acd
CH04e02	4	acd	ad	bc	acd	bcd	ac	ad	bc	acd	ad	bcd	acd	acd	bc	bd	ac	acd	acd	bcd	bc	bc	ac	--
GD162	4	bcd	ad	ac	bcd	acd	bd	bc	ad	bcd	bc	acd	bcd	bcd	ad	ac	bd	bcd	bcd	acd	ad	ad	bd	bcd
Hi04c10x_3	4	ac-	bd	ac	ac-	bc-	ad	ac	bd	ac-	ac	bc-	ac-	ac-	bd	bc	ac	ac-	ac-	bc-	bd	bd	ad	ac-
CH05d02	4	efg	eg	ef	efg	efg	ee	ee	ef	efg	eg	eef	efg	efg	ef	fg	ee	efg	eef	eef	fg	ef	eg	efg
Hi07b02_4	4	efg	eg	ef	efg	efg	fg	fg	eg	efg	ef	eeg	efg	efg	eg	ee	fg	efg	eeg	eeg	ee	eg	ef	efg
CH03a04	5	ad	bd	bd	bcd	bd	bd	bcd	bc	acd	bcd	bd	bcd	bcd	ad	bcd	bcd	bcd	bc	bcd	bd	acd	bc	bcd
CH03a09	5	bc	ac	ac	acd	ac	ac	acd	ad	bcd	acd	ac	acd	acd	bc	acd	acd	acd	ad	acd	ac	bcd	ad	acd
CH04g09y	5	bc	ac	ad	bcd	ad	bc	bcd	bd	bcd	bcd	bc	bcd	acd	ac	bcd	acd	bcd	bd	acd	bc	acd	ac	bcd
CH05e06	5	ac	bd	ad	bcd	bc	bd	bcd	bc	bcd	bcd	bd	bcd	acd	ad	bcd	acd	bcd	bc	acd	bd	bcd	ad	bcd
Hi04d02	5	ac	bd	bc	bcd	bd	ac	acd	ad	bcd	acd	ac	acd	bcd	bc	acd	bcd	acd	ad	bcd	ac	bcd	bc	acd
Hi11a03	5	ad	bc	bd	acd	ac	ad	acd	ac	acd	acd	ad	acd	bcd	bd	acd	bcd	acd	ac	bcd	ad	acd	bd	acd

Markers	LG	Aneuploid seedlings from the cross of 'M 26 × Fu 2'																						
		M26F01	M26F02	M26F03	M26F04	M26F05	M26F06	M26F07	M26F08	M26F09	M26F10	M26F11	M26F12	M26F13	M26F14	M26F15	M26F16	M26F17	M26F18	M26F19	M26F20	M26F21	M26F22	M26F23
CH04e03	5	fg	ef	fg	eeg	ee	fg	eeg	eg	eeg	eeg	eg	eeg	efg	fg	eeg	efg	eeg	ee	efg	eg	eeg	fg	eeg
CH04h02_2	5	hh	hh	hh	hhk	hk	hk	hkk	hk	hhk	kk	hk	hk-	hkk	hh	hhk	hkk	hhk	hk	hkk	hk	hkk	hh	hkk
CH04h02_4	5	h-	kk	h-	hk-	kk	h-	hk-	h-	hk-	h-	hh	hkk	hk-	h-	hk-	hk-	hk-	kk	hk-	h-	hk-	h-	hk-
Hi21c08	5	kk	kk	kk	hkk	hk	hk	hhk	hk	hkk	hhk	hk	hkk	hhk	kk	hkk	hhk	hkk	hk	hhk	hk	hkk	kk	hhk
CH02a08z	5	np	nn	np	np1p2	nn	np	np1p2	np	np1p2	np1p2	np	np1p	np1p2	np	np1p2	np1p2	np1p2	nn	np1p2	np	np1p2	np	np1p2
AJ000761	6	ac	ac	bd	acd	acd	ac	bcd	ad	bcd	bd	ac	bcd	acd	acd	acd	bcd	bcd	bcd	bcd	bd	bcd	bd	ad
CH03d07	6	ad	bc	bc	bcd	bcd	bc	acd	bd	bcd	ad	bc	acd	bcd	bcd	bcd	acd	acd	acd	bcd	ad	acd	ad	bd
CH03d12	6	ac	bd	bd	bcd	bcd	bd	acd	bc	acd	ac	bd	acd	bcd	bcd	bcd	acd	acd	acd	acd	ac	acd	ac	bc
CH03c01	6	eg	ef	ee	eeg	efg	eg	eeg	ef	efg	ef	ef	efg	efg	eeg	efg	eeg	eeg	efg	eeg	ee	eeg	ef	fg
Hi01d05	6	fg	eg	fg	efg	efg	fg	eeg	ef	eeg	ee	ef	eeg	efg	efg	efg	eeg	eeg	eeg	eeg	ef	eeg	ee	ef
Hi04c10x_2	7	ad	bc	bd	bc	ac	bd	ac	ad	ac	ad	bc	bc	bd	acd	bd	bd	ac	ad	ad	bc	acd	acd	ad
Hi05b09	7	ac	bc	ad	ad	bd	ac	bd	bc	bd	bc	ad	ad	ac	c--	ac	ac	bd	bc	bc	ad	c--	c--	bc
CH04e05	7	lm	ll	lm	lm	ll	lm	ll	ll	ll	ll	lm	lm	lm	lll	lm	lm	ll	ll	ll	lm	lll	lll	ll
CH05b06z_2	7	np	nn	nn	nn	nn	np	np	np	nn	nn	nn	nn	np	nnp	nn	np	nn	nn	nn	nn	nnp	nnp	np
CH01c06	8	bd	ac	ac	ad	bd	bc	bc	bc	bd	ac	ac	bd	ad	bc	ac	ac	bc	bc	bc	bd	ac	ad	bd
CH02g09	8	bc	ad	ad	ac	bc	bd	bd	bd	bc	ad	ad	bc	ac	bd	ad	ad	bd	bd	bd	bd	ad	ac	bc
Hi04b12	8	bc	ad	bc	bd	ad	ac	ac	ac	ad	bc	bc	ad	bd	ac	bc	bc	ac	ac	ac	ad	bc	bd	ad
Hi23g12	8	bd	ac	bd	bc	ac	ad	ad	ad	ac	bd	bd	ac	bc	ad	bd	bd	ad	ad	ad	ac	bd	bc	ac
Hi04e05	8	lm	ll	lm	lm	ll	lm	ll	ll	ll	lm	lm	ll	lm	ll	lm	lm	ll	ll	lm	ll	lm	lm	ll
CH01h02_1	9	ac	acd	bcd	bc	bcd	bd	ac	acd	acd	bcd	bcd	bd	acd	bcd	acd	bcd	bcd	ad	acd	acd	bcd	acd	acd
CH01h02_2	9	ac	ac-	bc-	bd	bc-	bc	ad	ac-	ac-	bc-	bc-	bc	ac-	bc-	ac-	bc-	bc-	ac	ac-	ac-	bc-	ac-	ac-
CH05c07	9	ac	acd	bcd	bc	acd	ad	bd	acd	bcd	acd	bcd	ad	bcd	bcd	acd	bcd	bcd	bc	bcd	bcd	bcd	acd	acd
GD142	9	bc	acd	bcd	bd	bcd	bc	ad	acd	acd	acd	acd	bc	acd	bcd	bcd	acd	acd	ad	acd	acd	bcd	acd	acd
Hi05e07	9	bc	bcd	acd	ac	bcd	ad	bc	bcd	bcd	acd	acd	ad	bcd	acd	bcd	acd	acd	bd	bcd	acd	acd	bcd	bcd
NH029a	9	bd	bcd	acd	ad	acd	ac	bd	bcd	bcd	acd	acd	ac	bcd	acd	bcd	acd	acd	bc	bcd	bcd	acd	bcd	bcd
CH05d08y_2	9	fg	efg	eef	ee	eeg	eg	ef	efg	efg	efg	eeg	eg	eeg	eeg	eeg	efg	efg	ef	efg	efg	eeg	efg	efg
Hi01d01	9	fg	efg	eeg	ee	eeg	eg	ef	efg	eeg	efg	eeg	eg	efg	eeg	efg	eeg	efg	ef	eef	efg	eeg	efg	efg
CH01f07a	10	bc	ac	acd	bd	acd	acd	ad	acd	bc	bc	acd	bc	bcd	bcd	ac	ac	bcd	acd	bcd	bcd	acd	acd	ac
CH01f12	10	bd	bd	bcd	ad	bcd	bcd	ad	acd	bc	ac	acd	bd	bcd	bcd	ad	bd	bcd	bcd	bcd	acd	acd	acd	ac
CH02a10	10	bd	ad	bcd	ac	acd	bcd	bc	bcd	ad	ad	bcd	ad	acd	acd	bd	bd	acd	bcd	bcd	acd	bcd	bcd	bd
CH02b03b	10	bc	ac	bcd	ad	bcd	bcd	bd	bcd	ac	ac	bcd	ac	acd	acd	bc	bc	acd	bcd	acd	acd	bcd	bcd	bc
CH02b07	10	bd	ad	acd	bd	bcd	bcd	bd	bcd	bc	bc	bcd	ad	acd	acd	ad	ad	acd	acd	acd	bcd	bcd	bcd	bc
CH03d11	10	bc	bd	c--	ad	c--	c--	ac	c--	bd	bd	c--	bd	bd-	bd-	ad	ad	bd-	c--	bd-	bd-	c--	c--	ad
Hi02d04	10	ad	bd	acd	ad	acd	acd	ad	acd	ac	ac	acd	bd	bcd	acd	bd	bd	bcd	bcd	bcd	acd	acd	acd	ac

Markers	LG	Aneuploid seedlings from the cross of ‘M 26 × Fu 2’																						
		M26F01	M26F02	M26F03	M26F04	M26F05	M26F06	M26F07	M26F08	M26F09	M26F10	M26F11	M26F12	M26F13	M26F14	M26F15	M26F16	M26F17	M26F18	M26F19	M26F20	M26F21	M26F22	M26F23
Hi04f08	10	bc	ac	c--	bc	c--	c--	bc	c--	bd	bd	c--	ac	ac-	c--	ac	ac	ac-	ac-	ac-	c--	c--	c--	bd
MS06g03	10	bc	ac	c--	ad	c--	c--	bd	c--	ac	ac	c--	ac	ac-	ac-	bc	bc	c--	c--	ac-	ac-	c--	c--	bc
CH04c06y_1	10	lm	ll	lll	lm	llm	llm	lm	llm	lm	lm	llm	ll	lll	lll	ll	ll	lll	lll	lll	llm	llm	llm	lm
CH02c11	10	nn	nn	np1p2	nn	np1p2	np1p2	nn	np1p2	np	np	np1p2	nn	np1p2	np1p2	nn	nn	np1p2	np1p2	np1p2	np1p2	np1p2	np1p2	np
MS02a01	10	nn	nn	np1p2	np	np1p2	np1p2	np	np1p2	nn	nn	np1p2	nn	np1p2	np1p2	nn	nn	np1p2	np1p2	np1p2	np1p2	np1p2	np1p2	nn
CH02d08	11	bc	bd	bcd	bd	ad	bd	ad	ac	bd	bd	acd	ac	bcd	bc	ac	bcd	bc	acd	ad	ad	ac	ac	acd
CH04g07	11	ac	bd	acd	ad	bd	ac	bd	ad	ad	ac	bcd	bd	acd	bd	bd	acd	bc	bcd	bd	bd	bd	bc	bcd
Hi06b06	11	ad	bc	acd	ac	bc	ad	bc	ac	ac	ad	bcd	bc	acd	bc	bc	acd	bd	bcd	bc	bc	bc	bd	bcd
CH04h02_1	11	np	nn	np1p	nn	np	np	np	nn	nn	np	np1p	nn	np1p	nn	nn	np1p	nn	np1p2	np	np	nn	nn	np1p
CH04h02_3	11	np	np	nnp	nn	nn	nn	nn	np	np	nn	nnp	np	nnp	np	np	nnp	np	nnp	nn	nn	np	np	nnp
CH01b12y	12	bc	ac	bd	bd	ac	acd	acd	acd	acd	ac	bcd	acd	acd	bcd	acd	ac	bcd	ad	bd	acd	ac	bcd	acd
CH01g12	12	ad	bc	ad	ac	bc	bcd	acd	bcd	bcd	bd	acd	bcd	bcd	acd	bcd	bc	bcd	bd	ad	bcd	bc	acd	bcd
CH05d04	12	ac	bd	ac	ad	bd	bcd	bcd	bcd	bcd	bd	acd	bcd	bcd	acd	bcd	bd	acd	bc	ac	bcd	bd	acd	bcd
CH05d11	12	bd	bd	bc	bd	bd	acd	acd	acd	acd	ac	acd	acd	bcd	bcd	acd	ad	bcd	ac	bc	acd	ad	acd	acd
NZ28f04	12	ac	ac	ad	ac	ac	bcd	bcd	bcd	bcd	bd	acd	bcd	bcd	acd	bcd	bc	acd	bd	ad	bcd	bc	acd	bcd
CH01f02	12	hk	hh	hk	kk	hk	hk-	hk-	hk-	hk-	hh	hk-	hk-	hk-	hk-	hk-	hk	hk-	hh	kk	hk-	hh	hk-	hk-
CH03h03z_2	12	np	np	nn	np	np	np1p2	np1p2	np1p2	np1p2	nn	np1p2	np1p2	np1p2	np1p2	np1p2	np	np1p2	nn	nn	np1p2	np	np1p2	np1p2
CH03a08	13	bc	bc	ad	bd	ac	ac	bc	ac	bcd	bcd	bd	bd	bd	ac	acd	bc	ad	bd	acd	acd	acd	acd	acd
CH05c06_1	13	bc	ad	bc	bd	ad	bc	ad	bd	bd-	bd-	bd	ad	ad	ad	bd-	bc	bc	bc	bd-	bd-	c--	c--	c--
CH05f04	13	ad	bd	ac	ac	bd	ac	bd	ad	acd	bcd	ad	bd	bd	bd	acd	ac	ac	ac	acd	acd	bcd	bcd	acd
CH05h05	13	ac	bc	ad	ad	bc	ad	bc	ac	acd	acd	ac	bc	bc	bc	acd	ad	ad	ad	acd	acd	bcd	bcd	acd
GD147	13	ac	ac	bd	ad	bc	bc	ac	bc	acd	acd	ad	ad	ad	bc	bcd	ac	bd	ad	bcd	bcd	bcd	bcd	bcd
Hi03e04	13	ac	ad	ad	ad	bc	ad	ad	ac	acd	acd	bc	ac	bd	bc	bcd	ad	ac	bd	bcd	bcd	bcd	bcd	acd
Hi20b03	13	ad	ac	bd	ac	ac	bc	ac	bc	bcd	acd	ac	ad	ad	ac	acd	ac	bd	ad	bcd	bcd	bcd	bcd	bcd
NH009b	13	bd	bd	ac	bc	bd	ad	bd	ad	acd	bcd	bd	bc	bc	bd	bcd	bd	ac	bc	acd	acd	acd	acd	acd
AU223486	13	hh	hk	k-	hk	k-	k-	hk	k-	hk-	hk-	hh	hh	hh	k-	hk-	hk	k-	hh	hk-	hk-	hk-	hk-	hk-
CH03h03z_1	13	hk	hk	k-	hh	k-	k-	hk	k-	hk-	hk-	hh	hh	hh	k-	hk-	hk	k-	hh	hk-	hk-	hk-	hk-	hk-
Hi05c06_2	13	h-	h-	kk	h-	hk	hk	h-	hk	hk-	hk-	h-	h-	h-	h-	hk-	h-	kk	h-	hk-	hk-	hk-	hk-	hk-
Hi07b02_3	13	ll	ll	lm	lm	ll	lm	ll	lm	llm	lll	ll	ll	ll	ll	lll	ll	lm	ll	llm	llm	llm	lll	llm
NZ03c01x_2	13	np	np	nn	nn	nn	np	np	np	np1p2	np1p2	np	nn	np	np	np1p2	np	nn	np	np1p2	np1p2	np1p2	np1p2	np1p2
CH03a02	14	ac	bd	acd	acd	ad	ac	ac	ac	acd	bc	bcd	bc	bc	bd	bc	acd	ac	ad	ac	ac	acd	bcd	bcd
CH03d08	14	bc	ad	bcd	bcd	bd	bc	bc	bd	bcd	bd	acd	ac	ac	ad	ad	bcd	bc	bd	ad	bc	bcd	acd	acd
CH01g05	14	k-	k-	hk-	hk-	k-	k-	k-	hk	hk-	hk	hk-	hh	k-	hk	hk	hk-	k-	hk	hk	hk	hk-	hk-	hk-
CH05g07z_1	14	h-	h-	hk-	hk-	h-	kk	h-	hk	hk-	hk	hk-	kk	h-	hk	hk	hk-	h-	hk	hk	hk	hk-	hk-	hk-

Markers	LG	Aneuploid seedlings from the cross of ‘M 26 × Fu 2’																						
		M26F01	M26F02	M26F03	M26F04	M26F05	M26F06	M26F07	M26F08	M26F09	M26F10	M26F11	M26F12	M26F13	M26F14	M26F15	M26F16	M26F17	M26F18	M26F19	M26F20	M26F21	M26F22	M26F23
CH05g07z_2	14	h-	h-	hk-	hk-	h-	h-	h-	hk	hk-	hk	hk-	kk	h-	hk	hk	hk-	h-	hk	hk	hk	hk-	hk-	hk-
CH02c02a_1	15	bd	acd	ad	bcd	bc	ac	ad	ad	bc	ac	bd	ac	bd	ad	bc	ac	bd	bd	bc	bd	bd	bd	bc
CH02c09	15	bc	bcd	bc	acd	ad	bcd	acd	bcd	bd	acd	ac	bcd	ac	bc	ad	bcd	bc	acd	ad	acd	acd	acd	acd
CH02d11	15	bc	bcd	bd	acd	ac	bcd	acd	bcd	bd	acd	ad	bcd	ad	bd	ac	bcd	bd	acd	ac	acd	acd	acd	acd
CH03b10	15	ac	acd	ad	bcd	bc	acd	bcd	acd	ad	bcd	bd	acd	bd	ad	bc	acd	ad	bcd	bc	bcd	bcd	bcd	bcd
Hi02g06	15	bc	c--	bc	c--	ad	c--	c--	ac-	bd	ac-	ac	c--	ac	bc	ad	ac-	bc	ac-	ad	ac-	ac-	ac-	ac-
Hi04c05	15	ad	acd	ad	acd	bc	acd	bcd	acd	ac	bcd	bd	acd	bd	ad	bc	acd	ad	bcd	bc	bcd	bcd	bcd	bcd
Hi06f09	15	ac	bcd	bc	bcd	ad	bcd	bcd	acd	ad	acd	ac	bcd	ac	bc	ad	acd	ac	acd	ad	acd	acd	acd	acd
NZ02b01	15	ac	acd	ac	bcd	bd	acd	bcd	acd	ad	bcd	bc	acd	bc	ac	bd	acd	ac	bcd	bd	bcd	bcd	bcd	bcd
CH02d10a	16	ac	bd	ac	ad	bcd	bcd	bd	bcd	bd	bcd	ad	bc	bd	ad	bc	bd	ad	bc	ac	bc	bd	ad	bc
CH05b06z_1	16	ad	ad	ac	bc	acd	bcd	ac	acd	ac	acd	bc	bd	ac	bcd	acd	bcd	acd	bcd	acd	bcd	bcd	acd	acd
CH05c06_2	16	ac	ad	ad	ad	bcd	acd	ad	acd	ad	acd	bc	ac	bd	bcd	bcd	acd	acd	bcd	bcd	bcd	bcd	bcd	acd
Hi01c11x	16	bd	bc	bd	ad	bcd	acd	bd	bcd	bc	bcd	ad	ac	bd	acd	bcd	acd	bcd	acd	bcd	acd	acd	bcd	bcd
Hi01d06y	16	ad	ac	ad	bd	acd	bcd	ad	acd	ac	acd	bd	bc	ac	bcd	acd	bcd	acd	bcd	acd	bcd	bcd	acd	acd
CH05a04	16	ee	ee	fg	eg	efg	eeg	eg	eeg	eg	efg	ef	ee	fg	efg	efg	eeg	eeg	eeg	efg	efg	efg	efg	eeg
Hi04e04	16	ef	fg	eg	fg	eeg	efg	fg	efg	fg	eeg	ee	ef	eg	eeg	eeg	efg	efg	efg	eeg	eeg	eeg	eeg	efg
CH04f10	16	lm	lm	lm	ll	llm	lll	lm	llm	lm	llm	ll	ll	lm	lll	llm	lll	llm	lll	llm	lll	lll	llm	llm
CH01h01	17	ad	bd	acd	ad	bc	bcd	ac	bcd	bd	bd	acd	bc	ac	acd	bcd	ac	ad	acd	acd	acd	acd	bcd	bd
CH04c06y_2	17	bd	ac	c--	bc	bd	c--	ac	c--	ac	ac	c--	ad	bd	bd-	c--	bd	bc	bd-	bd-	bd-	bd-	bd-	ac
CH05d08y_1	17	bc	bc	ac-	ac	ad	c--	bc	c--	ad	bc	c--	bd	ad	ac-	c--	ad	ac	ac-	ac-	ac-	ac-	ac-	bc
CH05g03	17	bd	bc	acd	ac	bc	bcd	ad	bcd	ac	bc	acd	bd	ad	acd	acd	ad	ac	acd	acd	acd	acd	bcd	ac
Hi03c05	17	bd	bd	ac-	ad	ac	bc-	bd	bc-	ac	bd	bc-	bc	ac	ac-	bc-	ac	ad	ac-	ac-	ac-	ac-	ac-	bd
Hi07b02_1	17	ad	ad	bcd	bd	ac	acd	ac	acd	bd	ad	bcd	ac	bc	bcd	acd	bc	bd	acd	bcd	bcd	bcd	acd	ad
Hi07b02_2	17	ac	ac	bcd	bc	ad	acd	bd	acd	bc	ac	bcd	ad	bd	bcd	acd	bd	bc	bcd	bcd	bcd	bcd	acd	ac
Hi05c06_1	17	h-	h-	hkk	h-	hk	hhk	h-	hkk	hk	hk	hhk	h-	h-	hkk	hhk	h-	kk	hkk	hhk	hkk	hhk	hkk	hk
CH04c06y_3	17	ll	ll	llm	ll	ll	llm	lm	llm	lm	lm	llm	ll	lm	lll	lll	lm	lm	llm	llm	lll	lll	llm	lm
GD96	17	lm	lm	lll	lm	ll	lll	ll	llm	lm	lm	lll	ll	ll	lll	lll	ll	lm	llm	lll	llm	lll	llm	lm

Note: ‘-‘ represents a null allele, or missing data; ‘p1’ and ‘p2’ are con-dominant alleles.
