# Supplementary material for: Molecular Genetic Features of Polyploidization and Aneuploidization Reveal Unique Patterns for Genome Duplication in Diploid Malus
Source: PLoS One. 2012 Jan 10;7(1):e29449. doi: 10.1371/journal.pone.0029449 (PMC3254611; doi:10.1371/journal.pone.0029449)
Supplement: Table S6 — The distributional features of microsatellite markers in the aneuploid seedlings from the cross of ‘M 27×Fu 2’. (PDF) [file pone.0029449.s007.pdf]

Markers	LG	Aneuploid seedlings from the cross of 'M 27 × Fu 2'																								
		MF01	MF02	MF03	MF04	MF05	MF06	MF07	MF08	MF09	MF10	MF11	MF12	MF13	MF14	MF15	MF16	MF17	MF18	MF19	MF20	MF21	MF22	MF23	MF24	MF25
CH05g08	1	bc	bd	bc	ac	ad	bc	ad	bc	ad	bd	ad	bc	bc	ac	bd	ad	bc	ad	bc	bd	bc	bc	bd	bc	ad
Hi02b10	1	bc	bc	bd	ad	ac	bd	ac	bd	ac	bc	ac	bc	bd	ad	bc	ac	bd	ac	bd	bc	bd	bd	bc	bd	ac
Hi02c07	1	bd	bc	ad	bc	ac	bd	ad	ad	bc	ac	ad	bc	bd	ac	bc	ad	ad	ad	bd	bd	bd	bd	bc	bd	bc
Hi07d08	1	bc	ad	ac	ad	bd	ac	bc	bc	ad	bd	bc	ad	ac	bd	ad	bc	bc	bc	ac	ac	ac	ac	ad	ac	ad
Hi12c02	1	ad	bd	ad	ac	bc	ad	bc	ad	bc	ac	bc	ad	ad	bd	ac	bc	ad	bc	ad	ac	ad	ad	ac	ad	bc
KA4B	1	h-	kk	h-	hh	h-	h-	kk	h-	h-	hh	h-	h-	h-	kk	hh	h-	h-	kk	h-	hh	h-	h-	hh	h-	h-
CH02a04z	2	ac	bd	bc	bc	ac	ac	bc	ac	bc	ac	bc	bcd	bcd	bcd	bcd	bc	bd	acd	ac	ac	ad	acd	ac	acd	acd
CH02c02a_3	2	bc	ad	ac	ad	bd	ac	bc	bc	ad	bd	bc	acd	acd	bcd	acd	bc	bc	bcd	ac	ac	ac	acd	ad	acd	acd
CH02c06	2	ac	bc	ac	ad	bd	bc	ad	bc	ad	ad	ac	bcd	bcd	acd	bcd	bc	bc	acd	ac	ac	ac	acd	bd	bcd	acd
CH03d01	2	ad	bc	bd	bd	ad	ad	bd	ad	ad	ad	bd	bcd	bcd	bcd	bcd	bd	bc	acd	ad	ad	ac	acd	ad	bcd	acd
CH03d10	2	ad	ad	bd	ac	bd	bd	ad	bd	ad	bd	ad	acd	acd	acd	acd	ad	ac	bcd	bd	bd	bc	bcd	bd	bcd	bcd
CH05e03	2	ac	ac	bc	ad	bc	bc	ac	bc	ac	bc	ac	acd	bcd	bcd	bcd	ac	ad	acd	bc	bc	bd	acd	bc	bcd	acd
CN493139	2	bd	ac	ac	ac	bc	bc	bc	bd	ad	ac	ac	bcd	acd	acd	acd	bc	ac	bcd	bc	ad	bd	bcd	bc	acd	bcd
Hi02a07	2	bc	ad	ac	ac	bc	bc	ac	bc	ac	bc	ac	acd	acd	acd	acd	ac	ad	bcd	bc	bc	bd	bcd	bc	acd	bcd
CH02c02a_2	2	lm	lm	lm	ll	lm	lm	lm	lm	lm	ll	ll	llm	lll	lll	lll	lm	lm	llm	lm	ll	lm	llm	ll	lll	llm
Hi05c06_3	2	nn	nn	nn	nn	nn	nn	nn	nn	nn	nn	nn	nnp	nnp	nnp	nnp	nn	np	nnp	nn	nn	np	nnp	nn	np	np
AU223657	3	bd	ad	ac	ad	ad	bd	acd	bcd	bc	bcd	acd	bc	bc	bd	ad	bc	bc	bcd	acd	ac	ad	bcd	bd	bc	bd
CH03e03	3	ad	ad	bd	ac	ad	bd	acd	bcd	bc	bcd	bcd	bc	bc	bd	ad	ac	bc	bcd	acd	ac	ad	bcd	bd	bc	bd
Hi07e08x	3	ac	bd	bc	bc	bc	ac	bcd	acd	ad	acd	acd	ad	ad	ac	bc	bd	ad	acd	bcd	bd	bc	acd	ac	ad	ac
CH03g07	3	fg	fg	ef	fg	fg	eg	eeg	efg	ee	efg	efg	ef	ee	eg	fg	ee	ee	efg	efg	ef	ee	eeg	eg	fg	eg
HGA8bY	3	nn	nn	nn	np	nn	np	np1p2	np1p2	nn	np1p2	np1p2	nn	np	np	nn	np	np	np1p2	np1p2	nn	np	np1p2	np	nn	np
Hi04c10x_1	3	np	nn	np	np	np	nn	np1p2	np1p2	np	np1p2	np1p2	np	nn	nn	np	nn	nn	np1p2	np1p2	np	nn	np1p2	nn	np	nn
CH02c02b	4	bc	ad	ac	ac	bcd	bc	acd	bcd	ac	bcd	acd	ad	ad	acd	acd	acd	acd	bc	bcd	bc	bcd	bd	bcd	acd	bcd
CH05d02	4	bd	bc	bd	ad	acd	ad	bcd	acd	ac	bcd	bcd	ac	ad	bcd	acd	bcd	acd	bc	bcd	bd	acd	ad	bcd	bcd	--
GD162	4	ac	bc	ac	ad	bcd	bc	acd	bcd	bd	acd	acd	bd	bc	acd	bcd	acd	bcd	ad	acd	ac	bcd	bc	acd	acd	bcd
Hi04c10x_3	4	bc	ac	bc	bd	ac-	ad	c--	ac-	ad	c--	c--	ad	ac	c--	ac-	c--	ac-	ad	c--	bc	ac-	ac	c--	c--	c--
Hi07b02_4	4	ac	bc	bd	ad	bcd	bc	acd	bcd	ad	acd	acd	bd	bc	acd	bcd	acd	bcd	ad	acd	ac	bcd	bc	acd	acd	acd
CH04e02	4	ef	ef	ee	eg	efg	fg	eeg	efg	ef	eeg	eeg	fg	fg	eeg	eeg	eeg	eeg	fg	eeg	ee	efg	eg	eeg	eeg	efg
CH02a08z	5	bcd	ac	bcd	ad	bcd	bcd	bcd	bcd	bcd	acd	acd	bc	ad	bcd	ac	bc	bcd	bcd	ad	acd	bcd	bcd	bcd	acd	bc
CH04g09y	5	acd	ad	bcd	ac	acd	acd	acd	bcd	acd	bcd	bcd	bd	ac	acd	ad	bd	acd	acd	ac	bcd	acd	acd	bcd	bcd	bd
Hi11a03	5	acd	ad	acd	bc	acd	acd	bcd	acd	acd	bcd	bcd	bd	bc	acd	bd	ad	acd	acd	bd	bcd	acd	acd	acd	bcd	ad
CH03a04	5	efg	ef	efg	eg	efg	efg	eeg	eeg	efg	eeg	eeg	ee	eg	efg	ee	ef	efg	efg	ee	eeg	efg	efg	efg	eeg	ef
CH03a09	5	efg	fg	efg	eg	efg	efg	eeg	efg	efg	eeg	eeg	ef	ee	efg	fg	fg	efg	efg	eg	eeg	efg	eeg	efg	eeg	ef
CH04e03	5	eeg	eg	eeg	ef	eeg	eeg	efg	efg	eeg	efg	efg	fg	ef	eeg	fg	fg	eeg	eeg	fg	efg	eeg	eeg	eeg	efg	eg
CH04h02_2	5	hhk	hk	hkk	kk	hhk	hkk	hhk	hkk	hhk	hhk	hkk	kk	h-	hkk	kk	hk	hkk	hkk	kk	hhk	hhk	hkk	hkk	hhk	hk

Markers	LG	Aneuploid seedlings from the cross of ‘M 27 × Fu 2’																								
		MF01	MF02	MF03	MF04	MF05	MF06	MF07	MF08	MF09	MF10	MF11	MF12	MF13	MF14	MF15	MF16	MF17	MF18	MF19	MF20	MF21	MF22	MF23	MF24	MF25
CH04h02_4	5	hk-	h-	hk-	kk	hk-	hk-	hk-	hk-	hk-	hk-	hk-	h-	kk	hk-	h-	kk	hk-	hk-	h-	hk-	hk-	hk-	hk-	hk-	kk
CH05e06	5	hk-	k-	hk-	hh	hk-	hk-	hk-	hk-	hk-	hk-	hk-	k-	hh	hk-	k-	hh	hk-	hk-	k-	hk-	hk-	hk-	hk-	hk-	hh
Hi04d02	5	hkk	hh	hhk	hh	hhk	hkk	hhk	hkk	hkk	hhk	hkk	hk	k-	hhk	hh	hk	hhk	hkk	hh	hkk	hkk	hhk	hhk	hhk	hk
Hi21c08	5	hhk	kk	hkk	hk	hkk	hhk	hkk	hkk	hhk	hkk	hhk	hk	h-	hkk	kk	hk	hkk	hhk	kk	hhk	hhk	hkk	hkk	hhk	hk
AJ000761	6	bd	bd	bd	ac	bcd	ac	ac	ac	bd	bd	ac	ad	acd	bcd	bc	ad	acd	bc	bc	acd	acd	ad	bd	ad	bcd
CH03c01	6	bc	bc	bc	ad	acd	ad	ad	ad	bc	bc	ad	ac	acd	acd	bd	ad	bcd	bd	bd	bcd	bcd	ac	bc	ac	acd
CH03d07	6	ac	ac	ac	bd	acd	bd	bd	bd	ac	ac	bd	bc	bcd	acd	ad	bd	bcd	ad	ad	bcd	bcd	bc	ac	bc	acd
CH03d12	6	ef	ee	ee	eg	eeg	fg	fg	fg	ee	ee	eg	ef	efg	eeg	ef	fg	eeg	eg	eg	efg	eeg	eg	ee	ef	eeg
Hi01d05	6	fg	fg	eg	ef	eef	ee	ee	ee	fg	fg	ef	eg	efg	eef	eg	ee	eef	ef	ef	efg	eef	ef	fg	eg	eef
Hi04c10x_2	7	bc	bc	ad	ad	bd	bc	bd	bd	ac	ac	ad	acd	bd	ac	bc	ac	ac	ad	ac	ac	bd	ad	ad	bc	ad
Hi05b09	7	bc	ad	ad	bc	ac	ad	ac	ac	bd	bd	bc	bcd	ac	bd	ad	bd	bd	bc	bd	bd	ac	bc	bc	ad	bc
CH04e05	7	ll	lm	lm	ll	lm	lm	lm	lm	ll	ll	ll	lll	lm	ll	lm	ll	ll	ll	ll	ll	lm	ll	ll	lm	ll
CH05b06z_2	7	nn	np	nn	nn	np	nn	nn	np	nn	nn	np	np1p2	np	nn	np	nn	np	np	nn	np	nn	np	nn	nn	np
CH01c06	8	ac	ad	ad	bd	ac	ad	bd	bc	bc	bc	ac	bc	ac	bc	ac	bd	bc	ac	ad	bc	bc	ac	bc	bc	ac
CH02g09	8	ad	ac	ac	bc	ad	ac	bc	bd	bd	bd	ad	bd	ad	bd	ad	bc	bd	ad	ac	bd	bd	ad	bd	bd	ad
Hi04b12	8	bc	bd	bd	ad	bc	bd	ad	ac	ac	ac	bc	ac	bc	ac	bc	ad	ac	bc	bd	ac	ac	bc	ac	ac	bc
Hi23g12	8	bc	bc	bd	ac	bd	bc	ac	ad	ad	ad	bd	ad	bd	ad	bd	ac	ad	bd	bc	ad	ad	bd	ad	ad	bd
Hi04e05	8	ll	ll	ll	lm	ll	ll	lm	lm	lm	lm	ll	lm	ll	lm	ll	lm	lm	ll	ll	lm	lm	ll	lm	lm	ll
CH01h02_2	9	bc	ad	bd	bcd	acd	acd	ad	ac	acd	ac	ad	bcd	acd	bcd	bcd	acd	ad	acd	bcd	bcd	acd	acd	acd	bcd	acd
CH05d08y_2	9	bc	ad	bc	acd	bcd	bcd	bd	ad	bcd	ad	bd	acd	acd	acd	acd	bcd	bd	bcd	acd	bcd	bcd	bcd	bcd	bcd	bcd
Hi05e07	9	ac	bc	ad	acd	bcd	bcd	bc	bd	bcd	bd	bc	acd	bcd	acd	acd	bcd	bc	bcd	acd	acd	bcd	bcd	bcd	acd	bcd
NH029a	9	ad	bd	ac	bcd	acd	bcd	bd	bc	acd	bc	bd	bcd	acd	bcd	acd	acd	bd	acd	acd	bcd	acd	bcd	acd	bcd	acd
CH01h02_1	9	ee	ee	eg	efg	eef	eef	fg	ef	eef	ef	ef	efg	eef	efg	efg	eef	fg	eef	efg	eef	eef	eef	eef	efg	eef
CH05c07	9	ef	fg	fg	efg	eeg	eeg	ee	eg	eeg	eg	eg	efg	eeg	efg	efg	eeg	ee	eeg	efg	efg	eeg	eeg	eeg	efg	eeg
GD142	9	ef	ee	eg	efg	eeg	eeg	eg	eg	eeg	eg	eg	efg	eeg	efg	efg	eeg	fg	eeg	efg	efg	eeg	eeg	eeg	efg	eeg
Hi01d01	9	nn	np	nn	np1p2	np1p2	np1p2	np	np	np1p2	np	np	np1p2	np1p2	np1p2	np1p2	np1p2	np	np1p2	np1p2	np1p2	np1p2	np1p2	np1p2	np1p2	np1p2
CH01f07a	10	ad	ac	bcd	ac	acd	acd	bc	bd	bcd	bd	bc	acd	acd	bd	bcd	acd	bcd	ac	bcd	bcd	ac	acd	bcd	acd	acd
CH01f12	10	ad	bc	bcd	bd	bcd	acd	ac	bd	bcd	ac	bd	bcd	bcd	bd	acd	bcd	acd	bc	bcd	acd	bc	bcd	acd	bcd	acd
CH02a10	10	bd	bc	acd	bc	bcd	bcd	ad	ac	acd	ac	ad	bcd	bcd	ac	acd	bcd	acd	bd	acd	acd	bd	bcd	acd	bcd	bcd
CH02b03b	10	bc	bd	bcd	bd	bcd	acd	ac	ad	acd	ad	ac	acd	acd	ad	bcd	acd	bcd	bc	acd	bcd	bc	acd	bcd	acd	acd
CH02b07	10	bd	bc	acd	ad	acd	bcd	bc	bd	acd	bc	bd	acd	acd	ad	bcd	acd	bcd	ac	acd	bcd	ac	bcd	acd	acd	bcd
CH02c11	10	ad	ac	bcd	ac	acd	bcd	bd	bc	bcd	bc	bd	acd	acd	bc	bcd	acd	bcd	ad	acd	acd	ad	acd	bcd	acd	bcd
Hi02d04	10	bd	ac	bcd	ad	bcd	acd	ac	ad	bcd	ac	ad	bcd	bcd	bd	acd	bcd	acd	bc	bcd	acd	bc	acd	bcd	bcd	acd

Markers	LG	Aneuploid seedlings from the cross of ‘M 27 × Fu 2’																								
		MF01	MF02	MF03	MF04	MF05	MF06	MF07	MF08	MF09	MF10	MF11	MF12	MF13	MF14	MF15	MF16	MF17	MF18	MF19	MF20	MF21	MF22	MF23	MF24	MF25
Hi04f08	10	bc	bd	acd	ac	acd	bcd	bd	bc	acd	bd	bc	acd	acd	ac	bcd	acd	bcd	ad	acd	bcd	ad	bcd	acd	acd	bcd
MS06g03	10	ad	ad	bcd	ac	acd	acd	bc	bd	bcd	bd	bc	acd	acd	bd	bcd	acd	bcd	ac	bcd	acd	ac	acd	bcd	acd	acd
CH04c06y_1	10	lm	lm	lll	ll	lll	llm	lm	lm	lll	lm	lm	lll	lll	ll	llm	lll	llm	ll	lll	llm	ll	llm	lll	lll	llm
CH03d11	10	np	nn	np1p2	np	np1p2	np1p2	np	nn	np1p2	nn	np	np1p2	np1p2	np	np1p2	np1p2	np1p2	nn	np1p2	np1p2	nn	np1p2	np1p2	nn	nn
MS02a01	10	np	np	np1p2	nn	np1p2	np1p2	nn	np	np1p2	np	nn	np1p2	np1p2	np	np1p2	np1p2	np1p2	nn	np1p2	np1p2	nn	np1p2	np1p2	np1p2	np1p2
CH02d08	11	ac	bd	ac	bd	bd	bd	ac	bcd	ac	ac	bcd	bd	bc	ac	bd	acd	ac	bc	ac	ad	bc	ac	bcd	ac	ad
CH04g07	11	bc	bd	bc	bd	ad	ad	ac	acd	ac	ac	bcd	ad	bc	ac	bd	bcd	ac	ac	bc	bd	ac	bd	acd	ac	bd
Hi06b06	11	ad	bc	bc	bc	ac	ac	ad	acd	ad	ad	bcd	ac	bd	ad	bc	bcd	ad	ad	bc	bc	ad	bc	acd	ad	ac
CH04h02_1	11	np	nn	np	np	nn	nn	np	np1p2	np	np	np1p2	np	np	nn	nn	np1p2	np	np	np	nn	np	np	np1p2	nn	nn
CH04h02_3	11	nn	nn	np	np	nn	nn	np	np	np	np	np	np	np	np	nn	np	np	np	np	nn	np	np	np	nn	nn
CH01b12y	12	bd	bcd	bc	bd	bd	bcd	acd	ac	bcd	acd	ac	ad	bd	ac	acd	bcd	acd	ac	acd	acd	acd	bd	bcd	bcd	acd
CH01g12	12	ad	acd	ac	bd	ac	acd	bcd	bd	acd	bcd	bd	bc	ac	ac	bcd	acd	bcd	bc	bcd	bcd	bcd	ac	acd	acd	bcd
CH05d11	12	bc	bcd	bc	bd	ac	bcd	acd	ad	acd	acd	bd	ac	bc	bc	acd	bcd	acd	ad	acd	acd	bcd	bc	bcd	acd	acd
NZ28f04	12	ac	acd	ad	ac	bd	acd	bcd	bc	bcd	bcd	bc	bd	ad	ad	bcd	acd	bcd	bc	bcd	bcd	acd	ac	acd	bcd	bcd
CH01f02	12	eg	efg	ee	eg	ef	eeg	efg	fg	efg	efg	eg	ef	ee	ee	efg	efg	efg	fg	efg	efg	eeg	eg	eeg	efg	efg
CH03h03z_2	12	np	np1p2	nn	np	np	np1p2	np1p2	nn	np1p2	np1p2	nn	np	np	nn	nn1p2	np1p2	np1p2	nn	np1p2	nn1p2	nn1p2	nn	nn1p2	np1p2	nn1p2
CH05d04	12	np	np1p2	nn	np	nn	np1p2	np1p2	np	np1p2	np1p2	np	nn	np	nn	np1p2	np1p2	np1p2	np	np1p2	np1p2	np1p2	np	np1p2	np1p2	np1p2
CH03a08	13	ad	ac	ac	bc	ad	bd	bc	acd	ad	bcd	acd	bc	ad	acd	bcd	ac	acd	acd	bd	ad	acd	acd	ac	acd	bcd
CH03h03z_1	13	bd	bc	bc	ac	bd	ad	ac	bcd	ad	acd	bcd	ac	bd	bcd	acd	bc	bcd	bcd	ad	bd	bcd	bcd	bc	bcd	acd
CH05f04	13	bc	bc	bc	ad	ad	bc	bd	bcd	ad	acd	bcd	bd	ac	acd	acd	bc	bcd	bcd	ac	ac	acd	bcd	ad	bcd	bcd
CH05h05	13	ad	ad	ad	bc	bc	ad	ac	acd	bc	bcd	acd	ac	bd	bcd	bcd	ad	acd	acd	bd	bd	bcd	acd	bc	acd	acd
GD147	13	bd	bc	bc	ac	bd	ad	ac	bcd	ad	acd	bcd	ac	bd	bcd	acd	bc	bcd	bcd	ad	bd	bcd	bcd	bc	bcd	acd
Hi03e04	13	ad	ad	ac	ad	bd	ad	ad	acd	ad	acd	acd	ad	ad	bcd	acd	ad	bcd	acd	ad	bc	bcd	acd	ad	bcd	bcd
Hi20b03	13	--	bd	bc	ac	bc	ad	bc	bcd	ad	acd	bcd	ac	bd	bcd	acd	bc	bcd	bcd	ad	bd	bcd	bcd	bc	bcd	acd
NH009b	13	ac	ad	ad	bd	ad	bc	ad	acd	bc	bcd	acd	bd	ac	acd	bcd	ad	acd	acd	bc	ac	acd	acd	ad	acd	bcd
AU223486	13	k-	k-	k-	hk	k-	hh	hk	hk-	hh	hk-	hk-	hk	k-	hk-	hk-	k-	hk-	hk-	hh	k-	hk-	hk-	k-	hk-	hk-
Hi05c06_2	13	kk	hk	hk	h-	hk	h-	hk	hk-	h-	hk-	hk-	h-	kk	hk-	hk-	hk	hk-	hk-	h-	kk	hk-	hk-	hk	hk-	hk-
Hi07b02_3	13	lm	lm	ll	ll	lm	lm	lm	llm	ll	lll	lll	lm	lm	lll	lll	lm	llm	llm	ll	ll	llm	llm	lm	llm	lll
CH05c06_1	13	np	np	np	nn	nn	np	np	np	nn	np	np	nn	nn	np	np	np	np	np	nn	nn	np	np	np	np	np
NZ03c01x_2	13	nn	nn	nn	np	nn	nn	np	np1p2	np	np1p2	np1p2	np	nn	np1p2	np1p2	nn	np1p2	np1p2	nn	nn	np1p2	np1p2	np	np1p2	np1p2
CH01g05	14	bc	ad	bcd	acd	bc	bd	bc	bcd	ac	ac	acd	acd	bc	acd	ac	bd	acd	bcd	bcd	bcd	bcd	bcd	bcd	bcd	ad
CH03a02	14	bc	bc	acd	bcd	bd	ad	ac	acd	bc	bc	bcd	bcd	ac	bcd	bd	ad	bcd	acd	acd	acd	acd	acd	acd	bcd	ad
CH03d08	14	bc	bd	acd	bcd	ad	ac	ad	acd	bd	bd	bcd	bcd	ad	bcd	bd	ac	bcd	acd	acd	acd	acd	acd	acd	acd	bc

Markers	LG	Aneuploid seedlings from the cross of ‘M 27 × Fu 2’																								
		MF01	MF02	MF03	MF04	MF05	MF06	MF07	MF08	MF09	MF10	MF11	MF12	MF13	MF14	MF15	MF16	MF17	MF18	MF19	MF20	MF21	MF22	MF23	MF24	MF25
CH05g07z_1	14	ac	ad	acd	acd	bc	ad	bc	bcd	bc	bc	acd	bcd	ac	acd	bc	ad	bcd	bcd	bcd	bcd	acd	acd	acd	bcd	bd
CH05g07z_2	14	kk	h-	hhk	hkk	hk	h-	h-	hkk	h-	h-	hhk	hkk	hk	kk	kk	h-	hkk	hhk	hhk	hkk	hhk	hhk	hkk	hkk	hk
CH02c02a_1	15	bd	ad	bc	acd	ac	bc	ac	ac	acd	bc	ad	acd	bcd	ad	acd	acd	bcd	bcd	acd	acd	acd	bd	ac	bcd	bcd
CH02c09	15	bc	ad	ac	bcd	ac	ac	bc	bc	bcd	ad	bd	acd	acd	bd	bcd	bcd	acd	bcd	acd	bcd	bcd	ad	ac	acd	acd
CH02d11	15	bc	ad	bd	acd	bd	bd	ad	ad	acd	bc	ac	bcd	bcd	ac	acd	acd	bcd	acd	bcd	acd	acd	bc	bd	bcd	bcd
CH03b10	15	ac	bd	ad	bcd	ad	ad	bd	bd	bcd	ac	bc	acd	acd	bc	bcd	bcd	acd	bcd	acd	bcd	bcd	ac	ad	acd	acd
Hi04c05	15	bc	ac	ad	bcd	ad	ad	bd	bd	acd	ac	bc	bcd	bcd	bc	acd	acd	bcd	acd	acd	acd	bcd	ac	ad	bcd	bcd
Hi06f09	15	ac	bc	ad	bcd	bd	bd	bd	bd	bcd	ac	bc	acd	acd	bc	bcd	bcd	acd	acd	bcd	bcd	bcd	bc	bd	acd	acd
NZ02b01	15	bc	ac	bd	acd	bd	bd	ad	ad	acd	bc	ac	bcd	bcd	ac	acd	acd	bcd	acd	acd	acd	acd	bc	bd	bcd	bcd
Hi02g06	15	nn	np	np	nnp	np	np	np	np	nnp	np	nn	nnp	nnp	nn	nnp	nnp	nnp	nnp	nnp	nnp	nnp	np	np	np	np
CH05c06_2	16	ad	ad	ac	ad	bcd	acd	acd	ad	acd	acd	acd	acd	acd	bc	ad	acd	bc	acd	ad	bc	bc	acd	acd	bcd	bcd
Hi01c11x	16	bc	bd	bc	bc	acd	acd	bcd	ac	bcd	bcd	acd	acd	bcd	bd	bd	acd	ad	acd	ac	bd	ad	bcd	bcd	acd	bcd
Hi01d06y	16	ac	ad	ac	ac	bcd	bcd	acd	bc	bcd	acd	bcd	bcd	acd	ad	ad	acd	bd	bcd	bc	ad	bd	acd	acd	bcd	acd
Hi04e04	16	bc	bc	bd	bc	acd	bcd	bcd	bc	bcd	bcd	bcd	bcd	bcd	ad	bc	acd	ad	bcd	ac	bd	ad	bcd	bcd	acd	bcd
CH02d10a	16	ef	ef	fg	fg	efg	eef	efg	fg	eef	efg	eef	eef	eef	eg	ee	eef	eg	eef	ef	fg	eg	eef	eef	efg	efg
CH05a04	16	eg	eg	ee	ee	efg	eeg	efg	ee	eeg	efg	eeg	eeg	eeg	ef	fg	eeg	ef	eeg	eg	ee	ef	eeg	eeg	efg	efg
CH05b06z_1	16	fg	fg	ef	fg	eeg	efg	eeg	ef	efg	eeg	efg	efg	efg	ee	eg	efg	ee	efg	fg	ee	ee	efg	efg	eeg	eeg
CH04f10	16	lm	ll	lm	lm	lll	lll	llm	ll	lll	llm	lll	lll	llm	lm	lm	lll	ll	lll	ll	lm	ll	llm	llm	lll	llm
CH01h01	17	bc	ad	bc	ac	bc	bcd	bcd	ad	bcd	acd	bcd	ac	acd	ac	ad	bcd	bd	bc	acd	bcd	bcd	bcd	bcd	bc	bcd
CH04c06y_2	17	ac	bd	bc	ac	bc	bd-	bd-	ad	c--	c--	c--	ad	bd-	ac	bd	bd-	bd	bc	c--	bd-	bd-	bd-	bd-	ac	bd-
CH05d08y_1	17	bc	ad	ac	bc	ac	ac-	c--	bd	c--	c--	ac-	bd	ac-	bc	ad	ac-	ad	ac	c--	ac-	ac-	ac-	ac-	bc	ac-
CH05g03	17	ac	bd	bc	bc	ac	acd	acd	bd	acd	bcd	acd	bc	bcd	ac	bd	acd	ad	ad	bcd	acd	bcd	acd	acd	ac	acd
Hi03c05	17	ac	bd	ad	bd	ad	ac-	ac-	bc	bc-	bc-	bc-	bc	ac-	bd	ac	ac-	ac	ad	bc-	ac-	ac-	ac-	ac-	bd	ac-
Hi07b02_1	17	bc	ac	ad	bd	ad	acd	acd	bc	acd	bcd	acd	bd	acd	bd	bc	acd	ac	ad	bcd	acd	acd	acd	acd	bd	acd
Hi07b02_2	17	bc	ad	bc	ac	bc	bcd	bcd	ad	bcd	acd	bcd	ac	acd	ac	ad	bcd	bc	bc	acd	bcd	bcd	bcd	bcd	bc	bcd
CH04c06y_3	17	lm	ll	ll	ll	ll	lll	lll	lm	lll	llm	lll	ll	llm	lm	lm	llm	lm	ll	llm	llm	lll	lll	lll	ll	lll
GD96	17	ll	ll	lm	lm	lm	llm	lll	ll	llm	lll	llm	lm	lll	lm	ll	lll	ll	lm	llm	lll	llm	lll	llm	lm	lll
Hi05c06_1	17	np	np	np	nn	nn	np1p2	np1p2	np	np1p2	np1p2	np1p2	nn	np1p2	nn	nn	np1p2	np	np	np1p2	np1p2	np1p2	np1p2	np1p2	np	np1p2

Note: ‘-‘ represents a null allele, or missing data; ‘p1’ and ‘p2’ are con-dominant alleles; ‘M‘ represents for ‘M27’ in the seedling names.
