# Supplementary material for: Molecular Genetic Features of Polyploidization and Aneuploidization Reveal Unique Patterns for Genome Duplication in Diploid Malus
Source: PLoS One. 2012 Jan 10;7(1):e29449. doi: 10.1371/journal.pone.0029449 (PMC3254611; doi:10.1371/journal.pone.0029449)
Supplement: Table S7 — The distributional features of microsatellite markers in the aneuploid seedlings from the cross of ‘CO 2×RO 6’. (PDF) [file pone.0029449.s008.pdf]

Markers	LG	Aneuploid seedlings from the cross of ‘CO 2 × RO 6’																									
		CR01	CR02	CR03	CR04	CR05	CR06	CR07	CR08	CR09	CR10	CR11	CR12	CR13	CR14	CR15	CR16	CR17	CR18	CR19	CR20	CR21	CR22	CR23	CR24	CR25	CR26	CR27
Hi07d08	1	bc	bc	ad	ad	bc	bc	bc	ac	ac	bc	ad	ac	bd	ac	bc	bd	bc	ac	bc	bc	ad	bd	bc	bd	ac	ac	bc
Hi12c02	1	bd	bc	ad	ac	bc	bc	ad	ad	bc	ad	ac	ad	bd	bc	bc	bc	ad	ad	bc	bd	ac	bd	bd	bc	ad	ac	bd
KA4B	1	h-	h-	kk	hk	h-	h-	kk	kk	h-	kk	hk	kk	h-	h-	h-	h-	kk	kk	h-	h-	hk	h-	h-	h-	kk	h-	h-
CH05g08	1	lm	ll	lm	lm	ll	ll	ll	lm	lm	ll	lm	lm	ll	lm	ll	ll	ll	lm	ll	ll	lm	ll	ll	ll	lm	lm	ll
Hi02b10	1	ll	ll	ll	ll	ll	lm	lm	lm	ll	lm	ll	lm	ll	ll	ll	ll	lm	ll	ll	lm	ll	ll	ll	lm	ll	ll	ll
Hi02c07	1	lm	lm	lm	lm	lm	ll	ll	ll	lm	ll	ll	ll	lm	lm	lm	lm	ll	lm	lm	ll	lm	lm	ll	ll	lm	lm	lm
CH02c06	2	ac	ad	ad	bc	acd	bd	bc	bc	ad	bc	bc	bc	bc	bcd	bd	acd	bd	acd	acd	bcd	bc	bcd	ad	acd	acd	bcd	ac
CH03d01	2	ad	ad	bd	ac	bcd	ad	ac	ad	ad	ac	bd	bd	ad	bcd	ad	bcd	ad	acd	bcd	bcd	bc	acd	ac	acd	bcd	acd	ac
CH05e03	2	ac	ad	bc	ad	bcd	ac	ad	ac	ac	ad	bc	bc	ac	bcd	bc	bcd	ac	acd	bcd	bcd	bd	acd	ad	acd	bcd	bcd	ad
CN493139	2	bc	bc	bc	bd	c--	bc	ad	ac	ac	bd	bc	ad	ac	c--	ac	ac-	ad	c--	c--	c--	ad	ac-	ac	c--	ac-	ac-	bc
Hi02a07	2	ee	eg	ee	eg	eeg	ef	fg	ef	ee	eg	ee	fg	fg	eeg	ef	efg	fg	eeg	eeg	eeg	fg	efg	fg	eeg	efg	eeg	ee
CH02a04z	2	h-	h-	kk	h-	hkk	h-	h-	h-	kk	h-	kk	kk	h-	hhk	kk	hhk	h-	hhk	hkk	hhk	hk	hkk	h-	hhk	hkk	hkk	h-
CH02c02a_2	2	lm	lm	lm	lm	llm	ll	ll	ll	ll	lm	lm	ll	ll	llm	ll	lll	ll	llm	llm	llm	ll	lll	ll	llm	lll	llm	lm
CH02c02a_3	2	nn	np	nn	np	np1p2	nn	np	nn	nn	np	nn	np	np	np1p2	nn	np1p2	np	np1p2	np1p2	np1p2	np	np1p2	np	np1p2	np1p2	np1p2	nn
CH03d10	2	nn	np	nn	np	np1p2	nn	np	nn	nn	np	nn	nn	nn	np1p2	nn	np1p2	nn	np1p2	np1p2	np1p2	np	np1p2	np	np1p2	np1p2	np1p2	np
Hi05c06_3	2	nn	np	nn	np	nnp	nn	np	nn	nn	np	nn	nn	nn	nnp	nn	nnp	nn	nnp	nnp	nnp	np	np	np	nnp	nnp	nnp	np
CH03e03	3	bc	ad	bc	ad	ac	ad	ac	ac	ac	acd	acd	bcd	bd	ad	acd	ac	bd	bcd	ad	bd	acd	bcd	bd	acd	acd	acd	bd
CH03g07	3	ad	ac	bc	bc	ad	bc	bd	bd	bd	bcd	bcd	acd	ac	bc	bcd	bc	ac	acd	bc	ac	bcd	acd	ac	bcd	acd	acd	ac
HGA8bY	3	ac	bd	ac	ac	ad	bc	bc	bd	bd	bc-	bc-	ac-	ad	bd	ac-	ac	bc	ac-	bc	ac	bc-	ac-	bc	ac-	bc-	bc-	bc
Hi04c10x_1	3	ac	bc	ac	ac	bd	ac	ad	ad	ad	ac-	ac-	c--	bc	ac	ac-	bc	bc	c--	ac	bc	ac-	ac-	bc	c--	c--	c--	ac
AU223657	3	ee	fg	ee	ef	fg	ef	eg	eg	eg	eeg	eeg	efg	fg	ee	efg	ef	ef	efg	ee	ef	eeg	eeg	ef	efg	efg	efg	ee
Hi07e08x	3	lm	ll	lm	ll	ll	ll	lm	lm	lm	lll	llm	lll	lm	lm	lll	ll	ll	lll	lm	ll	lll	llm	lm	lll	lll	lll	lm
CH02c02b	4	bc	bc	bc	bd	ad	bc	bd	bcd	acd	bcd	acd	acd	ac	acd	ac	ac	bc	bcd	ad	bcd	acd	bd	bcd	bcd	acd	acd	bcd
CH05d02	4	ac	bc	ac	ad	ad	bd	ac	bcd	bcd	bcd	acd	acd	bd	acd	bd	ad	bd	bcd	ac	acd	acd	bc	bcd	acd	bcd	bcd	bcd
GD162	4	ac	ad	ac	ad	ad	bd	ac	bcd	bcd	bcd	acd	acd	bd	acd	bd	ad	bd	bcd	ad	acd	acd	bc	bcd	acd	bcd	bcd	bcd
Hi04c10x_3	4	bc	bc	bc	bd	bd	ad	bc	ac-	c--	ac-	c--	c--	ad	c--	ad	bc	ad	ac-	bc	c--	c--	ac	ac-	c--	ac-	ac-	ac-
Hi07b02_4	4	ac	ac	ac	ac	ad	ad	ac	c--	ac-	c--	ac-	c--	bd	ac-	bd	ac	bd	c--	bc	ac-	c--	bd	c--	ac-	c--	c--	c--
CH04e02	4	ef	eg	ef	eg	eg	eg	ee	fg	fg	ef	eeg	eeg	efg	efg	efg	fg	fg	efg	eg	eeg	efg	ef	eeg	eeg	efg	efg	efg
CH03a09	5	bc	ac	bd	ac	bd	ac	acd	bc	ac	bc	bcd	bcd	bcd	acd	acd	ad	bcd	ac	acd	acd	ad	bc	acd	acd	bcd	ac	acd
Hi04d02	5	ad	ac	bc	bd	ac	ac	ac-	bd	ad	ad	ac-	c--	c--	c--	ac-	ad	c--	ac	c--	c--	bc	bd	ac-	ac-	c--	ad	ac-
Hi11a03	5	ad	ac	bc	bd	ac	ac	ac-	bd	ad	ad	ac-	bc-	bc-	ac-	ac-	ad	ac-	ac	bc-	bc-	bc	ad	ac-	ac-	bc-	ad	ac-
CH03a04	5	ef	fg	ee	ee	fg	fg	efg	ee	ef	ee	efg	eeg	eeg	efg	efg	ef	efg	fg	eeg	eeg	ee	ef	efg	efg	eeg	ef	efg
CH04e03	5	eg	eg	fg	fg	ee	ef	eeg	fg	eg	fg	eeg	efg	efg	eeg	efg	eg	eeg	ee	efg	efg	fg	eg	eeg	eeg	efg	fg	eeg
CH04g09y	5	eg	ef	ee	eg	ef	fg	efg	eg	fg	fg	eeg	eeg	eeg	eeg	efg	fg	eeg	fg	eeg	eeg	ee	eg	efg	efg	eeg	fg	efg

Markers	LG	Aneuploid seedlings from the cross of ‘CO 2 × RO 6’																										
		CR01	CR02	CR03	CR04	CR05	CR06	CR07	CR08	CR09	CR10	CR11	CR12	CR13	CR14	CR15	CR16	CR17	CR18	CR19	CR20	CR21	CR22	CR23	CR24	CR25	CR26	CR27
CH05e06	5	eg	eg	ee	fg	ef	fg	efg	eg	fg	eg	eeg	eeg	eeg	eeg	efg	fg	eeg	fg	eeg	eeg	ee	eg	efg	efg	eeg	fg	efg
Hi21c08	5	eg	eg	ee	eg	ef	fg	efg	eg	fg	fg	eeg	eeg	eeg	eeg	efg	fg	efg	fg	eeg	eeg	ee	eg	efg	efg	eeg	fg	efg
CH04h02_2	5	h-	h-	kk	hk	h-	hk	hhk	kk	h-	kk	hkk	hhk	hhk	hkk	hhk	hk	hkk	hk	hhk	hkk	hk	hk	hkk	hhk	hkk	hk	hhk
CH04h02_4	5	h-	h-	h-	h-	kk	kk	hk-	h-	h-	h-	hk-	hk-	hk-	hk-	hk-	h-	hk-	kk	hk-	hk-	h-	h-	hk-	hk-	hk-	h-	hk-
CH02a08z	5	np	np	np	np	nn	nn	nnp	np	np	np	nnp	nnp	nnp	nnp	nnp	np	nnp	nn	nnp	nnp	np	np	nnp	nnp	nnp	np	nnp
CH03d12	6	bc	ac	bc	bd	bd	ad	bcd	bc	ad	ac	bcd	ac	bc	bcd	bcd	bd	acd	ac	ac	ad	bd	bcd	acd	bcd	acd	acd	bcd
CH03c01	6	fg	eg	ef	ef	ef	eg	eeg	ef	eg	eg	eeg	eg	ef	efg	eeg	ee	eeg	eg	ef	eg	ee	efg	eeg	efg	eeg	eeg	efg
CH03d07	6	ee	eg	ee	fg	ef	eg	efg	ee	fg	fg	efg	ee	ef	efg	efg	ee	efg	ee	ee	eg	fg	eeg	efg	eeg	eeg	efg	efg
Hi01d05	6	ef	eg	ee	fg	fg	eg	efg	ef	fg	fg	efg	ee	ef	efg	efg	ef	efg	ee	ee	eg	fg	efg	eeg	efg	eeg	efg	efg
AJ000761	6	kk	h-	h-	h-	h-	h-	hkk	h-	h-	h-	hhk	h-	kk	hkk	hhk	h-	hkk	h-	h-	h-	h-	hkk	hhk	hkk	hkk	hhk	hhk
Hi04c10x_2	7	bc	bd	bd	bc	acd	ac	ac	ad	ac	ac	bcd	ac	bd	bd	bc	ac	bc	bc	bd	ad	bd	bcd	ac	ac	bd	bd	bcd
Hi05b09	7	ee	eg	ee	fg	eeg	fg	eg	fg	eg	fg	eeg	ef	ef	ee	ee	fg	eg	ee	eg	ef	eg	efg	fg	fg	ee	eg	efg
CH04e05	7	ll	ll	ll	ll	llm	lm	lm	lm	lm	lm	lll	lm	ll	ll	ll	lm	ll	ll	ll	lm	ll	lll	lm	lm	ll	ll	lll
CH05b06z_2	7	np	np	np	nn	np1p2	nn	nn	np	nn	np	np1p2	np	np	nn	nn	np	nn	nn	np	np	nn	np1p2	nn	np	nn	nn	np1p2
CH01c06	8	ac	ad	bc	ac	bd	ac	bc	bc	ac	bd	ad	ac	bd	ac	bc	bc	bc	ad	ad	ac	bd	ac	ac	ad	bc	bc	ac
CH02g09	8	ll	ll	lm	ll	lm	ll	ll	lm	ll	lm	ll	ll	lm	ll	lm	lm	lm	ll	ll	ll	lm	ll	ll	ll	lm	lm	ll
Hi04b12	8	ll	ll	lm	ll	lm	ll	lm	ll	ll	lm	ll	lm	lm	ll	lm	lm	lm	ll	ll	ll	lm	ll	ll	ll	lm	lm	lm
Hi04e05	8	np	nn	nn	nn	np	np	nn	nn	nn	np	np	nn	nn	np	nn	nn	np	np	nn	np	np	nn	nn	np	nn	nn	nn
Hi23g12	8	np	nn	nn	nn	np	nn	nn	nn	nn	np	np	nn	nn	np	nn	nn	np	np	nn	np	np	nn	nn	np	nn	nn	nn
CH01h02_1	9	bc	bcd	bc	ad	bcd	acd	bc	ac	bd	bcd	ac	bcd	acd	ad	bc	bcd	bd	bcd	acd	bcd	bcd	acd	bcd	ad	bcd	bcd	bcd
GD142	9	ad	acd	ad	bc	bcd	acd	bc	bc	ad	bcd	ac	bcd	bcd	bd	ac	bcd	ac	bcd	acd	acd	acd	acd	bcd	ac	acd	acd	acd
Hi01d01	9	bd	bcd	bd	ac	bcd	acd	bd	ad	bc	bcd	ad	bcd	acd	ac	bd	bcd	bc	bcd	acd	bcd	bcd	acd	acd	ac	bcd	bcd	bcd
CH05c07	9	fg	efg	fg	eg	efg	eeg	fg	eg	ef	efg	fg	efg	eeg	ee	fg	efg	ef	efg	eeg	efg	efg	efg	efg	ee	efg	efg	efg
NH029a	9	ef	eg-	fg	eg	efg	eeg	fg	eg	ef	efg	fg	eeg	eeg	ee	fg	eeg	ef	efg	eeg	efg	efg	efg	efg	eg	efg	efg	efg
Hi05e07	9	hh	hk-	hh	k-	hhk	hkk	hh	hk	k-	hhk	hh	hkk	hhk	k-	hh	hkk	k-	hhk	hhk	hkk	hhk	hhk	hkk	k-	hkk	hhk	hkk
CH01h02_2	9	lm	llm	lm	ll	llm	lll	lm	ll	lm	llm	ll	llm	lll	ll	lm	llm	lm	llm	lll	llm	llm	lll	lll	ll	llm	llm	llm
CH05d08y_2	9	ll	lll	ll	lm	lll	llm	ll	lm	ll	lll	ll	lll	lll	lm	ll	lll	ll	lll	llm	lll	llm	llm	lll	lm	lll	lll	lll
CH01f07a	10	a	bc	ac	ad	bcd	bcd	bc	bcd	bcd	bcd	bd	acd	bcd	bc	bd	bcd	bcd	bcd	bcd	bd	acd	bcd	bcd	ac	acd	acd	bcd
CH01f12	10	a	ad	bd	ac	acd	acd	ac	bcd	acd	bcd	ac	bcd	acd	bd	bc	acd	acd	acd	acd	ac	acd	acd	acd	bd	bcd	acd	bcd
CH02b03b	10	a	bc	ac	ad	bcd	bcd	bc	bcd	bcd	bcd	bd	acd	bcd	bc	bd	bcd	bcd	bcd	bcd	bd	acd	bcd	bcd	ac	acd	bcd	bcd
CH02b07	10	b	ac	ad	bc	bcd	bcd	bd	acd	acd	bcd	bc	acd	bcd	bd	ac	bcd	bcd	bcd	bcd	ac	acd	bcd	bcd	ad	bcd	acd	acd
Hi02d04	10	b	ac	ac	bd	bcd	bcd	bc	acd	acd	bcd	bd	acd	bcd	bc	ad	bcd	acd	acd	bcd	ad	acd	bcd	bcd	bc	bcd	acd	acd
MS06g03	10	a	bc	ac	ad	bcd	bcd	bc	bcd	bcd	bcd	ad	acd	bcd	bc	bd	bcd	bcd	bcd	bcd	bd	acd	acd	bcd	bc	acd	bcd	bcd
CH04c06y_1	10	l	ll	ll	lm	llm	llm	lm	lll	lll	llm	lm	lll	llm	lm	ll	llm	llm	lll	llm	lm	lll	llm	llm	ll	llm	lll	lll

Markers	LG	Aneuploid seedlings from the cross of 'CO 2 × RO 6'																										
		CR01	CR02	CR03	CR04	CR05	CR06	CR07	CR08	CR09	CR10	CR11	CR12	CR13	CR14	CR15	CR16	CR17	CR18	CR19	CR20	CR21	CR22	CR23	CR24	CR25	CR26	CR27
CH02a10	10	n	np	np	nn	np1p2	np1p2	nn	np1p2	np1p2	np1p2	nn	np1p2	np1p2	np	nn	np1p2	np1p2	np1p2	np1p2	nn	np1p2	np1p2	np1p21	np	np1p2	np1p2	np1p2
CH02c11	10	n	np	np	nn	np1p2	np1p2	nn	np1p2	np1p2	np1p2	nn	np1p2	np1p2	np	np	np1p2	np1p2	np1p2	np1p2	nn	np1p2	np1p2	np1p2	np	np1p2	np1p2	np1p2
CH03d11	10	n	np	nn	np	np1p2	np1p2	np	np1p2	np1p2	np1p2	np	np1p2	np1p2	nn	np	np1p2	np1p2	np1p2	np1p2	np	np1p2	np1p2	np1p2	nn	np1p2	np1p2	np1p2
Hi04f08	10	n	np	nn	np	nnp	nnp	np	nnp	nnp	nnp	np	nnp	nnp	nn	np	nnp	nnp	nnp	nnp	np	nnp	nnp	nnp	nn	nnp	nnp	nnp
MS02a01	10	n	nn	nn	np	np1p2	np1p2	nn	np1p2	np1p2	np1p2	np	np1p2	np1p2	nn	np	np1p2	np1p2	np1p2	np1p2	np	np1p2	np1p2	np1p2	nn	np1p2	np1p2	np1p2
CH02d08	11	ad	bd	bd	ac	bd	bc	acd	bd	acd	bc	ac	ac	bc	ad	acd	bd	ad	bcd	ad	ad	ac	bcd	ac	bc	acd	ac	ad
CH04g07	11	ad	bd	ac	ad	ad	ac	bcd	bc	bcd	ac	bc	bc	ac	bd	acd	ad	bc	acd	bd	bd	bc	acd	bc	ac	bcd	bc	bc
CH04h02_1	11	ac	ac	ac	bd	ad	bd	c--	ac	ac-	ad	ac	bd	bd	bc	c--	bc	bc	c--	ac	bc	bd	c--	bd	ad	ac-	bd	bc
Hi06b06	11	ac	bc	ad	ac	ac	ad	bcd	bd	bcd	ad	bd	bd	ad	bc	acd	ac	bd	acd	bc	bc	bd	acd	bd	ad	bcd	bd	ad
CH04h02_3	11	nn	nn	nn	np	np	np	nnp	nn	nnp	np	nn	np	np	nn	nnp	nn	nn	nnp	nn	nn	np	nnp	np	np	nnp	np	nn
CH05d04	12	bd	ac	bd	ac-	bc	ac	ac-	bc-	ac	ac	ac	ad	bc-	bd	bc	bd	bc-	bc-	ac-	ac	ac-	bc	bd	bc-	ac-	bc	bd
CH05d11	12	bc	bc	ad	ac-	ac	bc	ac-	bc-	bc	bd	bc	ad	ac-	bd	ac	ad	bc-	ac-	ac-	bc	bc-	ac	ad	ac-	bc-	ac	ad
NZ28f04	12	ad	ad	bc	bcd	bd	ad	acd	bcd	ad	ac	ad	bc	bcd	ac	bd	bc	acd	bcd	bcd	ad	acd	bd	bc	bcd	acd	bd	bc
CH01f02	12	ee	ee	ef	efg	ee	ef	efg	eeg	ee	eg	ee	fg	efg	eg	ef	fg	eeg	efg	efg	ee	eeg	ef	fg	efg	eeg	ef	eg
CH01g12	12	ee	ee	fg	efg	ef	ee	efg	eeg	ee	eg	ee	fg	efg	eg	ef	fg	eeg	efg	efg	ee	eeg	ef	fg	efg	eeg	ef	fg
CH01b12y	12	hk	kk	hh	hk-	kk	hk	hk-	hk-	hk	hk	hk	hk	hk-	hk	kk	kk	hk-	hk-	hk-	hk	hk-	hh	hk	hk-	hk-	hh	kk
CH03h03z_2	12	nn	nn	np	np1p	nn	nn	np1p	np1	nn	np	nn	np	np1	np	nn	np	np1p	np1	np1p	nn	np1	nn	np	np1p	np1p	nn	np
CH03a08	13	ac	bd	bc	ad	bc	acd	bcd	acd	bcd	bc	bd	bcd	ac	ac	bd	acd	acd	acd	bcd	bcd	bcd	ad	bc	ad	bc	bd	bcd
CH03h03z_1	13	bc	ad	ac	bd	ac	c--	ac-	c--	ac-	ac	ad	ac-	bc	bc	ad	c--	c--	c--	ac-	ac-	ac-	bd	ac	bd	ac	ad	ac-
CH05h05	13	ad	ac	ac	ad	ac	acd	acd	acd	acd	ac	bd	acd	ac	bc	ad	acd	bcd	acd	bcd	acd	bcd	ac	ac	ad	ac	bd	acd
Hi05c06_2	13	bc	ad	ad	ad	bc	ac-	ac-	ac-	bc-	ac	bd	c--	bc	bc	bd	c--	ac-	ac-	ac-	ac-	c--	ad	bc	ad	bc	bd	ac-
Hi20b03	13	ac	ac	bd	ac	ad	acd	bcd	acd	bcd	bd	bc	bcd	ad	bd	bc	acd	bcd	acd	bcd	bcd	bcd	ac	bd	ac	bd	bc	bcd
NH009b	13	ac	ac	bd	ac	ad	acd	bcd	acd	bcd	bd	bc	bcd	ad	bd	bc	acd	bcd	acd	bcd	bcd	bcd	ac	bd	ac	bd	bc	bcd
CH05c06_1	13	hk	hk	hk	hk	kk	hk-	hk-	hk-	hk-	hk	hh	hk-	kk	hk	hk	hk-	hk-	hk-	hk-	hk-	hk-	kk	hk	hk	hh	hh	hk-
CH05f04	13	k-	hk	hk	k-	hk	hkk	hhk	hkk	hhk	hk	hh	hkk	k-	k-	hk	hhk	hkk	hkk	hhk	hhk	hkk	k-	hk	k-	hk	hh	hhk
GD147	13	kk	kk	h-	kk	hk	hhk	hkk	hkk	hhk	h-	h-	hhk	hk	hk	h-	hkk	hhk	hhk	hkk	hkk	hhk	kk	h-	kk	hk	h-	hkk
Hi03e04	13	h-	h-	hk	h-	h-	hkk	hhk	hhk	hkk	hk	kk	hkk	h-	h-	kk	hhk	hkk	hkk	hhk	hhk	hkk	h-	hk	h-	hk	kk	hhk
AU223486	13	ll	ll	ll	ll	ll	lll	lll	lll	lll	ll	lm	lll	ll	lm	ll	lll	llm	lll	llm	lll	llm	ll	lm	ll	lm	lm	llm
Hi07b02_3	13	ll	lm	ll	lm	lm	llm	llm	llm	lll	ll	ll	lll	lm	ll	ll	llm	lll	llm	lll	llm	lll	lm	ll	lm	ll	ll	lll
NZ03c01x_2	13	lm	lm	ll	lm	ll	llm	lll	llm	lll	ll	ll	lll	lm	lm	lm	llm	llm	llm	lll	lll	lll	ll	ll	lm	ll	lm	lll
CH01g05	14	bc	bc	ac	ad	bcd	bd	acd	ac	ac	bcd	ad	bd	ac	bd	ad	bcd	bcd	bc	acd	bcd	bd	bcd	bcd	ac	ac	acd	bcd
CH03a02	14	ac	ac	bc	bc	acd	ad	bcd	bc	bc	acd	bc	ac	bc	ad	bd	acd	acd	ac	bcd	acd	ad	acd	acd	bc	bc	bcd	acd
CH03d08	14	bc	bc	ac	ad	bcd	bd	acd	ac	ac	bcd	ad	bd	ac	bd	ad	bcd	bcd	bc	acd	bcd	bd	bcd	bcd	ac	ac	acd	bcd

Markers	LG	Aneuploid seedlings from the cross of 'CO 2 × RO 6'																										
		CR01	CR02	CR03	CR04	CR05	CR06	CR07	CR08	CR09	CR10	CR11	CR12	CR13	CR14	CR15	CR16	CR17	CR18	CR19	CR20	CR21	CR22	CR23	CR24	CR25	CR26	CR27
CH05g07z_1	14	ac	ac	bc	bd	c--	ad	c--	ac	ac	ac-	ad	bd	bc	ad	bd	c--	ac-	bc	c--	ac-	ad	c--	c--	bc	ac	c--	c--
CH05g07z_2	14	n	np	np	nn	np1p2	np1p2	nn	np1p2	np1p2	np1p2	nn	np1p2	np1p2	np	nn	np1p2	np1p2	np1p2	np1p2	nn	np1p2	np1p2	np1p21	np	np1p2	np1p2	np1p2
CH02c02a_1	15	n	np	np	nn	np1p2	np1p2	nn	np1p2	np1p2	np1p2	nn	np1p2	np1p2	np	np	np1p2	np1p2	np1p2	np1p2	nn	np1p2	np1p2	np1p2	np	np1p2	np1p2	np1p2
CH02d11	15	n	np	nn	np	np1p2	np1p2	np	np1p2	np1p2	np1p2	np	np1p2	np1p2	nn	np	np1p2	np1p2	np1p2	np1p2	np	np1p2	np1p2	np1p2	nn	np1p2	np1p2	np1p2
CH03b10	15	n	np	nn	np	nnp	nnp	np	nnp	nnp	nnp	np	nnp	nnp	nn	np	nnp	nnp	nnp	nnp	np	nnp	nnp	nnp	nn	nnp	nnp	nnp
Hi04c05	15	n	nn	nn	np	np1p2	np1p2	nn	np1p2	np1p2	np1p2	np	np1p2	np1p2	nn	np	np1p2	np1p2	np1p2	np1p2	np	np1p2	np1p2	np1p2	nn	np1p2	np1p2	np1p2
Hi06f09	15	ad	bd	bd	ac	bd	bc	acd	bd	acd	bc	ac	ac	bc	ad	acd	bd	ad	bcd	ad	ad	ac	bcd	ac	bc	acd	ac	ad
CH02c09	15	ad	bd	ac	ad	ad	ac	bcd	bc	bcd	ac	bc	bc	ac	bd	acd	ad	bc	acd	bd	bd	bc	acd	bc	ac	bcd	bc	bc
NZ02b01	15	ac	ac	ac	bd	ad	bd	c--	ac	ac-	ad	ac	bd	bd	bc	c--	bc	bc	c--	ac	bc	bd	c--	bd	ad	ac-	bd	bc
Hi02g06	15	ac	bc	ad	ac	ac	ad	bcd	bd	bcd	ad	bd	bd	ad	bc	acd	ac	bd	acd	bc	bc	bd	acd	bd	ad	bcd	bd	ad
CH05a04	16	nn	nn	nn	np	np	np	nnp	nn	nnp	np	nn	np	np	nn	nnp	nn	nn	nnp	nn	nn	np	nnp	np	np	nnp	np	nn
CH05c06_2	16	bd	ac	bd	ac-	bc	ac	ac-	bc-	ac	ac	ac	ad	bc-	bd	bc	bd	bc-	bc-	ac-	ac	ac-	bc	bd	bc-	ac-	bc	bd
Hi01c11x	16	bc	bc	ad	ac-	ac	bc	ac-	bc-	bc	bd	bc	ad	ac-	bd	ac	ad	bc-	ac-	ac-	bc	bc-	ac	ad	ac-	bc-	ac	ad
Hi01d06y	16	ad	ad	bc	bcd	bd	ad	acd	bcd	ad	ac	ad	bc	bcd	ac	bd	bc	acd	bcd	bcd	ad	acd	bd	bc	bcd	acd	bd	bc
Hi04e04	16	ee	ee	ef	efg	ee	ef	efg	eeg	ee	eg	ee	fg	efg	eg	ef	fg	eeg	efg	efg	ee	eeg	ef	fg	efg	eeg	ef	eg
CH02d10a	16	ee	ee	fg	efg	ef	ee	efg	eeg	ee	eg	ee	fg	efg	eg	ef	fg	eeg	efg	efg	ee	eeg	ef	fg	efg	eeg	ef	fg
CH05b06z_1	16	hk	kk	hh	hk-	kk	hk	hk-	hk-	hk	hk	hk	hk	hk-	hk	kk	kk	hk-	hk-	hk-	hk	hk-	hh	hk	hk-	hk-	hh	kk
CH04f10	16	nn	nn	np	np1p2	nn	nn	np1p2	np1p2	nn	np	nn	np	np1p2	np	nn	np	np1p2	np1p2	np1p2	nn	np1p2	nn	np	np1p2	np1p2	nn	np
CH01h01	17	ac	bd	bc	ad	bc	acd	bcd	acd	bcd	bc	bd	bcd	ac	ac	bd	acd	acd	acd	bcd	bcd	bcd	ad	bc	ad	bc	bd	bcd
CH05d08y_1	17	bc	ad	ac	bd	ac	c--	ac-	c--	ac-	ac	ad	ac-	bc	bc	ad	c--	c--	c--	ac-	ac-	ac-	bd	ac	bd	ac	ad	ac-
CH05g03	17	ad	ac	ac	ad	ac	acd	acd	acd	acd	ac	bd	acd	ac	bc	ad	acd	bcd	acd	bcd	acd	bcd	ac	ac	ad	ac	bd	acd
Hi07b02_1	17	bc	ad	ad	ad	bc	ac-	ac-	ac-	bc-	ac	bd	c--	bc	bc	bd	c--	ac-	ac-	ac-	ac-	c--	ad	bc	ad	bc	bd	ac-
CH04c06y_2	17	ac	ac	bd	ac	ad	acd	bcd	acd	bcd	bd	bc	bcd	ad	bd	bc	acd	bcd	acd	bcd	bcd	bcd	ac	bd	ac	bd	bc	bcd
CH04c06y_3	17	ac	ac	bd	ac	ad	acd	bcd	acd	bcd	bd	bc	bcd	ad	bd	bc	acd	bcd	acd	bcd	bcd	bcd	ac	bd	ac	bd	bc	bcd
Hi05c06_1	17	hk	hk	hk	hk	kk	hk-	hk-	hk-	hk-	hk	hh	hk-	kk	hk	hk	hk-	hk-	hk-	hk-	hk-	hk-	kk	hk	hk	hh	hh	hk-
GD96	17	k-	hk	hk	k-	hk	hkk	hhk	hkk	hhk	hk	hh	hkk	k-	k-	hk	hhk	hkk	hkk	hhk	hhk	hkk	k-	hk	k-	hk	hh	hhk
Hi03c05	17	kk	kk	h-	kk	hk	hhk	hkk	hkk	hhk	h-	h-	hhk	hk	hk	h-	hkk	hhk	hhk	hkk	hkk	hhk	kk	h-	kk	hk	h-	hkk
Hi07b02_2	17	h-	h-	hk	h-	h-	hkk	hhk	hhk	hkk	hk	kk	hkk	h-	h-	kk	hhk	hkk	hkk	hhk	hhk	hkk	h-	hk	h-	hk	kk	hhk

Note: '-' represents a null allele, or missing data; 'p1' and 'p2' are con-dominant alleles
