# Supplementary material for: Molecular Genetic Features of Polyploidization and Aneuploidization Reveal Unique Patterns for Genome Duplication in Diploid Malus
Source: PLoS One. 2012 Jan 10;7(1):e29449. doi: 10.1371/journal.pone.0029449 (PMC3254611; doi:10.1371/journal.pone.0029449)
Supplement: Table S8 — The distributional features of microsatellite markers in the triploid seedlings from the crosses of ‘Gala×Fuji’ and ‘M 26×Fu 2’. (PDF) [file pone.0029449.s009.pdf]

Markers	LG	Triploid seedlings from the crosses of ‘Gala × Fuji’ and ‘M 26 × Fu 2’																		
		Gala × Fuji															M 26 × Fu 2			
		GF41	GF42	GF43	GF44	GF45	GF46	GF47	GF48	GF49	GF50	GF51	GF52	GF53	GF54	GF55	M26F24	M26F25	M26F26	M26F27
CH05g08	1	abd	abc	abd	abd	abc	abc	abd	abd	abd	abd	abc	abd	abd	abc	abd	abc	abd	abd	abc
Hi02b10	1	nnn	nnn	nnp	nnp	nnn	nnp	nnp	nnp	nnp	nnp	nnn	nnn	nnn	nnn	nnp	abd	abc	abc	abd
Hi02c07	1	lm1m2	lm1m2	lm1m2	lm1m2	lm1m2	lm1m2	lm1m2	lm1m2	lm1m2	lm1m2	lm1m2	lm1m2	lm1m2	lm1m2	lm1m2	hhk	hhk	hhk	hkk
Hi07d08	1	abc	abc	abc	abc	abd	abd	abc	abc	abd	abc	abd	abc	abc	abd	abc	abd	abd	abd	abd
Hi12c02	1	abd	abd	abd	abd	abd	abd	abd	abc	abc	abd	abd	abd	abd	abd	abd	abc	abc	abc	abc
KA4B	1	hkk	hkk	hhk	hkk	hkk	hhk	hhk	hhk	hhk	hhk	hkk	hkk	hhk	hkk	hhk	abd	abc	abc	abd
CH02a04z	2	abd	abc	abd	abc	abd	abd	abd	abd	abd	abc	abd	abc	abd	abd	abc	abc	abd	abc	abd
CH02c02a_2	2	lm1m2	lm1m2	lm1m2	lm1m2	lm1m2	lm1m2	lm1m2	lm1m2	lm1m2	lm1m2	lm1m2	lm1m2	lm1m2	lm1m2	lm1m2	llm	lll	lll	llm
CH02c02a_3	2	abc	abc	abc	abc	abd	abd	abc	abc	abd	abc	abd	abc	abc	abd	abc	nnp	nnp	nnn	nnn
CH02c06	2	abd	abc	abc	abc	abd	abc	abc	abc	abc	abd	abc	abc	abc	abd	abc	abd	abd	abc	abd
CH03d01	2	abd	abc	abd	abc	abc	abd	abd	abd	abd	abc	abd	abc	abd	abd	abc	abc	abd	abc	abd
CH03d10	2	lm1m2	lm1m2	lm1m2	lm1m2	lm1m2	lm1m2	lm1m2	lm1m2	lm1m2	lm1m2	lm1m2	lm1m2	lm1m2	lm1m2	lm1m2	hhk	hkk	hkk	hhk
CH05e03	2	abd	abc	abd	abd	abc	abd	abc	abd	abd	abc	abd	abc	abd	abd	abc	abd	abc	abd	abc
CN493139	2	abc	abd	abc	abc	abd	abc	abc	abc	abc	abd	abc	abc	abc	abd	abd	abd	abd	abd	abc
Hi02a07	2	abc	abd	abc	abd	abd	abc	abc	abc	abc	abd	abc	abd	abc	abc	abd	abd	abc	abd	abc
Hi05c06_3	2	nnn	nnp	nnn	nnp	nnp	nnn	nnn	nnn	nnn	nnp	nnn	nnp	nnn	nnp	nnp	nnp	nnn	nnp	nnn
AU223657	3	lm1m2	lm1m2	lm1m2	lm1m2	lm1m2	lm1m2	lm1m2	lm1m2	lm1m2	lm1m2	lm1m2	lm1m2	lm1m2	lm1m2	lm1m2	abd	abc	abc	abc
CH03e03	3	abd	abd	abc	abd	abc	abc	abd	abd	abd	abc	abc	abc	abc	abc	abc	abd	abc	abc	abc
CH03g07	3	abc	abc	abd	abd	abc	abc	abc	abc	abc	abd	abd	abc	abd	abc	abd	efg	efg	eef	eef
HGA8bY	3	abd	abc	abc	abc	abc	abd	abc	abc	abd	abc	abc	abc	abc	abd	abc	nnn	nnp	nnn	nnp
Hi04c10x_1	3	abd	abd	abc	abc	abc	abc	abc	abd	abd	abc	abc	abc	abc	abc	abc	nnn	nnp	nnn	nnp
Hi07e08x	3	abc	abc	abd	abd	abd	abd	abc	abc	abc	abd	abd	abd	abd	abd	abd	abc	abd	abd	abd
CH02c02b	4	abd	abc	abd	abc	abc	abd	abd	abd	abd	abc	abd	abc	abd	abd	abc	abd	abc	abd	abc
CH04e02	4	efg	efg	eef	eef	eef	eef	efg	eef	eef	eef	efg	efg	eef	efg	eef	abd	abd	abc	abd
CH05d02	4	abc	abc	abc	abd	abd	abd	abc	abd	abd	abd	abd	abc	abd	abc	abc	eef	efg	eef	eef
GD162	4	abd	abd	abc	abc	abc	abc	abd	abc	abc	abc	abd	abd	abc	abd	abd	abc	abc	abd	abc
Hi04c10x_3	4	abc	abc	abc	abd	abd	abd	abc	abd	abc	abd	abc	abd	abd	abc	abd	abc	abc	abd	abc
Hi07b02_4	4	eef	eef	eef	efg	efg	efg	eef	efg	efg	efg	eef	efg	efg	eef	efg	efg	eef	efg	efg
CH02a08z	5	nnn	nnp	nnn	nnp	nnp	nnn	nnp	nnp	nnp	nnn	nnp	nnp	nnp	nnn	nnp	nnp	nnn	nnp	nnp
CH03a04	5	abd	abc	abd	abc	abd	abc	abc	abd	abd	abd	abc	abd	abc	abd	abc	abc	abc	abc	abd
CH03a09	5	eef	efg	eef	efg	eef	efg	efg	eef	efg	eef	efg	efg	efg	eef	efg	abd	abd	abd	abc
CH04e03	5	eef	efg	eef	efg	efg	eef	efg	efg	efg	eef	eef	efg	efg	eef	efg	efg	eef	efg	efg
CH04g09y	5	abd	abc	abd	abc	abd	abd	abc	abc	abd	abc	abc	abc	abc	abd	abc	abc	abc	abc	abd

Markers	LG	Triploid seedlings from the crosses of ‘Gala × Fuji’ and ‘M 26 × Fu 2’																		
		Gala × Fuji															M 26 × Fu 2			
		GF41	GF42	GF43	GF44	GF45	GF46	GF47	GF48	GF49	GF50	GF51	GF52	GF53	GF54	GF55	M26F24	M26F25	M26F26	M26F27
CH04h02_2	5	hk-	hk-	hk-	hk-	hk-	hk-	hk-	hk-	hk-	hk-	hk-	hk-	hk-	hk-	hk-	hkk	hhk	hkk	hkk
CH04h02_4	5	hk-	hk-	hk-	hk-	hk-	hk-	hk-	hk-	hk-	hk-	hk-	hk-	hk-	hk-	hk-	hk-	hk-	hk-	hk-
CH05e06	5	abc	abd	abc	abd	abd	abc	abd	abd	abd	abc	abd	abd	abd	abc	abd	abd	abd	abd	abc
Hi04d02	5	hkk	hkk	hhk	hkk	hkk	hkk	hkk	hhk	hkk	hhk	hkk	hkk	hkk	hhk	hhk	abc	abc	abc	abd
Hi11a03	5	hk-	hk-	hk-	hk-	hk-	hk-	hk-	hk-	hk-	hk-	hk-	hk-	hk-	hk-	hk-	abd	abd	abd	abc
Hi21c08	5	hk-	hk-	hk-	hk-	hk-	hk-	hk-	hk-	hk-	hk-	hk-	hk-	hk-	hk-	hk-	hkk	hhk	hkk	hkk
AJ000761	6	hk-	hk-	hk-	hk-	hk-	hk-	hk-	hk-	hk-	hk-	hk-	hk-	hk-	hk-	hk-	abd	abc	abc	abc
CH03c01	6	hk-	hk-	hk-	hk-	hk-	hk-	hk-	hk-	hk-	hk-	hk-	hk-	hk-	hk-	hk-	eef	efg	eef	efg
CH03d07	6	abc	abd	abc	abd	abc	abd	abc	abc	abd	abc	abc	abd	abc	abd	abd	abc	abd	abd	abd
CH03d12	6	abd	abd	abd	abc	abc	abc	abd	abd	abc	abd	abc	abc	abd	abd	abc	abd	abc	abc	abc
Hi01d05	6	efg	efg	efg	eef	eef	eef	efg	efg	eef	efg	eef	eef	efg	efg	eef	efg	eef	eef	eef
CH04e05	7	lm1m2	lm1m2	lm1m2	lm1m2	lm1m2	lm1m2	lm1m2	lm1m2	lm1m2	lm1m2	lm1m2	lm1m2	lm1m2	lm1m2	lm1m2	lll	lll	lll	llm
CH05b06z_2	7	eef	efg	eef	eef	efg	eef	efg	eef	efg	eef	efg	eef	eef	efg	eef	nnp	nnn	nnp	nnn
Hi04c10x_2	7	abd	abc	abd	abd	abc	abd	abd	abc	abc	abd	abc	abd	abd	abc	abd	abd	abc	abd	abc
Hi05b09	7	llm	llm	llm	llm	llm	llm	llm	llm	llm	llm	llm	llm	llm	llm	llm	abc	abd	abc	abd
CH01c06	8	abd	abd	abc	abc	abc	abc	abc	abc	abc	abc	abd	abc	abd	abc	abc	abd	abc	abc	abc
CH02g09	8	lm1m2	lm1m2	lm1m2	lm1m2	lm1m2	lm1m2	lm1m2	lm1m2	lm1m2	lm1m2	lm1m2	lm1m2	lm1m2	lm1m2	lm1m2	abc	abd	abd	abd
Hi04b12	8	abc	abd	abd	abd	abd	abd	abd	abd	abd	abd	abc	abd	abc	abd	abd	abd	abc	abc	abc
Hi04e05	8	llm	llm	llm	llm	llm	llm	llm	llm	llm	llm	llm	llm	llm	llm	llm	lll	lll	lll	lll
Hi23g12	8	nnp	nnn	nnp	nnp	nnp	nnn	nnn	nnn	nnp	nnn	nnp	nnp	nnp	nnp	nnp	abc	abd	abd	abd
CH01h02_1	9	hhk	hhk	hhk	hhk	hhk	hkk	hhk	hhk	hkk	hhk	hhk	hkk	hkk	hhk	hkk	abd	abc	abc	abc
CH01h02_2	9	abd	abd	abd	abc	abc	abc	abd	abc	abc	abd	abd	abc	abd	abd	abc	abc	abd	abd	abd
CH05c07	9	abd	abc	abc	abd	abc	abd	abd	abc	abd	abd	abd	abd	abd	abd	abd	abc	abd	abd	abd
CH05d08y_2	9	nnp	nnp	nnn	nnp	nnp	nnp	nnn	nnn	nnn	nnp	nnn	nnn	nnp	nnn	nnp	efg	efg	eef	eef
GD142	9	abc	abc	abc	abc	abc	abc	abc	abd	abd	abc	abc	abc	abc	abc	abd	abc	abc	abd	abd
Hi01d01	9	lm1m2	lm1m2	lm1m2	lm1m2	lm1m2	lm1m2	lm1m2	lm1m2	lm1m2	lm1m2	lm1m2	lm1m2	lm1m2	lm1m2	lm1m2	efg	efg	eef	eef
Hi05e07	9	abc	abc	abc	abc	abd	abd	abc	abd	abd	abc	abc	abd	abd	abc	abd	abd	abc	abc	abc
NH029a	9	abd	abd	abd	abd	abd	abc	abd	abd	abc	abd	abd	abc	abc	abd	abc	abc	abd	abd	abd
CH01f07a	10	abc	abd	abd	abc	abc	abd	abd	abd	abd	abd	abc	abd	abc	abd	abc	abd	abd	abc	abc
CH01f12	10	abd	abc	abc	abc	abd	abd	abd	abc	abd	abd	abc	abd	abc	abd	abd	abc	abc	abc	abc
CH02a10	10	lm1m2	lm1m2	lm1m2	lm1m2	lm1m2	lm1m2	lm1m2	lm1m2	lm1m2	lm1m2	lm1m2	lm1m2	lm1m2	lm1m2	lm1m2	abc	abc	abd	abd
CH02b03b	10	abc	abc	abd	abd	abd	abc	abc	abd	abc	abc	abd	abc	abc	abc	abd	abd	abd	abc	abc
CH02b07	10	abc	abc	abd	abd	abc	abd	abc	abd	abc	abc	abc	abc	abd	abc	abd	abd	abc	abc	abc

Markers	LG	Triploid seedlings from the crosses of 'Gala × Fuji' and 'M 26 × Fu 2'																		
		Gala × Fuji															M 26 × Fu 2			
		GF41	GF42	GF43	GF44	GF45	GF46	GF47	GF48	GF49	GF50	GF51	GF52	GF53	GF54	GF55	M26F24	M26F25	M26F26	M26F27
CH02c11	10	abd	abd	abd	abc	abc	abd	abd	abc	abd	abd	abc	abd	abc	abd	abc	nnp	nnp	nnn	nnn
CH03d11	10	lm1m2	lm1m2	lm1m2	lm1m2	lm1m2	lm1m2	lm1m2	lm1m2	lm1m2	lm1m2	lm1m2	lm1m2	lm1m2	lm1m2	lm1m2	abd	abc	abd	abd
CH04c06y_1	10	lm1m2	lm1m2	lm1m2	lm1m2	lm1m2	lm1m2	lm1m2	lm1m2	lm1m2	lm1m2	lm1m2	lm1m2	lm1m2	lm1m2	lm1m2	llm	llm	llm	llm
Hi02d04	10	llm	llm	llm	llm	llm	llm	llm	llm	llm	llm	llm	llm	llm	llm	llm	abc	abd	abd	abd
Hi04f08	10	nnn	nnp	nnp	nnp	nnn	nnn	nnn	nnp	nnn	nnn	nnp	nnn	nnp	nnn	nnn	abd	abc	abc	abc
MS02a01	10	nnn	nnn	nnn	nnp	nnp	nnn	nnn	nnp	nnn	nnn	nnp	nnn	nnp	nnn	nnp	nnp	nnp	nnn	nnn
MS06g03	10	abd	abc	abc	abd	abd	abd	abc	abd	abc	abc	abd	abc	abd	abc	abd	abd	abd	abc	abc
CH02d08	11	abd	abd	abd	abd	abd	abc	abd	abc	abc	abd	abd	abc	abd	abc	abd	abd	abd	abc	abd
CH04g07	11	abd	abd	abd	abd	abd	abc	abd	abc	abc	abd	abd	abc	abd	abc	abd	abd	abd	abd	abd
CH04h02_1	11	abc	abc	abc	abc	abd	abd	abd	abd	abd	abc	abd	abd	abc	abd	abc	nnp	nnp	nnn	nnp
CH04h02_3	11	nnn	nnn	nnn	nnn	nnp	nnp	nnp	nnn	nnp	nnn	nnp	nnp	nnn	nnp	nnn	nnn	nnn	nnp	nnn
Hi06b06	11	abd	abc	abc	abc	abc	abd	abc	abd	abd	abc	abc	abd	abc	abd	abc	abc	abc	abc	abc
CH01b12y	12	abd	abd	abc	abd	abd	abd	abc	abc	abc	abd	abd	abc	abd	abc	abd	abc	abd	abc	abd
CH01f02	12	eef	efg	eef	efg	efg	efg	eef	efg	eef	efg	efg	efg	efg	eef	efg	hk-	hk-	hk-	hk-
CH01g12	12	abc	abc	abc	abc	abc	abc	abd	abd	abd	abc	abc	abd	abc	abd	abd	abd	abc	abd	abc
CH03h03z_2	12	nnp	nnn	nnn	nnn	nnn	nnn	nnp	nnn	nnp	nnn	nnn	nnp	nnn	nnp	nnp	nnp	nnp	nnn	nnp
CH05d04	12	efg	eef	eef	eef	eef	efg	efg	eef	efg	eef	eef	efg	eef	eef	efg	abc	abd	abc	abd
CH05d11	12	efg	eef	eef	eef	eef	eef	efg	eef	efg	eef	eef	eef	eef	efg	efg	abc	abd	abc	abd
NZ28f04	12	abc	abd	abd	abd	abd	abd	abc	abd	abc	abd	abd	abc	abd	abc	abc	abd	abc	abd	abc
AU223486	13	hk-	hk-	hk-	hk-	hk-	hk-	hk-	hk-	hk-	hk-	hk-	hk-	hk-	hk-	hk-	hhk	hhk	hkk	hhk
CH03a08	13	abc	abd	abd	abd	abc	abc	abc	abd	abc	abc	abd	abd	abc	abd	abc	abd	abd	abc	abc
CH03h03z_1	13	abc	abd	abd	abd	abc	abc	abc	abd	abc	abc	abd	abd	abc	abd	abc	hhk	hhk	hkk	hhk
CH05c06_1	13	abd	abd	abd	abd	abd	abd	abc	abd	abc	abd	abc	abd	abc	abc	abd	abd	abd	abd	abd
CH05f04	13	abc	abd	abc	abc	abc	abc	abd	abd	abd	abc	abd	abc	abc	abd	abc	abc	abc	abc	abc
CH05h05	13	abd	abc	abd	abd	abd	abd	abc	abc	abc	abc	abc	abd	abd	abc	abd	abd	abd	abd	abd
GD147	13	hkk	hkk	hhk	hkk	hhk	hkk	hhk	hkk	hhk	hhk	hhk	hkk	hkk	hhk	hhk	abc	abd	abc	abc
Hi03e04	13	abc	abc	abd	abd	abd	abc	abc	abc	abd	abc	abc	abc	abc	abc	abd	abd	abc	abc	abc
Hi05c06_2	13	hkk	hkk	hhk	hkk	hhk	hkk	hkk	hkk	hhk	hhk	hkk	hkk	hkk	hhk	hkk	hkk	hhk	hkk	hhk
Hi07b02_3	13	llm	llm	llm	llm	llm	llm	llm	llm	llm	llm	llm	llm	llm	llm	llm	lll	llm	llm	lll
Hi20b03	13	abc	abd	abd	--	abc	abc	abc	--	abd	abc	abd	abc	abc	abd	abc	abc	abd	abc	abc
NH009b	13	abd	abc	abc	abc	abd	abd	abd	abc	abd	abd	abc	abd	abd	abc	abd	abd	abc	abd	abd
NZ03c01x_2	13	llm	llm	llm	llm	llm	llm	llm	llm	llm	llm	llm	llm	llm	llm	llm	nnn	nnn	nnp	nnp

Markers	LG	Triploid seedlings from the crosses of 'Gala × Fuji' and 'M 26 × Fu 2'																		
		Gala × Fuji															M 26 × Fu 2			
		GF41	GF42	GF43	GF44	GF45	GF46	GF47	GF48	GF49	GF50	GF51	GF52	GF53	GF54	GF55	M26F24	M26F25	M26F26	M26F27
CH01g05	14	abc	abd	abc	abd	abd	abd	abc	abd	abc	abd	abc	abc	abd	abd	abd	hkk	hhk	hkk	hhk
CH03a02	14	abd	abd	abc	abc	abc	abc	abd	abc	abd	abc	abd	abc	abc	abd	abc	abc	abd	abc	abd
CH03d08	14	abd	abc	abc	abc	abc	abc	abd	abc	abd	abd	abd	abd	abc	abc	abc	abd	abc	abd	abd
CH05g07z_1	14	hhk	hkk	hkk	hkk	hkk	hkk	hhk	hkk	hhk	hkk	hkk	hhk	hkk	hhk	hhk	hkk	hhk	hkk	hhk
CH05g07z_2	14	hhk	hkk	hkk	hkk	hkk	hkk	hhk	hkk	hhk	hkk	hkk	hhk	hkk	hhk	hhk	hkk	hhk	hkk	hhk
CH02c02a_1	15	abc	abc	abc	abc	abd	abc	abc	abd	abc	abd	abc	abc	abc	abc	abd	abd	abc	abd	abc
CH02c09	15	lm1m2	lm1m2	lm1m2	lm1m2	lm1m2	lm1m2	lm1m2	lm1m2	lm1m2	lm1m2	lm1m2	lm1m2	lm1m2	lm1m2	lm1m2	abc	abd	abc	abd
CH02d11	15	abc	abd	abc	abc	abc	abc	abd	abc	abc	abc	abc	abd	abc	abc	abd	abd	abc	abd	abd
CH03b10	15	abd	abd	abd	abd	abd	abd	abd	abd	abd	abd	abd	abc	abd	abd	abd	abc	abd	abc	abc
Hi02g06	15	nnp	nnp	nnn	nnn	nnp	nnn	nnp	nnp	nnp	nnn	nnp	nnp	nnn	nnp	nnn	abd	abd	abd	abd
Hi04c05	15	abd	abd	abd	abd	abd	abd	abc	abc	abd	abd	abc	abd	abd	abd	abd	abc	abc	abc	abc
Hi06f09	15	abd	abd	abd	abd	abd	abd	abc	abd	abd	abd	abd	abc	abd	abd	abd	abd	abd	abd	abd
NZ02b01	15	abd	abc	abd	abd	abd	abd	abc	abc	abd	abd	abd	abd	abd	abd	abd	abd	abd	abd	abd
CH02d10a	16	abc	abc	abc	abc	abc	abc	abd	abd	abd	abc	abc	abd	abc	abd	abd	abc	abd	abc	abd
CH04f10	16	abd	abd	abc	abc	abc	abc	abd	abc	abd	abd	abc	abd	abd	abd	abd	llm	lll	llm	llm
CH05a04	16	abc	abc	abd	abd	abd	abd	abc	abd	abc	abc	abc	abc	abc	abc	abc	efg	eef	eef	eef
CH05b06z_1	16	efg	eef	efg	eef	eef	efg	efg	efg	eef	efg	efg	eef	efg	efg	eef	abc	abc	abc	abc
CH05c06_2	16	abc	abc	abd	abd	abd	abc	abc	abc	abd	abc	abc	abc	abc	abc	abd	abd	abc	abc	abc
Hi01c11x	16	eef	eef	eef	efg	efg	eef	efg	eef	efg	eef	eef	eef	eef	eef	efg	abd	abd	abd	abd
Hi01d06y	16	abd	abd	abc	abc	abc	abc	abd	abc	abd	abd	abc	abd	abd	abd	abd	abd	abd	abd	abd
Hi04e04	16	abd	abd	abc	abc	abc	abc	abd	abc	abc	abd	abd	abd	abd	abd	abd	efg	eef	eef	eef
CH01h01	17	abd	abc	abc	abd	abc	abd	abd	abc	abd	abd	abd	abd	abd	abd	abd	abd	abc	abc	abc
CH04c06y_2	17	abc	abd	abc	abd	abd	abd	abd	abc	abd	abc	abd	abd	abc	abd	abc	abd	abc	abc	abc
CH04c06y_3	17	lm1m2	lm1m2	lm1m2	lm1m2	lm1m2	lm1m2	lm1m2	lm1m2	lm1m2	lm1m2	lm1m2	lm1m2	lm1m2	lm1m2	lm1m2	lm1m2	lm1m2	lm1m2	lm1m2
CH05d08y_1	17	abc	abc	abc	abd	abd	abd	abd	abc	abd	abc	abc	abd	abc	abd	abc	abc	abd	abd	abd
CH05g03	17	abd	abc	abc	abd	abc	abd	abd	abc	abd	abd	abd	abd	abd	abd	abd	abc	abc	abd	abc
GD96	17	lm1m2	lm1m2	lm1m2	lm1m2	lm1m2	lm1m2	lm1m2	lm1m2	lm1m2	lm1m2	lm1m2	lm1m2	lm1m2	lm1m2	lm1m2	llm	lll	llm	lll
Hi03c05	17	llm	llm	llm	llm	llm	llm	llm	llm	llm	llm	llm	llm	llm	llm	llm	abd	abc	abc	abc
Hi05c06_1	17	llm	llm	llm	llm	llm	llm	llm	llm	llm	llm	llm	llm	llm	llm	llm	hhk	hhk	hkk	hhk
Hi07b02_1	17	llm	llm	llm	llm	llm	llm	llm	llm	llm	llm	llm	llm	llm	llm	llm	abd	abc	abc	abc
Hi07b02_2	17	nnn	nnp	nnn	nnn	nnp	nnn	nnn	nnp	nnp	nnn	nnn	nnn	nnn	nnp	nnn	abc	abd	abd	abd

Note: '-' represents a null allele, or missing data; 'p1' and 'p2' , 'm1' and 'm2'are con-dominant alleles, respectively.
