# Supplementary material for: Molecular Genetic Features of Polyploidization and Aneuploidization Reveal Unique Patterns for Genome Duplication in Diploid Malus
Source: PLoS One. 2012 Jan 10;7(1):e29449. doi: 10.1371/journal.pone.0029449 (PMC3254611; doi:10.1371/journal.pone.0029449)
Supplement: Table S9 — The distributional features of microsatellite markers in the triploid seedlings from the crosses of ‘Fuji×Gala’ and ‘M 27×Fu 2’. (PDF) [file pone.0029449.s010.pdf]

Markers	LG	Triploid seedlings from the crosses of 'Fuji × Gala' and 'M 27 × Fu 2'																		
		Fuji × Gala																M 27 × Fu 2		
		FG38	FG39	FG40	FG41	FG42	FG43	FG44	FG45	FG46	FG47	FG48	FG49	FG50	FG51	FG52	FG53	M27F26	M27F27	M27F28
CH05g08	1	abd	abc	abd	abd	abc	abd	abd	abd	abc	abc	abd	abc	abd	abd	abc	abc	abc	abc	abd
Hi02b10	1	llm	---	llm	llm	llm	lll	lll	llm	llm	llm	llm	llm	lll	lll	lll	lll	abd	abd	abc
Hi02c07	1	nnp	nnn	nnp	nnp	nnp	nnn	nnn	nnp	nnn	nnp	nnp	nnp	nnn	nnn	nnn	nnn	abd	abd	abc
Hi07d08	1	abc	abd	abd	abc	abd	abc	abc	abc	abd	abd	abc	abd	abd	abc	abd	abd	abc	abc	abd
Hi12c02	1	abd	abc	abd	abd	abc	abc	abc	abd	abd	abc	abd	abc	abc	abd	abc	abd	abd	abd	abc
KA4B	1	hkk	hkk	hkk	hkk	hkk	hhk	hhk	hkk	hkk	hkk	hhk	hkk	hhk	hkk	hhk	hhk	hkk	hhk	hhk
CH02a04z	2	abc	abd	abc	abc	abd	abc	abc	abd	abd	abd	abd	abd	abc	abd	abc	abd	abd	abc	abd
CH02c02a_2	2	nnn	nnn	nnn	nnn	nnn	nnn	nnp	nnp	nnp	nnp	nnp	nnn	nnn	nnp	nnn	nnn	llm	lll	llm
CH02c02a_3	2	abc	abd	abd	abc	abd	abc	abc	abc	abd	abd	abc	abd	abd	abc	abd	abd	abc	abc	abd
CH02c06	2	abc	abc	abd	abc	abd	abd	abc	abc	abd	abc	abd	abd	abc	abc	abd	abc	abd	abc	abd
CH03d01	2	abd	abd	abc	abd	abd	abc	abd	abd	abd	abd	abd	abd	abd	abd	abc	abd	abc	abd	abc
CH03d10	2	nnn	nnp	nnp	nnp	nnp	nnp	nnn	nnn	nnn	nnp	nnn	nnp	nnp	nnn	nnp	nnp	abc	abd	abc
CH05e03	2	abc	abd	abc	abd	abd	abc	abd	abd	abd	abd	abc	abd	abc	abd	abc	abd	abd	abc	abd
CN493139	2	abc	abc	abc	abd	abc	abd	abc	abc	abc	abc	abd	abd	abd	abc	abd	abc	abd	abc	abd
Hi02a07	2	abc	abc	abd	abc	abc	abd	abc	abc	abc	abc	abc	abc	abc	abc	abd	abc	abd	abc	abd
Hi05c06_3	2	llm	llm	lll	llm	llm	lll	llm	llm	llm	llm	llm	llm	llm	llm	lll	llm	nnp	nnn	nnp
AU223657	3	nnp	nnp	nnp	nnn	nnn	nnp	nnp	nnp	nnn	nnn	nnp	nnn	nnp	nnp	nnp	nnn	abc	abc	abd
CH03e03	3	abc	abd	abd	abc	abc	abd	abd	abc	abd	abc	abd	abd	abd	abd	abc	abd	abc	abc	abd
CH03g07	3	abc	abc	abc	abc	abd	abc	abc	abd	abc	abd	abc	abd	abc	abd	abd	abd	eef	eef	eef
HGA8bY	3	abc	abc	abd	abc	abc	abd	abd	abc	abd	abd	abc	abc	abc	abc	abc	abd	nnn	nnp	nnp
Hi04c10x_1	3	abd	abd	abd	abc	abc	abd	abc	abc	abd	abd	abd	abc	abc	abd	abc	abc	nnp	nnn	nnn
Hi07e08x	3	abc	abd	abd	abc	abc	abd	abd	abc	abd	abc	abd	abd	abd	abd	abc	abd	abd	abd	abc
CH02c02b	4	abc	abc	abd	abc	abc	abd	abc	abc	abc	abc	abc	abc	abc	abc	abd	abc	abd	abc	abd
CH04e02	4	eef	eef	efg	eef	eef	efg	eef	eef	efg	efg	efg	efg	eef	eef	efg	eef	efg	eef	eef
CH05d02	4	abc	abc	abd	abc	abd	abd	abc	abd	abc	abd	abd	abd	abc	abc	abd	abc	abc	abd	abd
GD162	4	abc	abd	abc	abd	abd	abc	abd	abc	abd	abc	abc	abc	abd	abd	abc	abd	abd	abc	abc
Hi04c10x_3	4	abd	abd	abc	abd	abc	abc	abd	abc	abd	abc	abc	abc	abd	abd	abc	abd	abd	abc	abd
Hi07b02_4	4	eef	efg	eef	efg	efg	eef	efg	efg	efg	eef	eef	eef	efg	efg	eef	efg	abd	abc	abd
CH02a08z	5	lm1m2	lm1m2	lm1m2	lm1m2	lm1m2	lm1m2	lm1m2	lm1m2	lm1m2	lm1m2	lm1m2	lm1m2	lm1m2	lm1m2	lm1m2	lm1m2	abd	abc	abc
CH03a04	5	abc	abc	abc	abc	abd	abd	abc	abc	abc	abc	abc	abc	abc	abc	abd	abc	eef	efg	efg
CH03a09	5	efg	efg	efg	efg	eef	eef	efg	efg	eef	efg	efg	efg	efg	efg	eef	efg	efg	eef	efg
CH04e03	5	efg	eef	efg	efg	efg	efg	efg	efg	eef	efg	efg	efg	eef	efg	eef	eef	efg	eef	eef
CH04g09y	5	abd	abc	abd	abd	abc	abd	abd	abd	abc	abc	abd	abc	abd	abd	abc	abc	abc	abd	abd

Markers	LG	Triploid seedlings from the crosses of ‘Fuji × Gala’ and ‘M 27 × Fu 2’																		
		Fuji × Gala																M 27 × Fu 2		
		FG38	FG39	FG40	FG41	FG42	FG43	FG44	FG45	FG46	FG47	FG48	FG49	FG50	FG51	FG52	FG53	M27F26	M27F27	M27F28
CH04h02_2	5	abc	abd	abc	abd	abc	abc	abc	abc	abd	abc	abd	abc	abd	abc	abd	abd	hkk	hhk	hhk
CH04h02_4	5	hkk	hkk	hkk	hkk	hkk	hhk	hhk	hkk	hhk	hkk	hkk	hkk	hkk	hkk	hkk	hkk	hk-	hk-	hk-
CH05e06	5	hk-	hk-	hk-	hk-	hk-	hk-	hk-	hk-	hk-	hk-	hk-	hk-	hk-	hk-	hk-	hk-	hk-	hk-	hk-
Hi04d02	5	abd	abc	abd	abd	abd	abd	abd	abd	abc	abd	abd	abd	abc	abd	abc	abc	hkk	hhk	hhk
Hi11a03	5	hkk	hkk	hkk	hkk	hkk	hhk	hhk	hkk	hhk	hkk	hkk	hkk	hkk	hkk	hkk	hkk	abd	abc	abc
Hi21c08	5	hkk	hkk	hkk	hkk	hkk	hhk	hhk	hkk	hhk	hkk	hkk	hkk	hkk	hkk	hkk	hkk	hkk	hhk	hhk
AJ000761	6	hkk	hkk	hkk	hkk	hkk	hhk	hhk	hkk	hhk	hkk	hkk	hkk	hkk	hkk	hkk	hkk	abc	abd	abd
CH03c01	6	hk-	hk-	hk-	hk-	hk-	hk-	hk-	hk-	hk-	hk-	hk-	hk-	hk-	hk-	hk-	hk-	abd	abc	abc
CH03d07	6	hk-	hk-	hk-	hk-	hk-	hk-	hk-	hk-	hk-	hk-	hk-	hk-	hk-	hk-	hk-	hk-	abc	abd	abd
CH03d12	6	abc	abc	abc	abc	abc	abd	abc	abd	abd	abc	abd	abc	abc	abd	abd	abd	efg	eef	efg
Hi01d05	6	abc	abd	abd	abd	abd	abc	abd	abc	abc	abd	abd	abd	abd	abc	abc	abc	eef	efg	eef
CH04e05	7	eef	efg	efg	efg	efg	eef	efg	eef	eef	efg	efg	efg	efg	eef	eef	eef	lm	ll	lm
CH05b06z_2	7	nnn	nnn	nnp	nnn	nnp	nnn	nnn	nnp	nnp	nnn	nnp	nnp	nnp	nnp	nnp	nnp	nnp	nnn	nnn
Hi04c10x_2	7	eef	efg	eef	eef	eef	efg	eef	efg	efg	efg	eef	eef	eef	eef	efg	eef	abc	abc	abc
Hi05b09	7	abd	abc	abd	abd	abd	abd	abc	abd	abc	abc	abc	abd	abd	abc	abd	abd	abd	abd	abd
CH01c06	8	nnp	nnp	nnn	nnn	nnn	nnp	nnp	nnn	nnn	nnp	nnn	nnn	nnn	nnn	nnn	nnp	abc	abc	abc
CH02g09	8	abd	abd	abd	abd	abc	abc	abc	abc	abc	abd	abc	abc	abc	abc	abc	abc	abd	abd	abd
Hi04b12	8	nnn	nnn	nnp	nnn	nnp	nnp	nnn	nnp	nnp	nnp	nnn	nnp	nnp	nnp	nnn	nnn	abc	abc	abc
Hi04e05	8	abc	abc	abc	abc	abd	abd	abd	abd	abd	abc	abd	abd	abd	abd	abd	abd	lm	ll	lm
Hi23g12	8	nnn	nnn	nnp	nnn	nnp	nnn	nnp	nnn	nnp	nnn	nnn	nnp	nnn	nnn	nnp	nnp	abd	abd	abd
CH01h02_1	9	llm	lll	llm	lll	llm	llm	llm	lll	lll	lll	lll	llm	lll	lll	llm	llm	efg	efg	efg
CH01h02_2	9	hhk	hkk	hhk	hhk	hhk	hhk	hkk	hkk	hkk	hhk	hhk	hkk	hhk	hkk	hhk	hkk	abc	abd	abd
CH05c07	9	abd	abd	abd	abd	abd	abc	abc	abc	abc	abd	abd	abc	abd	abd	abd	abc	eef	eef	eef
CH05d08y_2	9	abd	abc	abd	abd	abc	abc	abc	abd	abc	abc	abd	abc	abd	abd	abd	abd	abd	abc	abc
GD142	9	lm1m2	lm1m2	lm1m2	lm1m2	lm1m2	lm1m2	lm1m2	lm1m2	lm1m2	lm1m2	lm1m2	lm1m2	lm1m2	lm1m2	lm1m2	lm1m2	efg	efg	efg
Hi01d01	9	abd	abd	abc	abc	abc	abd	abd	abd	abd	abc	abc	abd	abd	abd	abc	abc	nnp	nnp	nnp
Hi05e07	9	nnn	nnn	nnn	nnp	nnp	nnn	nnp	nnp	nnn	nnn	nnn	nnp	nnn	nnp	nnp	nnp	abd	abc	abc
NH029a	9	abc	abc	abc	abc	abc	abd	abd	abc	abd	abc	abc	abd	abc	abd	abc	abd	abc	abd	abd
CH01f07a	10	abd	abd	abd	abd	abd	abc	abc	abc	abc	abd	abd	abc	abd	abc	abd	abc	abc	abc	abd
CH01f12	10	abc	abd	abc	abd	abc	abd	abc	abd	abc	abd	abc	abd	abc	abd	abd	abc	abc	abc	abc
CH02a10	10	abc	abd	abc	abd	abd	abd	abc	abc	abc	abd	abd	abc	abd	abd	abd	abd	abd	abd	abc
CH02b03b	10	nnn	nnn	nnn	nnp	nnn	nnp	nnn	nnp	nnn	nnp	nnp	nnn	nnn	nnn	nnn	nnp	abc	abc	abd
CH02b07	10	abd	abd	abc	abd	abc	abd	abc	abd	abc	abd	abc	abd	abc	abd	abd	abd	abc	abc	abc

Markers	LG	Triploid seedlings from the crosses of 'Fuji × Gala' and 'M 27 × Fu 2'																		
		Fuji × Gala																M 27 × Fu 2		
		FG38	FG39	FG40	FG41	FG42	FG43	FG44	FG45	FG46	FG47	FG48	FG49	FG50	FG51	FG52	FG53	M27F26	M27F27	M27F28
CH02c11	10	abc	abc	abd	abc	abd	abc	abd	abc	abd	abc	abd	abc	abd	abc	abc	abc	abd	abd	abc
CH03d11	10	nnp	nnp	nnp	nnp	nnp	nnn	nnp	nnn	nnp	nnn	nnn	nnp	nnp	nnn	nnp	nnn	nnp	nnp	nnn
CH04c06y_1	10	nnn	nnn	nnn	nnn	nnn	nnp	nnn	nnn	nnn	nnp	nnp	nnn	nnp	nnp	nnn	nnp	llm	lll	lll
Hi02d04	10	nnn	nnn	nnp	nnn	nnn	nnn	nnp	nnn	nnp	nnp	nnp	nnp	nnn	nnp	nnn	nnp	abc	abc	abc
Hi04f08	10	llm	lll	lll	lll	lll	lll	llm	lll	llm	lll	lll	llm	lll	lll	lll	lll	abd	abd	abd
MS02a01	10	lll	llm	lll	llm	lll	llm	llm	llm	lll	llm	lll	llm	lll	llm	llm	llm	nnn	nnn	nnp
MS06g03	10	abd	abc	abd	abc	abd	abc	abc	abc	abc	abc	abd	abc	abd	abd	abc	abc	abd	abd	abc
CH02d08	11	abc	abd	abc	abc	abd	abd	abc	abd	abd	abd	abc	abc	abd	abd	abc	abc	abd	abd	abc
CH04g07	11	abc	abd	abd	abd	abd	abd	abc	abd	abc	abc	abd	abc	abd	abd	abc	abc	abd	abd	abc
CH04h02_1	11	abd	abd	abd	abd	abc	abc	abd	abc	abc	abc	abd	abd	abc	abc	abd	abd	nnp	nnp	nnn
CH04h02_3	11	lll	lll	lll	lll	llm	llm	lll	llm	llm	llm	lll	lll	llm	llm	lll	lll	nnn	nnn	nnp
Hi06b06	11	abd	abc	abc	abc	abc	abc	abd	abc	abd	abd	abc	abd	abc	abc	abd	abd	abc	abc	abd
CH01b12y	12	abc	abc	abd	abc	abd	abc	abc	abc	abd	abc	abd	abd	abc	abc	abd	abc	abd	abd	abc
CH01f02	12	eef	eef	efg	eef	efg	eef	eef	eef	efg	eef	efg	efg	eef	eef	efg	efg	efg	eef	eef
CH01g12	12	abd	abd	abc	abc	abc	abd	abc	abd	abc	abd	abc	abc	abd	abd	abc	abc	abc	abc	abd
CH03h03z_2	12	lll	lll	llm	llm	llm	lll	lll	lll	llm	lll	llm	llm	lll	lll	lll	llm	nnp	nnp	nnp
CH05d04	12	efg	efg	eef	eef	efg	efg	efg	efg	eef	efg	eef	eef	eef	efg	efg	eef	nnn	nnn	nnn
CH05d11	12	eef	eef	efg	efg	efg	eef	eef	eef	efg	eef	efg	efg	eef	eef	eef	efg	abc	abc	abc
NZ28f04	12	abc	abc	abd	abd	abd	abc	abc	abc	abd	abc	abd	abd	abc	abc	abc	abd	abd	abd	abd
AU223486	13	hhk	hhk	hhk	hhk	hkk	hhk	hkk	hhk	hkk	hkk	hhk	hkk	hhk	hkk	hkk	hkk	hhk	hhk	hhk
CH03a08	13	abc	abc	abc	abd	abc	abc	abc	abc	abc	abd	abc	abd	abc	abc	abc	abc	abd	abd	abd
CH03h03z_1	13	abd	abc	abc	abd	abc	abc	abc	abc	abc	abd	abc	abd	abc	abc	abc	abc	abd	abd	abd
CH05c06_1	13	abd	abd	abc	abd	abc	abd	abc	abd	abd	abc	abd	abc	abc	abd	abc	abd	nnp	nnn	nnp
CH05f04	13	abd	abc	abd	abc	abd	abc	abc	abc	abc	abd	abc	abd	abc	abc	abc	abc	abc	abd	abc
CH05h05	13	abc	abd	abd	abd	abc	abd	abc	abc	abd	abc	abd	abc	abc	abd	abd	abd	abd	abc	abd
GD147	13	hkk	hhk	hhk	hhk	hkk	hkk	hkk	hhk	hhk	hkk	hkk	hkk	hhk	hkk	hkk	hkk	abd	abd	abd
Hi03e04	13	abd	abd	abd	abc	abc	abd	abc	abc	abc	abc	abc	abd	abd	abd	abc	abc	abd	abd	abc
Hi05c06_2	13	hkk	hhk	hhk	hhk	hkk	hkk	hkk	hhk	hhk	hkk	hkk	hkk	hhk	hkk	hkk	hkk	hhk	hhk	hkk
Hi07b02_3	13	nnp	nnp	nnp	nnp	nnn	nnn	nnn	nnp	nnp	nnn	nnn	nnn	nnp	nnn	nnp	nnn	ll	ll	lm
Hi20b03	13	abd	abc	abc	abd	abc	abc	abc	abc	abc	abd	abc	abd	abc	abd	abc	abc	abd	abd	abd
NH009b	13	abd	abd	abd	abc	abd	abd	abd	abd	abd	abc	abd	abc	abd	abd	abd	abd	abc	abc	abc
NZ03c01x_2	13	nnp	nnp	nnn	nnn	nnp	nnp	nnn	nnp	nnn	nnn	nnp	nnn	nnp	nnn	nnp	nnn	nnp	nnn	nnp

Markers	LG	Triploid seedlings from the crosses of ‘Fuji × Gala’ and ‘M 27 × Fu 2’																		
		Fuji × Gala																M 27 × Fu 2		
		FG38	FG39	FG40	FG41	FG42	FG43	FG44	FG45	FG46	FG47	FG48	FG49	FG50	FG51	FG52	FG53	M27F26	M27F27	M27F28
CH01g05	14	abc	abd	abd	abd	abd	abd	abc	abc	abd	abc	abc	abc	abd	abc	abd	abd	abc	abc	abd
CH03a02	14	abd	abd	abc	abc	abd	abd	abd	abd	abc	abd	abd	abd	abc	abd	abc	abc	abc	abd	abc
CH03d08	14	abd	abd	abc	abc	abc	abc	abd	abd	abc	abd	abd	abd	abc	abc	abc	abc	abd	abc	abd
CH05g07z_1	14	hhk	hhk	hkk	hkk	hkk	hhk	hhk	hhk	hkk	hhk	hkk	hkk	hhk	hhk	hhk	hkk	hk-	hk-	hk-
CH05g07z_2	14	hhk	hhk	hkk	hkk	hkk	hhk	hhk	hhk	hkk	hhk	hkk	hkk	hhk	hhk	hhk	hkk	abd	abc	abd
CH02c02a_1	15	abd	abc	abd	abc	abd	abc	abd	abc	abd	abc	abd	abd	abc	abc	abc	abc	abc	abc	abd
CH02c09	15	nnn	nnn	nnp	nnn	nnp	nnn	nnn	nnp	nnn	nnp	nnn	nnn	nnp	nnn	nnp	nnn	abd	abd	abc
CH02d11	15	abc	abc	abd	abc	abd	abc	abd	abc	abd	abc	abc	abd	abc	abd	abc	abc	abd	abd	abc
CH03b10	15	abd	abc	abd	abd	abd	abc	abd	abc	abd	abc	abc	abd	abc	abc	abd	abc	nnp	nnn	nnn
Hi02g06	15	lm1m2	lm1m2	lm1m2	lm1m2	lm1m2	lm1m2	lm1m2	lm1m2	lm1m2	lm1m2	lm1m2	lm1m2	lm1m2	lm1m2	lm1m2	lm1m2	abd	abc	abd
Hi04c05	15	abd	abd	abc	abd	abc	abc	abc	abd	abc	abd	abd	abc	abd	abc	abd	abd	abd	abd	abc
Hi06f09	15	abc	abd	abc	abd	abc	abd	abc	abd	abc	abd	abd	abc	abd	abd	abd	abd	abc	abd	abc
NZ02b01	15	abc	abd	abc	abc	abc	abd	abc	abd	abc	abd	abd	abc	abd	abd	abd	abd	eef	efg	efg
CH02d10a	16	abd	abd	abc	abc	abc	abd	abd	abd	abc	abd	abc	abc	abd	abd	abc	abc	ll	ll	lm
CH04f10	16	abc	abc	abc	abc	abd	abc	abd	abd	abd	abd	abc	abd	abc	abd	abd	abc	efg	eef	eef
CH05a04	16	abd	abd	abd	abc	abc	abd	abc	abc	abc	abc	abd	abc	abd	abd	abc	abd	efg	eef	eef
CH05b06z_1	16	eef	eef	eef	efg	efg	eef	efg	eef	efg	efg	efg	eef	eef	efg	eef	efg	abd	abd	abc
CH05c06_2	16	abd	abd	abd	abc	abc	abd	abc	abc	abc	abc	abc	abd	abd	abd	abc	abc	abc	abc	abd
Hi01c11x	16	efg	efg	efg	eef	eef	efg	eef	eef	eef	eef	efg	efg	efg	efg	efg	eef	abc	abc	abd
Hi01d06y	16	abc	abc	abc	abc	abd	abc	abd	abd	abd	abd	abc	abd	abc	abd	abd	abc	abc	abc	abd
Hi04e04	16	abc	abc	abc	abd	abd	abc	abd	abd	abd	abd	abd	abc	abc	abc	abd	abc	abc	abd	abd
CH01h01	17	abd	abc	abd	abd	abc	abc	abc	abd	abc	abc	abd	abc	abd	abd	abd	abd	abc	abd	abd
CH04c06y_2	17	abd	abc	abc	abd	abd	abd	abc	abd	abd	abd	abc	abd	abc	abd	abc	abd	lm1m2	lm1m2	lm1m2
CH04c06y_3	17	nnp	nnn	nnn	nnn	nnp	nnn	nnn	nnp	nnp	nnp	nnp	nnp	nnn	nnp	nnp	nnn	abc	abd	abd
CH05d08y_1	17	abc	abc	abc	abd	abd	abd	abc	abd	abd	abd	abc	abd	abc	abd	abd	abd	abd	abc	abc
CH05g03	17	abd	abc	abd	abd	abc	abc	abd	abc	abc	abc	abd	abc	abd	abd	abd	abd	ll	lm	lm
GD96	17	nnn	nnn	nnp	nnn	nnn	nnn	nnp	nnn	nnp	nnn	nnp	nnp	nnn	nnp	nnn	nnp	abc	abd	abd
Hi03c05	17	nnp	nnp	nnn	nnp	nnp	nnp	nnn	nnp	nnn	nnn	nnn	nnn	nnp	nnp	nnp	nnn	nnp	nnn	nnp
Hi05c06_1	17	nnp	nnp	nnp	nnp	nnp	nnn	nnn	nnn	nnp	nnn	nnn	nnn	nnn	nnn	nnp	nnn	abc	abd	abd
Hi07b02_1	17	nnn	nnn	nnn	nnn	nnn	nnn	nnn	nnp	nnn	nnp	nnp	nnp	nnp	nnp	nnp	nnn	abd	abc	abc
Hi07b02_2	17	lll	llm	llm	llm	lll	llm	lll	llm	lll	lll	llm	lll	llm	llm	llm	llm	abc	abc	abd

Note: ‘-‘ represents a null allele, or missing data; ‘p1’ and ‘p2’ , ‘m1’ and ‘m2’are con-dominant alleles, respectively.
