# Supplementary material for: Molecular Genetic Features of Polyploidization and Aneuploidization Reveal Unique Patterns for Genome Duplication in Diploid Malus
Source: PLoS One. 2012 Jan 10;7(1):e29449. doi: 10.1371/journal.pone.0029449 (PMC3254611; doi:10.1371/journal.pone.0029449)
Supplement: Table S10 — The distributional features of microsatellite markers in the triploid seedlings from three crosses. (PDF) [file pone.0029449.s011.pdf]

Markers	LG	Triploid seedlings from three crosses																				
		Fuji × Pink Lady									Pink Lady × Fuji										CO 2 × RO 6	
		FP27	FP28	FP29	FP30	FP31	FP32	FP33	FP34	FP35	**PF25**	**PF26**	**PF27**	**PF28**	**PF29**	**PF30**	**PF31**	**PF32**	**PF33**	**PF34**	CR28	CR29
CH05g08	1	abc	abd	abd	abd	abc	abd	abc	abd	abc	abc	abc	abd	abd	abc	abd	abc	abd	abc	abd	lm1m2	lm1m2
Hi02b10	1	llm	llm	lll	lll	llm	lll	lll	lll	lll	nnn	nnp	nnp	nnp	nnn	--	nnp	nnn	nnn	nnn	lm1m2	lm1m2
Hi02c07	1	nnn	nnp	nnn	nnn	nnp	nnn	nnn	nnn	nnn	lll	llm	llm	llm	lll	lll	llm	lll	lll	lll	llm	llm
Hi07d08	1	abc	abd	abd	abd	abc	abc	abd	abc	abd	abc	abc	abd	abd	abc	abc	abd	abc	abc	abd	abc	abd
Hi12c02	1	abd	abd	abc	abc	abd	abc	abc	abc	abd	abd	abc	abc	abc	abd	abc	abd	abc	abd	abc	abc	abc
KA4B	1	hhk	hkk	hkk	hkk	hkk	hhk	hhk	hhk	hkk	hkk	hhk	hkk	hhk	hkk	hkk	hkk	hhk	hhk	hhk	hk-	hk-
CH02a04z	2	abc	abc	abc	abc	abd	abc	abd	abc	abd	abd	abc	abc	abd	abd	abc	abd	abc	abc	abd	hk-	hk-
CH02c02a_2	2	nnn	nnp	nnp	nnn	nnp	nnn	nnp	nnn	nnp	lll	llm	llm	lll	llm	lll	llm	llm	lll	llm	llllm	llllm
CH02c02a_3	2	abc	abd	abd	abd	abc	abc	abd	abc	abd	abc	abc	abd	abd	abc	abc	abc	abc	abc	abd	nnn	nnn
CH02c06	2	abd	abc	abd	abc	abd	abd	abc	abd	abc	abd	abd	abc	abd	abc	abc	abc	abd	abc	abd	abd	abd
CH03d01	2	abd	abc	abc	abc	abd	abc	abd	abd	abc	abd	abc	abc	abd	abc	abc	abd	abc	abc	abd	abd	abd
CH03d10	2	nnn	nnp	nnp	nnp	nnn	nnp	nnn	nnn	nnp	llm	lll	lll	llm	lll	lll	llm	lll	lll	llm	nnn	nnn
CH05e03	2	abc	abd	abd	abd	abc	abd	abc	abc	abd	abc	abd	abd	abc	abd	abc	abc	abd	abd	abc	abc	abc
CN493139	2	abc	abc	abd	abc	abc	abc	abd	abc	abc	abd	abd	abc	abd	abd	abd	abc	abc	abd	abc	abc	abd
Hi02a07	2	hhk	hhk	hkk	hhk	hkk	hhk	hkk	hkk	hkk	hkk	hkk	hhk	hhk	hhk	hkk	hhk	hhk	hkk	hhk	eef	eef
Hi05c06_3	2	llm	lll	lll	lll	llm	lll	llm	llm	lll	nnn	nnn	nnp	nnn	nnp	nnp	nnn	nnp	nnp	nnn	nnn	nnn
AU223657	3	nnp	nnp	nnn	nnn	nnp	nnn	nnp	nnp	nnn	lm1m2	lm1m2	lm1m2	lm1m2	lm1m2	lm1m2	lm1m2	lm1m2	lm1m2	lm1m2	eef	eef
CH03e03	3	abc	abd	abc	abd	abc	abc	abd	abc	abd	abd	abc	abd	abc	abd	abc	abc	abc	abc	abd	abc	abd
CH03g07	3	abd	abd	abd	abd	abc	abc	abd	abc	abd	abd	abd	abd	abc	abc	abc	abc	abc	abc	abd	abd	abc
HGA8bY	3	abd	abd	abd	abc	abc	abc	abd	abc	abc	abd	abd	abd	abc	abc	abd	abc	abc	abd	abc	abc	abc
Hi04c10x_1	3	abd	abd	abd	abc	abc	abc	abd	abc	abc	abd	abd	abd	abc	abc	abd	abc	abc	abc	abd	abd	abc
Hi07e08x	3	abc	abc	abd	abc	abd	abd	abc	abd	abd	abc	abd	abc	abd	abc	abd	abc	abd	abd	abc	lm1m2	lm1m2
CH02c02b	4	abc	abd	abd	abd	abc	abd	abc	abc	abd	abc	abd	abd	abc	abd	abd	abc	abd	abd	abc	abc	abc
CH04e02	4	efg	eef	eef	eef	efg	efg	efg	eef	efg	eef	efg	eef	eef	efg	eef	efg	efg	eef	efg	efg	eef
CH05d02	4	abd	abc	abc	abc	abd	abd	abc	abd	abc	abc	abd	abc	abd	abc	abc	abc	abd	abc	abd	abd	abd
GD162	4	abd	abc	abc	abc	abd	abd	abc	abd	abc	abc	abd	abc	abd	abc	abc	abc	abd	abc	abd	abd	abd
Hi04c10x_3	4	abd	abc	abc	abc	abd	abd	abc	abd	abc	abc	abd	abc	abd	abc	abc	abc	abd	abc	abd	abd	abd
Hi07b02_4	4	abd	abc	abc	abc	abd	abd	abc	abd	abc	abc	abd	abc	abd	abc	abd	abc	abd	abc	abd	abd	abd
CH02a08z	5	lm1m2	lm1m2	lm1m2	lm1m2	lm1m2	lm1m2	lm1m2	lm1m2	lm1m2	nnp	nnn	nnn	nnn	nnn	nnn	nnn	nnp	nnn	nnn	nnp	nnp
CH03a04	5	abd	abd	abd	abd	abc	abd	abd	abd	abc	abc	abd	abc	abc	abd	abc	abc	abc	abc	abd	eef	eef
CH03a09	5	abd	abc	abc	abc	abd	abc	abc	abd	abd	abc	abc	abd	abd	abd	abd	abd	abd	abd	abc	abd	abc
CH04e03	5	abd	abd	abd	abc	abc	abd	abd	abd	abc	abc	abc	abd	abd	abd	abd	abc	abd	abd	abd	efg	efg
CH04g09y	5	eef	efg	efg	efg	eef	efg	efg	efg	eef	eef	eef	efg	efg	efg	efg	efg	efg	efg	eef	eef	efg

Markers	LG	Triploid seedlings from three crosses																				
		Fuji × Pink Lady									Pink Lady × Fuji										CO 2 × RO	
		FP27	FP28	FP29	FP30	FP31	FP32	FP33	FP34	FP35	**PF25**	**PF26**	**PF27**	**PF28**	**PF29**	**PF30**	**PF31**	**PF32**	**PF33**	**PF34**	CR28	CR29
CH04h02_2	5	abc	abd	abd	abd	abc	abd	abd	abd	abc	abd	abd	abc	abc	abd	abc	abc	abd	abc	abd	hhk	hhk
CH04h02_4	5	lll	lll	lll	lll	llm	lll	lll	lll	lll	nnn	nnn	nnp	nnp	nnp	nnp	nnp	nnn	nnp	nnn	hk-	hk-
CH05e06	5	abc	abd	abd	abd	abc	abd	abd	abd	abd	abc	abc	abd	abc	abd	abd	abd	abd	abd	abd	eef	efg
Hi04d02	5	abc	abc	abc	abd	abc	abc	abc	abc	abd	abd	abd	abc	abc	abc	abc	abd	abc	abd	abc	abc	abd
Hi11a03	5	abd	abd	abd	abd	abc	abd	abd	abc	abd	abc	abc	abd	abd	abd	abc	abd	abd	abd	abd	abd	abd
Hi21c08	5	hkk	hkk	hkk	hkk	hkk	hkk	hhk	hkk	hkk	hkk	hhk	hkk	hkk	hkk	hkk	hkk	hkk	hkk	hkk	efg	efg
AJ000761	6	abd	abd	abc	abd	abd	abc	abd	abd	abc	abd	abc	abc	abd	abd	abd	abc	abd	abd	abc	hhk	hkk
CH03c01	6	abd	abc	abd	abc	abd	abd	abc	abc	abd	abd	abd	abd	abc	abd	abc	abd	abc	abc	abd	eef	efg
CH03d07	6	abc	abd	abc	abd	abd	abc	abd	abc	abc	abc	abc	abc	abd	abd	abd	abc	abc	abd	abc	efg	eef
CH03d12	6	abd	abc	abd	abc	abc	abd	abd	abc	abd	abd	abd	abd	abd	abc	abc	abd	abc	abc	abd	abd	abc
Hi01d05	6	eef	efg	eef	efg	efg	eef	efg	efg	eef	efg	efg	efg	eef	efg	eef	efg	eef	eef	efg	efg	eef
CH04e05	7	nnn	nnn	nnn	nnn	nnn	nnp	nnn	nnn	nnp	lm1m2	lm1m2	lm1m2	lm1m2	lm1m2	lm1m2	lm1m2	lm1m2	lm1m2	lm1m2	llm	lll
CH05b06z_2	7	abc	abd	abd	abc	abd	abd	abc	abd	abc	abc	abd	abd	abc	abd	abc	abc	abc	abd	abd	nnn	nnn
Hi04c10x_2	7	abd	abd	abc	abd	abc	abc	abd	abd	abd	abd	abc	abd	abd	abc	abd	abc	abd	abc	abc	abc	abd
Hi05b09	7	nnp	nnn	nnp	nnp	nnp	nnp	nnp	nnp	nnn	lm1m2	lm1m2	lm1m2	lm1m2	lm1m2	lm1m2	lm1m2	lm1m2	lm1m2	lm1m2	efg	efg
CH01c06	8	abc	abd	abc	abd	abc	abc	abc	abc	abc	abc	abd	abc	abd	abd	abc	abc	abc	abc	abd	abc	abd
CH02g09	8	nnp	nnp	nnp	nnn	nnn	nnp	nnn	nnp	nnn	lm1m2	lm1m2	lm1m2	lm1m2	lm1m2	lm1m2	lm1m2	lm1m2	lm1m2	lm1m2	lll	lll
Hi04b12	8	abd	abd	abc	abd	abc	abd	abc	abc	abc	abd	abc	abd	abd	abc	abd	abc	abd	abd	abc	lm1m2	lm1m2
Hi04e05	8	nnn	nnp	nnp	nnp	nnn	nnp	nnp	nnp	nnn	lll	lll	llm	llm	lll	llm	lll	llm	llm	lll	nnp	nnp
Hi23g12	8	abc	abc	abd	abc	abd	abd	abd	abd	abc	abc	abd	abc	abc	abd	abc	abc	abc	abc	abd	nnp	nnp
CH01h02_1	9	hhk	hhk	hhk	hhk	hhk	hhk	hhk	hhk	hkk	hhk	hkk	hkk	hhk	hhk	hkk	hhk	hkk	hhk	hkk	abd	abc
CH01h02_2	9	abc	abd	abc	abc	abd	abd	abd	abd	abc	abd	abd	abd	abc	abc	abd	abc	abd	abc	abc	lm1m2	lm1m2
CH05c07	9	abc	abc	abd	abc	abd	abd	abc	abd	abc	abc	abd	abc	abc	abd	abd	abc	abd	abd	abd	eef	efg
CH05d08y_2	9	lm1m2	lm1m2	lm1m2	lm1m2	lm1m2	lm1m2	lm1m2	lm1m2	lm1m2	nnp	nnp	nnp	nnp	nnn	nnp	nnp	nnn	nnn	nnn	lm1m2	lm1m2
GD142	9	abc	abc	abc	abc	abd	abd	abc	abc	abd	abc	abd	abd	abc	abc	abd	abd	abd	abc	abd	abc	abc
Hi01d01	9	nnn	nnn	nnp	nnn	nnn	nnn	nnn	nnp	nnn	lm1m2	lm1m2	lm1m2	lm1m2	lm1m2	lm1m2	lm1m2	lm1m2	lm1m2	lm1m2	abc	abd
Hi05e07	9	abc	abc	abd	abc	abc	abc	abd	abc	abd	abd	abc	abc	abd	abd	abc	abd	abc	abc	abd	hkk	hhk
NH029a	9	abd	abd	abc	abc	abd	abd	abc	abd	abc	abc	abd	abd	abc	abc	abc	abc	abd	abc	abc	efg	efg
CH01f07a	10	abc	abc	abd	abc	abd	abc	abd	abd	abc	abc	abd	abd	abc	abd	abc	abc	abc	abc	abc	abc	abc
CH01f12	10	abd	abc	abc	abc	abd	abc	abd	abc	abc	abd	abd	abc	abc	abd	abc	abc	abd	abc	abc	abc	abc
CH02a10	10	nnp	nnn	nnn	nnn	nnp	nnp	nnn	nnn	nnn	lm1m2	lm1m2	lm1m2	lm1m2	lm1m2	lm1m2	lm1m2	lm1m2	lm1m2	lm1m2	nnn	nnn
CH02b03b	10	abc	abd	abc	abd	abc	abd	abc	abc	abd	abc	abc	abc	abd	abc	abd	abd	abd	abd	abd	abc	abc
CH02b07	10	abd	abc	abc	abc	abd	abd	abc	abc	abc	abd	abd	abc	abc	abc	abc	abc	abc	abc	abc	abc	abc

Markers	LG	Triploid seedlings from three crosses																				
		Fuji × Pink Lady									Pink Lady × Fuji										CO 2 × RO 6	
		FP27	FP28	FP29	FP30	FP31	FP32	FP33	FP34	FP35	**PF25**	**PF26**	**PF27**	**PF28**	**PF29**	**PF30**	**PF31**	**PF32**	**PF33**	**PF34**	CR28	CR29
CH02c11	10	abd	abc	abd	abc	abd	abd	abc	abd	abc	abd	abc	abd	abc	abd	abc	abc	abc	abc	abc	nnn	nnp
CH03d11	10	nnn	nnp	nnp	nnp	nnn	nnn	nnn	nnn	nnp	lm1m2	lm1m2	lm1m2	lm1m2	lm1m2	lm1m2	lm1m2	lm1m2	lm1m2	lm1m2	nnn	nnn
CH04c06y_1	10	nnn	nnp	nnn	nnn	nnp	nnn	nnp	nnn	nnn	lll	llm	lll	lll	llm	lll	lll	llm	lll	lll	llm	lll
Hi02d04	10	nnn	nnp	nnp	nnn	nnp	nnp	nnp	nnp	nnp	lm1m2	lm1m2	lm1m2	lm1m2	lm1m2	lm1m2	lm1m2	lm1m2	lm1m2	lm1m2	abd	abd
Hi04f08	10	llm	lll	lll	lll	llm	llm	lll	lll	lll	nnn	nnn	nnp	nnp	nnp	nnp	nnp	nnp	nnp	nnp	nnp	nnp
MS02a01	10	abc	abd	abd	abd	abc	abc	abd	abd	abd	abc	abc	abd	abd	abc	abd	abd	abc	abd	abd	nnn	nnn
MS06g03	10	abc	abd	abc	abd	abc	abd	abd	abc	abc	abd	abc	abc	abd	abd	abc	abd	abd	abd	abd	abc	abc
CH02d08	11	abc	abc	abd	abc	abc	abd	abc	abc	abc	abd	abd	abc	abc	abc	abd	abc	abd	abd	abd	abc	abc
CH04g07	11	abc	abd	abc	abd	abc	abd	abd	abc	abc	abd	abd	abd	abd	abd	abc	abc	abc	abd	abd	abc	abc
CH04h02_1	11	abd	abd	abc	abd	abd	abc	abd	abc	abd	abc	abd	abd	abd	abc	abc	abd	abc	abc	abc	abd	abd
CH04h02_3	11	lll	lll	llm	lll	lll	llm	lll	llm	lll	nnn	nnp	nnp	nnp	nnp	nnn	nnp	nnn	nnn	nnn	nnp	nnp
Hi06b06	11	abd	abc	abd	abc	abd	abc	abc	abd	abd	abd	abc	abc	abc	abc	abd	abc	abd	abc	abc	abd	abd
CH01b12y	12	abc	abd	abc	abd	abd	abd	abc	abc	abd	abc	abd	abc	abd	abd	abc	abc	abc	abd	abd	hk-	hk-
CH01f02	12	abc	abc	abc	abd	abc	abd	abd	abd	abd	abd	abc	abd	abc	abc	abd	abd	abd	abc	abc	efg	efg
CH01g12	12	abc	abc	abc	abd	abc	abc	abd	abd	abc	abc	abc	abd	abc	abc	abd	abc	abd	abc	abc	efg	efg
CH03h03z_2	12	lll	llm	llm	lll	llm	llm	lll	lll	llm	nnp	nnn	nnp	nnn	nnp	nnp	nnp	nnp	nnn	nnn	nnp	nnp
CH05d04	12	efg	eef	efg	eef	efg	efg	eef	eef	efg	eef	efg	eef	efg	efg	eef	eef	eef	efg	efg	abd	abd
CH05d11	12	efg	eef	eef	efg	eef	efg	efg	efg	eef	efg	eef	eef	eef	efg	efg	efg	efg	eef	efg	abd	abd
NZ28f04	12	abd	abd	abd	abc	abd	abd	abc	abc	abd	abc	abd	abc	abd	abc	abc	abc	abc	abd	abd	abc	abc
AU223486	13	llm	llm	lll	lll	lll	llm	lll	llm	lll	nnn	nnn	nnn	nnp	nnn	nnp	nnp	nnn	nnp	nnp	lll	llm
CH03a08	13	abd	abc	abd	abc	abd	abc	abc	abd	abd	abc	abc	abd	abd	abc	abd	abd	abd	abc	abd	abc	abd
CH03h03z_1	13	abd	abc	abd	abc	abd	abc	abc	abd	abc	abd	abc	abd	abd	abc	abd	abd	abd	abc	abd	abc	abd
CH05c06_1	13	lll	llm	lll	lll	llm	llm	lll	llm	lll	nnn	nnp	nnp	nnn	nnn	nnp	nnn	nnp	nnn	nnn	hk-	hk-
CH05f04	13	abc	abd	abc	abd	abc	abd	abd	abc	abd	abc	abc	abc	abc	abc	abd	abd	abd	abd	abc	hk-	hk-
CH05h05	13	abd	abd	abd	abc	abd	abc	abd	abd	abc	abd	abd	abd	abc	abd	abc	abd	abc	abc	abd	abc	abc
GD147	13	hkk	hhk	hkk	hkk	hhk	hhk	hkk	hhk	hkk	hkk	hkk	hkk	hhk	hkk	hkk	hhk	hkk	hhk	hkk	hk-	hk-
Hi03e04	13	hhk	hkk	hkk	hkk	hkk	hkk	hhk	hkk	hkk	hhk	hhk	hhk	hkk	hhk	hkk	hkk	hhk	hkk	hhk	hk-	hk-
Hi05c06_2	13	abc	abc	abd	abc	abd	abc	abc	abd	abd	abc	abc	abc	abd	abc	abd	abd	abd	abc	abd	abc	abd
Hi07b02_3	13	nnn	nnp	nnp	nnn	nnp	nnp	nnn	nnp	nnn	llm	llm	llm	lll	llm	lll	lll	llm	lll	llm	llm	lll
Hi20b03	13	abd	abc	abd	abc	abd	abd	abc	abd	abd	abd	abc	abd	abd	abc	abd	abd	abd	abc	abc	abd	abc
NH009b	13	abc	abd	abc	abd	abc	abd	abd	abc	abd	abd	abd	abc	abc	abd	abc	abd	abc	abd	abd	abd	abc
NZ03c01x_2	13	nnn	nnn	nnp	nnp	nnp	nnp	nnn	nnn	nnp	lll	lll	llm	llm	llm	lll	llm	llm	lll	llm	lll	lll

Markers	LG	Triploid seedlings from three crosses																				
		Fuji × Pink Lady									Pink Lady × Fuji										CO 2 × ROc	
		FP27	FP28	FP29	FP30	FP31	FP32	FP33	FP34	FP35	**PF25**	**PF26**	**PF27**	**PF28**	**PF29**	**PF30**	**PF31**	**PF32**	**PF33**	**PF34**	CR28	CR29
CH01g05	14	abd	abd	abc	abc	abc	abd	abc	abc	abd	abd	abc	abd	abd	abd	abc	abd	abd	abc	abd	abc	abc
CH03a02	14	abc	abc	abc	abd	abc	abc	abd	abd	abc	abd	abd	abc	abc	abc	abd	abc	abc	abc	abd	abc	abc
CH03d08	14	abd	abd	abd	abd	abd	abc	abd	abd	abc	abd	abd	abc	abc	abc	abc	abc	abc	abd	abc	abc	abc
CH05g07z_1	14	hkk	hkk	hkk	hhk	hkk	hkk	hhk	hhk	hkk	hhk	hkk	hhk	hkk	hkk	hhk	hhk	hhk	hkk	hkk	abc	abc
CH05g07z_2	14	hkk	hkk	hkk	hhk	hkk	hkk	hhk	hhk	hkk	hhk	hkk	hhk	hkk	hkk	hhk	hhk	hhk	hkk	hkk	hhk	hhk
CH02c02a_1	15	abd	abd	abc	abd	abd	abd	abd	abc	abd	abc	abc	abc	abc	abd	abc	abc	abd	abc	abc	abc	abd
CH02c09	15	nnp	nnp	nnp	nnn	nnp	nnn	nnn	nnp	nnp	lm1m2	lm1m2	lm1m2	lm1m2	lm1m2	lm1m2	lm1m2	lm1m2	lm1m2	lm1m2	lm1m2	lm1m2
CH02d11	15	abd	abc	abc	abd	abc	abd	abd	abc	abd	abc	abd	abd	abc	abd	abc	abc	abd	abc	abc	abd	abc
CH03b10	15	abc	abc	abc	abc	abd	abc	abd	abc	abc	abd	abc	abd	abd	abc	abd	abd	abc	abd	abd	abd	abc
Hi02g06	15	lm1m2	lm1m2	lm1m2	lm1m2	lm1m2	lm1m2	lm1m2	lm1m2	lm1m2	nnp	nnp	nnn	nnn	nnp	nnp	nnp	nnp	nnp	nnp	nnp	nnp
Hi04c05	15	abc	abc	abc	abd	abd	abc	abc	abd	abc	abd	abc	abd	abd	abc	abd	abd	abc	abc	abd	abd	abc
Hi06f09	15	lm1m2	lm1m2	lm1m2	lm1m2	lm1m2	lm1m2	lm1m2	lm1m2	lm1m2	nnn	nnp	nnp	nnn	nnp	nnp	nnn	nnp	nnn	nnn	abd	abc
NZ02b01	15	abd	abd	abd	abd	abc	abc	abd	abc	abd	abc	abd	abd	abc	abd	abc	abc	abd	abc	abc	lm1m2	lm1m2
CH02d10a	16	abc	abc	abd	abc	abc	abc	abd	abd	abc	abc	abc	abd	abc	abc	abd	abd	abd	abc	abc	efg	efg
CH04f10	16	lll	llm	llm	llm	lll	lll	lll	llm	llm	nnp	nnn	nnn	nnn	nnn	nnn	nnp	nnn	nnp	nnp	llm	lll
CH05a04	16	abd	abd	abc	abd	abd	abd	abc	abd	abd	abc	abc	abd	abd	abc	abd	abd	abd	abc	abd	abd	abd
CH05b06z_1	16	abd	abc	abd	abc	abc	abc	abd	abc	abc	abd	abd	abd	abc	abd	abc	abc	abc	abd	abc	efg	efg
CH05c06_2	16	abd	abd	abc	abc	abd	abd	abc	abd	abd	abc	abc	abd	abd	abc	abd	abd	abd	abd	abd	abd	abd
Hi01c11x	16	efg	efg	eef	eef	efg	efg	eef	efg	efg	eef	eef	efg	efg	efg	efg	efg	efg	efg	efg	abc	abc
Hi01d06y	16	efg	efg	eef	eef	efg	efg	eef	efg	efg	eef	eef	efg	efg	eef	efg	efg	efg	efg	efg	abc	abc
Hi04e04	16	efg	efg	eef	eef	efg	efg	eef	efg	efg	eef	eef	efg	efg	efg	efg	efg	efg	efg	efg	abc	abc
CH01h01	17	abd	abd	abc	abc	abc	abc	abd	abc	abc	abd	abd	abd	abd	abc	abc	abc	abc	abc	abc	abd	abc
CH04c06y_2	17	llm	lll	lll	llm	lll	lll	lll	lll	lll	nnp	nnp	nnp	nnp	nnp	nnn	nnp	nnn	nnn	nnp	lll	lll
CH04c06y_3	17	nnn	nnn	nnn	nnp	nnn	nnn	nnn	nnp	nnn	lll	lll	lll	lll	lll	llm	llm	lll	llm	lll	lll	lll
CH05d08y_1	17	abc	abd	abd	abd	abc	abc	abc	abd	abd	abd	abd	abd	abc	abc	abd	abd	abd	abc	abd	abc	abd
CH05g03	17	lm1m2	lm1m2	lm1m2	lm1m2	lm1m2	lm1m2	lm1m2	lm1m2	lm1m2	nnn	nnp	nnp	nnn	nnn	nnp	nnp	nnn	nnp	nnn	abd	abc
GD96	17	nnp	nnp	nnn	nnn	nnn	nnn	nnp	nnn	nnn	llm	lll	lll	lll	lll	llm	llm	llm	llm	llm	nnp	nnn
Hi03c05	17	nnn	nnn	nnn	nnp	nnp	nnn	nnn	nnp	nnn	lm1m2	lm1m2	lm1m2	lm1m2	lm1m2	lm1m2	lm1m2	lm1m2	lm1m2	lm1m2	nnn	nnn
Hi05c06_1	17	nnn	nnp	nnp	nnp	nnn	nnp	nnn	nnn	nnp	llm	llm	lll	llm	llm	lll	lll	llm	lll	llm	llm	llm
Hi07b02_1	17	nnn	nnn	nnn	nnn	nnn	nnp	nnp	nnp	nnp	lm1m2	lm1m2	lm1m2	lm1m2	lm1m2	lm1m2	lm1m2	lm1m2	lm1m2	lm1m2	abc	abc
Hi07b02_2	17	lll	llm	lll	llm	lll	llm	llm	lll	lll	nnn	nnn	nnp	nnp	nnn	nnp	nnp	nnp	nnp	nnn	nnn	nnn

Note: '-' represents a null allele, or missing data; 'p1' and 'p2' , 'm1' and 'm2'are con-dominant alleles, respectively.
