# Supplementary material for: Molecular Genetic Features of Polyploidization and Aneuploidization Reveal Unique Patterns for Genome Duplication in Diploid Malus
Source: PLoS One. 2012 Jan 10;7(1):e29449. doi: 10.1371/journal.pone.0029449 (PMC3254611; doi:10.1371/journal.pone.0029449)
Supplement: Table S11 — The distributional features of microsatellite markers in the tetraploid seedlings from six crosses. (PDF) [file pone.0029449.s012.pdf]

| Markers | LG | Tetraploid seedlings from six crosses | | | | | | | | | | | | |
|---|---|---|---|---|---|---|---|---|---|---|---|---|---|---|
| | | Gala × Fuji | | | | Fuji × Gala | | | Fuji × Pink Lady | | Pink Lady × Fuji | | M 26 × Fu | M 27 × Fu |
| | | GF56 | GF57 | GF58 | GF59 | FG54 | FG55 | FG56 | FP36 | FP37 | PF35 | PF36 | M26F28 | M27F29 |
| CH05g08 | 1 | abcd | abcd | abcd | abcd | abcd | abcd | abcd | abcd | abcd | abcd | abcd | abcd | abcd |
| Hi02b10 | 1 | nnnp | nnnp | nnnp | nnnp | lllm | lllm | lllm | lllm | lllm | nnnp | nnnp | abcd | abcd |
| Hi02c07 | 1 | lm1lm2 | lm1lm2 | lm1lm2 | lm1lm2 | np1np2 | np1np2 | np1np2 | nnnp | nnnp | lllm | lllm | hkhk | abcd |
| Hi07d08 | 1 | abcd | abcd | abcd | abcd | abcd | abcd | abcd | abcd | abcd | abcd | abcd | abcd | abcd |
| Hi12c02 | 1 | abcd | abcd | abcd | abcd | abcd | abcd | abcd | abcd | abcd | abcd | abcd | abcd | abcd |
| KA4B | 1 | hkhk | hkhk | hkhk | hkhk | hkhk | hkhk | hkhk | hkhk | hkhk | hkhk | hkhk | abcd | hkhk |
| CH02a04z | 2 | abcd | abcd | abcd | abcd | abcd | abcd | abcd | abcd | abcd | abcd | abcd | abcd | abcd |
| CH02c02a_2 | 2 | lm1lm2 | lm1lm2 | lm1lm2 | lm1lm2 | np1np2 | np1np2 | np1np2 | nnnp | nnnp | lllm | lllm | lllm | lllm |
| CH02c02a_3 | 2 | abcd | abcd | abcd | abcd | abcd | abcd | abcd | abcd | abcd | abcd | abcd | np1np2 | abcd |
| CH02c06 | 2 | abcd | abcd | abcd | abcd | abcd | abcd | abcd | abcd | abcd | abcd | abcd | abcd | abcd |
| CH03d01 | 2 | abcd | abcd | abcd | abcd | abcd | abcd | abcd | abcd | abcd | abcd | abcd | abcd | abcd |
| CH03d10 | 2 | lm1lm2 | lm1lm2 | lm1lm2 | lm1lm2 | np1np2 | np1np2 | np1np2 | nnnp | nnnp | lllm | lllm | hkhk | abcd |
| CH05e03 | 2 | abcd | abcd | abcd | abcd | abcd | abcd | abcd | abcd | abcd | abcd | abcd | abcd | abcd |
| CN493139 | 2 | abcd | abcd | abcd | abcd | abcd | abcd | abcd | abcd | abcd | abcd | abcd | abcd | abcd |
| Hi02a07 | 2 | abcd | abcd | abcd | abcd | abcd | abcd | abcd | hkhk | hkhk | hkhk | hkhk | abcd | abcd |
| Hi05c06_3 | 2 | nnnp | nnnp | nnnp | nnnp | lllm | lllm | lllm | lllm | lllm | nnnp | nnnp | nnnp | nnnp |
| AU223657 | 3 | lm1lm2 | lm1lm2 | lm1lm2 | lm1lm2 | np1np2 | np1np2 | np1np2 | np1np2 | np1np2 | llm1m2 | llm1m2 | abcd | abcd |
| CH03e03 | 3 | abcd | abcd | abcd | abcd | abcd | abcd | abcd | abcd | abcd | abcd | abcd | abcd | abcd |
| CH03g07 | 3 | abcd | abcd | abcd | abcd | abcd | abcd | abcd | abcd | abcd | abcd | abcd | efeg | efeg |
| HGA8bY | 3 | abcd | abcd | abcd | abcd | abcd | abcd | abcd | abcd | abcd | abcd | abcd | np1np2 | np1np2 |
| Hi04c10x_1 | 3 | abcd | abcd | abcd | abcd | abcd | abcd | abcd | abcd | abcd | abcd | abcd | np1np2 | np1np2 |
| Hi07e08x | 3 | abcd | abcd | abcd | abcd | abcd | abcd | abcd | abcd | abcd | abcd | abcd | abcd | abcd |
| CH02c02b | 4 | abcd | abcd | abcd | abcd | abcd | abcd | abcd | abcd | abcd | abcd | abcd | abcd | abcd |
| CH04e02 | 4 | efeg | efeg | efeg | efeg | efeg | efeg | efeg | efeg | efeg | efeg | efeg | abcd | efeg |
| CH05d02 | 4 | abcd | abcd | abcd | abcd | abcd | abcd | abcd | abcd | abcd | abcd | abcd | efeg | abcd |
| GD162 | 4 | abcd | abcd | abcd | abcd | abcd | abcd | abcd | abcd | abcd | abcd | abcd | abcd | abcd |
| Hi04c10x_3 | 4 | abcd | abcd | abcd | abcd | abcd | abcd | abcd | abcd | abcd | abcd | abcd | abcd | abcd |
| Hi07b02_4 | 4 | efeg | efeg | efeg | efeg | efeg | efeg | efeg | abcd | abcd | abcd | abcd | efeg | abcd |
| CH02a08z | 5 | np1np2 | np1np2 | np1np2 | np1np2 | lm1lm2 | lm1lm2 | lm1lm2 | llm1m2 | llm1m2 | np1np2 | np1np2 | np1np2 | abcd |
| CH03a04 | 5 | abcd | abcd | abcd | abcd | abcd | abcd | abcd | abcd | abcd | abcd | abcd | abcd | efeg |
| CH03a09 | 5 | efeg | efeg | efeg | efeg | efeg | efeg | efeg | abcd | abcd | abcd | abcd | abcd | efeg |
| CH04e03 | 5 | efeg | efeg | efeg | efeg | efeg | efeg | efeg | abcd | abcd | abcd | abcd | efeg | efeg |
| CH04g09y | 5 | abcd | abcd | abcd | abcd | abcd | abcd | abcd | efeg | efeg | efeg | efeg | abcd | abcd |

| Markers | LG | Tetraploid seedlings from six crosses | | | | | | | | | | | | |
|---|---|---|---|---|---|---|---|---|---|---|---|---|---|---|
| | | Gala × Fuji | | | | Fuji × Gala | | | Fuji × Pink Lady | | Pink Lady × Fuji | | M 26 × Fu | M 27 × Fu |
| | | GF56 | GF57 | GF58 | GF59 | FG54 | FG55 | FG56 | FP36 | FP37 | PF35 | PF36 | M26F28 | M27F29 |
| CH04h02_2 | 5 | hkhk | hkhk | hkhk | hkhk | hkhk | hkhk | hkhk | abcd | abcd | abcd | abcd | np1np2 | hkhk |
| CH04h02_4 | 5 | hkhk | hkhk | hkhk | hkhk | hkhk | hkhk | hkhk | lllm | lllm | nnnp | nnnp | hkhk | np1np2 |
| CH05e06 | 5 | abcd | abcd | abcd | abcd | abcd | abcd | abcd | abcd | abcd | abcd | abcd | abcd | hkhk |
| Hi04d02 | 5 | hkhk | hkhk | hkhk | hkhk | hkhk | hkhk | hkhk | abcd | abcd | abcd | abcd | abcd | hkhk |
| Hi11a03 | 5 | hkhk | hkhk | hkhk | hkhk | hkhk | hkhk | hkhk | abcd | abcd | abcd | abcd | abcd | abcd |
| Hi21c08 | 5 | hkhk | hkhk | hkhk | hkhk | hkhk | hkhk | hkhk | hkhk | hkhk | hkhk | hkhk | hkhk | hkhk |
| AJ000761 | 6 | hkhk | hkhk | hkhk | hkhk | hkhk | hkhk | hkhk | abcd | abcd | abcd | abcd | abcd | abcd |
| CH03c01 | 6 | hkhk | hkhk | hkhk | hkhk | hkhk | hkhk | hkhk | abcd | abcd | abcd | abcd | efeg | abcd |
| CH03d07 | 6 | abcd | abcd | abcd | abcd | abcd | abcd | abcd | abcd | abcd | abcd | abcd | abcd | abcd |
| CH03d12 | 6 | abcd | abcd | abcd | abcd | abcd | abcd | abcd | abcd | abcd | abcd | abcd | abcd | efeg |
| Hi01d05 | 6 | efeg | efeg | efeg | efeg | efeg | efeg | efeg | efeg | efeg | efeg | efeg | efeg | efeg |
| CH04e05 | 7 | lm1lm2 | lm1lm2 | lm1lm2 | lm1lm2 | np1np2 | np1np2 | np1np2 | np1np2 | np1np2 | llm1m2 | llm1m2 | lllm | lllm |
| CH05b06z_2 | 7 | efeg | efeg | efeg | efeg | efeg | efeg | efeg | abcd | abcd | abcd | abcd | np1np2 | np1np2 |
| Hi04c10x_2 | 7 | abcd | abcd | abcd | abcd | abcd | abcd | abcd | abcd | abcd | abcd | abcd | abcd | abcd |
| Hi05b09 | 7 | lllm | lllm | lllm | lllm | nnnp | nnnp | nnnp | np1np2 | np1np2 | llm1m2 | llm1m2 | abcd | abcd |
| CH01c06 | 8 | abcd | abcd | abcd | abcd | abcd | abcd | abcd | abcd | abcd | abcd | abcd | abcd | abcd |
| CH02g09 | 8 | lm1lm2 | lm1lm2 | lm1lm2 | lm1lm2 | np1np2 | np1np2 | np1np2 | np1np2 | np1np2 | llm1m2 | llm1m2 | abcd | abcd |
| Hi04b12 | 8 | abcd | abcd | abcd | abcd | abcd | abcd | abcd | abcd | abcd | abcd | abcd | abcd | abcd |
| Hi04e05 | 8 | lllm | lllm | lllm | lllm | nnnp | nnnp | nnnp | nnnp | nnnp | lllm | lllm | lllm | lllm |
| Hi23g12 | 8 | nnnp | nnnp | nnnp | nnnp | lllm | lllm | lllm | abcd | abcd | abcd | abcd | abcd | abcd |
| CH01h02_1 | 9 | hkhk | hkhk | hkhk | hkhk | hkhk | hkhk | hkhk | hkhk | hkhk | hkhk | hkhk | abcd | efeg |
| CH01h02_2 | 9 | abcd | abcd | abcd | abcd | abcd | abcd | abcd | abcd | abcd | abcd | abcd | abcd | abcd |
| CH05c07 | 9 | abcd | abcd | abcd | abcd | abcd | abcd | abcd | abcd | abcd | abcd | abcd | abcd | efeg |
| CH05d08y_2 | 9 | np1np2 | np1np2 | np1np2 | np1np2 | lm1lm2 | lm1lm2 | lm1lm2 | llm1m2 | llm1m2 | np1np2 | np1np2 | efeg | abcd |
| GD142 | 9 | abcd | abcd | abcd | abcd | abcd | abcd | abcd | abcd | abcd | abcd | abcd | abcd | efeg |
| Hi01d01 | 9 | lm1lm2 | lm1lm2 | lm1lm2 | lm1lm2 | np1np2 | np1np2 | np1np2 | np1np2 | np1np2 | llm1m2 | llm1m2 | efeg | np1np2 |
| Hi05e07 | 9 | abcd | abcd | abcd | abcd | abcd | abcd | abcd | abcd | abcd | abcd | abcd | abcd | abcd |
| NH029a | 9 | abcd | abcd | abcd | abcd | abcd | abcd | abcd | abcd | abcd | abcd | abcd | abcd | abcd |
| CH01f07a | 10 | abcd | abcd | abcd | abcd | abcd | abcd | abcd | abcd | abcd | abcd | abcd | abcd | abcd |
| CH01f12 | 10 | abcd | abcd | abcd | abcd | abcd | abcd | abcd | abcd | abcd | abcd | abcd | abcd | abcd |
| CH02a10 | 10 | lm1lm2 | lm1lm2 | lm1lm2 | lm1lm2 | np1np2 | np1np2 | np1np2 | np1np2 | np1np2 | llm1m2 | llm1m2 | abcd | abcd |
| CH02b03b | 10 | abcd | abcd | abcd | abcd | abcd | abcd | abcd | abcd | abcd | abcd | abcd | abcd | abcd |
| CH02b07 | 10 | abcd | abcd | abcd | abcd | abcd | abcd | abcd | abcd | abcd | abcd | abcd | abcd | abcd |

| Markers | LG | Tetraploid seedlings from six crosses | | | | | | | | | | | | |
|---|---|---|---|---|---|---|---|---|---|---|---|---|---|---|
| | | Gala × Fuji | | | | Fuji × Gala | | | Fuji × Pink Lady | | Pink Lady × Fuji | | M 26 × Fu | M 27 × Fu |
| | | GF56 | GF57 | GF58 | GF59 | FG54 | FG55 | FG56 | FP36 | FP37 | PF35 | PF36 | M26F28 | M27F29 |
| CH02c11 | 10 | abcd | abcd | abcd | abcd | abcd | abcd | abcd | abcd | abcd | abcd | abcd | np1np2 | abcd |
| CH03d11 | 10 | lm1lm2 | lm1lm2 | lm1lm2 | lm1lm2 | np1np2 | np1np2 | np1np2 | np1np2 | np1np2 | llm1m2 | llm1m2 | abcd | nnnp |
| CH04c06y_1 | 10 | lm1lm2 | lm1lm2 | lm1lm2 | lm1lm2 | np1np2 | np1np2 | np1np2 | nnnp | nnnp | lllm | lllm | lllm | lllm |
| Hi02d04 | 10 | lllm | lllm | lllm | lllm | nnnp | nnnp | nnnp | np1np2 | np1np2 | llm1m2 | llm1m2 | abcd | abcd |
| Hi04f08 | 10 | nnnp | nnnp | nnnp | nnnp | lllm | lllm | lllm | lllm | lllm | nnnp | nnnp | abcd | abcd |
| MS02a01 | 10 | nnnp | nnnp | nnnp | nnnp | lllm | lllm | lllm | abcd | abcd | abcd | abcd | np1np2 | np1np2 |
| MS06g03 | 10 | abcd | abcd | abcd | abcd | abcd | abcd | abcd | abcd | abcd | abcd | abcd | abcd | abcd |
| CH02d08 | 11 | abcd | abcd | abcd | abcd | abcd | abcd | abcd | abcd | abcd | abcd | abcd | abcd | abcd |
| CH04g07 | 11 | abcd | abcd | abcd | abcd | abcd | abcd | abcd | abcd | abcd | abcd | abcd | abcd | abcd |
| CH04h02_1 | 11 | abcd | abcd | abcd | abcd | abcd | abcd | abcd | abcd | abcd | abcd | abcd | hkhk | nnnp |
| CH04h02_3 | 11 | nnnp | nnnp | nnnp | nnnp | lllm | lllm | lllm | lllm | lllm | nnnp | nnnp | nnnp | hkhk |
| Hi06b06 | 11 | abcd | abcd | abcd | abcd | abcd | abcd | abcd | abcd | abcd | abcd | abcd | abcd | abcd |
| CH01b12y | 12 | abcd | abcd | abcd | abcd | abcd | abcd | abcd | abcd | abcd | abcd | abcd | abcd | abcd |
| CH01f02 | 12 | efeg | efeg | efeg | efeg | efeg | efeg | efeg | abcd | abcd | abcd | abcd | hkhk | efeg |
| CH01g12 | 12 | abcd | abcd | abcd | abcd | abcd | abcd | abcd | abcd | abcd | abcd | abcd | abcd | abcd |
| CH03h03z_2 | 12 | nnnp | nnnp | nnnp | nnnp | lllm | lllm | lllm | lllm | lllm | nnnp | nnnp | np1np2 | np1np2 |
| CH05d04 | 12 | efeg | efeg | efeg | efeg | efeg | efeg | efeg | efeg | efeg | efeg | efeg | abcd | np1np2 |
| CH05d11 | 12 | efeg | efeg | efeg | efeg | efeg | efeg | efeg | efeg | efeg | efeg | efeg | abcd | abcd |
| NZ28f04 | 12 | abcd | abcd | abcd | abcd | abcd | abcd | abcd | abcd | abcd | abcd | abcd | abcd | abcd |
| AU223486 | 13 | hkhk | hkhk | hkhk | hkhk | hkhk | hkhk | hkhk | lllm | lllm | nnnp | nnnp | hkhk | hkhk |
| CH03a08 | 13 | abcd | abcd | abcd | abcd | abcd | abcd | abcd | abcd | abcd | abcd | abcd | abcd | abcd |
| CH03h03z_1 | 13 | abcd | abcd | abcd | abcd | abcd | abcd | abcd | abcd | abcd | abcd | abcd | hkhk | abcd |
| CH05c06_1 | 13 | abcd | abcd | abcd | abcd | abcd | abcd | abcd | lllm | lllm | nnnp | nnnp | abcd | nnnp |
| CH05f04 | 13 | abcd | abcd | abcd | abcd | abcd | abcd | abcd | abcd | abcd | abcd | abcd | abcd | abcd |
| CH05h05 | 13 | abcd | abcd | abcd | abcd | abcd | abcd | abcd | abcd | abcd | abcd | abcd | abcd | abcd |
| GD147 | 13 | hkhk | hkhk | hkhk | hkhk | hkhk | hkhk | hkhk | hkhk | hkhk | hkhk | hkhk | abcd | abcd |
| Hi03e04 | 13 | abcd | abcd | abcd | abcd | abcd | abcd | abcd | hkhk | hkhk | hkhk | hkhk | abcd | abcd |
| Hi05c06_2 | 13 | hkhk | hkhk | hkhk | hkhk | hkhk | hkhk | hkhk | abcd | abcd | abcd | abcd | hkhk | hkhk |
| Hi07b02_3 | 13 | lllm | lllm | lllm | lllm | nnnp | nnnp | nnnp | nnnp | nnnp | lllm | lllm | lllm | lllm |
| Hi20b03 | 13 | abcd | abcd | abcd | abcd | abcd | abcd | abcd | abcd | abcd | abcd | abcd | abcd | abcd |
| NH009b | 13 | abcd | abcd | abcd | abcd | abcd | abcd | abcd | abcd | abcd | abcd | abcd | abcd | abcd |
| NZ03c01x_2 | 13 | lllm | lllm | lllm | lllm | nnnp | nnnp | nnnp | nnnp | nnnp | lllm | lllm | np1np2 | np1np2 |

| Markers | LG | Tetraploid seedlings from six crosses | | | | | | | | | | | | |
|---|---|---|---|---|---|---|---|---|---|---|---|---|---|---|
| | | Gala × Fuji | | | | Fuji × Gala | | | Fuji × Pink Lady | | Pink Lady × Fuji | | M 26 × Fu | M 27 × Fu |
| | | GF56 | GF57 | GF58 | GF59 | FG54 | FG55 | FG56 | FP36 | FP37 | PF35 | PF36 | M26F28 | M27F29 |
| CH01g05 | 14 | abcd | abcd | abcd | abcd | abcd | abcd | abcd | abcd | abcd | abcd | abcd | hkhk | abcd |
| CH03a02 | 14 | abcd | abcd | abcd | abcd | abcd | abcd | abcd | abcd | abcd | abcd | abcd | abcd | abcd |
| CH03d08 | 14 | abcd | abcd | abcd | abcd | abcd | abcd | abcd | abcd | abcd | abcd | abcd | abcd | abcd |
| CH05g07z_1 | 14 | hkhk | hkhk | hkhk | hkhk | hkhk | hkhk | hkhk | hkhk | hkhk | hkhk | hkhk | hkhk | abcd |
| CH05g07z_2 | 14 | hkhk | hkhk | hkhk | hkhk | hkhk | hkhk | hkhk | hkhk | hkhk | hkhk | hkhk | hkhk | hkhk |
| CH02c02a_1 | 15 | abcd | abcd | abcd | abcd | abcd | abcd | abcd | abcd | abcd | abcd | abcd | abcd | abcd |
| CH02c09 | 15 | lm1lm2 | lm1lm2 | lm1lm2 | lm1lm2 | np1np2 | np1np2 | np1np2 | np1np2 | np1np2 | llm1m2 | llm1m2 | abcd | abcd |
| CH02d11 | 15 | abcd | abcd | abcd | abcd | abcd | abcd | abcd | abcd | abcd | abcd | abcd | abcd | abcd |
| CH03b10 | 15 | abcd | abcd | abcd | abcd | abcd | abcd | abcd | abcd | abcd | abcd | abcd | abcd | abcd |
| Hi02g06 | 15 | np1np2 | np1np2 | np1np2 | np1np2 | lm1lm2 | lm1lm2 | lm1lm2 | llm1m2 | llm1m2 | np1np2 | np1np2 | abcd | nnnp |
| Hi04c05 | 15 | abcd | abcd | abcd | abcd | abcd | abcd | abcd | abcd | abcd | abcd | abcd | abcd | abcd |
| Hi06f09 | 15 | abcd | abcd | abcd | abcd | abcd | abcd | abcd | llm1m2 | llm1m2 | np1np2 | np1np2 | abcd | abcd |
| NZ02b01 | 15 | abcd | abcd | abcd | abcd | abcd | abcd | abcd | abcd | abcd | abcd | abcd | abcd | abcd |
| CH02d10a | 16 | abcd | abcd | abcd | abcd | abcd | abcd | abcd | abcd | abcd | abcd | abcd | abcd | efeg |
| CH04f10 | 16 | abcd | abcd | abcd | abcd | abcd | abcd | abcd | lllm | lllm | nnnp | nnnp | lllm | lllm |
| CH05a04 | 16 | abcd | abcd | abcd | abcd | abcd | abcd | abcd | abcd | abcd | abcd | abcd | efeg | efeg |
| CH05b06z_1 | 16 | efeg | efeg | efeg | efeg | efeg | efeg | efeg | abcd | abcd | abcd | abcd | abcd | efeg |
| CH05c06_2 | 16 | abcd | abcd | abcd | abcd | abcd | abcd | abcd | abcd | abcd | abcd | abcd | abcd | abcd |
| Hi01c11x | 16 | efeg | efeg | efeg | efeg | efeg | efeg | efeg | efeg | efeg | efeg | efeg | abcd | abcd |
| Hi01d06y | 16 | abcd | abcd | abcd | abcd | abcd | abcd | abcd | efeg | efeg | efeg | efeg | abcd | abcd |
| Hi04e04 | 16 | abcd | abcd | abcd | abcd | abcd | abcd | abcd | efeg | efeg | efeg | efeg | efeg | abcd |
| CH01h01 | 17 | abcd | abcd | abcd | abcd | abcd | abcd | abcd | abcd | abcd | abcd | abcd | abcd | abcd |
| CH04c06y_2 | 17 | abcd | abcd | abcd | abcd | abcd | abcd | abcd | lllm | lllm | nnnp | nnnp | abcd | abcd |
| CH04c06y_3 | 17 | lm1lm2 | lm1lm2 | lm1lm2 | lm1lm2 | np1np2 | np1np2 | np1np2 | nnnp | nnnp | lllm | lllm | llm1m2 | llm1m2 |
| CH05d08y_1 | 17 | abcd | abcd | abcd | abcd | abcd | abcd | abcd | abcd | abcd | abcd | abcd | abcd | abcd |
| CH05g03 | 17 | abcd | abcd | abcd | abcd | abcd | abcd | abcd | llm1m2 | llm1m2 | np1np2 | np1np2 | abcd | abcd |
| GD96 | 17 | lm1lm2 | lm1lm2 | lm1lm2 | lm1lm2 | np1np2 | np1np2 | np1np2 | nnnp | nnnp | lllm | lllm | lllm | llm1m2 |
| Hi03c05 | 17 | lllm | lllm | lllm | lllm | nnnp | nnnp | nnnp | np1np2 | np1np2 | llm1m2 | llm1m2 | abcd | abcd |
| Hi05c06_1 | 17 | lllm | lllm | lllm | lllm | nnnp | nnnp | nnnp | nnnp | nnnp | lllm | lllm | hkhk | np1np2 |
| Hi07b02_1 | 17 | lllm | lllm | lllm | lllm | nnnp | nnnp | nnnp | np1np2 | np1np2 | llm1m2 | llm1m2 | abcd | abcd |
| Hi07b02_2 | 17 | nnnp | nnnp | nnnp | nnnp | lllm | lllm | lllm | lllm | lllm | nnnp | nnnp | abcd | abcd |
