# Supplementary material for: Molecular Genetic Features of Polyploidization and Aneuploidization Reveal Unique Patterns for Genome Duplication in Diploid Malus
Source: PLoS One. 2012 Jan 10;7(1):e29449. doi: 10.1371/journal.pone.0029449 (PMC3254611; doi:10.1371/journal.pone.0029449)
Supplement: Table S12 — Aneuploid seedlings and their cytotypes in the seven apple diploid crosses. (PDF) [file pone.0029449.s013.pdf]

Crosses	2*n*-1	2*n*+1	2*n*+3	2*n*+6	2*n*+7	2*n*+8	2*n*+9	2*n*+10	2*n*+11	Total
Gala × Fuji	2	5	3	5	7	7	5	4	2	40
Fuji × Gala	1	4	3	5	8	7	5	2	2	37
Fuji × Pink Lady	1	3	1	4	5	6	3	2	1	26
Pink Lady × Fuji	1	2	0	4	6	5	3	3	0	24
M 26 × Fu 2	0	1	1	5	6	4	3	2	1	23
M 27 × Fu 2	0	2	2	4	7	5	3	2	0	25
CO 2 × RO 6	1	2	1	5	7	7	2	1	1	27
Total	6	19	11	32	46	41	24	16	7	202
