# Supplementary material for: Molecular Genetic Features of Polyploidization and Aneuploidization Reveal Unique Patterns for Genome Duplication in Diploid Malus
Source: PLoS One. 2012 Jan 10;7(1):e29449. doi: 10.1371/journal.pone.0029449 (PMC3254611; doi:10.1371/journal.pone.0029449)
Supplement: Table S13 — ‘ 2n−1 ’ aneuploid seedlings and their affected chromosome. ‘LG02’ represents a chromosome; ‘GF01’ is a seedling from a cross of ‘Gala×Fuji’. ‘GF’, ‘FG’, ‘FP’, ‘PF’, ‘M26F’, ‘M27F’, and ‘CR’ represent a cross of ‘Gala×Fuji’, ‘Fuji×Gala’, ‘Fuji×Pink Lady’, ‘Pink Lady×Fuji’, ‘M 26×Fu 2’, ‘M 27×Fu 2’, and ‘CO 2×RO 6’, respectively. 1 = the affected Linkage Group (chromosome) from the spermatozoa that fertilized “normal” ova in the respective individual progeny. (PDF) [file pone.0029449.s014.pdf]

Progenies	The affected chromosome														
	LG02	LG03	LG04	LG05	LG06	LG07	LG09	LG10	LG11	LG12	LG13	LG14	LG15	LG16	LG17
GF01	1														
GF02															1
FG01								1							
FP01	1														
PF01															1
CR01								1							
