# Supplementary material for: Molecular Genetic Features of Polyploidization and Aneuploidization Reveal Unique Patterns for Genome Duplication in Diploid Malus
Source: PLoS One. 2012 Jan 10;7(1):e29449. doi: 10.1371/journal.pone.0029449 (PMC3254611; doi:10.1371/journal.pone.0029449)
Supplement: Table S15 — ‘ 2n+3 ’ aneuploid seedlings and their extra chromosomes. (PDF) [file pone.0029449.s016.pdf]

Progenies	The affected chromosomes														
	LG02	LG03	LG04	LG05	LG06	LG07	LG09	LG10	LG11	LG12	LG13	LG14	LG15	LG16	LG17
GF08		1	1	1											
GF09	1	1	1												
GF10							1	1		1					
FG06													1	1	1
FG07			1	1	1										
FG08								1			1			1	
FP05	1							1		1					
M26F02		1					1						1		
M27F03				1				1				1			
M27F04							1					1	1		
CR04										1				1	1
