# Supplementary material for: Molecular Genetic Features of Polyploidization and Aneuploidization Reveal Unique Patterns for Genome Duplication in Diploid Malus
Source: PLoS One. 2012 Jan 10;7(1):e29449. doi: 10.1371/journal.pone.0029449 (PMC3254611; doi:10.1371/journal.pone.0029449)
Supplement: Table S16 — ‘ 2n+6 ’ aneuploid seedlings and their extra chromosomes. (PDF) [file pone.0029449.s017.pdf]

Progenies	The affected chromosomes														
	LG02	LG03	LG04	LG05	LG06	LG07	LG09	LG10	LG11	LG12	LG13	LG14	LG15	LG16	LG17
GF11	1	1		1	1								1		1
GF12	1	1	1		1				1					1	
GF13	1			1	1	1					1			1	
GF14		1			1		1	1						1	1
GF15				1	1		1			1	1	1			
FG09			1				1	1		1		1			1
FG10	1						1			1			1	1	1
FG11				1	1		1			1	1		1		
FG12	1		1				1	1		1	1				
FG13	1	1	1					1				1			1
FP06		1	1	1			1	1						1	
FP07			1				1	1					1	1	1
FP08	1		1	1	1								1		1
FP09		1	1		1				1			1			1
PF04							1	1			1	1		1	1
PF05							1	1	1	1			1		1
PF06	1		1	1			1				1	1			
PF07			1				1	1		1	1		1		
M26F03	1						1	1	1			1			1
M26F04		1	1	1	1							1	1		
M26F05		1	1		1		1	1						1	
M26F06	1							1		1			1	1	1
M26F07	1	1		1	1					1			1		
M27F05			1	1	1		1	1						1	
M27F06				1			1	1		1				1	1
M27F07		1	1	1						1				1	1
M27F08		1	1	1					1		1	1			
CR05	1					1	1	1				1		1	
CR06							1	1			1		1	1	1
CR07				1	1				1	1	1	1			
CR08			1					1		1	1		1	1	
CR09			1					1	1		1			1	1
