# Supplementary material for: Molecular Genetic Features of Polyploidization and Aneuploidization Reveal Unique Patterns for Genome Duplication in Diploid Malus
Source: PLoS One. 2012 Jan 10;7(1):e29449. doi: 10.1371/journal.pone.0029449 (PMC3254611; doi:10.1371/journal.pone.0029449)
Supplement: Table S17 — ‘ 2n+7 ’ aneuploid seedlings and their extra chromosomes. (PDF) [file pone.0029449.s018.pdf]

Progenies	The affected chromosomes														
	LG02	LG03	LG04	LG05	LG06	LG07	LG09	LG10	LG11	LG12	LG13	LG14	LG15	LG16	LG17
GF16			1	1				1		1			1	1	1
GF17				1	1		1	1	1	1	1				
GF18	1		1				1	1		1	1				1
GF19	1	1	1					1		1		1			1
GF20		1	1		1		1	1		1	1				
GF21	1					1	1	1		1				1	1
GF22		1		1	1			1		1		1			1
FG14	1		1		1		1		1				1	1	
FG15	1			1	1		1	1			1			1	
FG16		1					1	1		1			1	1	1
FG17			1	1	1		1			1	1	1			
FG18	1	1		1	1						1		1	1	
FG19		1	1				1	1		1		1			1
FG20				1			1	1		1		1	1		1
FG21				1	1			1		1	1		1		1
FP10	1		1				1	1	1	1	1				
FP11	1		1				1	1		1		1			1
FP12	1		1	1			1				1	1		1	
FP13			1				1	1			1		1	1	1
FP14	1		1				1	1	1			1			1
PF08		1	1	1	1							1	1		1
PF09		1	1		1		1	1		1	1				
PF10	1			1			1	1		1				1	1
PF11		1		1	1					1			1	1	1
PF12	1	1	1		1				1				1	1	
PF13	1			1	1		1				1			1	1
M26F08		1					1	1		1			1	1	1
M26F09			1	1	1		1			1	1	1			
M26F10	1	1		1			1				1		1	1	
M26F11			1				1	1	1	1		1			1
M26F12	1	1	1	1	1					1			1		
M26F13			1	1	1		1	1	1	1					
M27F09				1			1	1		1			1	1	1
M27F10		1	1	1						1	1			1	1
M27F11		1	1	1					1		1	1		1	
M27F12	1					1	1	1				1	1	1	
M27F13	1				1		1	1					1	1	1
M27F14	1		1	1	1		1				1	1			
M27F15	1		1				1	1		1	1		1		
CR10		1	1				1	1				1		1	1
CR11		1	1	1	1	1							1	1	
CR12		1	1	1			1	1			1			1	
CR13				1			1	1		1			1	1	1
CR14	1		1	1	1								1	1	1
CR15		1		1	1				1				1	1	1
CR16	1						1	1			1	1		1	1
