# Supplementary material for: Molecular Genetic Features of Polyploidization and Aneuploidization Reveal Unique Patterns for Genome Duplication in Diploid Malus
Source: PLoS One. 2012 Jan 10;7(1):e29449. doi: 10.1371/journal.pone.0029449 (PMC3254611; doi:10.1371/journal.pone.0029449)
Supplement: Table S18 — ‘ 2n+8 ’ aneuploid seedlings and their extra chromosomes. (PDF) [file pone.0029449.s019.pdf]

Progenies	The affected chromosomes														
	LG02	LG03	LG04	LG05	LG06	LG07	LG09	LG10	LG11	LG12	LG13	LG14	LG15	LG16	LG17
GF23		1	1	1	1							1	1	1	1
GF24		1	1	1	1		1	1		1	1				
GF25	1			1				1	1	1			1	1	1
GF26	1		1	1	1			1					1	1	1
GF27	1	1	1		1			1	1					1	1
GF28	1		1	1	1		1				1			1	1
GF29			1				1	1		1	1		1	1	1
FG22			1	1	1		1			1	1	1	1		
FG23	1	1		1			1	1			1	1		1	
FG24			1	1			1	1	1	1				1	1
FG25	1	1	1	1	1					1			1	1	
FG26		1	1	1	1		1	1	1	1					
FG27				1			1	1	1	1			1	1	1
FG28		1	1	1			1			1	1			1	1
FP15		1	1	1					1	1	1			1	1
FP16	1	1				1	1	1					1	1	1
FP17					1	1	1	1		1			1	1	1
FP18			1	1			1	1		1	1	1	1		
FP19	1		1				1	1		1	1		1	1	
FP20	1	1	1				1	1				1		1	1
PF14	1	1	1	1	1	1	1							1	
PF15			1	1	1		1	1	1	1	1				
PF16	1		1				1	1		1	1		1		1
PF17	1	1		1	1			1		1				1	1
PF18	1	1	1		1		1	1		1	1				
M26F14	1				1	1	1	1		1				1	1
M26F15		1		1	1		1			1	1			1	1
M26F16	1			1	1		1		1			1	1	1	
M26F17	1		1	1	1		1	1		1				1	
M27F16			1				1	1	1	1			1	1	1
M27F17			1	1	1			1		1	1	1	1		
M27F18	1	1		1			1				1	1	1	1	
M27F19		1	1				1	1		1		1	1		1
M27F20				1	1		1	1		1		1	1		1
CR17				1	1			1		1	1	1	1		1
CR18	1	1	1				1	1	1	1	1				
CR19	1			1			1	1		1	1	1			1
CR20	1		1	1			1				1	1		1	1
CR21		1	1				1	1		1	1		1	1	
CR22	1	1			1		1	1	1			1			1
CR23			1	1	1		1	1				1		1	1
