# Supplementary material for: Molecular Genetic Features of Polyploidization and Aneuploidization Reveal Unique Patterns for Genome Duplication in Diploid Malus
Source: PLoS One. 2012 Jan 10;7(1):e29449. doi: 10.1371/journal.pone.0029449 (PMC3254611; doi:10.1371/journal.pone.0029449)
Supplement: Table S19 — ‘ 2n+9 ’ aneuploid seedlings and their extra chromosomes. (PDF) [file pone.0029449.s020.pdf]

Progenies	The affected chromosomes														
	LG02	LG03	LG04	LG05	LG06	LG07	LG09	LG10	LG11	LG12	LG13	LG14	LG15	LG16	LG17
GF30				1	1		1	1	1	1			1	1	1
GF31		1	1	1			1			1	1		1	1	1
GF32	1	1	1	1					1	1	1	1		1	
GF33	1					1	1	1			1	1	1	1	1
GF34	1					1	1	1			1	1	1	1	1
FG29	1		1	1			1	1		1	1	1	1		
FG30	1		1		1		1	1		1	1		1	1	
FG31	1	1	1		1		1	1				1		1	1
FG32	1	1	1	1	1	1	1	1						1	
FG33	1		1	1	1		1	1	1	1	1				
FP21	1		1				1	1		1	1		1	1	1
FP22	1	1	1	1	1			1		1		1			1
FP23		1	1	1	1		1					1	1	1	1
PF19		1	1	1	1		1	1		1	1				1
PF20	1			1			1	1	1	1			1	1	1
PF21		1	1	1	1			1		1			1	1	1
M26F18	1	1	1		1			1	1				1	1	1
M26F19	1		1	1	1		1	1			1			1	1
M26F20		1		1			1	1		1	1		1	1	1
M27F21			1	1	1		1			1	1	1	1		1
M27F22	1	1		1			1	1			1	1		1	1
M27F23			1	1			1	1	1	1		1		1	1
CR24	1	1	1	1	1					1			1	1	1
CR25	1	1	1	1	1		1	1	1	1					
