# Supplementary material for: Molecular Genetic Features of Polyploidization and Aneuploidization Reveal Unique Patterns for Genome Duplication in Diploid Malus
Source: PLoS One. 2012 Jan 10;7(1):e29449. doi: 10.1371/journal.pone.0029449 (PMC3254611; doi:10.1371/journal.pone.0029449)
Supplement: Table S20 — ‘ 2n + 10 ’ aneuploid seedlings and their extra chromosomes. (PDF) [file pone.0029449.s021.pdf]

Progenies	The affected chromosomes														
	LG02	LG03	LG04	LG05	LG06	LG07	LG09	LG10	LG11	LG12	LG13	LG14	LG15	LG16	LG17
GF35	1	1	1	1	1	1	1	1						1	1
GF36	1	1	1	1	1		1	1	1	1	1				
GF37	1		1				1	1	1	1	1		1	1	1
GF38	1	1	1	1	1			1		1		1		1	1
FG34		1	1	1	1		1				1	1	1	1	1
FG35	1	1	1	1	1		1	1		1	1				1
FP24	1			1			1	1	1	1		1	1	1	1
FP25		1	1	1	1		1	1		1			1	1	1
PF22			1	1	1		1	1	1	1			1	1	1
PF23		1	1	1			1	1		1	1		1	1	1
PF24	1	1	1	1					1	1	1	1		1	1
M26F21				1	1	1	1	1			1	1	1	1	1
M26F22	1					1	1	1		1	1	1	1	1	1
M27F24	1		1	1			1	1		1	1	1	1	1	
M27F25	1		1		1		1	1		1	1		1	1	1
CR26	1	1	1		1		1	1				1	1	1	1
