# Supplementary material for: Molecular Genetic Features of Polyploidization and Aneuploidization Reveal Unique Patterns for Genome Duplication in Diploid Malus
Source: PLoS One. 2012 Jan 10;7(1):e29449. doi: 10.1371/journal.pone.0029449 (PMC3254611; doi:10.1371/journal.pone.0029449)
Supplement: Table S21 — ‘2 n + 11 ’ aneuploid seedlings and their extra chromosomes. (PDF) [file pone.0029449.s022.pdf]

Progenies	The affected chromosomes														
	LG02	LG03	LG04	LG05	LG06	LG07	LG09	LG10	LG11	LG12	LG13	LG14	LG15	LG16	LG17
GF39	1	1		1			1	1		1	1	1	1	1	1
GF40	1		1	1	1		1	1		1	1		1	1	1
FG36	1	1	1	1	1		1	1			1	1		1	1
FG37	1		1	1	1		1	1	1	1			1	1	1
FP26		1	1	1			1	1	1	1	1		1	1	1
M26F23	1	1	1	1			1		1	1	1	1	1	1	
CR27			1	1	1	1	1	1			1	1	1	1	1
